# Revision of the European species of *Omphale* Haliday (Hymenoptera, Chalcidoidea, Eulophidae)

**DOI:** 10.3897/zookeys.232.3625

**Published:** 2012-10-25

**Authors:** Christer Hansson, Ekaterina Shevtsova

**Affiliations:** 1 Scientific Associate of the Natural History Museum, Cromwell Road, South Kensington, London SW7 5BD, United Kingdom; 2 Department of Biology, Lund University, Sölvegatan 35, Lund, Sweden

**Keywords:** Gall-midges, Cecidomyiidae, koinobionts, endoparasitoids, thelytoky, female-biased sex-ratios, morphological characters, male genitalia, phallobase, aedeagus, wing interference patterns, WIPs, taxonomy, synonymy, new combinations, neotype, identification key

## Abstract

The European species of *Omphale* Haliday (Eulophidae: Entedoninae) are revised. The revision includes 37 species, of which eleven are newly described and the remaining 26 species are redescribed. The species are classified into six species groups, with six unplaced species. All species are fully diagnosed and thoroughly illustrated. Identification keys are provided for females and males. Two new morphological features to aid classification and identification are introduced: male genitalia and wing interference patterns (WIPs). The former has been used successfully in the classification of New World *Omphale* and the latter is used for the first time in a taxonomic revision. Male genitalia in *Omphale* have considerable interspecific variation, an unusual trait among chalcidoid Hymenoptera, and are demonstrated to be useful for classification of species and species-groups, and they also possess the only autapomorphy for *Omphale*. WIPs are useful to help separate some species, but cannot be used to define either the genus or species groups. Distributional data are compiled for each species and suggest a pan-european distribution for most species. Gall-midges are the known hosts for 14 species, and the absence of host overlap between species suggests that host specialization is a driving force for speciation. Several *Omphale* species are known only from females, or have a strong female biased sex ratio, suggesting thelytokous development. Apart from the 37 species included in this revision, the status for nine additional species (names) in species group *aetius* remain unsolved. For nomenclatorial stability, a **neotype** is designated for *Eulophus lugens* Nees (= *Omphale lugens* (Nees)). *Elachestus obscurus* Förster and *Derostenus sulciscuta* Thomson are transferred from *Holcopelte* to *Omphale* **comb. n.** *Derostenus radialis* Thomson and *Achrysocharella americana* Girault are synonymized with *Omphale theana* (Walker), and *Omphale teresis* Askew is synonymized with *Omphale phruron* (Walker), **syn. n. **The status of genus *Pholema* Graham is revised as it is removed from synonymy with *Omphale* and instead synonymized with *Neochrysocharis* Kurdjumov, **syn. n.,** and the type species for *Pholema*, *Pholema microstoma* Graham, is transferred to *Neochrysocharis*, **comb. n.** *Eugerium orbatum* Szelényi, previously transferred to *Omphale*, is synonymized with *Asecodes congruens* (Nees), **syn. n.**

## Introduction

The genus *Omphale* Haliday is one of the largest genera of Entedoninae (Chalcidoidea: Eulophidae), with 259 species (Noyes 2012). It is a cosmopolitan group with uneven levels of knowledge from different parts of the World. The most recent taxonomic studies on this group are from the Americas (Hansson 1996b, 1997, 2004). Other parts of the World, except Europe, are very poorly known. Though originally described from Europe, the *Omphale* fauna from Europe has never been subject to a comprehensive taxonomic revision. Graham was the first (1959, 1963) to study European *Omphale* from an extensive taxonomic/nomenclatural aspect, but his focus was on northern Europe and mainly on the United Kingdom. Graham (1963) included 25 species, of which six were newly described. Subsequently six new species have been described (Graham 1970, Gijswijt 1976, Szelényi 1978, Askew 2003), bringing the total to 31 European *Omphale* species.

The keys, species descriptions and classification in Graham are exclusively based on externomorphological characters. As *Omphale* species are very plain and possess relatively few such characters, of which several show a high intraspecific variation, the species are difficult to distinguish. This sparsity of characters makes *Omphale* one of the more difficult genera of Entedoninae to classify at the species level, and it was consequently one of the few genera in Europe that had not been studied comprehensively. The poor knowledge of this genus in Europe is further demonstrated by the fact that eleven new species are described in this paper. The new species are mainly from Sweden and the United Kingdom, two of the most well investigated countries in the World as far as the fauna of Hymenoptera and *Omphale* is concerned.

The relatively few available externomorphological characters have been an impediment for the classification of this group. However, later taxonomic studies from the New World *Omphale* (Hansson 1996b, 1997, 2004) have included new characters from male genitalia, which have not been applied to the European species. As opposed to the majority of Eulophidae genera where the phallobase and aedeagus – the two parts of male genitalia – show little interspecific variation, species of *Omphale* frequently display species-specific as well as species-group specific features in these structures. As well, wing interference patterns (WIPs), a new character present in minute winged insects discovered by Shevtsova et al. (2011), have been useful in the classification of some eulophid groups but were never investigated in *Omphale*. These new characters enhance the possibilities of determining species identities and relationships of European *Omphale*.

### Male genitalia

Male genitalia in many insect groups tend to evolve divergent forms relatively rapidly compared to other structures, and can display a formidable morphological diversity (Eberhard 2009). Male genitalia are therefore frequently the richest source of morphological characters in insects (e.g. Grimaldi and Engel 2005), with important characters on different taxonomic levels. Examples of this are commonly found in e.g. Diptera (Grimaldi and Nguyen 1999), Coleoptera (Hubweber and Schmitt 2009), and Lepidoptera (Sohn and Nishida 2011). However, in the Hymenoptera, male genitalia usually show little variation at both the species and genus level (Michener 1956). There are some exceptions to this among some of the smallest species of the Chalcidoidea, in the Aphelinidae (Viggiani and Battaglia 1984) and Trichogrammatidae (Nagarkatti and Nagaraja 1968, 1971; Viggiani 1971, Owen et al. 2007), where characters in male genitalia are used for the separation of species. Male genitalia in most Eulophidae conforms to the situation in most Chalcidoidea, having little variation and thus little information value (e.g. Graham 1987) – with some notable exceptions. The most striking exception is genus *Perditorulus* Hansson, a recently described group confined to the Americas with its main distribution in tropical America, and currently comprising close to a hundred described species. The original description of the genus (Hansson 1996a) included 33 species, all with species-specific genitalia, and with a remarkable morphological diversity of structure, unparalleled within the Chalcidoidea. A subsequent contribution to *Perditorulus* (Hansson 2004) has firmly established and expanded these initial findings. Another group with variation in male genitalia, and therefore with useful information for the classification, is *Omphale* (Hansson 1996b, 1997, 2004), which was one of the incentives for this study of the European species.

Male genitalia in Chalcidoidea are relatively simple, consisting of the phallobase and aedeagus (Snodgrass 1941) (Fig. 478). The phallobase is the most complicated structure, forming a semiopen tube made up from three parts, the basal ring and a pair each of parameral and volsellar plates. In Eulophidae, the basal ring is not visible, and the parameral and volsellar plates are completely fused. The parameres are continuations of the parameral plates. The volsellar plates are strengthened by a ridge, the volsellar ridge, that extends along the entire length of the plates, and at the apex of this ridge there is a lobe – the digitus (digitus volsellaris) – with (digital) spines at the apex. Inside the tubular phallobase lies the aedeagus, consisting of a larger apical part – the penis valves, and two long aedeagal apodemes.

Most genera belonging to the Entedoninae have a phallobase and an aedeagus as in Fig. 478, i.e. a phallobase with weak volsellar setae (vs), two digital spines (ds), and an aedeagus with very few structures. There is little variation in this “ground-plan” within the subfamily, and species of same genus and even species from different genera are more or less indistinguishable in this structure. However, male genitalia in European *Omphale* exhibit considerable variation in both the phallobase and the aedeagus (Figs 479–501). Male genitalia are thus an extremely useful structure for the classification and species discrimination in this group which is so deprived of other morphological characters.

### Wing interference patterns (WIPs)

Wing interference patterns (WIPs) occur in transparent wings with a very thin membrane as is usually found in small insects (Shevtsova et al. 2011). WIPs appear when the wings are viewed against a dark background and visualize uneven thickness of the wing membrane. These patterns have been used to define a newly described genus from the Neotropical region (Hansson 2011). Shevtsova and Hansson (2011) demonstrated the usefulness of WIPs to separate species in the genus *Achrysocharoides* Girault, where some species also displayed sexual dimorphism in these patterns. Consequently WIPs can be useful for the classification at both genus and species levels. For the first time, WIPs are included herein in a large revision.

Species of *Omphale* do not have sexually dimorphic WIPs and all WIPs illustrated here are from females. *Omphale* species do not display the elaborate WIPs found in some species of *Achrysocharoides*, many of which have distinct eye-catching spots in the forewing (Shevtsova and Hansson 2011), and there is no common pattern for the genus or species-groups. Nevertheless these patterns can hold important clues for the separation of species. For example, in the *salicis*-group, both *Omphale cornula* and *Omphale theana* have the forewing predominantly in one colour (Figs 99, 129), which separates them from the other species in the group which have multicoloured WIPs (Figs 77, 84, 114). *Omphale theana* and *Omphale cornula* can be separated quite easily from each other through their WIPs even though they are basically in one colour. In *Omphale cornula* the area just below the marginal vein is differently coloured (Fig. 99), while *Omphale theana* has the same colour over the entire wing surface (Fig. 129). Similarly, in the *admirabilis*-group *Omphale telephe* has a one-colour WIP with an easily recognizable narrow, curved and differently coloured band just below the marginal vein (Fig. 49), while the other species have multicoloured WIPs. The multicoloured WIPs in this group also have diagnostic features, as *Omphale admirabilis* and *Omphale breviventris* have wide diagonal bands across the forewing (Figs 21, 34), whereas *Omphale versicolor* has apical ½ of forewing in one colour and basal ½ with wide colour bands running more or less straight from marginal vein to the hind margin of the wing (Fig. 64). Both species in the *clypealis*-group also have multicoloured WIPs, but the pattern is much more distinct in *Omphale clypealis* (Fig. 327) compared to *Omphale parma* (Fig. 342). Alternately, in the *aetius*-group (Figs 269, 284, 297, 312) and in most species in *phruron*-group (Figs 145, 189, 219, 234, 247, 254), WIPs are less distinct for the species and more difficult to use for their separation.

### Materials and methods

Collection acronyms used in the text with the names of persons who assisted with the loan of material are given in parentheses:

BMNH the Natural History Museum, London, United Kingdom (S. Ryder, N. Dale-Skey Papilloud).

CH private collection of Christer Hansson.

HNHM Hungarian Natural History Museum, Budapest, Hungary (S. Csösz).

LUZM Lund University Zoological Museum, Sweden (R. Danielsson).

NHRS Swedish Museum of Natural History, Stockholm, Sweden (H. Vårdal).

NMID National Museum of Ireland (Natural History), Dublin, Ireland (it has not been possible to examine any material from this museum).

NMW Naturhistorisches Museum Wien, Austria (D. Zimmermann).

OUMNH Hope Entomological Collections, Oxford University Museum of Natural History, United Kingdom (J. Hogan).

RMNH Naturalis Biodiversity Center, Leiden, the Netherlands (W. Hogenes, M.J. Gijswijt).

USNM United States National Museum of Natural History, Washington, D.C., U.S.A.

ZISP Zoological Museum of the Zoological Institute of the Russian Academy of Sciences, Saint Petersburg, Russia (S. Belokobylski).

ZMUC Zoological Museum, University of Copenhagen, Denmark.

*Terminology* – Morphological terms follow Gibson (1997). Morphology is also illustrated in Hansson (2004) and http://www.neotropicaleulophidae.com . Terminology for male genitalia is from Snodgrass (1941) (Fig. 478). The length of the phallobase on the slide preparations is measured from the apex of the paramere to the base of the phallobase (Fig. 478).

*Specimen preparations and imaging* – Fresh material collected with a sweep-net were collected and held in 80% ethanol. Wet specimens were subsequently dried using a critical point drier and mounted on a rectangular card as described by Noyes (1982). Preparations of male genitalia were done according to Noyes (1982). Photos of these slides were made with a Nikon phase contrast microscope, using 20x magnification, and the drawings based on these slides made with Adobe Illustrator^©^ and prepared in Adobe Photoshop^©^. Colour photos were made from a Nikon SMZ1000 stereomicroscope and 5MP Nikon DS-L1 camera. To eliminate reflections from the metallic and shiny body the light source used was a dome-light, as described by Kerr et al. (2008). Photos were taken at different focus levels, and Helicon Focus Pro version 4.75 was used to merge these into a single image. The making of photos of wing interference patterns is described in detail by Shevtsova and Hansson (2011). SEM photos were made from uncoated specimens on their original cardboard mounting. This was possible to do in low vacuum mode on a JEOL^®^ JSM 5600LV SEM microscope.

## Systematics

### Omphale

Haliday

http://species-id.net/wiki/Omphale

Omphale Haliday, 1833:339. Type species: *Omphale salicis* Haliday, by monotypy.
Smaragdites Haliday, 1833:419. Type species: S. *admirabilis* Haliday, by monotypy. Synonymy by Graham 1963:240.
Secodes Förster, 1856:78, 81. Type species: *Secodes fagi* Förster, by monotypy. Synonymy by Graham 1963:240.
Holcopelte Förster, 1856:78. Type species: *Elachistus obscurus* Förster, 1841:40, by original designation. Synonymy by Hansson 2004:142.
Euderomyia Girault, 1913:176. Type species: *Euderomyia carlylei* Girault, by original designation. Synonymy by Bouček 1988:727.
Chrysocharoideus Ashmead, 1904:370. Type species: *Chrysocharis thoracicus* Ashmead, by original designation. Synonymy by LaSalle and Schauff 1992:12.
Chrysocharomyia Dodd, *in*Girault 1915:207. Type species: *Chrysocharomyia elongata* Girault, by original designation. Synonymy by Bouček 1988:727.
Paromphale Girault & Dodd, *in*Girault 1915:211-212. Type species: *Paromphale flavicorpus* Girault, by original designation. Synonymy by Bouček 1988:727.
Raphaelonia Girault, 1924:173. Type species: *Raphaelonia sulcatiscutum* Girault, by monotypy. Synonymy by Bouček 1988:727.
Eugerium Graham, 1959:202. Type species: *Cirrospilus isander* Walker, by original designation. Synonymy by Hansson 1996b:5.
Exodontomphale Bouček, 1984:65. Type species: *Exodontomphale taborskyi* Bouček, by original designation. Synonymy by Schauff 1991:61.

#### Diagnosis.

Clypeus delimited by grooves at least laterally (e.g. Figs 117, 257, 367, 378); sensilla ampullacea (peglike sensilla) asymmetric; pronotum reduced and usually not visible in dorsal view; occiput without median groove or fold between occipital margin and occipital foramen; male genitalia (Figs 478–501): phallobase with enlarged volsellar setae, paramere with one seta at apex, digitus with two spines (very rarely with one or three spines). The species groups used here are based mainly on the appearance of the male genitalia, and most species have specific characters in this structure. However, since the visualisation of this structure involves slide preparations, characters in the male genitalia have been avoided as much as possible in the identification key, but in a few cases, when this is the only way to separate species, they are used.

#### Identification.

There are no taxonomically or nomenclaturally updated keys to European genera of Eulophidae. The most recent keys are in Graham (1959) and Peck et al. (1964). They are useful for identification by keeping in mind that *Holcopelte* and *Eugerium* come out as separate genera in the keys. Another option is to use the key for Nearctic genera of Chalcidoidea (Schauff et al. 1997), but *Holcopelte* is treated as a genus separate from *Omphale*.

#### Description.

Flagellum with 2–3 small and discoid anelli; five flagellomeres, free or with apical 2–3 flagellomeres more or less fused, occasionally with a distinct club with apical flagellomeres fused and wider than proximal flagellomeres (degree of fusion of apical flagellomeres is in many cases difficult to assess, and varies intraspecifically). Sensilla ampullacea (peglike sensilla) asymmetric, long or short. Ventral sense area of male scape reaching along almost entire length of scape. Males with verticillate or scattered arrangement of setae. Mandibles usually endodont with two large teeth at apex and with one to several smaller teeth above large teeth. A few Nearctic species have exodont mandibles, but this feature has not been found in European species. Cly- peus entirely delimited, quadrangular to semicircular or triangular in shape. Lower clypeal margin usually arcuately protruding, in a few species straight. Lower frons usually with a cross-ridge reaching almost from eye to eye (see Hansson 1996b, fig. 1), cross-ridge is protruding and is not an edge resulting from the collapse of frons above the antennal toruli, cross-ridge missing in some species. Frontal suture between lower and upper frons present. Antennal scrobes either more or less broadly separated and never joining, or joining at or below frontal suture. Surface between antennal scrobes more or less raised to form an interscrobal ridge. Occipital margin rounded off, rarely with an edge or a carina. Without a weak median groove or fold from occipital margin down to occipital foramen.

Pronotum reduced and hardly visible in dorsal view. Mesoscutum usually with weak delimited notauli; midlobe of mesoscutum with two pair of setae, some non-European species either with one pair or without setae. Scutellum usually without grooves but occasionally with a median groove with variable strength and length, always with one pair of setae usually situated in posterior half. Dorsellum short, smooth, convex or flat, occasionally with strong sculpture. Transepimeral sulcus curved or straight. Propodeum usually smooth and shiny, without anteromedian pit or raised carinae, with or without a narrow transverse proximal groove; propodeal callus with two setae; petiolar foramen semicircular to triangular, usually wide to fit the wide petiole. Forewing with a narrow and bare costal cell; speculum open or closed below; radial cell bare or hairy; with or without one hair line from stigmal vein; postmarginal vein 0.2–2.1× as long as stigmal vein in European species. Legs usually long and slender.

Petiole short and wide, occasionally as long as wide, pale or dark. Gaster usually subsessile; ovate to lanceolate in female. The male genitalia display important characters useful in classification of the species groups, but also in the separation of species.

#### The species-groups.

Graham (1963) initially divided the European *Omphale* into four groups with two unplaced species. However, he did so without any motivation and the groups were not diagnosed. The species groups presented here largely follow the groups presented by Graham, but now defined by morphological characters, and due to the addition of species two groups have been added. Characters in the male genitalia are used extensively to group species and to separate these groups. Graham was not aware of such characters and some species have therefore been transferred from their original placement. The European species are here separated into six groups, and with six unplaced species. The unplaced species comprise species that do not fit in any of the species groups, and that do not share features with each other to form species groups on their own.

#### Biology.

Hosts or host plant/fungi associations are known for 16 of the 37 European species (Table 1), and in all cases gall midges (Diptera: Cecidomyiidae) are the target group. Host records from North America (Hansson 1996b) and Central America (Hansson 2004) support the findings for European species, i.e. that *Omphale* species are exclusively parasitoids on gall midges. Dziurzynski (1961) investigated the biology of *Omphale lugens* in Poland (given as *Secodes coactus* in the Dziurzynski publication). *Omphale lugens* is a koinobiont primary endoparasitoid and the female oviposits in the second instar larvae of its gall midge host, *Mikiola fagi*, which induces galls on the upper surface of leaves of beech (*Fagus sylvatica*). This parasitoid is solitary or gregarious with up to ten individuals per host. If more than one larva is present per host either the same female lays more than one egg per host, or more than one female oviposits in the same host larva. However, because of the high mortality among gregarious larvae eventually only one parasitoid per host larva will complete its development. The parasitoid speeds up the development of its host, indicated by a faster growth rate in parasitized gall midge larvae, as compared to non-parasitized larvae. There does not seem to be additional, abnormal, instars in the host, just an acceleration of growth. The gall midge larva is killed before pupation by the feeding of the last instar parasitoid larva. *Omphale lugens* has four larval instars and pupates inside the empty larval skin of its host, still remaining inside the gall.

**Table 1.** 

*Omphale aethiops*	*Dasineura epilobii* (Diptera: Cecidomyiidae) on *Chamaenerion angustifolium* (Gijswijt 1976), collected investigating flowers of *Silene dioica* with cecidomyiid larvae (Askew 2003); two of the female specimens from Sweden (in RMNH) have been reared from *Dasineura traili* (new record), a gall midge associated with *Ranunculus*.
*Omphale brevis*	*Cystiphora taraxaci* (Diptera: Cecidomyiidae) (Gijswijt 1976), *Cystiphora sonchi* on *Sonchus palustris* (Vidal 1993); *Cystiphora sanguinea* on *Hieracium sabaudum* (Askew 2003).
*Omphale chryseis*	*Contarinia medicaginis* (Diptera: Cecidomyiidae) (Bouček and Askew 1968) - endoparasitoid of larvae (Královič 1964).
*Omphale clymene*	*Dasineura pyri* (Diptera: Cecidomyiidae) (new record).
*Omphale clypealis*	*Dasineura brassicae* (Diptera: Cecidomyiidae) (e.g. Gijswijt 1976).
*Omphale erginnus*	Associatedwith bracket fungi, possibly on a Cecidomyiidae (Diptera) (Hansson 1996b).
*Omphale grahami*	*Dasineura trifolii*, *Dasineura glechomae* (Diptera: Cecidomyiidae) (Gijswijt 1976).
*Omphale isander*	From *Mycodiplosis* sp. (Diptera: Cecidomyiidae) feeding on leaf rust on *Populus* (Kamijo 1986).
*Omphale lugens*	*Mikiola fagi* (Dziurzynski 1961), *Contarinia tiliarum* & *Dasyneura alni* (Gijswijt 1976), *Placochela nigripes* (new record), all hosts are Diptera: Cecidomyiidae.
*Omphale lugubris*	Associated with *Picea* (Gijswijt 1976), but not reared from host.
*Omphale obscura*	*Dasineura viciae* (Diptera: Cecidomyiidae) (De Stefani 1905); unidentified budgall on *Galium mollugo* (Bouček and Askew 1968).
*Omphale phruron*	*Dasineura pyri* (Diptera: Cecidomyiidae) (Gijswijt 1976)
*Omphale rubigus*	*Trigonodiplosis* sp. (Diptera: Cecidomyiidae) on *Vicia cracca* (Bouček and Askew 1968).
*Omphale salicis*	*Contarinia lentis* (Diptera: Cecidomyiidae) (probable record) (Szelényi 1944), *Contarinia loti* (Gijswijt 1976); *Contarinia vincetoxici* (new record).
*Omphale incognita*	*Geocrypta galii* (Diptera: Cecidomyiidae) on *Galium* spp.
*Omphale euphorbiae*	*Bayeria capitigena* (Diptera: Cecidomyiidae) on *Euphorbia esula*.

The only other *Omphale* species for which more detailed biological information is available is *Omphale clypealis*. This species is an important biological control agent against the brassica pod midge (*Dasineura brassicae*) on rape (*Brassica napus*), and has thus been the subject of biological investigation (Williams 2003). Similar to *Omphale lugens*, *Omphale clypealis* is a koinobiont endoparasitoid. Females oviposit into mature gall midge larvae in their pod gall, and the parasitoid larva is inside the host when it leaves the gall and burrows into the ground for pupation. The pupa is subsequently killed by the parasitoid. The sex ratio for *Omphale clypealis* is strongly female biased, and Murchie (1996) reared 97–100% females from samples of the brassica pod midge in the U.K. Material from this species that has been available for this investigation, 147 females and three males, suggests the same female bias. This skewed sex ratio indicates that *Omphale clypealis* is thelytokous, as are possibly some other *Omphale* species, such as *Omphale rubigus* where males have never been found, and *Omphale theana* in which very few males are recorded.

If data from these two species are transferrable, *Omphale* species are koinobiont endoparasitoids, possibly solitary – at least with only one surviving parasitoid per host. Little is known about the host specificity of *Omphale* species. However, known host records suggest host specialization because there are no overlap between the species. The host record for *Omphale phruron*, *Dasineura pyri* – same host as in *Omphale clymene*, is possibly based on a misidentification of the parasitoid. The specimens of *Omphale phruron* from *Dasineura pyri* have not been available for this investigation and as the species in this group are difficult to identify misidentification cannot be ruled out.

#### Distribution.

Even though several parts of the World are very poorly investigated or not investigated at all, existing records show that *Omphale* is a cosmopolitan genus, known from all zoogeographical regions (Noyes 2012). As can be seen from previous investigations (Hansson 1996b, 1997, 2004), and this investigation (Figs 502–538), the species have a very large distribution. Many European species are distributed throughout Europe, and the more limited distribution in some species is possibly due to lack of distributional data. Five European species, *Omphale acamas*, *Omphale erginnus*, *Omphale salicis*, *Omphale theana*, *Omphale versicolor*, are also found in North America (Hansson 1996b), and one of these (*Omphale erginnus*) is distributed south to Central America (Hansson 2004).

### Key to the European species

Females

**Table:** 

1	Clypeus paler and less metallic than surrounding parts of frons (Figs 324, 339), *and* antenna with a 3-segmented clava (Figs 329, 344)	2
–	Clypeus with same colour as surrounding parts of frons, either metallic or non-metallic and pale; antenna *usually* with clava 1–2-segmented	3
2(1)	Clypeus yellowish white (Fig. 324); forewing with 10–16 admarginal setae; gaster short, MM/LG = 0.7–0.8	*Omphale clypealis* (Thomson)
–	Clypeus brown with weak to strong metallic tinges (Fig. 329); forewing with 6–10 admarginal setae; gaster long, MM/LG = 0.4–0.5	*Omphale parma* sp. n.
3(1)	Frons above frontal suture and vertex smooth and shiny (e.g. Figs 396, 411); occipital margin with an edge or a carina (Fig. 7)	4
–	Frons and vertex with at least some parts reticulate; occipital margin *usually* rounded (Fig. 8)	9
4(3)	Propodeum smooth, without longitudinal carinae (e.g. Fig. 394)	5
–	Propodeum with longitudinal median and/or lateral carinae (e.g. Figs 376, 409)	7
5(4)	Notauli as distinct smooth deep grooves in posterior ⅔, grooves gradually widening towards posterior part (Fig. 394)	*Omphale rubigus* (Walker)
–	Notauli with at least outer margin indistinct, smooth or reticulate (Figs 361, 365)	6
6(5)	Forecoxae dark brown, mid- and hind coxae yellowish brown (Fig. 384); gaster 1.2–1.6× as long as mesosoma	*Omphale rossica* sp. n.
–	All coxae pale brown (Fig. 356); gaster 1.9× as long as mesosoma	*Omphale erugata* sp. n.
7(4)	Notauli as distinct and narrow grooves of same width in posterior ½ (Fig. 409); petiole quadrangular with anterior part drawn out to a sharp edge that covers the propodeal nucha (Fig. 409)	*Omphale sulciscuta* (Thomson)
–	Notauli more or less triangular, with posterior part wider than anterior part (Fig. 361, 376); petiole without anterodorsal extension	8
8(7)	Forewing with admarginal setae from marginal vein and with radial cell bare (Fig. 359); gaster elongate (Fig. 356), 1.9× as long as mesosoma	*Omphale erugata* sp. n.
–	Forewing with admarginal setae arising mainly from wing membrane and with radial cell hairy (Fig. 374); gaster 1.0–1.4× as long as mesosoma (Fig. 369)	*Omphale obscura* (Förster)
9(3)	Forewing with admarginal setae arising from ventral surface of marginal vein (occasionally with a few of the apical setae arising from wing membrane just behind marginal vein) (Fig. 10), and with radial cell *usually* bare (Fig. 11), part between lower margin of eye and mouth opening *usually* with more or less strong sculpture (strigose–reticulate) (Fig. 5)	10
–	Forewing with admarginal setae predominantly arising from ventral surface of wing membrane just behind marginal vein (Fig. 9), and with radial cell *usually* setose (Fig. 12), also with part between lower margin of eye and mouth opening *usually* smooth or with very weak sculpture (Fig. 6)	39
10(9)	Forewing with stigmal vein enlarged and with membrane around stigmal vein infuscate (Fig. 471); completely yellow non-metallic species (Fig. 468)	*Omphale melina* Yefremova & Kriskovich
–	Forewing stigmal vein not so enlarged; with at least some body-part metallic or dark brown	11
11(10)	Marginal fringe of forewing very long, with setae along outer margin 0.3× as long as width of wing (Fig. 443); ventral margin of clypeus with median tooth (Fig. 447)	*Omphale isander* (Walker)
–	Marginal fringe of forewing short, at most 1.5× as long as width of wing; ventral margin of clypeus without median tooth	12
12(11)	Forewing speculum open below (Fig. 14)	13
–	Forewing speculum closed below (Fig. 13)	16
13(12)	Antenna with flagellomeres 1–2 pale brown, 3–5 yellow (Fig. 422); coxae white	*Omphale aceris* (Erdös)
–	Antennal flagellomeres dark brown; coxae yellowish brown, pale brown or metallic	14
14(13)	Coxae and femora bluish green metallic, tibiae and tarsi dark brown (Fig. 336); antenna with 3-segmented clava (Fig. 344)	*Omphale parma* sp. n.
–	Coxae and femora completely or predominantly non metallic, tibiae and tarsi yellow to yellowish brown; antenna with 1 or 2-segmented clava	15
15(14)	Femora pale brown and hind coxa predominantly metallic (Fig. 243); gaster longer, 1.5–1.6× as long as mesosoma (Fig. 243); postmarginal vein 1.5× as long as stigmal vein (Fig. 246)	*Omphale sti* sp. n.
–	Femora yellow and hind coxa yellowish brown (Fig. 154); gaster shorter, 1.4–1.5× as long as mesosoma (Fig. 154); postmarginal vein 1.8–2.0× as long as stigmal vein (Fig. 159)	*Omphale clymene* (Walker)
16(12)	Lateral pronotum, prosternum and legs citron yellow to yellowish white (Figs 15, 210, 473)	17
–	At least some of above-mentioned body parts brown to metallic	19
17(16)	Lower frons metallic (Fig. 18)	*Omphale admirabilis* (Haliday)
–	Lower frons yellow non metallic (Figs 211, 474)	18
18(17)	Gaster elongate (Fig. 473), 2.0× as long as mesosoma	*Omphale ochra* sp. n.
–	Gaster shorter (Fig. 210), 1.4–1.5× as long as mesosoma	*Omphale matrana* Erdös
19(16)	Upper frons and vertex smooth and shiny (Figs 396, 397)	*Omphale rubigus* (Walker)
–	Upper frons and/or vertex partly to completely reticulate, reticulation sometimes very weak	20
20(19)	Occipital margin with a sharp carina (Fig. 433); setae on vertex long, seta situated in middle of ocellar triangle as long as distance between posterior ocelli (Fig. 433)	*Omphale erginnus* (Walker)
–	Occipital margin usually rounded, but occasionally with an edge; setae on vertex short, seta in middle of ocellar triangle distinctly shorter than distance between posterior ocelli	21
21(20)	Vertex and mesoscutum with distinctly different colours: vertex bright green metallic to golden green and mesoscutum dark brown with metallic tinges (Figs 351, 353)	*Omphale brevibuccata* Szelényi
–	Vertex and mesoscutum with different combination of colours, both usually metallic	22
22(21)	Lower frons and face yellow to yellowish white (Figs 211, 474)	23
–	Lower frons and face predominantly metallic (e.g. Fig. 281)	24
23(22)	Vertex dark brown with metallic tinges (Fig. 211); mesoscutum dark brown with golden and green metallic tinges (Fig. 212), midlobe with one pair of setae (posterior pair) (Fig. 207)	*Omphale matrana* Erdös
–	Vertex yellowish brown (Fig. 474); mesoscutum with anterior ½ golden green with a median yellowish brown stripe, posterior ½ yellowish brown (Fig. 475), midlobe with two pairs of setae (Fig. 398)	*Omphale ochra* sp. n.
24(22)	Head with part between lower margin of eye and mouth margin smooth (Fig. 6)	25
–	Head with part between lower margin of eye and mouth margin reticulate–strigose (Fig. 5)	26
25(24)	Forecoxa black to dark brown metallic, mid- and hind coxae yellowish brown (Fig. 278); gaster 2.0× as long as mesosoma	*Omphale connectens* (Walker)
–	All coxae predominantly yellowish white (Fig. 293); gaster very long (Fig. 293), 2.5× as long as mesosoma	*Omphale dolichura* sp. n.
26(24)	All coxae dark and usually metallic	27
–	At least some coxa predominantly pale (yellowish white to yellowish brown)	31
27(26)	Forewing with stigmal vein large and abruptly narrowed at base (Fig. 458); flagellum short (pedicel + flagellum 1.3× as long as distance between eyes) (Fig. 453)	*Omphale lugens* (Nees)
–	Forewing with stigmal vein either not enlarged or gradually narrowing off towards base; flagellum longer, at least 1.4× as long as distance between eyes	28
28(27)	Gaster with 7^th^ tergite 1.3–4.5× as long as width at base	*Omphale salicis* Haliday
–	Gaster with 7^th^ tergite 0.5–0.8× as long as width at base	29
29(28)	Flagellomeres 2–4 ventrally with two sets of setae, one attached subbasally and one attached subapically (Fig. 1)	*Omphale euphorbiae* sp. n.
–	Flagellomeres 2–4 ventrally with one set of setae attached subbasally and reaching beyond apex of flagellomere attached to (Fig. 2)	30
30(29)	Antenna long, pedicel + flagellum 1.8× as long as distance between eyes; gaster with 7^th^ tergite 0.6× as long as width at base; mesoscutum *frequently* bicoloured (Fig. 229)	*Omphale phruron* (Walker)
–	Antenna short, pedicel + flagellum 1.5× as long as distance between eyes; gaster with 7^th^ tergite 0.3× as long as width at base; mesoscutum unicoloured (Fig. 94)	*Omphale cornula* sp. n.
31(26)	Forewing with 2–5 admarginal setae	32
–	Forewing with 6–12 admarginal setae	34
32(31)	Gaster with 7^th^ tergite 1.3–4.3× as long as width at base	*Omphale theana* (Walker)
–	Gaster with 7^th^ tergite 0.3–1.1× as long as width at base	33
33(32)	Gaster with 7^th^ tergite 0.3× as long as width at base; flagellomeres 2–4 ventrally with two sets of setae, one set attached subbasally and one set attached subapically (Fig. 1)	*Omphale lydia* sp. n.
–	Gaster with 7^th^ tergite 0.6–1.1× as long as width at base; flagellomeres 2–4 ventrally with one set of setae attached subbasally and reaching beyond apex of flagellomere attached to (Fig. 2)	*Omphale acuminata* Gijswijt
34(31)	Gaster with 7^th^ tergite 1.1–4.2× as long as width at base; fore- and midcoxae yellowish white with base brown, hind coxa predominantly metallic (Fig. 78)	*Omphale chryseis* Graham
–	Gaster with 7^th^ tergite 0.1–0.4× as long as width at base; all coxae with same colour (usually yellowish brown, sometimes with base of all coxae brown to metallic)	35
35(34)	Frons smooth and shiny (Fig. 257); coxae yellowish white and femora yellowish brown to dark brown (Fig. 248)	*Omphale tenuicornis* sp. n.
–	Frons at least partly reticulate; coxae usually darker, but *if* coxae are yellowish white then femora are also yellowish white	36
36(35)	Femora yellowish white to yellowish brown (Fig. 138)	*Omphale brevis* Graham
–	Femora predominantly pale brown to dark brown	37
37(36)	Flagellomeres 1–3 ventrally with two sets of setae, one attached close to base and one attached in apical ½ of each flagellomere (Fig. 1)	*Omphale nitens* Graham
–	Flagellomeres 1–3 ventrally with one set of setae attached close to base of each flagellomere and reaching beyond apex of flagellomere attached to (Fig. 2)	38
38(37)	Coxae completely pale (Fig. 138)	*Omphale brevis* Graham
–	Coxae with base brown to metallic (Fig. 183)	*Omphale incognita* sp. n.
39(9)	Head with part between lower margin of eye and mouth opening strigose–reticulate (Fig. 5)	40
–	Head with part between lower margin of eye and mouth opening smooth (Fig. 6)	41
40(39)	Forewing with speculum open below (Fig. 14)	*Omphale versicolor* (Nees)
–	Forewing with speculum closed below (Fig. 13)	*Omphale telephe* (Walker)
41(39)	Gaster very long (Fig. 293), 2.5× as long as mesosoma, and legs predominantly pale	*Omphale dolichura* sp. n.
–	Gaster shorter, at most 2.2× as long as mesosoma, and then with legs predominantly dark	42
42(41)	Legs entirely yellow to white	43
–	With at least parts of some coxa or femur infuscate, brown or metallic	44
43(42)	Lateral pronotum and prosternum yellow to yellowish white (Fig. 15); gaster elongate (Fig. 15), 1.4–1.6× as long as length of mesosoma	*Omphale admirabilis* (Haliday)
–	Lateral pronotum and prosternum metallic (Fig. 30); gaster short (Fig. 30), 1.0× as long as length of mesosoma	*Omphale breviventris* Graham
44(42)	Flagellomeres 1–3 ventrally with only one set of setae attached subbasally reaching beyond apex of flagellomere attached to (Fig. 2); 2-segmented clava solid, with two apical flagellomeres completely fused (Fig. 314); legs dark (Fig. 306); small species (1.1–1.6 mm)	*Omphale lugubris* Askew
–	Flagellomeres 1–3 ventrally with at least two sets of setae, one attached subbasally and one subapically (Fig. 1); 2-segmented antennal clava less solid, i.e. with a distinct constriction between the two flagellomeres; leg colour and size variable	45
45(44)	Gaster very long (Fig. 263), 2.0–2.2× as long as mesosoma, with 7th tergite 1.5–2× as long as basal width and with posterior ⅔ thickly setose (Fig. 277); coxae and femora dark (Fig. 263); body bronze-black (Figs 263, 264); large species (1.9–3.1 mm)	*Omphale aethiops* Graham
–	Gaster less than 2× as long as mesosoma, 7^th^ tergite usually not longer than basal width and sparsely setose; coxae and femora paler; body usually more brightly coloured, e.g. metallic green or purple, brassy or coppery; length usually less than 2mm	remaining species in species-group *aetius* Females and males of the following species are impossible to separate through their morphology: *Omphale acamas* (Walker), *Omphale aetius* (Walker), *Omphale betulicola* Graham, *Omphale coilus* (Walker), *Omphale epaphus* (Walker), *Omphale grahami* Gijswijt, *Omphale phaola* (Walker), *Omphale varipes* (Thomson) (see notes at the end of this paper)

Males

**Table:** 

1	Clypeus paler and less metallic than surrounding parts of frons (Figs 325, 340)	2
–	Clypeus as metallic, or pale as surrounding parts of frons	3
2(1)	Clypeus yellowish white (Fig. 325); forewing with 10–16 admarginal setae	*Omphale clypealis* (Thomson)
–	Clypeus brown with weak to strong metallic tinges (Fig. 340); forewing with 6–10 admarginal setae	*Omphale parma* sp. n.
3(1)	Head strongly sclerotized and not collapsing after death, frons above frontal suture and vertex smooth and shiny (Figs 382, 415); occipital margin with an edge or a carina (Figs 383, 416)	4
–	Head usually collapsing after death (unless critical point-dried), with at least some part on frons above frontal sture and vertex reticulate; occipital margin *usually* rounded	5
4(3)	Coxae dark brown to black (Fig. 402), hind coxa with apical part usually paler; petiole quadrangular with anterior part drawn out to a sharp edge that covers the propodeal nucha (as in Fig. 409)	*Omphale sulciscuta* (Thomson)
–	Coxae yellowish white to yellowish brown (Fig. 369), forecoxa occasionally pale brown; petiole without anterodorsal extension	*Omphale obscura* (Förster)
5(3)	Scape long, slender at base and expanded above the middle (Figs 27, 55, 70)	6
–	Scape shorter, wide at base and widest in the middle	8
6(5)	Forewing speculum open below (Fig. 63)	*Omphale versicolor* (Nees)
–	Forewing speculum closed below (Figs 20, 48)	7
7(6)	Lateral parts of pronotum, prepectus and entire legs citron yellow to yellowish white (Fig. 15)	*Omphale admirabilis* (Haliday)
–	Lateral parts of pronotum and prepectus metallic, coxae yellowish brown to dark and metallic, femora pale brown to dark brown (Fig. 43)	*Omphale telephe* (Walker)
8(5)	Forewing with admarginal setae arising from ventral surface of marginal vein (occasionally with a few of the apical setae arising from wing membrane just behind marginal vein) (Fig. 10), and with radial cell *usually* bare (Fig. 11), also part between lower margin of eye and mouth opening *usually* with more or less strong sculpture (Fig. 5) (strigose-reticulate)	9
–	Forewing with admarginal setae predominantly arising from ventral surface of wing membrane just behind marginal vein (Fig. 9), and with radial cell *usually* setose (Fig. 12), also with part between lower margin of eye and mouth opening *usually* smooth or with very weak sculpture (Fig. 6)	24
9(8)	Marginal fringe of forewing very long, e.g. setae along outer margin are 0.3× as long as width of wing (Fig. 443); ventral margin of clypeus with median tooth (Fig. 451)	*Omphale isander* (Walker)
–	Marginal fringe of forewing short; ventral margin of clypeus without median tooth	10
10(9)	Forewing speculum open below (Fig. 14)	11
–	Forewing speculum closed below (Fig. 13)	12
11(10)	Coxae and femora bluish green metallic, tibiae and tarsi dark brown (as in Fig. 336)	*Omphale parma* sp. n.
–	Coxae yellowish brown, femora, tibiae and tarsi yellow (as in Fig. 154)	*Omphale clymene* (Walker)
12(10)	Occipital margin with a sharp carina (Fig. 437); setae on vertex long, with seta situated in middle of ocellar triangle as long as distance between posterior ocelli (Fig. 437)	*Omphale erginnus* (Walker)
–	Occipital margin usually rounded, but occasionally with an edge; setae on vertex short, with seta situated in middle of ocellar triangle shorter than distance between posterior ocelli	13
13(12)	Forecoxa black to dark brown metallic, mid- and hind coxae yellowish brown (as in Fig. 278); head with part between lower margin of eye and mouth margin shiny with very weak sculpture and partly smooth, to completely smooth (Fig. 291)	*Omphale connectens* (Walker)
–	Coxae with different combination of colours; head with part between lower margin of eye and mouth margin reticulate–strigose (Fig. 5)	14
14(13)	All coxae dark and usually metallic	15
–	At least some coxa predominantly pale (yellowish white to yellowish brown)	21
15(14)	Forewing with stigmal vein large and abruptly narrowed at base (as in Fig. 458); genitalia very distinct and different from the other species in the genus, aedeagus with very long apodemes (Fig. 501) and phallobase with lower part of digitus drawn out with a spine at apex (Fig. 501)	*Omphale lugens* (Nees)
–	Forewing with stigmal vein either not enlarged, or gradually narrowing off towards base; genitalia very different from *Omphale lugens*	16
16(15)	Flagellomeres 1–4 with a basal whorl of setae but without setae apical to whorl (Fig. 3)	17
–	Flagellomeres 1–4 with a basal whorl of setae and with setae apical to whorl (Fig. 4)	18
17(16)	Scutellum metallic with sides non metallic yellowish brown (Fig. 95)	*Omphale cornula* sp. n.
–	Scutellum entirely metallic (Fig. 230)	*Omphale phruron* (Walker)
18(16)	Femora dark brown with apices yellowish brown (as in Fig. 183); phallobase with digitus 1.4× as long as wide (Fig. 489)	*Omphale incognita* sp. n.
–	Femora completely dark; phallobase with digitus transverse	19
19(18)	Tibiae yellowish brown (as in Fig. 108); phallobase with volsellar setae crossed in slide preparations (Fig. 484)	*Omphale salicis* Haliday
–	Tibiae brown; phallobase with volsellar setae never crossed in slide preparations	20
20(19)	Thoracic dorsum bright golden green (Fig. 171); forewing with 8–11 admarginal setae	*Omphale euphorbiae* sp. n.
–	Thoracic dorsum golden red to golden purple with green metallic tinges (Fig. 230); forewing with 6–7 admarginal setae	*Omphale phruron* (Walker)
21(14)	Hind coxa predominantly metallic (as in Fig. 78)	*Omphale chryseis* Graham
–	Hind coxa predominantly yellowish white or yellowish brown	22
22(21)	Forewing with 2–5 admarginal setae; phallobase with volsellar setae crossed in slide preparations (Fig. 485)	*Omphale theana* (Walker)
–	Forewing with 5–11 admarginal setae; phallobase with volsellar setae never crossed in slide preparations (Figs 486, 491)	23
23(22)	Flagellum long and slender (Fig. 260), 1^st^ flagellomere 4.7× as long as wide; genitalia with aedeagus long and slender (Fig. 491), about 6× as long as wide	*Omphale tenuicornis* sp. n.
–	Flagellum stout (Fig. 151), 1^st^ flagellomere 1.9× as long as wide; genitalia with aedeagus stout (Fig. 486), about 3.5× as long as wide	*Omphale brevis* Graham
24(8)	Legs long and slender (as in Fig. 263), tarsi especially so, hind tarsus 0.9× as long as hind tibia and 1.1× as long as hind femur; dark and large species (1.9 mm)	*Omphale aethiops* Graham
–	Legs shorter, hind tarsus 0.8× or less as long as hind tibia and as long as hind femur; usually paler and smaller species	25
25(24)	Small species (1.3 mm); femora dark brown; mesosoma dark brown with metalic tinges (Fig. 308)	*Omphale lugubris* Askew
–	Usually larger species; femora pale; mesosoma usually with brighter colours	remaining species of *aetius*-group - inseparable (see above in the key to females, couplet 45)

### Species treatments

#### Species group *admirabilis*

**Diagnosis**. Forewing with admarginal setae arising from both membrane and from marginal vein, or predominantly from the membrane, radial cell hairy (Fig. 48) or with upper ½ bare (Fig. 20); clypeus metallic with sides straight (e.g. Fig. 67); head with a frontal cross-ridge (e.g. Fig. 24); flagellomeres with short and asymmetric sensilla; male flagellomeres with scattered setae. Male genitalia: phallobase with volsellar setae late- rally flattened and attached at or above base of volsellar ridges (Figs 479–481); aedeagus wide with apex narrowed, terminal parts of aedeagal apodemes fused (Figs 479–481).

##### Omphale admirabilis

(Haliday)

http://species-id.net/wiki/Omphale_admirabilis

Figures 4 15 –29 479 502

Smaragdites admirabilis Haliday, 1833:419. Holotype male in OUMNH, examined.
Entedon admirabilis (Haliday), Reinhard (1858).
Omphale admirabilis (Haliday), Graham (1959).
Omphale admirabilis (Haliday), Graham (1963).

###### Material.

**Type material.** **Holotype** male, type no. 616 in OUMNH. **Additional material.** 25♀ 13♂: France 4♀ (BMNH, RMNH), Sweden 1♀ 4♂ (CH, NHRS), United Kingdom 20♀ 9♂ (BMNH).

###### Diagnosis.

Lateral pronotum, prosternum and legs citron yellow to yellowish white (Fig. 15), in male also with gena yellowish white; thoracic dorsum brilliant green metallic to brilliant golden red (Figs 16, 17), with very fine engraved reticulation (Figs 22, 26), hence shiny; postmarginal vein 2× as long as stigmal vein (Fig. 20); male scape long (Fig. 27), with almost ½ of length reaching above level of vertex.

###### Description.

*Female*. Length of body 1.5–2.0 mm. Antenna with scape with outer surface white, inner surface with basal ½ white and apical ½ dark brown; pedicel and flagellum dark brown (Fig. 15); pedicel + flagellum 1.8× as long as distance between eyes; first flagellomere 1.2× as long as second (Fig. 23), and 1.0× as wide as second flagellomere; flagellomeres 1–4 with scattered short setae (Fig. 23); clava 2-segmented (Fig. 23). Face golden green (Fig. 18), with very weak striae (Fig. 24); clypeus golden green, smooth, rectangular, 1.7× as wide as high; gena dark brown with golden tinges, to purple metallic; lower frons bright green metallic, with raised reticulation, smooth below level of toruli; interscrobal area with raised reticulation; antennal scrobes join frontal suture separately; frontal suture V-shaped; upper frons golden red with very weak reticulation, shiny; vertex golden green to golden red, smooth outside ocellar triangle, with engraved reticulation inside triangle (Fig. 25). Occipital margin rounded (Fig. 25).

Mesoscutum bright golden green, or golden red (Fig. 16), with engraved reticulation (Fig. 22), midlobe with two pairs of setae; notauli as indistinct impressions in posterior ½. Scutellum bright golden green, or golden red (Fig. 16), with engraved reticulation (Fig. 22); 1.1× as long as wide, with anterior margin smoothly curved forwards. Axillae blue to green metallic (Fig. 16). Dorsellum green metallic (Fig. 16), smooth and flat (Fig. 22), 0.4× as long as wide, and 0.6× as long as length of median propodeum. Lateral pronotum and propleuron citron yellow to yellowish white (Fig. 15); prepectus green metallic, in three specimens citron yellow or yellowish white; acropleuron citron yellow to yellowish white; mesepisternum golden green with upper part citron yellow to yellowish white; upper mesepimeron golden green to golden red; lower mesepimeron dark brown with metallic tinges; transepimeral sulcus very weakly curved forwards. Propodeum green metallic (Fig. 16), smooth (Fig. 22), with an anteromedian fovea; propodeal callus with two setae. Legs citron yellow to yellowish white (Fig. 15); midleg with first tarsomere 0.3× as long as length of tarsus. Forewing transparent (Fig. 20), veins yellowish brown and setae dark brown; speculum closed; admarginal setae 8–11, arising from both marginal vein and from membrane behind vein; radial cell bare in upper ½, setose in lower ½; postmarginal vein 2.0× as long as stigmal vein; stigmal vein slightly enlarged. Hind wing (Fig. 20) transparent, apex rounded. Forewing WIP (Fig. 21) with apical ⅓ blue, basal ⅔ with diagonal bands in yellow, magenta and blue.

Petiole dark brown to yellow. Gaster golden with green metallic tinges, to golden red with first tergite green metallic, smooth, elongate and 1.4–1.6× as long as length of mesosoma; 7^th^ tergite 0.1× as long as length of gaster.

*Male*. Length of body 1.1–1.6 mm. Features as in female except as follows. Antenna with scape with outer surface yellowish white, inner surface with basal ½ white and apical ½ green metallic, narrow in basal ½ and then rapidly expanding towards apex (Fig. 27), elongate with almost ½ the length above the level of vertex; pedicel and flagellum pale brown; pedicel + flagellum 3.3× as long as distance between eyes; flagellomeres 1–4 with scattered setae; clava 1-segmented. Face greenish blue metallic (Fig. 19), smooth; clypeus greenish blue metallic, smooth, rectangular, 2.0× as wide as high; gena yellowish white; lower frons golden to bright golden green (Fig. 19), smooth (Fig. 28); interscrobal area smooth; upper frons red metallic, smooth; vertex golden red.

Pronotum, propleuron and prepectus yellowish white. Mesoscutum golden red (Fig. 17). Scutellum golden red (Fig. 17) to golden green; 1.2× as long as wide. Axillae green metallic (Fig. 17). Dorsellum green metallic with red tinges (Fig. 17), smooth and slightly convex to almost flat (Fig. 26). Acropleuron yellowish white; mesepisternum golden green. Propodeum golden with green and red metallic tinges (Fig. 17). Legs yellowish white. Forewing transparent, veins and setae dark brown; admarginal setae 8–9, arising from both marginal vein and membrane below vein.

Petiole yellowish white. Gaster with first tergite golden red, remaining tergites dark brown with metallic tinges, with a pale spot across median part of tergites 1–2, 1.0–1.1× as long as length of mesosoma. Phallobase and aedeagus as in Fig. 479.

**Figures 1–14.**
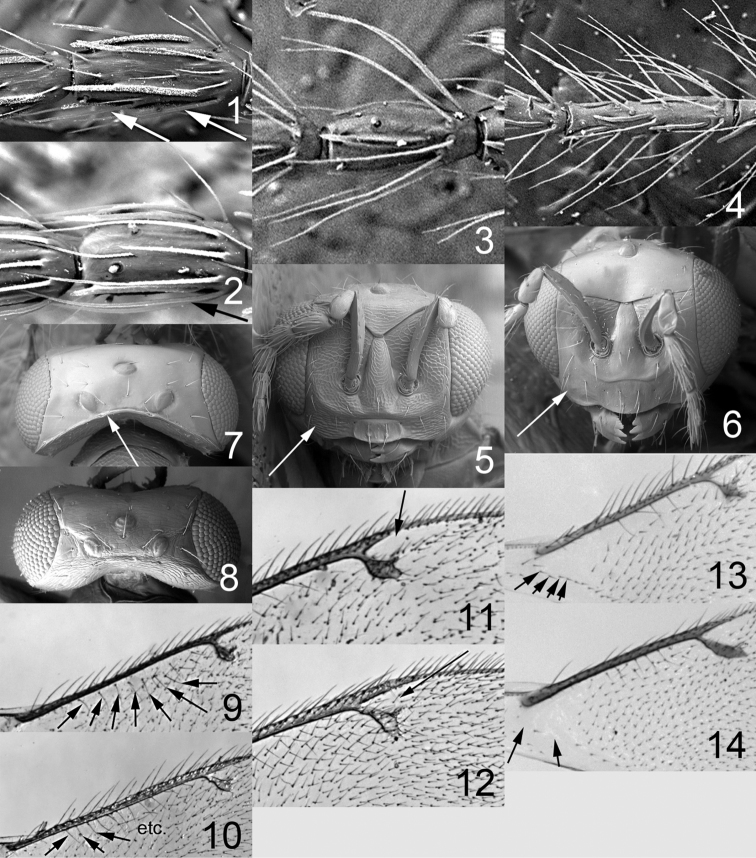
*Omphale* spp.: **1** *Omphale chryseis*, 2nd flagellomere, female **2** *Omphale lugens*, 2nd flagellomere, female **3** *Omphale phruron*, 2nd flagellomere, male **4** *Omphale admirabilis*, 2nd flagellomere, male **5** *Omphale connectens*, head in frontal view, female **6** *Omphale lugens*, head in frontal view, female **7** *Omphale sulciscuta*, vertex, female **8** *Omphale acuminata*, vertex, female **9** *Omphale sulciscuta*, inner part of forewing, arrows point at admarginal setae **10** *Omphale connectens*, inner part of forewing, arrows point at admarginal setae **11** *Omphale salicis*, part of forewing, arrow points at bare radial cell **12** *Omphale aethiops*, part of forewing, arrow points at hairy radial cell **13** *Omphale phruron*, base of forewing, speculum closed **14** *Omphale clypealis*, base of forewing, speculum open.

**Figures 15–21.**
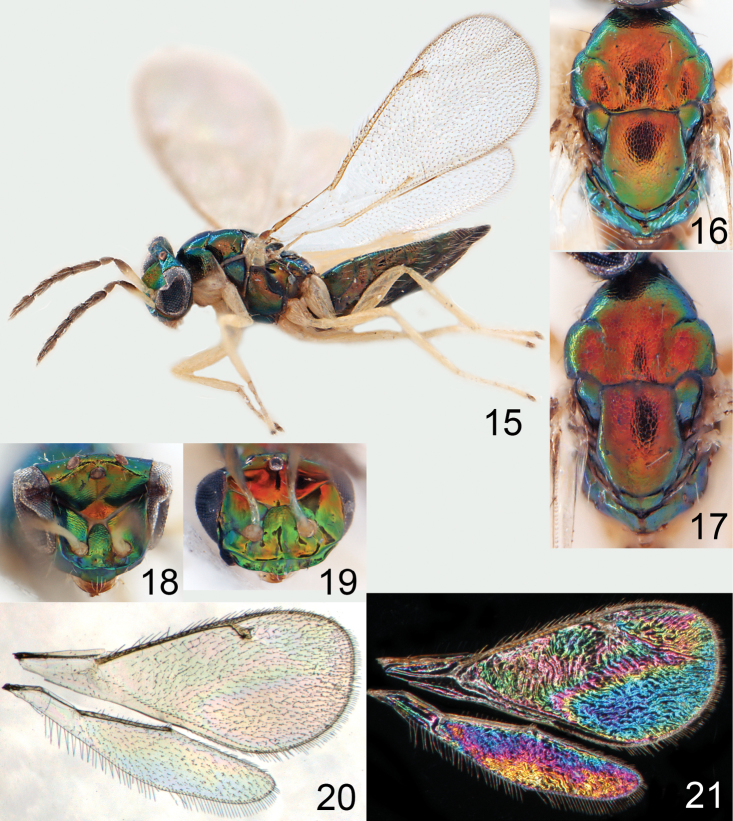
*Omphale admirabilis*: **15** habitus in lateral view, female, length of specimen 1.8 mm **16** thoracic dorsum, female **17** thoracic dorsum, male **18** head in frontal view, female **19** head in frontal view, male **20** transparent wings, female **21** wing interference patterns, female.

**Figures 22–29.**
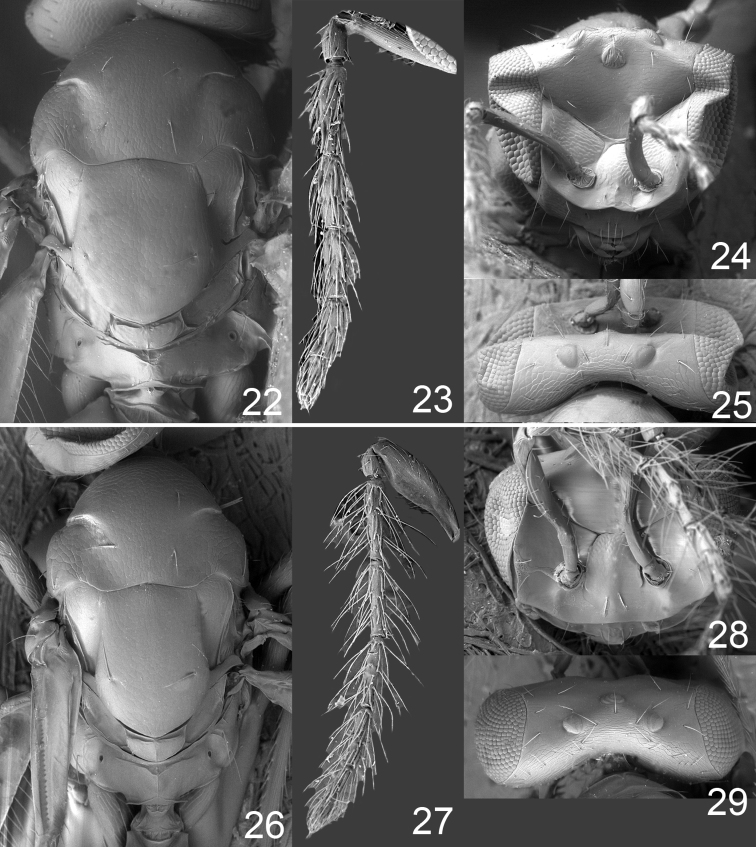
*Omphale admirabilis*: **22** thoracic dorsum, female **23** antenna, female **24** head in frontal view, female **25** vertex, female **26** thoracic dorsum, male **27** antenna, male **28** head in frontal view, male **29** vertex, male.

###### Hosts. 

Unknown.

###### Distribution.

Austria (Bouček and Askew 1968), Czech Republic (Bouček and Askew 1968), France (**new record**), Sweden (Hedqvist 2003), United Kingdom (Haliday 1833) (Fig. 502).

##### Omphale breviventris

Graham

http://species-id.net/wiki/Omphale_breviventris

Figures 30 –38 503

Omphale breviventris Graham, 1970:207. Holotype female in OUMNH, examined.

###### Material.

**Type material.** **Holotype** female, type no. 1222 in OUMNH, paratype 1♀ (BMNH). **Additional material.** 5♀: Germany 2♀ (RMNH), Netherlands 1♀ (RMNH), United Kingdom 2♀ (BMNH).

###### Diagnosis.

**Figures 30–34.**
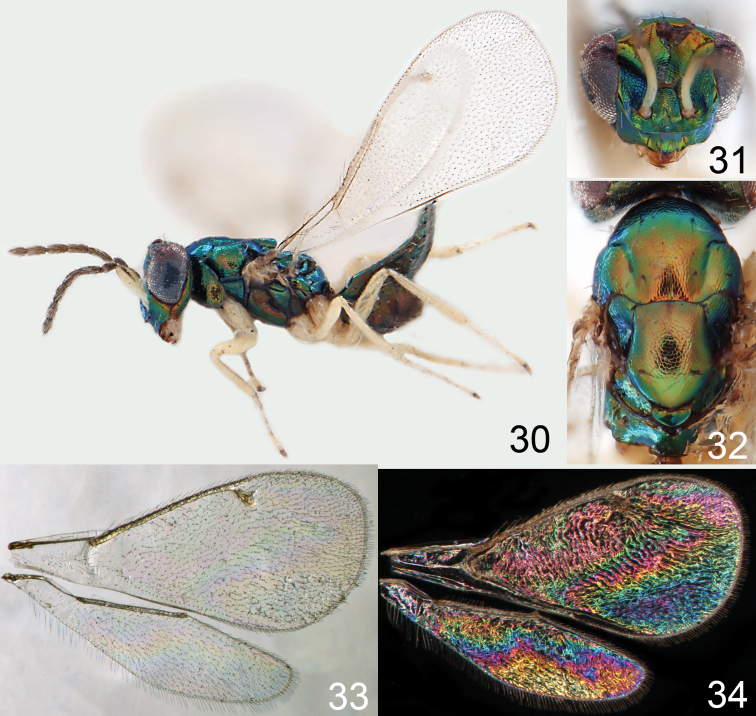
*Omphale breviventris*, female: **30** habitus in lateral view, length of specimen 1.7 mm **31** head in frontal view **32** thoracic dorsum **33** transparent wings **34** wing interference patterns.

Gaster short, as long as length of mesosoma; scape and legs yellowish white to citron yellow (Fig. 30); head and mesosoma bright metallic (green with golden tinges, golden red) (Figs 31, 32).

###### Description.

*Female*. Length of body 1.4–1.9 mm. Antenna with scape yellowish white to citron yellow with apical ½ of dorsal edge dark brown; pedicel dark brown; flagellum dark brown with golden tinges; pedicel + flagellum 2.1× as long as distance between eyes; first flagellomere 1.2× as long and 1.0× as wide as second flagellomere (Fig. 36); flagellomeres with scattered short setae; clava 2-segmented. Face bright green metallic with golden tinges (Fig. 31), strigose; clypeus bright green metallic with golden tinges, smooth, rectangular, 1.4× as wide as high; gena bright green metallic with golden tinges; lower frons bright green metallic with golden tinges, with raised reti-culation (Fig. 37), subtorular area smooth; interscrobal area with raised reticulation; antennal scrobes join frontal suture separately; frontal suture V-shaped; upper frons bright green metallic with golden tinges, with engraved reticulation; vertex golden red, with engraved reticulation. Occipital margin rounded (Fig. 38).

**Figures 35–42.**
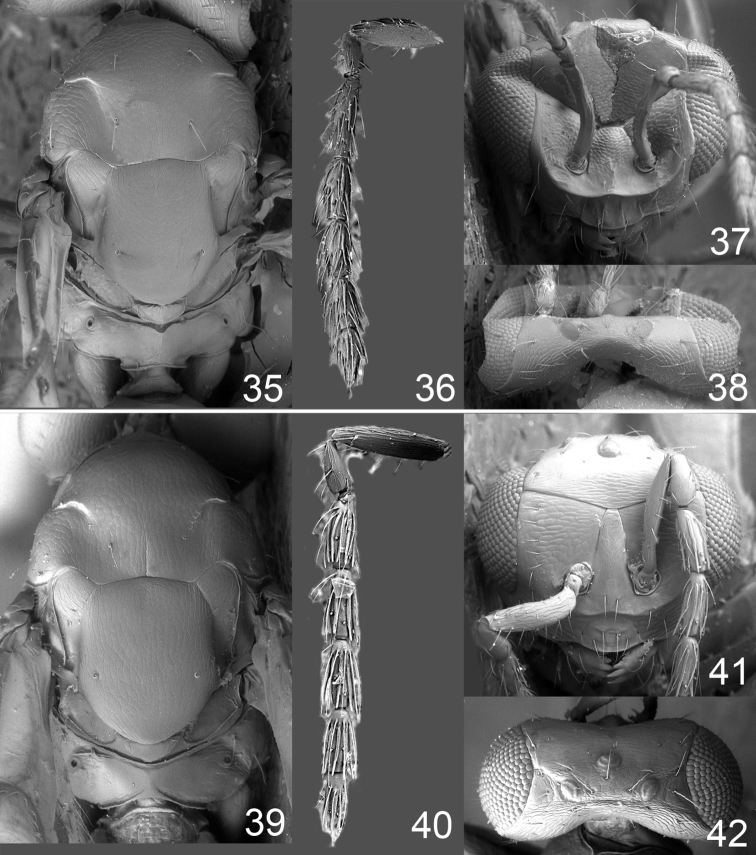
*Omphale* spp., females:**35–38**. *Omphale breviventris*: **35** thoracic dorsum **36** antenna **37** head in frontal view **38** vertex **39–42**. *Omphale acuminata*: **39** thoracic dorsum **40** antenna **41** head in frontal view **42** vertex.

Mesoscutum golden with green tinges (Fig. 32), to golden red, with engraved reticulation (Fig. 35), midlobe with two pairs of setae; notauli as indistinct impressions. Scutellum golden with lateral and posterior margins green metallic (Fig. 32), to golden red, with engraved reticulation (Fig. 35); 1.2× as long as wide, with anterior margin smoothly and weakly curved forwards. Axillae green metallic (Fig. 32). Dorsellum bluish green (Fig. 32), smooth and flat with raised lateral and posterior margins (Fig. 35), 0.5× as long as wide, and 0.6× as long as length of median propodeum. Lateral pronotum, propleuron and prepectus green metallic with golden tinges (Fig. 30); acropleuron dark brown; mesepisternum golden red; upper mes-epimeron green metallic with golden tinges; lower mesepimeron dark brown with golden tinges; transepimeral sulcus very weakly curved forwards. Propodeum green metallic with golden tinges (Fig. 32), smooth with a small fovea anteromedially (Fig. 35); propodeal callus with two setae. Legs yellowish white to citron yellow, hind coxa brown at very base; midleg with first tarsomere 0.3× as long as length of tarsus. Forewing transparent, veins yellowish white and setae dark brown (Fig. 33); speculum closed; admarginal setae 8–12, arising from both marginal vein and wing membrane; radial cell setose; postmarginal vein 1.0× as long as stigmal vein; stigmal vein slender. Hind wing transparent, apex rounded (Fig. 33). Forewing WIP (Fig. 34) as in *Omphale admirabilis* with apical ⅓ blue, and basal ⅔ with diagonal bands in yellow, magenta and blue.

Petiole dark brown. Gaster golden with green tinges, short, 1.0× as long as length of mesosoma; 7^th^ tergite 0.06× as long as length of gaster.

*Male*. Unknown.

###### Hosts.

Unknown.

###### Distribution.

Germany (Graham 1970), Netherlands (Gijswijt 1976), United Kingdom (Graham 1970) (Fig. 503).

##### Omphale telephe

(Walker)

http://species-id.net/wiki/Omphale_telephe

Figures 4 43 –57 480 504

Entedon telephe Walker, 1839:75. Lectotype male in BMNH, examined.
Omphale telephe (Walker), Graham (1963).

###### Material.

**Type material.** Lectotype male, type no. 5.2715 in BMNH. **Additional material.** 134♀ 21♂: Germany 1♀ (RMNH), Netherlands 3♀ (RMNH), Sweden 6♀ 3♂ (CH, LUZM, NHRS, RMNH), United Kingdom 123♀ 19♂ (BMNH).

###### Diagnosis.

Row of admarginal setae with some, or most, from membrane behind marginal vein and radial cell setose (Fig. 48); postmarginal vein 0.7–0.8× as long as stigmal vein, stigmal vein usually enlarged (but narrow in some specimens); face between lower corner of eye and clypeus strigose (Fig. 52); female flagellum with a strong constriction between flagellomeres 4 and 5, clava thus 1-segmented (Fig. 51); transepimeral sulcus straight; scutellum usually golden purple and mesoscutum golden with green tinges (Figs 44, 45); forewing WIP with a narrow elongate area with bands in yellow, magenta and blue just behind marginal vein (Fig. 49).

###### Description.

*Female*. Length of body 1.3**–**2.0 mm. Antenna with scape yellowish brown with dorsal margin dark brown; pedicel and flagellum dark brown; pedicel + flagellum 1.8× as long as distance between eyes; first flagellomere 1.2× as long and 1.1× as wide as second flagellomere (Fig. 51); flagellomeres 2–4 ventrally with two sets of setae, one attached at base and one in apical ⅓ of flagellomere; clava 1-segmented. Face green metallic (Fig. 46), strigose (Fig. 52); clypeus green metallic, smooth or with weak and coarse longitudinal striae, semicircular and 1.9× as wide as high; gena purple metallic or golden purple; lower frons golden green, with raised reticulation; interscrobal area golden, smooth with weak reticulation in upper ⅓; antennal scrobes join on frontal suture; frontal suture V-shaped; upper frons golden green or golden purple with weak reticulation; vertex golden purple to golden red, inside ocellar triangle with engraved weak reticulation, outside triangle smooth (Fig. 53). Occipital margin rounded (Fig. 53).

**Figures 43–49.**
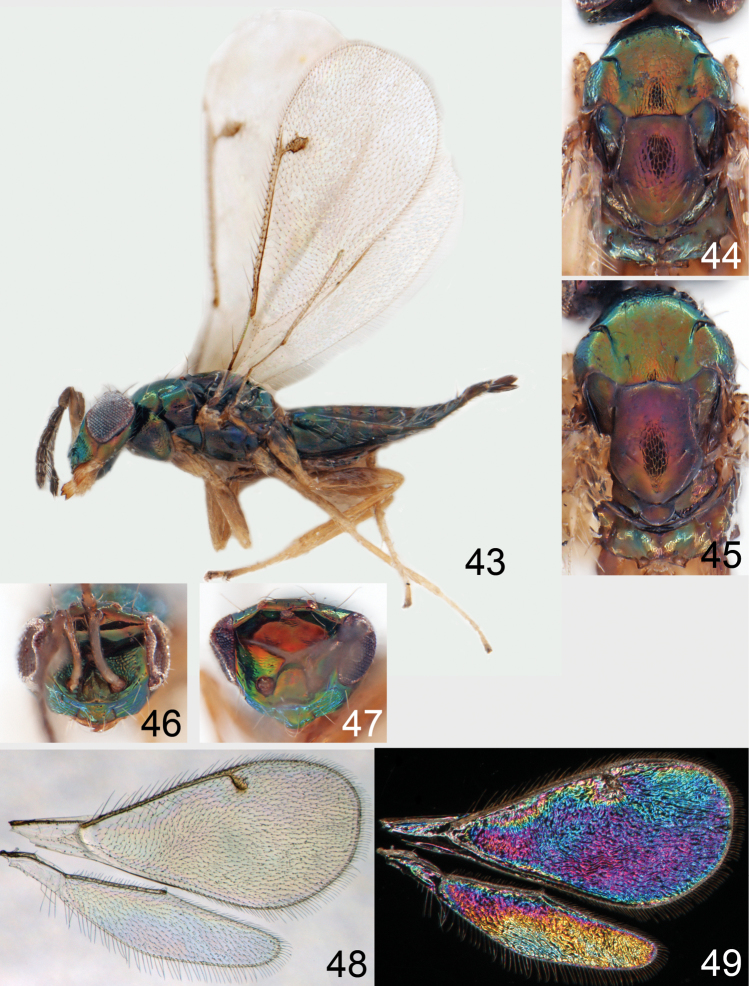
*Omphale telephe*: **43** habitus in lateral view, female, length of specimen 1.7 mm **44** thoracic dorsum, female **45** thoracic dorsum, male **46** head in frontal view, female **47** head in frontal view, male **48** transparent wings, female **49** wing interference patterns, female.

**Figures 50–57.**
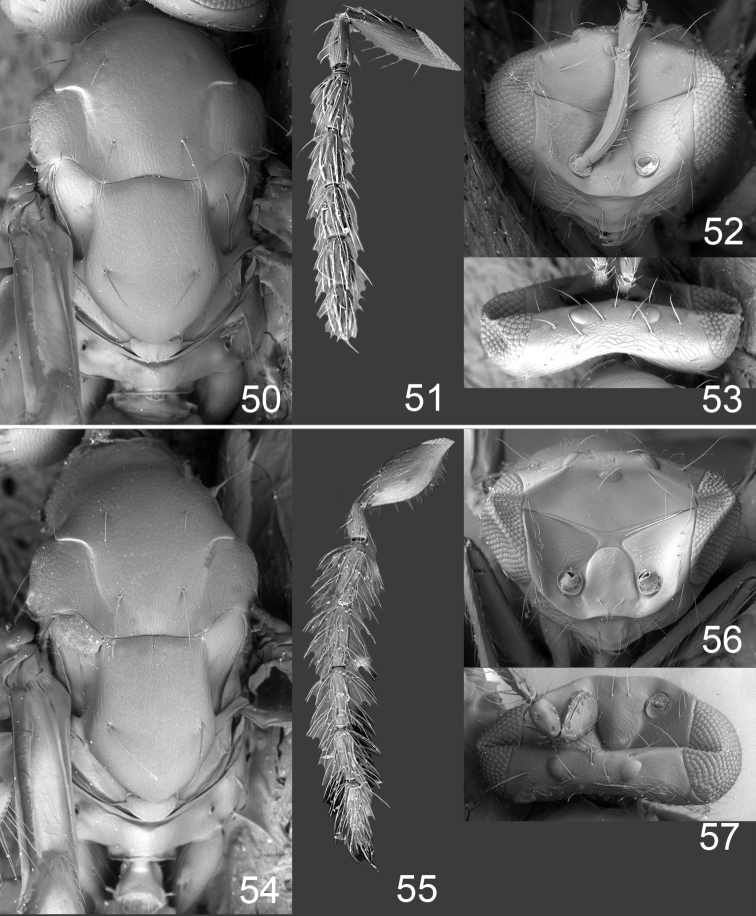
*Omphale telephe*: **50** thoracic dorsum, female **51** antenna, female **52** head in frontal view, female **53** vertex, female **54** thoracic dorsum, male **55** antenna, male **56** head in frontal view, male **57** vertex, male.

Mesoscutum golden with green tinges (Fig. 44), with engraved reticulation (Fig. 50), midlobe with two pairs of setae; notauli as indistinct impressions in posterior ½. Scutellum golden purple (Fig. 44) with engraved reticulation and with posterior margin smooth (Fig. 50); 1.1× as long as wide, with anterior margin smoothly curved forwards. Axillae golden purple (Fig. 44). Dorsellum golden purple (Fig. 44), smooth and slightly convex (Fig. 50), 0.5× as long as wide, and 0.8× as long as length of median propodeum. Lateral pronotum golden purple (Fig. 43); propleuron golden green; prepectus golden green; acropleuron dark brown; mes-episternum golden purple; upper mesepimeron blue metallic with purple metallic tinges; lower mesepimeron blue or green metallic; transepimeral sulcus straight. Propodeum golden green (Fig. 44), smooth (Fig. 50), in large specimens with weak reticulation; propodeal callus with two setae. Fore- and midcoxae yellowish brown with base pale brown, hind coxa with basal ½ dark brown with purple or green metallic tinges and apical ½ yellowish brown (Fig. 43), some large specimens with all coxae dark brown and metallic; femora pale brown to dark brown; tibiae yellowish brown; foretarsus dark brown, mid- and hind tarsi yellowish brown; midleg with first tarsomere 0.3× as long as length of tarsus. Forewing transparent, veins yellowish brown and setae dark brown (Fig. 48); speculum closed; admarginal setae 8–11, arising mainly from membrane below vein; radial cell setose; postmarginal vein 0.7–0.8× as long as stigmal vein; stigmal vein usually enlarged but sometimes long and slender. Hind wing transparent, apex rounded (Fig. 48). Forewing WIP (Fig. 49) magenta with narrow margin in blue, area just behind marginal vein with thin lines in yellow, magenta and blue.

Petiole yellowish brown to pale brown. Gaster with first tergite golden green, remaining tergites golden purple, elongate and 1.4–1.5× as long as length of mesosoma; 7^th^ tergite 0.1× as long as length of gaster.

*Male*. Length of body 1.4**–**1.8 mm. Features as in female except as follows. Antenna with scape golden green with basal ¼ yellowish white; pedicel + flagellum 2.5× as long as distance between eyes; flagellomeres 1–4 with scattered setae (Fig. 55). Face golden green with part close to eyes golden red (Fig. 47); clypeus golden green, with weak but coarse carinae, shiny, trapezoid; gena purple metallic; antennal scrobes join frontal suture separately (Fig. 56); upper frons golden red, with very weak reticulation and shiny; vertex golden red with purple metallic tinges.

Mesoscutum golden red to golden green (Fig. 45). Scutellum purple with posterior ⅓ golden (Fig. 45); 1.3× as long as wide. Axillae golden (Fig. 45). Dorsellum 0.7× as long as wide, and 0.7× as long as length of median propodeum. Entire lateral mesosoma purple metallic. Propodeum golden red with green metallic tinges (Fig. 45). Legs with coxae yellowish brown, hind coxa with basal 1/5 brown metallic; femora yellowish brown to dark brown; fore- and midtarsi dark yellowish brown, hind tarsus yellowish brown; admarginal setae 6–10; postmarginal vein 0.9–1.0× as long as stigmal vein.

Petiole brown. Gaster with first tergite golden, remaining tergites dark brown metallic, 1.1× as long as length of mesosoma. Phallobase and aedeagus as in Fig. 480.

###### Hosts.

Unknown.

###### Distribution.

Germany (**new record**), Netherlands (Gijswijt 1976), Sweden (Hansson 1991), United Kingdom (Walker 1839) (Fig. 504).

##### Omphale versicolor

(Nees)

http://species-id.net/wiki/Omphale_versicolor

Figures 58 –72 481 505

Eulophus versicolor Nees, 1834:169. Neotype female in LUZM, examined.
Entedon anthylla Walker, 1839:85. Lectotype female in BMNH, examined. Syno-nymized by Graham (1963:260).
Derostenus (Omphale) versicolor (Nees), Thomson (1878:269).
Omphale versicolor (Nees), Graham (1959:201).
Omphale versicolor (Nees), Graham (1963:260).
Omphale versicolor (Nees), Hansson (1996b:17).

###### Material.

**Type material.** Neotype female of *Eulophus versicolor*, type no. 118:1 in LUZM, and lectotype female of *Entedon anthylla*, type no. 5.2039 in BMNH. **Additional material.** 173♀ 65♂: France 9♀ (BMNH, RMNH), Germany 1♀ (RMNH), Netherlands 2♀ (RMNH), Slovenia 1♀ (RMNH), Sweden 111♀ 23♂ (BMNH, CH, LUZM, NHRS, RMNH), United Kingdom 53♀ 42♂ (BMNH).

###### Diagnosis.

**Figures 58–64:**
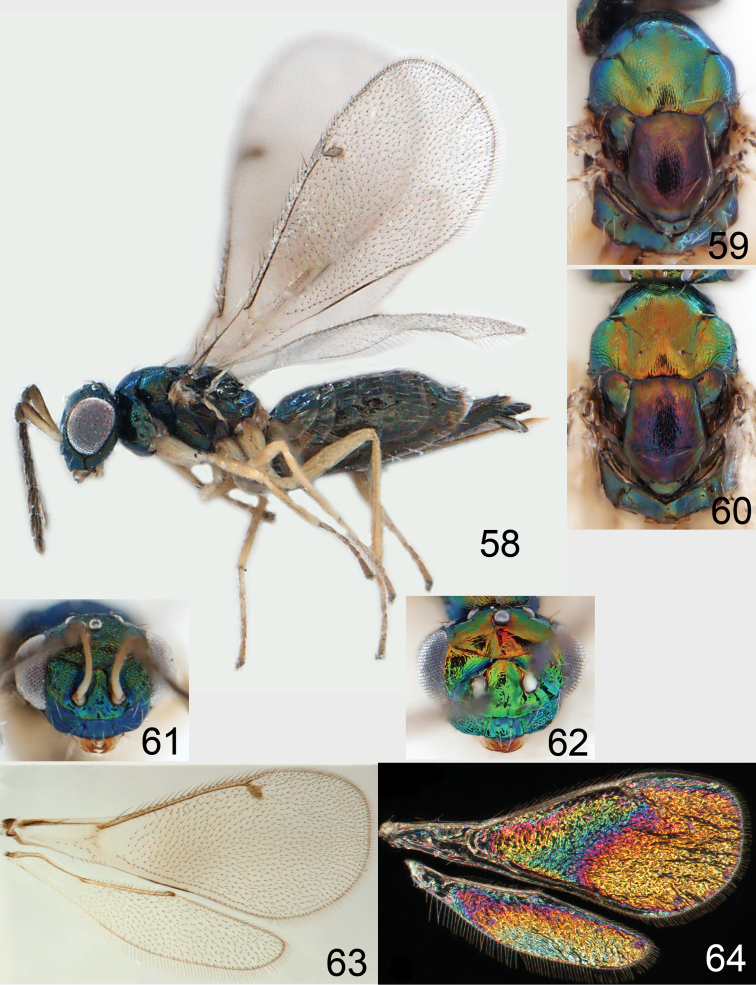
. *Omphale versicolor*: **58** habitus in lateral view, female, length of specimen 2.0 mm **59** thoracic dorsum, female **60** thoracic dorsum, male **61** head in frontal view, female **62** head in frontal view, male **63** transparent wings, female **64** wing interference patterns, female.

Forewing with open speculum, enlarged stigmal vein and with radial cell hairy (Fig. 63); scutellum usually metallic purple (Figs 59, 60); upper mesepimeron metallic purple (Fig. 58); legs yellow to yellowish white (Fig. 58); antennal scrobes join frontal suture separately (Figs 67, 71); clypeus with same colour as face (Figs 61, 62); face strigose (Figs 67, 71); female antenna with basal ½ of first flagellomere as wide as 2^nd^ but then gradually narrows off towards apex (Fig. 66); male scape elongate, reaching above level of vertex, narrow in basal ⅓ and enlarged in apical ⅔ (Fig. 70).

###### Description.

*Female*. Length of body 1.4–2.3 mm. Antenna with scape with outer surface yellowish brown, inner surface with basal ⅓ yellow to yellowish white and apical ⅔ pale brown; pedicel and flagellum dark brown and metallic; pedicel + flagellum 2.0× as long as distance between eyes; first flagellomere 1.1–1.3× as long as second flagellomere, at base with same width as second and gradually narrowing towards apex (Fig. 66); flagellomeres 1–3 with ventral setae short, basal setae not reaching apex of flagellomere attached to; longitudinal sensilla on flagellomeres scattered; clava 2-segmented. Face green metallic with golden tinges, to bluish green metallic (Fig. 61), strigose (Fig. 67); clypeus green metallic, to bluish green metallic, smooth, rectangular to trapezoid, 1.2× as wide as high; gena golden red with green tinges, to golden green; lower frons green metallic with golden tinges, with raised reticulation, subtorular area smooth, interscrobal area with raised reticulation; antennal scrobes join frontal suture separately; frontal suture U-shaped; upper frons golden red with green tinges, to golden green with engraved reticulation; vertex golden red with green tinges, with engraved reticulation (Fig. 68). Occipital margin rounded (Fig. 68).

**Figures 65–72.**
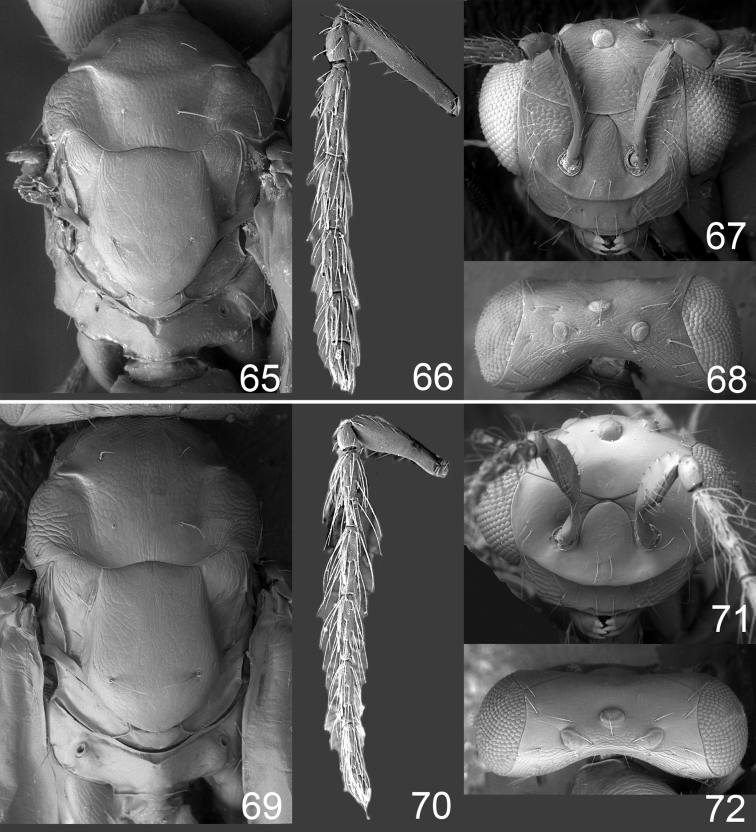
*Omphale versicolor*: **65** thoracic dorsum, female **66** antenna, female **67** head in frontal view, female **68** vertex, female **69** thoracic dorsum, male **70** antenna, male **71** head in frontal view, male **72** vertex, male.

Mesoscutum green metallic with golden tinges (Fig. 59), to bluish green metallic, with engraved reticulation (Fig. 65), midlobe with two pairs of setae; notauli as indistinct impressions in posterior ½. Scutellum purple metallic (Fig. 59) to golden red, occasionally golden green, with engraved and weak reticulation (Fig. 65); 1.2× as long as wide, with anterior margin smoothly curved forwards. Axillae green metallic with golden tinges (Fig. 59). Dorsellum green metallic (Fig. 59), smooth and flat (Fig. 65), 0.3× as long as wide, and 0.5–0.6× as long as length of median propodeum. Lateral pronotum green metallic with golden and red tinges (Fig. 58); propleuron greenish blue metallic; prepectus green metallic to golden red; acropleuron dark brown; mesepi-sternum golden, to golden red; upper mesepimeron brown with purple metallic shine; lower mesepimeron brown metallic; transepimeral sulcus curved forwards. Propodeum green metallic (Fig. 59), smooth with a fovea anteromedially (Fig. 65); propodeal callus with two setae. Foreleg yellow to yellowish white with base of coxa pale brown and tarsus dark brown (Fig. 58); midleg yellow to yellowish white, with tarsomere 3 and 4 brown to yellowish brown, first tarsomere 0.3× as long as length of tarsus; hind leg yellow to yellowish white with base of coxa brown and tarsus pale brown with tarsomere 4 brown to yellowish brown. Forewing transparent, veins yellowish brown and setae dark brown (Fig. 63); speculum open; admarginal setae 10–19, arising mainly from wing membrane; radial cell setose; postmarginal vein 0.8× as long as stigmal vein; stigmal vein enlarged. Hind wing transparent, apex rounded (Fig. 63). Forewing WIP (Fig. 64) with apical ½ yellow, basal ½ with wide bands in magenta, blue and yellow.

Petiole pale brown to yellowish brown. Gaster with tergite 1 green metallic, tergites 2–7 brown with green, red and golden tinges, elongate and 1.7–2.0× as long as length of mesosoma; 7^th^ tergite 0.2× as long as length of gaster; 7^th^ tergite with hairless basal part smooth and apical hairy part sculptured.

*Male*. Length of body 1.1–1.7 mm. Features as in female except as follows. Antenna (Fig. 70) with scape long, apical ⅓ reaching above level of vertex, narrow in basal ⅓ and yellowish white, expanded in apical ⅔ which is brown non metallic on outer surface and brown with strong green metallic shine on inner surface; pedicel with same metallic shine as inner upper surface of scape; flagellum brown with golden tinges; pedicel + flagellum 2.7× as long as distance between eyes; first flagellomere 1.2–1.3× as long as second; flagellomeres with scattered setae; clava 1-segmented. Face green metallic (Fig. 62), sometimes with blue tinges; clypeus green metallic, rectangular; gena golden red; lower frons green metallic with golden tinges and upper ⅓ bronze, with raised weak reticulation (Fig. 71); interscrobal area with raised weak reticulation in upper ⅓; upper frons bright golden red and smooth.

Mesoscutum green metallic (Fig. 60), midlobe with strong yellowish red shine and sidelobes with golden tinges. Scutellum purple metallic (Fig. 60), occasionally golden green or bluish green metallic; 1.3–1.4× as long as wide. Axillae green metallic with purple tinges (Fig. 60). Dorsellum brown with green metallic tinges (Fig. 60), 0.5× as long as wide, and 0.5–0.7× as long as length of median propodeum. Propleuron and prepectus green metallic; mesepisternum golden red, sometimes with green tinges; upper mesepimeron purple metallic; lower mesepimeron brown purple metallic tinges. Propodeum brown with green metallic tinges (Fig. 60). Legs yellowish-white, tarsi pale brown. Forewing veins and setae dark brown; admarginal setae 16–19, arising mainly from wing membrane; postmarginal vein 1.1× as long as stigmal vein.

Petiole pale brown. Gaster with tergite 1 green metallic, tergites 2–7 brown, 1.1–1.2× as long as length of mesosoma. Phallobase and aedeagus as in Fig. 481.

###### Host.

Unknown.

###### Distribution.

Czech Republic (Bouček and Askew 1968), France (**new record**), Germany (Nees 1834), Hungary (Erdös 1956), Netherlands (**new record**), Slovenia (**new record**), Sweden (Thomson 1878), United Kingdom (Walker 1839); Canada and USA (Hansson 1996b) (Fig. 505).

#### Species group *salicis*

**Diagnosis**. Forewing with admarginal setae arising from marginal vein, and with radial cell bare and long – extending well beyond postmarginal vein (e.g. Fig. 76); male flagellomeres with verticillate setae and in *Omphale chryseis* and *Omphale salicis* with setae apical to basal whorl of setae. Male genitalia (Figs 482–485): phallobase with volsellar setae curved and laterally flattened, not attached on extensions and always crossed in slide mounts but pointed downwards in situ. Species-groups *salicis* and *phruron* share diagnostic externomorphological characters and species are difficult to assign to appropriate group using such characters. The best way to diagnose these groups is to use the appearance and orientation of the volsellar setae in the male genitalia.

##### Morphometric analyses of species in the *salicis* group

Prior to this investigation five species were included in the *salicis* group: *Omphale chryseis*, *Omphale radialis*, *Omphale salicis*, *Omphale theana* (Graham 1963), and *Omphale acuminata* that was described after 1963 also belongs here. Females in this group have been distinguished through the length of gaster, the length of the 7^th^ gastral tergite, and the number of admarginal setae in forewing (Graham 1959, 1963). Based on the number of admarginal setae the species can initially be divided into two groups, those with 2–5 admarginal setae (*Omphale acuminata*, *Omphale radialis*, *Omphale theana*), and those with 5–14 (*Omphale chryseis*, *Omphale salicis*). There is a slight overlap in this character, the mean value for the former group is 3.6, and for the latter 7.8. This subdivision is strengthened by the setation on ventral part of female flagellomeres 2–4, the former group with a single set of setae on each flagellomere, setae attached subbasally, the latter group with two sets of setae, one set attached subbasally, and one medially or subapically.

We have had access to a large number of females of this group and with respect to the length of gaster and of 7^th^ gastral tergite, two linked characters (females with long gasters also have a long 7^th^ tergite) we noticed a substantial and what appeared to be continuous variation and therefore decided to analyze this variation. The gaster is difficult to measure and get comparable measurements because it is usually more or less distorted, but the 7^th^ tergite does not shrivel and can be measured in a comparable way on most specimens. To quantify the variation of this character, measurements of length to the basal width of the 7^th^ gastral tergite in females were taken and plotted against each other in scatter diagrams (Figs 539, 540), one diagram for each subgroup.

The species *Omphale theana* and *Omphale radialis* are very similar and were separated by Graham (1959, 1963) only through the length of the 7^th^ gastral tergite and the length of gaster in female: *Omphale theana* with 7^th^ tergite 2.7–4× (3.3× in type of *Omphale theana*) as long as its basal width and gaster 2–2.5× as long as mesosoma, *Omphale radialis* with 7^th^ tergite 1.7–2.3× (2.0× in type of *Omphale radialis*) as long as its basal width and gaster 1.7–1.9× as long as mesosoma. In the data from specimens of *theana/radialis* (Fig. 539) the variation of the length of 7^th^ gastral tergite is continuous and it is not possible to distinguish any clusters. Since there are no other characters by which these species differ we conclude that *Omphale radialis* and *Omphale theana* belong to one single species with a highly variable gaster length in female. Since *Omphale theana* is the older name, *Omphale radialis* becomes a junior synonym. *Omphale acuminata* is very similar to *Omphale theana* and differs only in the length of female gaster and thus through the length of 7^th^ gastral tergite (the male is not known for *Omphale acuminata*), but in these species a t-test of the data showed a statistical significant difference.

In *Omphale chryseis* and *Omphale salicis* there is also considerable variation in the length of the 7^th^ gastral tergite, and the data also overlap (Fig. 540). Even though *Omphale chryseis* on average has a shorter 7^th^ tergite, the overlap makes it difficult to use this character alone to separate the species, but in combination with the colour of coxae and the WIPs they can be readily distinguished (see below under descriptive part).

To conclude, the length of female gaster and of the length of 7^th^ gastral tergite show a considerable intraspecific variation in this species group, and great care must be taken when using this character for species separation/identification.

The length of the ovipositor is connected to the length of the gaster, and the variable length of the ovipositor certainly has implications for the ability to parasitize the hosts inside their galls. However, very little is known about the biology of the species in this group.

##### Omphale acuminata

Gijswijt

http://species-id.net/wiki/Omphale_acuminata

Figures 8 39–42 73–77 506 539

Omphale acuminata Gijswijt, 1976:79. Holotype female in RMNH, examined.
Omphale acuminata Gijswijt, Askew (2003).

###### Material.

**Type material.** **Holotype** female in RMNH. **Additional material.** 86♀: Germany 1♀ (RMNH), Russia 69♀ (BMNH, CH, LUZM), Sweden 13♀ (BMNH, CH, LUZM), United Kingdom 3♀ (BMNH).

###### Diagnosis.

**Figures 73–77.**
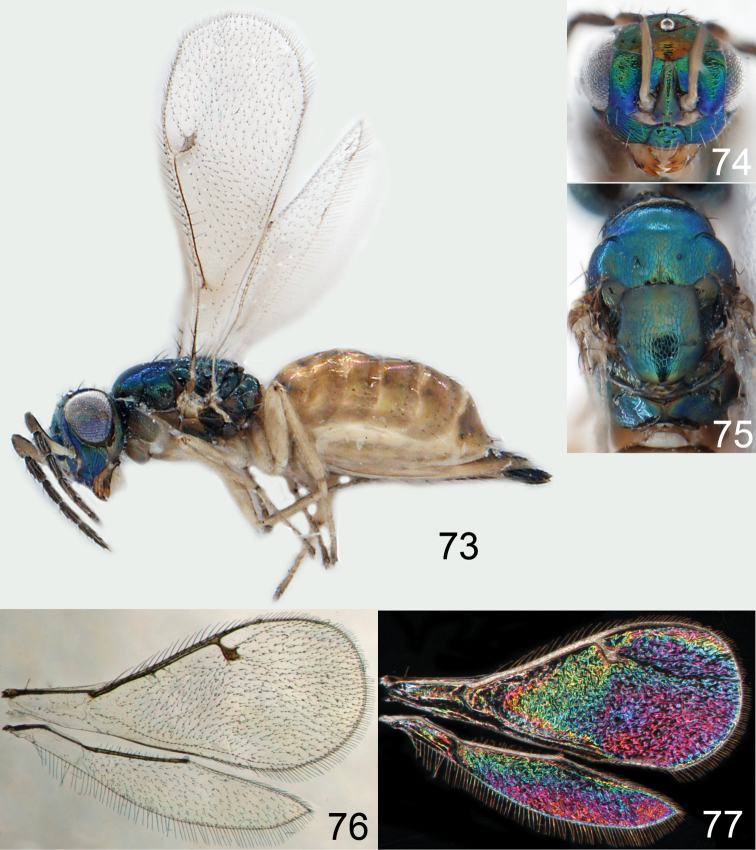
*Omphale acuminata*, female: **73** habitus in lateral view, length of specimen 1.8 mm **74** head in frontal view **75** thoracic dorsum **76** transparent wings **77** wing interference patterns.

Coxae yellowish white to yellowish brown (Fig. 73); forewing short and high (Fig. 76) with 2–5 admarginal setae and a long radial cell; mesepisternum yellowish brown with metallic tinges (Fig. 73); scutellum frequently with pale and weakly metallic parts; female flagellomeres 2-4 ventrally with one set of setae attached subbasally and reaching beyond apex of flagellomere attached to (Fig. 40). Very similar to *Omphale theana*, differs only in the length of 7^th^ gastral tergite in female, which is shorter in *Omphale acuminata* (ratio length/width at base= 0.6–1.1, average= 0.9, n= 59), see above under “Morphometric analyses…” and in having a different WIP – forewing with two large areas with different colours in *Omphale acuminata* (Fig. 77), but with a single colour in *Omphale theana* (Fig. 129).

###### Description.

*Female*. Length of body 1.2–2.0 mm. Antenna with scape pale brown with base yellowish white and dorsal edge dark brown, to yellowish brown with apical ¼ and dorsal edge dark brown; pedicel and flagellum dark brown; pedicel + flagellum 2.1× as long as distance between eyes; first flagellomere 1.0× as long and 1.3× as wide as second flagellomere (Fig. 40); flagellomeres 2–4 ventrally with one set of setae attached subbasally and reaching beyond apex of flagellomere attached to; longitudinal sensilla on flagellomeres about as long as flagellomere attached to; clava 2-segmented. Face bluish green metallic (Fig. 74), strigose (Fig. 41); clypeus bluish green metallic, smooth, trapezoid, 1.3× as wide as high; gena pale brown with metallic tinges, to dark brown with green metallic tinges; lower frons bluish green metallic with parts close to eyes and antennal scrobes purple metallic, with engraved strong reticulation, subtorular area smooth; interscrobal area brown with metallic tinges, smooth; antennal scrobes join on frontal suture; frontal suture V-shaped; upper frons golden with engraved weak reticulation; vertex golden green with blue tinges, with engraved very weak reticulation (Fig. 42). Occipital margin rounded (Fig. 42).

Mesoscutum bluish green metallic with golden tinges (Fig. 75), with engraved reticulation (Fig. 39), midlobe with one pair (posterior pair) of setae; anterior ½ of notauli as wide grooves and posterior ½ as indistinct impressions. Scutellum golden with green metallic tinges (Fig. 75), with engraved reticulation (Fig. 39), or dark brown metallic with sides and anterior margin yellowish brown with metallic tinges; 1.2× as long as wide, with anterior margin smoothly curved forwards. Axillae golden (Fig. 75). Dorsellum golden purple (Fig. 75), smooth and slightly convex (Fig. 39), 0.4× as long as wide, and 0.7× as long as length of median propodeum. Lateral pronotum brown with metallic tinges (Fig. 73); prepectus yellowish brown to brown with metallic tinges; mesepisternum yellowish brown with metallic tinges; transepimeral sulcus weakly curved forwards. Propodeum dark brown with golden green tinges (Fig. 75), median part with purple metallic tinges, smooth (Fig. 39); propodeal callus with two setae. Coxae yellowish white to yellowish brown (Fig. 73), hind coxae with base brown with metallic tinges; fore- and midfemora yellowish white, hind femur yellowish brown; tibiae yellowish white; tarsi yellowish white to yellowish brown with 4^th^ tarsomere darker; midleg with first tarsomere 0.4× as long as length of tarsus. Forewing transparent, veins yellowish brown and setae dark brown (Fig. 76); speculum closed; admarginal setae 2–5, arising from marginal vein; radial cell bare and long, 2.4× as long as length of postmarginal vein; postmarginal vein 1.0–1.2× as long as stigmal vein; stigmal vein very narrow at base, expanding rapidly from base. Hind wing transparent, apex pointed (Fig. 76). Forewing WIP (Fig. 77) with apical ½ magenta and basal ½ yellow.

Petiole yellowish brown. Gaster with tergites 1–6 yellowish brown with posterior margin brown, 7^th^ tergite brown to dark brown; gaster elongate and 1.6–1.8× as long as length of mesosoma; 7^th^ tergite 0.6–1.1× as long as its basal width.

*Male*. Unknown.

###### Host.

Unknown.

###### Distribution.

France (Askew 2003), Germany (**new record**), Greece (Gijswijt 1976), Russia (**new record**), Sweden (Hansson 1991), United Kingdom (Askew 2003) (Fig. 506).

###### Remarks.

Males are not known for *Omphale acuminata* and as species group classification relies heavily on characters in male genitalia the placement of *Omphale acuminata* in the *salicis* group is provisional. However, the female of *Omphale acuminata* is very similar to the female of *Omphale theana* and until males are found *Omphale acuminata* is best placed in the *salicis* group.

##### Omphale chryseis

Graham

http://species-id.net/wiki/Omphale_chryseis

Figures 1 78 –92 482 507 540

Omphale chryseis Graham, 1963:255. Holotype female in OUMNH, examined.

###### Material.

**Type material.** **Holotype** female, type no. 1294 in OUMNH. **Additional material.** 826♀ 248♂: France 2♀ 2♂ (BMNH, RMNH), Germany 12♀ (CH, LUZM, RMNH), Hungary 48♀ 1♂ (BMNH, CH), Netherlands 1♀ 2♂ (RMNH), Poland 1♀ 1♂ (BMNH), Romania 5♀ (BMNH, CH), Russia 2♂ (CH), Spain 1♂ (RMNH), Sweden 753♀ 238♂ (BMNH, CH, LUZM, NHRS), United Kingdom 4♀ 1♂ (BMNH).

###### Diagnosis.

**Figures 78–84.**
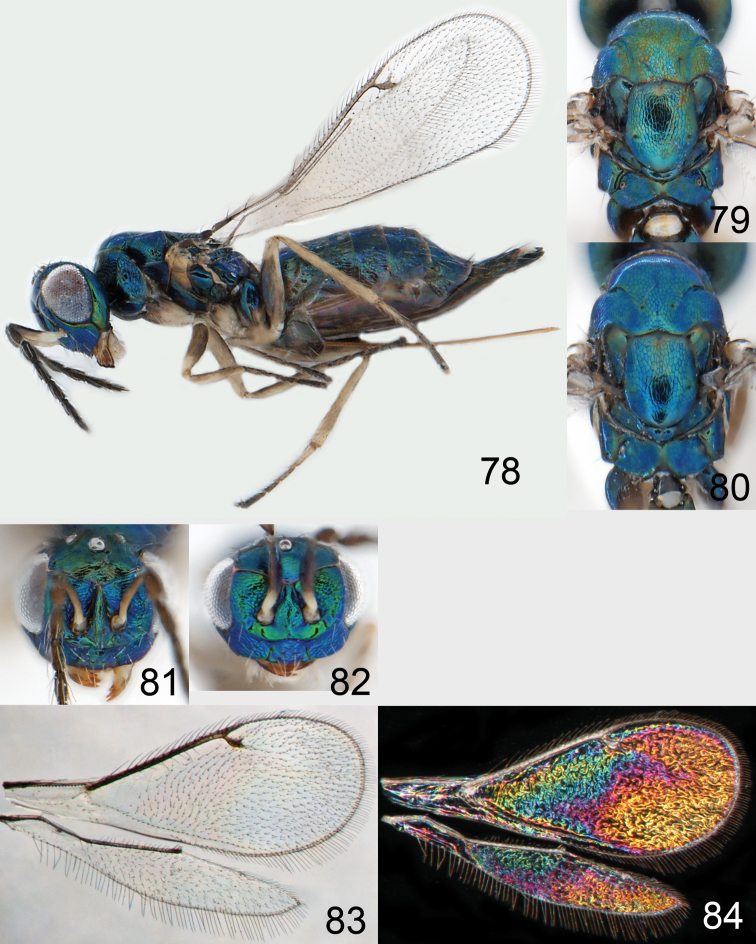
*Omphale chryseis*: **78** habitus in lateral view, female, length of specimen 1.9 mm **79** thoracic dorsum, female **80** thoracic dorsum, male **81** head in frontal view, female **82** head in frontal view, male **83** transparent wings, female **84** wing interference patterns, female.

Legs with fore- and midcoxae predominantly pale and hind coxa predominantly metallic (Fig. 78), femora predominantly brown, tibiae yellowish brown and tarsi predominantly dark brown; female with gaster elongate, 1.7–2.1× as long as length of mesosoma, and with 7^th^ gastral tergite 1.1–4.2× (average= 1.6, n= 53) as long as width at base; female flagellomeres 1–4 ventrally with two sets of long setae, one attached subbasally and one attached medially or subapically (Fig. 86), male flagellomeres 1–4 each with a basal whorls of setae and with scattered setae apical to whorl (Fig. 90); male scape predominantly dark and metallic; female forewing with 5–12 (average= 7.5, n= 53) admarginal setae, male with 7–9 admarginal setae; head and thoracic dorsum usually bluish green metallic (Figs 79–82). Similar to *Omphale salicis* from which it can be distinguished through the colour of coxae – fore- and midcoxae pale and hind coxa dark in *Omphale chryseis*, all coxae dark in *Omphale salicis*; *Omphale chryseis* on average has a shorter 7^th^ gastral tergite in female. These species can also be separated through their WIP in forewings: *Omphale chryseis* apical ½ yellow and basal ½ with wide bands in magenta, blue and yellow (Fig. 84), *Omphale salicis* has a narrow straight yellow line from stigmal vein to hind margin of wing separating an apical blue and a basal magenta area (Fig. 114).

###### Description.

*Female*. Length of body 1.4–2.1 mm. Antenna with scape yellowish white with apical ⅓ and entire dorsal edge dark brown; pedicel and flagellum dark brown; pedicel + flagellum 1.9× as long as distance between eyes; first flagellomere 1.1× as long and 1.4× as wide as second flagellomere (Fig. 86); flagellomeres 1–4 ventrally with two sets of long setae, one attached subbasally and one attached medially or subapically; longitudinal sensilla on flagellomeres as long as flagellomere attached to; clava 2-segmented. Face bluish green metallic (Fig. 81), strigose (Fig. 87); clypeus bluish green metallic, smooth or with weak reticulation, semicircular to trapezoid, 1.4× as wide as high; gena greenish blue metallic; lower frons bluish purple to bluish green metallic, with engraved rather strong reticulation; interscrobal area with engraved weak reticulation; antennal scrobes join on frontal suture; frontal suture V-shaped; upper frons bluish green metallic with engraved weak reticulation; vertex bluish green metallic, to golden green, with engraved very weak reticulation, partly smooth outside ocellar triangle (Fig. 88). Occipital margin rounded (Fig. 88).

**Figures 85–92.**
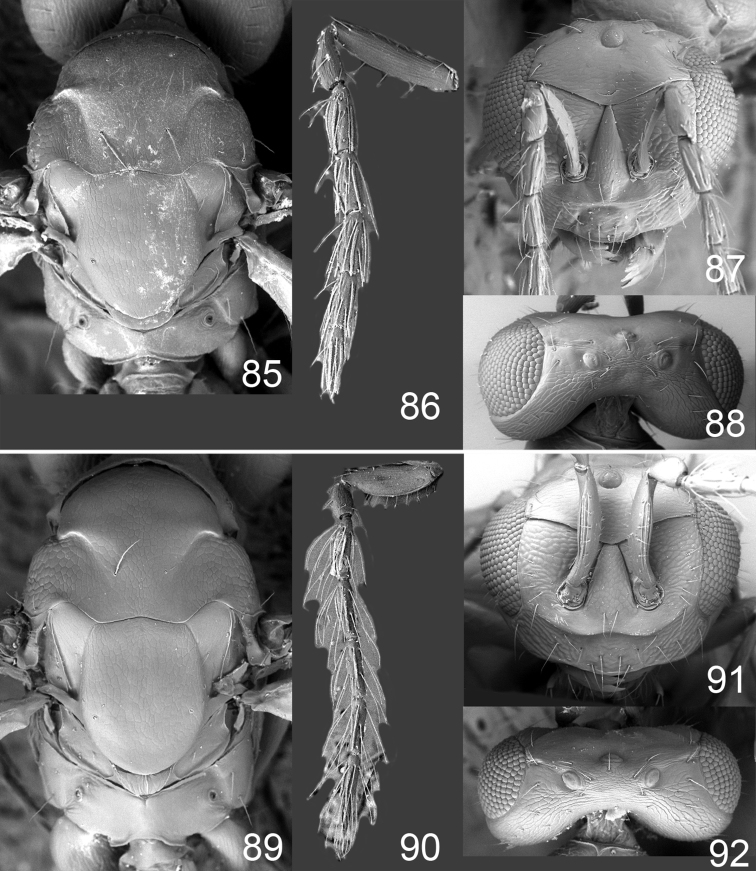
*Omphale chryseis*: **85** thoracic dorsum, female **86** antenna, female **87** head in frontal view, female **88** vertex, female **89** thoracic dorsum, male **90** antenna, male **91** head in frontal view, male **92** vertex, male.

Mesoscutum bluish green metallic (Fig. 79) with or without purple metallic tinges, with engraved reticulation (Fig. 85), midlobe with two pairs of setae; notauli as indistinct impressions in posterior ½. Scutellum bluish green metallic (Fig. 79), with engraved reticulation (Fig. 85); 1.2× as long as wide, with anterior margin smoothly curved forwards. Axillae bluish green metallic (Fig. 79). Dorsellum golden green (Fig. 79), smooth and convex (Fig. 85), 0.2× as long as wide, and 0.6× as long as length of median propodeum. Lateral pronotum and propleuron bluish purple to bluish green metallic (Fig. 78); prepectus dark brown with bluish green metallic tinges; acropleuron and mesepisternum pale brown and shiny; mesepimeron golden green with blue metallic tinges; transepimeral sulcus curved forwards. Propodeum bluish green metallic (Fig. 79), smooth (Fig. 85); propodeal callus with two setae. Fore- and midcoxae yellowish white with base pale brown (Fig. 78), hind coxa predominantly bluish green metallic with purple tinges with apical part yellowish brown; femora predominantly pale brown to dark brown with apical part yellowish brown; tibiae yellowish brown; tarsi predominantly dark brown, mid- and hind tarsi with first tarsomere paler; midleg with first tarsomere 0.4× as long as length of tarsus. Forewing transparent, veins pale brown and setae dark brown (Fig. 83); speculum closed; admarginal setae 6–11, arising from marginal vein; radial cell bare and long, 2.2× as long as length of postmarginal vein; postmarginal vein 0.9× as long as stigmal vein; stigmal vein long and slightly enlarged. Hind wing transparent, apex rounded (Fig. 83). Forewing WIP (Fig. 84) with apical ½ yellow and basal ½ with wide bands in magenta, blue and yellow.

Petiole dark brown. Gaster dark brown, tergites 1 and 6 with bluish green metallic tinges, 2–5 with golden purple tinges, 7 with golden green tinges, smooth, elongate and 1.7–2.1× as long as length of mesosoma; 7^th^ tergite 0.2–0.3× as long as length of gaster.

*Male*. Length of body 1.2–1.5 mm. Features as in female except as follows. Antenna with scape dark brown with blue or green metallic tinges, with basal ⅓–¼ yellowish white; pedicel + flagellum 2.8× as long as distance between eyes; flagellomeres with scattered setae (Fig. 90); clava 1-segmented. Face bluish green to green metallic (Fig. 82); clypeus bluish green to green metallic, smooth, trapezoid (Fig. 91), 1.6× as wide as high; gena golden red to golden green; lower frons golden green to bluish green metallic, with engraved and strong reticulation; interscrobal area with weak reticulation; upper frons golden green to bluish green metallic; vertex golden green with red metallic tinges, with engraved weak reticulation (Fig. 92).

Axillae golden green (Fig. 80). Dorsellum bluish green metallic (Fig. 80), smooth or with weak reticulation and convex (Fig. 89), 0.3× as long as wide, and 0.4× as long as length of median propodeum. Lateral pronotum and propleuron bluish green metallic; prepectus bluish green metallic; acropleuron and mesepisternum pale brown with metallic tinges; mesepimeron golden green. Midleg with first tarsomere 0.3× as long as length of tarsus. Forewing admarginal setae 7–9.

Petiole dark brown. Gaster with first tergite bluish green metallic, remaining tergites dark brown to black with golden and green metallic tinges, smooth, 1.1–1.4× as long as length of mesosoma. Phallobase and aedeagus as in Fig. 482.

###### Host.

*Contarinia medicaginis* (Diptera: Cecidomyiidae) (Bouček and Askew 1968) - endoparasite of larvae (Královič 1964).

###### Distribution.

Czech Republic (Bouček and Askew 1968), France (Bouček and Askew 1968), Germany (Gijswijt 1976), Hungary (**new record**), Netherlands (**new record**), Poland (**new record**), Romania (**new record**), Russia (St. Petersburg area) (**new record**), Spain (**new record**), Sweden (Bouček and Askew 1968), United Kingdom (Graham 1963) (Fig. 507).

###### Remarks.

The description of *Omphale chryseis* was based on three female specimens and it is possible that these represent two different species. Graham (1963) described the colour of coxae as “green, pale at apex, or fore- and midcoxae pale testaceous and dark at base only”. We have only examined the holotype, not the paratypes, and the holotype has pale fore- and midcoxae, and a dark hind coxa, and this is how we interpret *Omphale chryseis*, of which we have examined over a thousand specimens – this species is easily collected when sweeping *Medicago*. The specimen(s) in the type series with all coxae green is probably *Omphale salicis* with a short gaster. Graham distinguished *Omphale chryseis* from *Omphale salicis* mainly in the length of the female gaster (the male of *Omphale chryseis* was unknown to Graham). As shown above, the length of female gaster in these two species is highly variable and cannot be trusted to separate them.

##### Omphale cornula sp. n.

urn:lsid:zoobank.org:act:A32F508A-0FA0-466A-9835-57DAE227E203

http://species-id.net/wiki/Omphale_cornula

Figures 93 –107 483 508

###### Material.

**Holotype** female (ZMUC), glued to a card, labelled: “DENMARK: E-Jutland, Glatved, 56°17'N, 10°50'E, 16.vii.2003, T. Munk”. **Paratypes**. 10♀ 4♂: DENMARK: 1♀ from same locality as holotype but collected 11.vii.2005 (ZMUC). FRANCE: 1♀ “Dordogne, Les Eyzies, 4.viii.1974, M.W.R. de V. Graham” (BMNH); 1♀ “Dordogne, nr. Allas “Thomas” (3) 5.viii.1974, M.W.R. de V. Graham” (BMNH); 2♂ “Dordogne, nr. St Cyprien (Castels marsh) (2), 5.viii.1974, M.W.R. de V. Graham” (BMNH); 1♀ from same locality as previous but collected 7.viii.1974 (BMNH); 1♀ “Dept Jura, Viry, 21.vii.1971, M.J. Gijswijt” (RMNH); 1♀ “Gavarnie, Soula de Sarre, 1400–1450 m, 16.vii.1975, A.C. & W.N. Ellis” (RMNH). NETHERLANDS: 1♀ “Kenn-duin, 18.viii.1965, M.J. Gijswijt” (RMNH). SWEDEN: 1♀ 1♂ “Skåne, Vomb, 55°40'N, 13°33'E, 1.viii.1984, C. Hansson” (BMNH); 1♀ from same locality as previous but collected 18.vii.2006 (BMNH); 1♀ “Skåne, Silvåkra, 13.vii.1979, C. Hansson” (CH). UNITED KINGDOM: 1♂ “England, Surrey, Coulsdon Common, Happy Valley, 14.vii.1979, J.S. Noyes” (BMNH).

###### Diagnosis.

**Figures 93–99.**
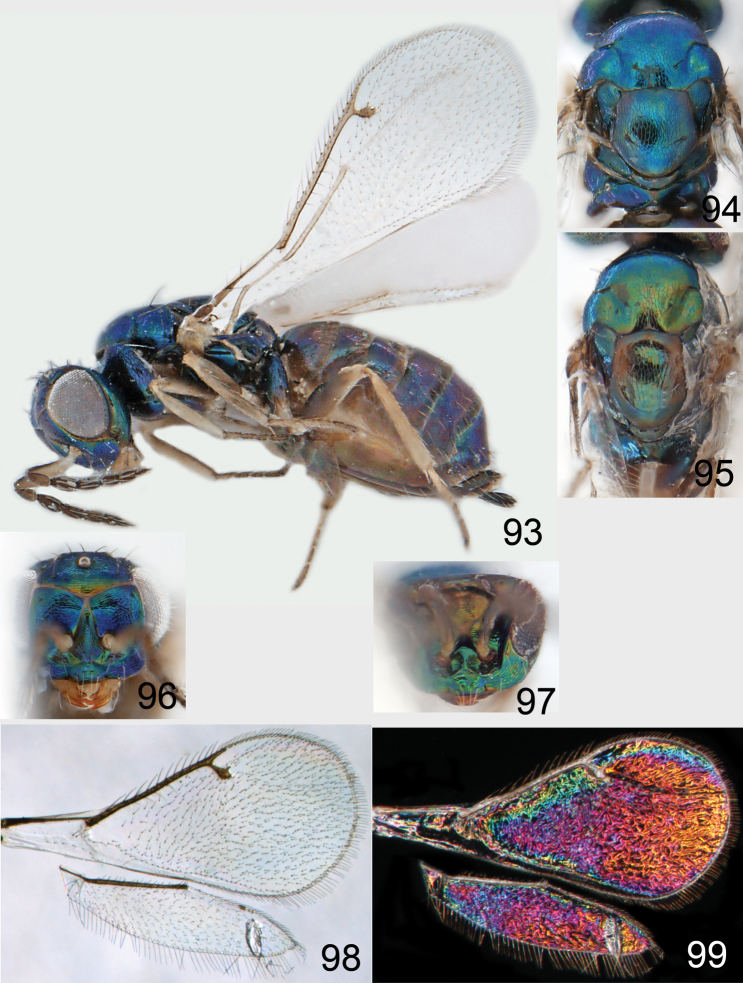
*Omphale cornula*: **93** habitus in lateral view, female, length of specimen 1.5 mm **94** thoracic dorsum, female **95** thoracic dorsum, male **96** head in frontal view, female **97** head in frontal view, male **98** transparent wings, female **99** wing interference patterns, female.

Female flagellum short (Figs 93, 101), pedicel and flagellum 1.5× as long as distance between eyes; thoracic dorsum bluish green metallic, scutellum usually with lateral parts pale or palish (Figs 94, 95); all coxae metallic (Fig. 93); female gaster 1.3–1.5× as long as mesosoma.

###### Description.

*Female*. Length of body 1.2–1.5 mm. Antenna with scape pale brown with dorsal edge dark brown; pedicel and flagellum dark brown; pedicel + flagellum 1.5× as long as distance between eyes; first flagellomere 1.2× as long and 1.2× as wide as second flagellomere (Fig. 101); flagellomeres 2–4 ventrally with one set of setae attached subbasally and reaching beyond apex of flagellomere attached to; clava 2-segmented. Face bluish purple metallic (Fig. 96), strigose Fig. 102; clypeus golden green, smooth, semicircular, 2.0× as wide as high; gena golden green; lower frons with parts between antennal scrobes and eyes bluish purple metallic, with strong reticulation, interscrobal area and parts below level of toruli golden green, with weak reticulation; antennal scrobes join on frontal suture; frontal suture V-shaped; upper frons golden green, with weak reticulation; vertex bluish green metallic, with very weak reticulation (Fig. 103). Occipital margin rounded (Fig. 103).

**Figures 100–107.**
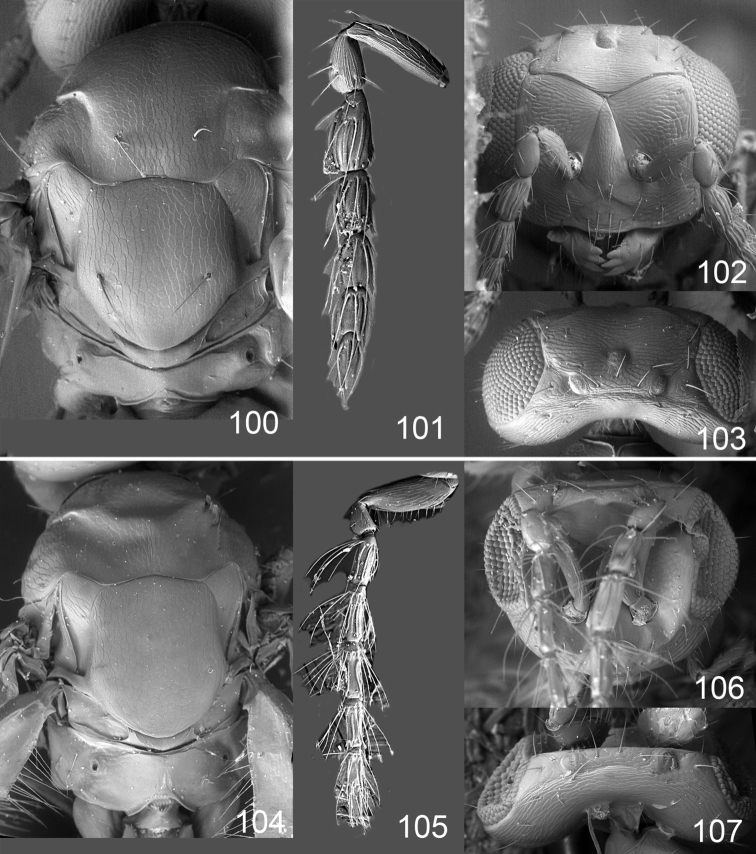
*Omphale cornula*: **100** thoracic dorsum, female **101** antenna, female **102** head in frontal view, female **103** vertex, female **104** thoracic dorsum, male **105** antenna, male **106** head in frontal view, male **107** vertex, male.

Mesoscutum bluish green metallic (Fig. 94), with engraved reticulation (Fig. 100), midlobe with one pair of setae (posterior pair); notauli as narrow grooves in anterior ½ and as indistinct impressions in posterior ½. Scutellum bluish green metallic, holotype with lateral parts dark brown with bluish green metallic tinges, one paratype with three wide longitudinal stripes, with lateral stripes pale brown with metallic tinges and median stripe bluish-green metallic (as in Fig. 200), one paratype with scutellum bluish green metallic with anterior corners yellowish brown (Fig. 94); with engraved reticulation (Fig. 100); 1.0× as long as wide, with anterior margin smoothly curved forward. Axillae bluish green metallic (Fig. 94). Dorsellum brown with metallic tinges (Fig. 94), smooth and convex (Fig. 100), 0.3× as long as wide, and 0.8× as long as length of median propodeum. Entire lateral mesosoma bluish green metallic (Fig. 93); transepimeral sulcus curved forwards. Propodeum with median part golden green and lateral parts bluish green metallic (Fig. 94), smooth (Fig. 100); propodeal callus with two setae. Legs with coxae bluish green metallic (Fig. 93); femora dark brown; tibiae yellowish brown to pale brown; tarsi dark brown; midleg with first tarsomere 0.3× as long as length of tarsus. Forewing transparent, veins yellowish brown and setae dark brown (Fig. 98); speculum closed; admarginal setae 4–7 arising from marginal vein; radial cell bare; postmarginal vein 1.2× as long as stigmal vein; stigmal vein slender. Hind wing transparent, apex pointed (Fig. 98). Forewing WIP (Fig. 99) with apical ½ a mix of yellow and magenta, basal ½ magenta, surface close to foremargin blue.

Petiole dark brown. Gaster dark brown with bluish green to bluish purple metallic tinges, elongate ovate and 1.2–1.5× as long as length of mesosoma; 7^th^ tergite short, 0.3× as long as wide and 0.05× as long as length of gaster.

*Male*. Length of body 1.0–1.2 mm. Features as in female except as follows. Scape golden green with base yellowish brown, pedicel and flagellum dark brown metallic; pedicel + flagellum 2.3× as long as distance between eyes; flagellomeres 1-4 with verticillate setae and with setae reaching beyond apex of flagellomere attached to, without scattered setae apical to basal whorl (Fig. 105); clava 1-segmented. Face golden green (Fig. 97), strigose (Fig. 106); clypeus golden green, with irregular sculpture, trapezoid, 1.6× as wide as high; gena golden green or golden red; lower frons golden green, with weak reticulation; antennal scrobes join on frontal suture; frontal suture V-shaped; upper frons golden red, with weak reticulation; vertex golden red, with engraved weak reticulation (Fig. 107).

Mesoscutum golden green (Fig. 95) or golden, with engraved reticulation (Fig. 104), midlobe with one pair of setae (posterior pair). Scutellum golden green with sides yellowish brown (Fig. 95); 1.1× as long as wide. Axillae golden green (Fig. 95). Dorsellum dark brown with metallic green tinges (Fig. 95), concave and smooth (Fig. 104), 0.3× as long as wide, and 0.4× as long as length of median propodeum. Propodeum golden with red tinges. Lateral pronotum, prepectus, mesepisternum and mesepi-meron metallic bluish green. Legs with coxae and femora dark brown metallic; tibiae yellowish brown to pale brown; foretarsus dark brown, mid- and hind tarsi yellowish brown. Forewing admarginal setae 4.

Gaster dark brown with metallic tinges, 1.0–1.3× as long as length of mesosoma. Phallobase and aedeagus as in Fig. 483.

###### Host.

Unknown.

###### Distribution.

Denmark, France, Netherlands, Sweden, United Kingdom (Fig. 508).

###### Etymology.

From the Latin *cornu* = horn, *cornula* is a diminutive form, referring to the short antennae.

##### Omphale salicis

Haliday

http://species-id.net/wiki/Omphale_salicis

Figures 11 108 –122 484 509

Omphale salicis Haliday, 1833:339. Lectotype female in NMID, not examined.
Eulophus subulatus Nees, 1834:167. Type not located. Synonymized by Thomson (1878:268).
Eulophus terebrator Förster, 1841:42. Type not located. Synonymized by Bouček and Askew (1968:135).
Omphale salicis Haliday (Graham 1963).
Omphale salicis Haliday (Hansson 1996b).

###### Material.

**Type material.** We have been unable to examine any material from the museum in NMID and we base our interpretation of *Omphale salicis* on the information in Graham (1963) where the lectotype for *Omphale salicis* was designated. **Additional material.** 269♀ 70♂: Austria 1♀ (RMNH), Czech Republic 1♀ 2♂ (BMNH, RMNH), France 3♀ 5♂ (RMNH), Germany 1♀ 2♂ (RMNH), Greece 1♀ (LUZM), Hungary 2♀ (BMNH, RMNH), Ireland 5♀ 3♂ (BMNH), Italy 3♀ (RMNH), Norway 2♀ (LUZM), Portugal 1♀ (RMNH), Russia 22♀ 1♂ (CH, LUZM), Sweden 100♀ 23♂ (BMNH, CH, LUZM, RMNH), Switzerland 1♀ (RMNH), United Kingdom 126♀ 34♂ (BMNH, CH).

###### Diagnosis.

**Figures 108–114.**
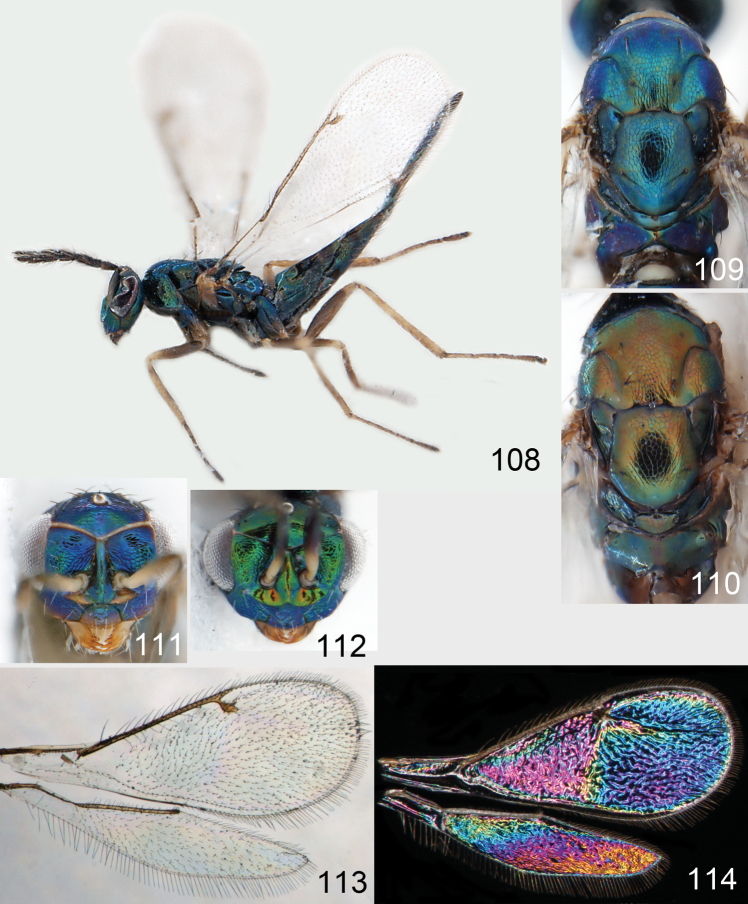
*Omphale salicis*: **108** habitus in lateral view, female, length of specimen 2.3 mm **109** thoracic dorsum, female **110** thoracic dorsum, male **111** head in frontal view, female **112** head in frontal view, male **113** transparent wings, female **114** wing interference patterns, female.

Female 7th gastral tergite 1.4–4.5× (average= 2.6, n= 109) as long as its basal width; coxae in both sexes predominantly dark and metallic (Fig. 108); female forewing with 6–14 (average= 8, n= 109) admarginal setae, male with 6–12 admarginal setae; female flagellomeres 1–4 ventrally with two sets of long setae, one attached subbasally and one attached medially or subapically (Fig. 116), male flagellomeres 1–4 each with a basal whorl of setae and with scattered setae apical to whorl (Fig. 120). Similar to *Omphale chryseis* but with all coxae dark and metallic, and female on average with a longer 7^th^ gastral tergite. These species can also be separated through their WIP in forewings: *Omphale salicis* has a narrow straight yellow line from stigmal vein to hind margin of wing separating an apical blue and a basal magenta area (Fig. 114), *Omphale chryseis* has apical ½ yellow and basal ½ with wide bands in magenta, blue and yellow (Fig. 84). Also similar to *Omphale theana* but with more admarginal setae in forewing, female flagellomeres 1–4 ventrally with two sets of long setae, coxae predominantly dark and metallic, mesosoma predominantly dark and metallic, scutellum very occasionally with sides brownish; forewing WIPs different: *Omphale salicis* with apical and basal halves with different colours (Fig. 114), *Omphale theana* with one colour (Fig. 129).

###### Description.

*Female*. Length of body 1.4–3.1 mm. Antenna with scape brown with dorsal margin dark brown; pedicel and flagellum dark brown; pedicel + flagellum 2.0× as long as distance between eyes; first flagellomere 1.1× as long and 1.0× as wide as second flagellomere (Fig. 116); flagellomeres 1–4 ventrally with two sets of long setae, one attached subbasally and one attached medially or subapically; clava 2-segmented. Face golden green to bluish green metallic (Fig. 111), strigose-reticulate (Fig. 117); clypeus golden green, smooth, semicircular, 1.6× as wide as high; gena golden green with or without red metallic tinges; lower frons including interscrobal area golden green, green or bluish green metallic, with strong reticulation; antennal scrobes join frontal suture separately; frontal suture V-shaped; upper frons golden, with very weak reticulation; vertex golden red with green tinges, with very weak reticulation (Fig. 118). Occipital margin rounded (Fig. 118).

**Figures 115–122.**
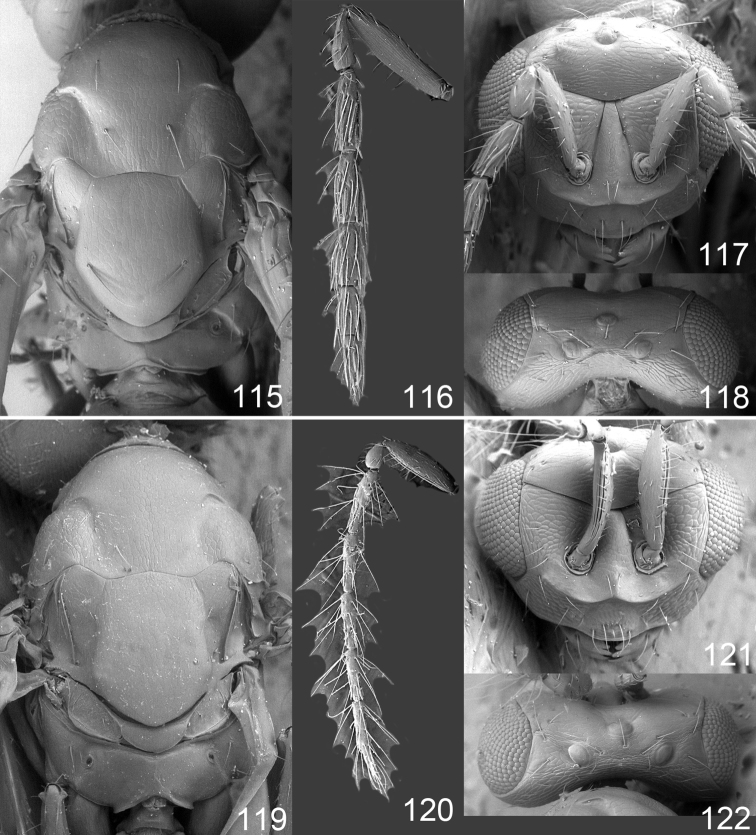
*Omphale salicis*: **115** thoracic dorsum, female **116** antenna, female **117** head in frontal view, female **118** vertex, female **119** thoracic dorsum, male **120** antenna, male **121** head in frontal view, male **122** vertex, male.

Mesoscutum golden green, green or bluish green metallic (Fig. 109), occasionally golden red, with engraved reticulation (Fig. 115), midlobe with 1–2 pairs of setae; notauli as indistinct impressions in posterior ½. Scutellum golden green, green or bluish green metallic (Fig. 109), occasionally golden red, very rarely with lateral parts brown with metallic tinges, with engraved and weak reticulation (Fig. 115), 1.2× as long as wide, with anterior margin smoothly curved forward. Axillae golden green or green metallic (Fig. 109). Dorsellum golden green (Fig. 109), convex and smooth (Fig. 115), 0.3× as long as wide, and 0.9× as long as length of median propodeum. Entire lateral mesosoma golden green, green or bluish green metallic (Fig. 108); transepimeral sulcus curved forwards. Propodeum golden green, green or bluish green metallic (Fig. 109), smooth (Fig. 115); propodeal callus with two setae. Coxae green or bluish green metallic (Fig. 108), midcoxa sometimes paler; femora dark brown; tibiae yellowish brown; foretarsus dark brown, mid- and hind tarsi with segments 1–3 yellowish brown and 4 dark brown; midleg with first tarsal segment 0.4× as long as tarsus. Forewing transparent, veins yellowish brown, setae dark brown (Fig. 113); speculum closed; admarginal setae 6–14, arising from marginal vein; radial cell bare and long, 3.2× as long as length of postmarginal vein; postmarginal vein 0.8× as long as stigmal vein. Hind wing tran-sparent, apex pointed (Fig. 113). Forewing WIP (Fig. 114) with apical ½ blue and basal ½ magenta, separated by a straight and narrow yellow line.

Petiole dark brown. Gaster with tergites 1–6 golden green to bluish green metallic with posterior ½ dark brown metallic, shiny, smooth, elongate and 2.5–2.7× as long as length of mesosoma; 7^th^ tergite 0.2× as long as length of gaster.

*Male*. Length of body 1.0–1.7 mm. Features as in female except as follows. Antenna with scape dark brown metallic with basal part yellowish white; pedicel + flagellum 3.4× as long as distance between eyes; flagellomeres 1–4 with verticillate setae and with setae apical to basal whorl (Fig. 120); clava 1-segmented. Face golden red or bluish green metallic (Fig. 112); clypeus golden green, bluish green metallic, 1.5× as wide as high; gena golden red, green metallic; lower frons golden red with green metallic tinges, bluish green metallic, with weak reticulation (Fig. 121), interscrobal area smooth; upper frons golden red; vertex golden red, smooth (Fig. 122).

Mesoscutum golden red (Fig. 110), bluish green metallic, with engraved reticulation (Fig. 119), midlobe with one pair of setae (posterior pair). Scutellum golden red (Fig. 110), golden green, or bluish green metallic; 1.3× as long as wide. Axillae golden red (Fig. 110). Dorsellum golden, 0.5× as long as wide, and 0.7× as long as length of median propodeum. Entire lateral mesosoma golden green with red metallic tinges. Propodeum golden red with lateral parts golden green (Fig. 110). Coxae golden green or bluish green metallic, midcoxa sometimes paler; femora dark brown to pale brown; midleg with first tarsal segment 0.3× as long as tarsus. Forewing admarginal setae 6–12; postmarginal vein 0.9× as long as stigmal vein.

Petiole dark brown. Gaster with first tergite golden green in anterior ½, posterior ½ and remaining tergites dark brown with purple metallic tinges, 0.9-1.1× as long as length of mesosoma. Phallobase and aedeagus as in Fig. 484.

###### Hosts.

*Contarinia lentis* (Diptera: Cecidomyiidae) (probable record) (Szelényi 1944), *Contarinia loti* (Gijswijt 1976); *Contarinia vincetoxici* (**new record**).

###### Distribution.

Austria (Kirchner 1867), Czech Republic (Bouček and Askew 1968), France (De Gaulle 1908), Germany (Nees 1834), Greece (**new record**), Hungary (Szelényi 1944), Ireland (**new record**), Italy (**new record**), Netherlands (Bouček and Askew 1968), Norway (**new record**), Portugal (**new record**), Russia (St. Petersburg area) (**new record**), Sweden (Thomson 1878), Switzerland (**new record**), United Kingdom (Haliday 1833), Yugoslavia (Bouček and Askew 1968); Canada (Hansson 1996b) (Fig. 509).

##### Omphale theana

(Walker)

http://species-id.net/wiki/Omphale_theana

Figures 123 –137 485 510 539

Entedon theana Walker, 1839:81. Lectotype female in BMNH, examined.
Entedon ithonus Walker, 1839:82. Lectotype female in BMNH, examined. Synonymized by Graham (1963:254).
Derostenus (Derostenus) radialis Thomson, 1878:269. Lectotype female in LUZM, examined. Syn. n.
Omphale radialis (Thomson), Dalla Torre (1898).
Achrysocharella americana Girault, 1916:295. Holotype female in USNM, examined. Syn. n.
Omphale theana (Walker), Graham (1959).
Omphale theana (Walker), Graham (1963).
Omphale radialis (Thomson), Graham (1963).
Omphale theana (Walker), Hansson (1996b).
Omphale radialis (Thomson), Hansson (1996b).

###### Material. 

**Type material.** Lectotype females of *Entedon theana*, type no. 5.2033, and *Entedon ithonus*, type no. 5.2034, both types in BMNH; lectotype female of *Derostenus radialis*, type no. 117:1 in LUZM; holotype female of *Achrysocharella americana*, type no 19586 in USNM. **Additio-nal material.** 334♀ 3♂: France 11♀ 1♂ (BMNH, RMNH), Germany 6♀ (RMNH), Hungary 10♀ (BMNH, CH), Netherlands 3♀ (RMNH), Russia 64♀ (BMNH, CH, LUZM), Sweden 175♀ (BMNH, CH, LUZM), United Kingdom 65♀ 2♂ (BMNH).

###### Diagnosis.

**Figures 123–129.**
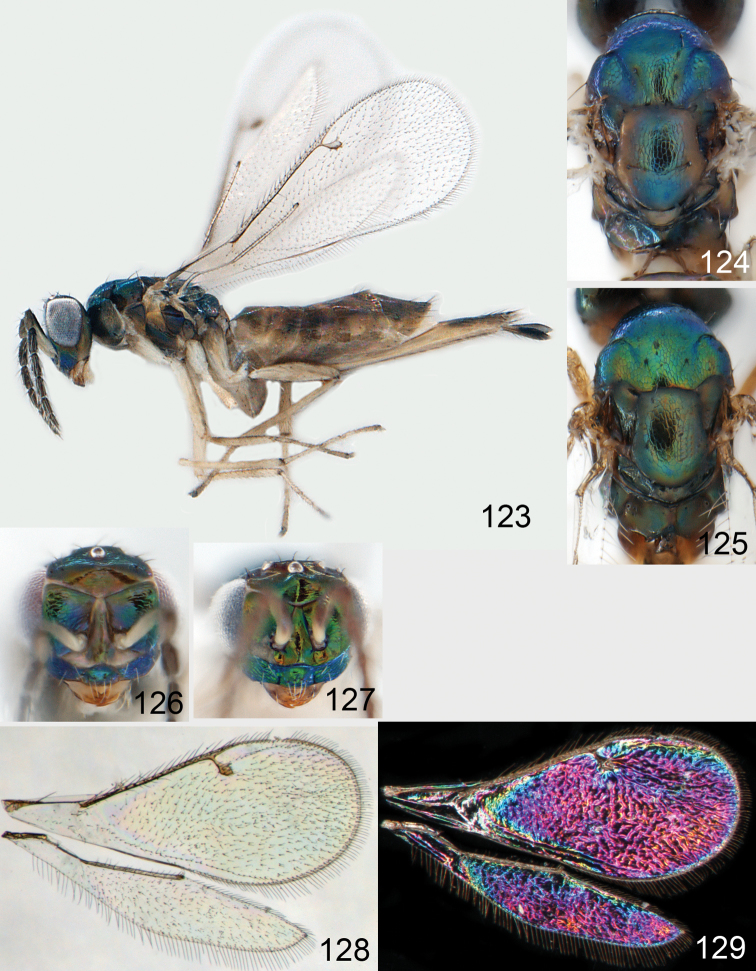
*Omphale theana*: **123** habitus in lateral view, female, length of specimen 2.1 mm **124** thoracic dorsum, female **125** thoracic dorsum, male **126** head in frontal view, female **127** head in frontal view, male **128** transparent wings, female **129** wing interference patterns, female.

Female with 7th gastral tergite 1.3–4.3× (average= 2.5, n= 156) as long as its basal width; interscrobal area, prosternum, prepectus and sides of scutellum frequently brownish-testaceous non metallic; coxae yellowish brown to yellowish white (Fig. 123), base with or without infuscation; forewing with 2–5 admarginal setae; female flagellomeres 2–4 ventrally with one set of setae attached subbasally and reaching beyond apex of flagellomere attached to (Fig. 131); male flagellomeres 1–4 with a single basal whorl of setae (Fig. 135), flagellomere 5 with scattered setae; forewing interference pattern, both sexes, with one colour (Fig. 129) – hue varies depending on size of specimen, which is different from the other species in this group that have different colours on basal and apical halves of the forewing. Very similar to *Omphale acuminata*, differs in the length of the 7^th^ gastral tergite in female, which is longer in *Omphale theana*, see above “Morphometric analyses…”, and in having a different WIP – forewing with one colour in *Omphale theana* (Fig. 129), but with two large areas with different colours in *Omphale acuminata* (Fig. 77). Through the elongate female gaster also similar to *Omphale salicis*, from which it differs in having fewer admarginal setae in forewing (6–14 in *Omphale salicis*), in having coxae predominantly non-metallic, and in WIPs – the unicoloured forewing WIP in *Omphale theana* separates it from the other species in this group.

###### Description.

*Female*. Length of body 1.2–2.3 mm. Antenna with scape yellowish brown with apical ⅓ and dorsal margin dark brown; pedicel and flagellum dark brown; pedicel + flagellum 2.1× as long as distance between eyes; first flagellomere 1.0× as long and 1.0× as wide as second flagellomere (Fig. 131); flagellomeres 1–4 ventrally with one set of setae attached subbasally and reaching beyond apex of flagellomere attached to; clava 2-segmented. Face golden green to bluish green metallic (Fig. 126), strigose (Fig. 132); clypeus golden green with margin towards frons frequently brown, wrinkled to smooth, trapezoid to semicircular, 1.7× as wide as high; gena not visible on type of *Omphale theana* but brown with golden tinges in non-type material; lower frons golden green to bluish green metallic, with weak reticulation; interscrobal area pale brown, yellowish brown, or dark brown metallic, smooth; antennal scrobes join frontal suture separately; frontal suture V-shaped; upper frons golden to bluish green metallic, with very weak reticulation; vertex brown with golden to golden green tinges, with very weak reticulation (Fig. 133). Occipital margin rounded (Fig. 133).

**Figures 130–137.**
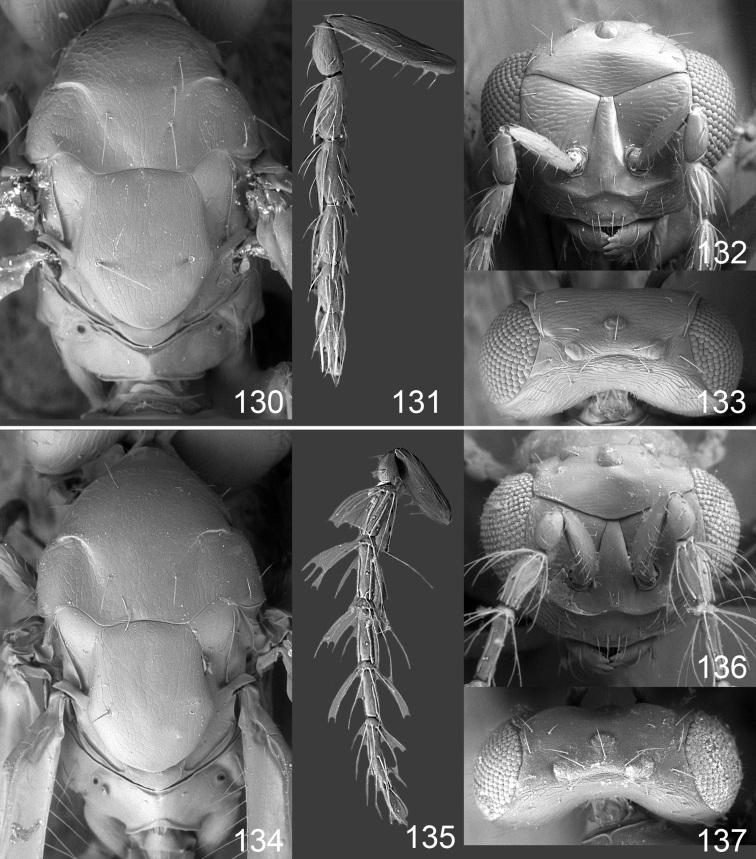
*Omphale theana*: **130** thoracic dorsum, female **131** antenna, female **132** head in frontal view, female **133** vertex, female **134** thoracic dorsum, male **135** antenna, male **136** head in frontal view, male **137** vertex, male.

Mesoscutum golden red with green metallic tinges, golden green, or bluish green metallic (Fig. 124), with engraved reticulation (Fig. 130)), midlobe with two pairs (1–2 pairs in non-type material) of setae; notauli as indistinct impressions in posterior ½. Scutellum golden red, golden green, or bluish green metallic (Fig. 124), and frequently partly brown to yellowish brown – then usually with lateral parts non metallic, with engraved and weak reticulation (Fig. 130), 1.1× as long as wide, with anterior margin smoothly curved forward. Axillae brown with golden tinges (Fig. 124). Dorsellum brown to yellowish brown (Fig. 124), convex and smooth (Fig. 130), 0.4× as long as wide, and 1.0× as long as length of median propodeum. Entire lateral mesosoma brown with metallic tinges (Fig. 123), to with propleuron, lateral pronotum, prepectus and mesepisternum yellowish brown; transepimeral sulcus curved forwards. Propodeum dark brown with metallic tinges (Fig. 124), smooth (Fig. 130); propodeal callus with two setae. Legs yellowish brown, with base of hind coxa brown (Fig. 123); midleg with first tarsal segment 0.4× as long as tarsus. Forewing transparent, veins yellowish brown, setae dark brown (Fig. 128); speculum closed; admarginal setae 4 (2–5 in non-type material), arising from marginal vein; radial cell bare and long, 2.0× as long as length of postmarginal vein; postmarginal vein 0.9–1.0× as long as stigmal vein. Hind wing transparent, apex pointed (Fig. 128). Forewing WIP (Fig. 129) entirely in magenta.

Petiole brown. Gaster dark brown, to yellowish brown with posterior margin of tergites dark brown, and shiny, smooth, elongate and 2.8× (2.0–2.7× in non-type material) as long as length of mesosoma; 7^th^ tergite 0.3–0.5× as long as length of gaster.

*Male*. Length of body 1.2–1.3 mm. Features as in female except as follows. Antennal pedicel + flagellum 3.1× as long as distance between eyes; flagellomeres 1–4 with verticillate setae and with setae reaching beyond apex of flagellomere attached to (Fig. 135); clava 1-segmented. Face metallic bluish green (Fig. 127), strigose (Fig. 136); clypeus metallic bluish green, smooth, 1.2× as wide as high; gena golden green; lower frons golden green or golden red; upper frons golden or golden red; vertex golden red, with weak reticulation (Fig. 137).

Mesoscutum bluish green metallic (Fig. 125) or golden red, midlobe with one pair of setae (posterior pair) (Fig. 134). Scutellum golden green or golden red, 1.2× as long as wide. Axillae golden. Dorsellum dark brown with golden tinges (Fig. 125); 0.7× as long as length of median propodeum. Entire lateral mesosoma golden green or bluish green metallic. Propodeum golden red. Legs yellowish brown, hind coxa with base dark brown metallic; midleg with first tarsomere 0.3× as long as length of tarsus. Forewing admarginal setae 3–5; postmarginal vein 0.9× as long as stigmal vein.

Petiole dark brown. Gaster dark brown metallic, smooth, 1.1× as long as length of mesosoma. Phallobase and aedeagus as in Fig. 485.

###### Host.

Unknown.

###### Distribution.

Czech Republic (Bouček and Askew 1968), Faroes (Kryger and Schmiedeknecht 1938), France (Gijswijt 1976), Germany (Gijswijt 1976), Hungary (Erdös 1956), Netherlands (Gijswijt 1976), Russia (Yefremova 2002), Sweden (Thomson 1878), United Kingdom (Walker 1839); Canada (Hansson 1996b), USA (Girault 1916) (Fig. 510).

#### Species group *phruron*

**Diagnosis**. Forewing with admarginal setae arising from marginal vein, and with radial cell bare and long – extending well beyond postmarginal vein (e.g. Fig. 159); male flagellomeres with verticillate setae, with or without setae apical to basal whorl. Male genitalia: phallobase with volsellar setae more or less straight and attached on long or short extensions, and more or less parallel in slide mounts (Figs 486–491). Species-groups *phruron* and *salicis* share diagnostic externomorphological characters and species are difficult to assign to appropriate group using such characters. The best way to diagnose these groups is to use the appearance and orientation of the volsellar setae in the male genitalia.

##### Omphale brevis

Graham

http://species-id.net/wiki/Omphale_brevis

Figures 138 –153 486 511

Omphale brevis Graham, 1963: 257. Holotype female in OUMNH, examined.

###### Material.

**Type material.** **Holotype** female, type no. 1296 in OUMNH. **Additional material.** 24♀ 8♂: Germany 4♀ 2♂ (RMNH), Netherlands 9♀ 4♂ (RMNH), Romania 1♀ (CH), Sweden 8♀ 2♂ (BMNH, CH, NHRS), United Kingdom 2♀ (BMNH).

###### Diagnosis.

**Figures 138–145.**
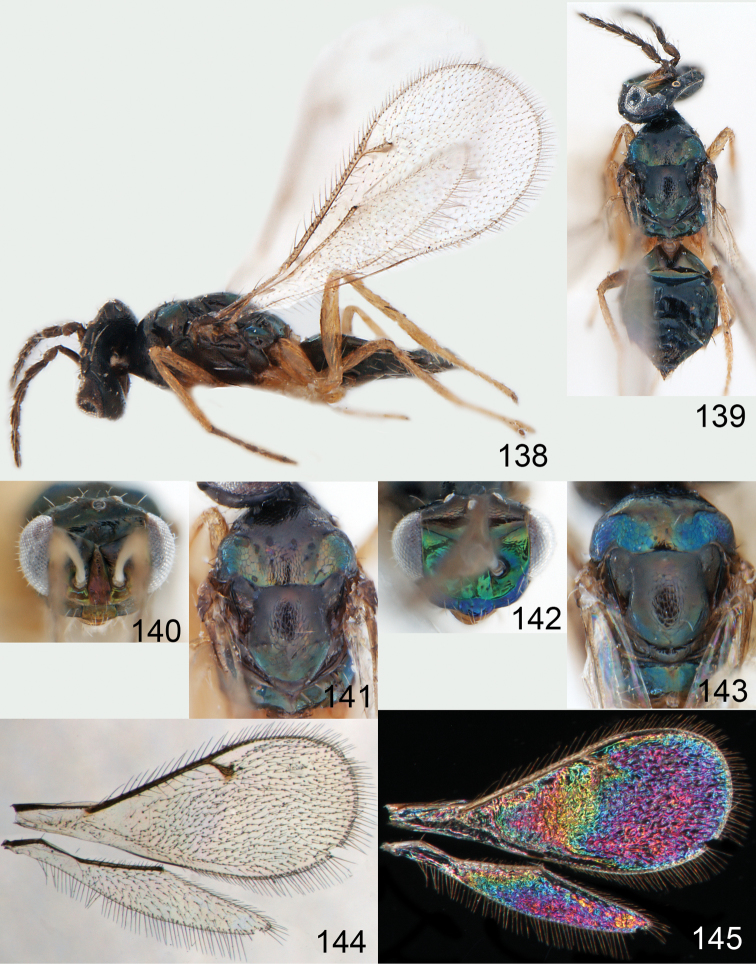
*Omphale brevis*: **138** habitus in lateral view, female, length of specimen 1.3 mm **139** habitus in dorsal view, female **140** head in frontal view, female **141** thoracic dorsum, female **142** head in frontal view, male **143** thoracic dorsum, male **144** transparent wings, female **145** wing interference patterns, female.

Female gaster short ovate, 1.0–1.4× as long as mesosoma (Fig. 139); forewing with 8–10 admarginal setae and postmarginal vein 1.4–1.5× as long as stigmal vein (Fig. 144); legs and petiole yellowish brown (Fig. 138), femora frequently dark brown; male flagellomeres 1–4 with basal setae in a whorl (Fig. 151). Similar to *Omphale phruron* but with coxae completely pale and shorter gaster in female. Male genitalia: phallobase (Fig. 486) with volsellar setae on short extensions and with apex of setae 0.4× the length of setae from apex of phallobase, digitus 0.8× as long as wide; aedeagus short and stout (Fig. 486), with penis valves 1.3× as long as wide.

###### Description.

*Female*. Length of body 0.9–1.4 mm. Antenna with scape yellowish brown with apical ⅓ dark brown, pedicel and flagellum dark brown; pedicel + flagellum 2.0× as long as distance between eyes; first flagellomere 1.1× as long and 1.3× as wide as second flagellomere (Fig. 147); flagellomeres 2–4 ventrally with one set of setae attached close to base of each flagellomere and reaching beyond apex of flagellomere attached to; clava 2-segmented. Face golden purple (Fig. 140), with weak striae (Fig. 148); clypeus golden purple, smooth, rectangular to trapezoid, 1.6× as wide as high; gena dark brown with golden purple tinges; lower frons golden purple, with weak reticulation; antennal scrobes join on frontal suture; frontal suture V-shaped; upper frons golden purple, with weak reticulation; vertex golden purple, with engraved weak reticulation (Fig. 149). Occipital margin rounded (Fig. 149).

**Figures 146–153.**
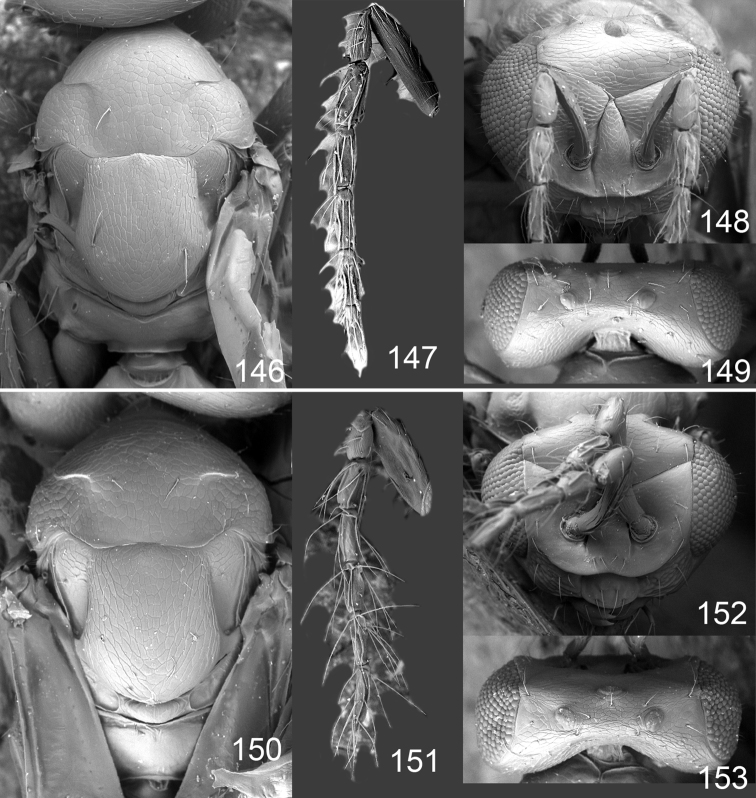
*Omphale brevis*: **146** thoracic dorsum, female **147** antenna, female **148** head in frontal view, female **149** vertex, female **150** thoracic dorsum, male **151** antenna, male **152** head in frontal view, male **153** vertex, male.

Mesoscutum with midlobe golden purplish and sidelobes golden green (Fig. 141), with engraved reticulation (Fig. 146), midlobe with one pair of setae (posterior pair); notauli as indistinct impressions in posterior ½. Scutellum golden purplish with posterior margin golden green (Fig. 141), with engraved reticulation (Fig. 146); 1.1× as long as wide; with anteromedian margin almost straight. Axillae golden purple (Fig. 141). Dorsellum golden purple (Fig. 141), slightly concave with very weak reticulation (Fig. 146), 0.4× as long as wide, and 0.6× as long as length of median propodeum. Entire lateral mesosoma golden purple (Fig. 138); transepimeral sulcus curved forwards. Propodeum golden green with median part golden purple (Fig. 141), smooth (Fig. 146); propodeal callus with two setae. Legs yellowish brown (Fig. 138), in holotype entirely so, but non-types frequently with femora pale brown to dark brown; midleg with first tarsomere 0.4× as long as length of tarsus. Forewing transparent, veins yellowish brown, setae dark brown (Fig. 144); speculum closed; admarginal setae 8–10, arising from membrane just behind marginal vein (holotype), or from ventral part of marginal vein and from membrane just behind marginal vein (in additional material); radial cell bare; postmarginal vein 1.4× as long as stigmal vein, stigmal vein slender. Hind wing transparent, apex pointed (Fig. 144). Forewing WIP (Fig. 145) with apical ½ magenta with narrow blue borders, basal ½ with wide bands in yellow, magenta and blue.

Petiole yellowish brown. Gaster dark brown with golden green and blue metallic tinges, ovate and 1.0–1.4× as long as length of mesosoma; 7^th^ tergite 0.03× as long as length of gaster.

*Male*. Length of body 1.0–1.3 mm. Antenna with scape yellowish brown in basal ½, dark brown metallic in apical ½; pedicel + flagellum 2.6× as long as distance between eyes; flagellomeres 1–4 with verticillate setae and with setae about as long as flagellomere attached to (Fig. 151), with or without a few scattered setae apical to basal whorl; clava 1-segmented. Face bluish purple metallic (Fig. 142), strigose (Fig. 152); clypeus bluish purple metallic, smooth, trapezoid to semicircular, 1.6× as wide as high; gena dark brown metallic; lower frons bluish green metalic, with engraved weak reticulation, partly smooth; antennal scrobes join frontal suture separately; upper frons with very weak reticulation.

Mesoscutum with midlobe golden purplish and sidelobes green metallic (Fig. 143). Scutellum golden purple with posterior margin green metallic (Fig. 143). Dorsellum convex with weak sculpture (Fig. 150), 0.5× as long as wide, and 0.4× as long as length of median propodeum. Entire lateral mesosoma dark brown metallic. Propodeum golden green with median part purplish (Fig. 143). Legs with coxae yellowish brown with base infuscate, femora pale brown to dark brown; tibiae yellowish brown; tarsi yellowish brown with apical tarsomere dark brown. Forewing with admarginal setae 7–9, arising from marginal vein and from membrane just behind marginal vein; postmarginal vein 1.5× as long as stigmal vein.

Petiole dark brown. Gaster dark brown metallic, 1.1× as long as length of mesosoma. Phallobase and aedeagus as in Fig. 486.

###### Hosts.

*Cystiphora taraxaci* (Diptera: Cecidomyiidae) (Gijswijt 1976), *Cystiphora sonchi* on *Sonchus palustris* (Vidal 1993); *Cystiphora sanguinea* on *Hieracium sabaudum* (Askew 2003).

###### Distribution.

Germany (Gijswijt 1976), Netherlands (Gijswijt 1976), Romania (**new record**), Sweden (Hedqvist 2003), United Kingdom (Graham 1963) (Fig. 511).

##### Omphale clymene

(Walker)

http://species-id.net/wiki/Omphale_clymene

Figures 154 –168 487 512

Entedon clymene Walker, 1839:91. Lectotype female in BMNH, examined.
Omphale clymene (Walker), Graham (1959).
Omphale clymene (Walker), Graham (1963).

###### Material.

**Type material.** Lectotype female, type no. 5.2037 in BMNH. **Additional material.** 134♀ 4♂: Denmark 1♀ (LUZM), France 8♀ (BMNH, RMNH), Germany 1♀ (RMNH), Hungary 54♀ (BMNH, CH), Netherlands 5♀ (RMNH), Slovenia 1♀ (RMNH), Sweden 38♀ 4♂ (CH), United Kingdom 26♀ (BMNH).

###### Diagnosis.

**Figures 154–160.**
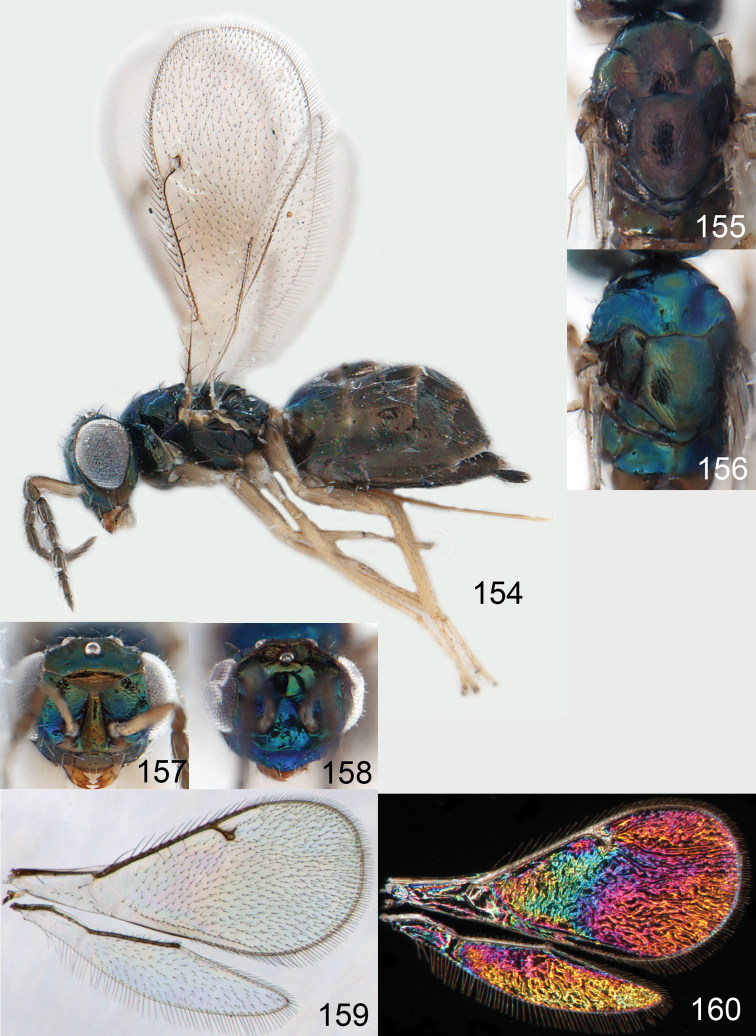
*Omphale clymene*: **154** habitus in lateral view, female, length of specimen 1.4 mm **155** thoracic dorsum, female **156** thoracic dorsum, male **157** head in frontal view, female **158** head in frontal view, male **159** transparent wings, female **160** wing interference patterns, female.

Forewing speculum open below (Fig. 159), postmarginal vein 1.8-2.0× as long as stigmal vein, with 3–6 admarginal setae; female antenna with first flagellomere slightly enlarged and distinctly wider than second flagellomere (Fig. 162), flagellomeres 1–4 with a set of long setae attached at base and reaching beyond apex of flagellomere attached to; male antenna with flagellomeres with verticillate setae (Fig. 166); coxae yellowish brown (Fig. 154); forewing with row of admarginal setae with all, or most, from ventral marginal vein and with radial cell (usually) bare (Fig. 159). Male genitalia: phallobase (Fig. 487) with volsellar setae on long extensions and with apex of setae 0.2× the length of setae from apex of phallobase, digitus triangular and as long as wide; aedeagus short and stout (Fig. 487), with penis valves 1.8× as long as wide.

###### Description.

*Female*. Length of body 1.1–1.6 mm. Antenna with scape yellowish brown with dorso-apical ⅓ dark brown; pedicel and flagellum dark brown; pedicel + flagellum 1.8× as long as distance between eyes; first flagellomere 1.0× as long and 1.3× as wide as second flagellomere (Fig. 162); flagellomeres 1–4 with scattered short setae and ventrally with a set of long setae attached at base and reaching beyond apex of flagellomere attached to; longitudinal sensilla on flagellomeres as long as flagellomere attached to; clava 2-segmented. Face dark brown with golden tinges (Fig. 157), strigose (Fig. 163); clypeus dark brown with green metallic tinges, smooth, semicircular to trapezoid, 2.0× as wide as high; gena dark brown with golden tinges; lower frons golden with green metallic tinges or spots, purple metallic, or bluish green metallic, with raised weak reticulation; interscrobal area smooth; antennal scrobes join frontal suture separately; frontal suture V-shaped; upper frons golden red with very weak reticulation, shiny; vertex golden with green tinges to purple metallic, smooth outside ocellar triangle, with very weak reticulation inside triangle (Fig. 164). Occipital margin rounded (Fig. 164).

**Figures 161–168.**
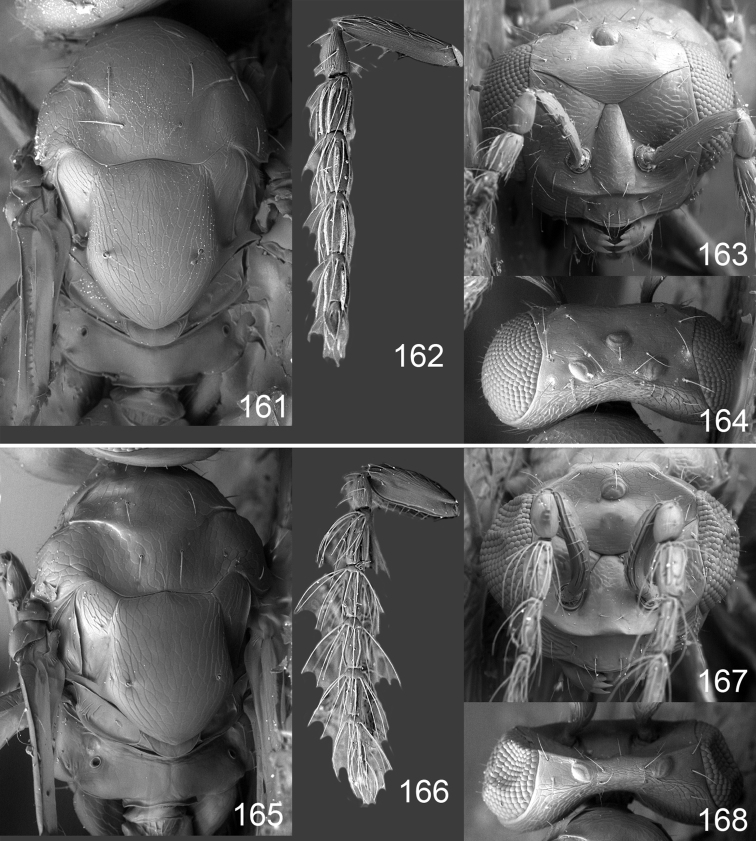
*Omphale clymene*: **161** thoracic dorsum, female **162** antenna, female **163** head in frontal view, female **164** vertex, female **165** thoracic dorsum, male **166** antenna, male **167** head in frontal view, male **168** vertex, male.

Mesoscutum golden green, bronze (Fig. 155), or blue metallic, with engraved reticulation (Fig. 161), midlobe with two pairs of setae; notauli as indistinct impressions in posterior ½. Scutellum black with green metallic tinges, golden, bronze (Fig. 155), or blue metallic, with engraved reticulation (Fig. 161); 1.2× as long as wide, with anterior margin smoothly curved forwards. Axillae black with green metallic tinges or bronze (Fig. 155). Dorsellum black with golden and green metallic tinges (Fig. 155), smooth and flat (Fig. 161), with posterior margin raised, 0.3× as long as wide, and 0.5× as long as length of median propodeum. Lateral pronotum green metallic (Fig. 154); propleuron dark brown with metallic tinges; prepectus black metallic; acropleuron dark brown; mesepisternum dark brown with metallic tinges; mesepimeron dark brown metallic; transepimeral sulcus distinctly curved. Propodeum golden with green and purple metallic tinges (Fig. 155), or blue metallic, smooth (Fig. 161); propodeal callus with two setae. Coxae yellowish brown (Fig. 154), fore- and hind coxa with base dark brown; femora, tibiae and tarsi yellow; midleg with first tarsomere 0.3× as long as length of tarsus. Forewing transparent, veins yellowish brown and setae dark brown (Fig. 159); speculum open; admarginal setae 3–5, arising from marginal vein and from membrane just below vein; radial cell bare; postmarginal vein 1.8–2.0× as long as stigmal vein; stigmal vein long and slender. Hind wing transparent, apex rounded (Fig. 159). Forewing WIP (Fig. 160) with apical ½ magenta, basal ½ predominantly blue, border between apical and basal parts a mix of magenta and blue, basal ½ with a small ovate magenta spot just behind marginal vein and just before stigmal vein.

Petiole yellow to yellowish brown. Gaster with first tergite green metallic, remaining tergites dark brown with golden, purple and green metallic tinges, smooth, elongate and 1.4–1.5× as long as length of mesosoma; 7^th^ tergite 0.09× as long as length of gaster.

*Male*. Length of body 1.1–1.2 mm. Features as in female except as follows. Antenna with scape with outer surface yellowish white, inner surface dark brown with green and blue metallic tinges, with dorsal edge dark brown; pedicel + flagellum 2.4× as long as distance between eyes; flagellomeres 1–4 with verticillate setae (Fig. 166); clava 1-segmented. Face bluish purple metallic (Fig. 158); clypeus bluish purple metallic or bluish green metallic, rectangular (Fig. 167), 1.8× as wide as high; gena golden red; lower frons blue metallic, with raised reticulation; interscrobal area with weak reticulation; upper frons bluish green metallic, smooth; vertex inside ocellar triangle purple metallic, outside triangle golden green.

Mesoscutum bluish green metallic (Fig. 156), with engraved reticulation (Fig. 165). Scutellum green metallic with blue metallic tinges (Fig. 156), with engraved reticulation (Fig. 165); 1.1× as long as wide. Axillae golden green (Fig. 156). Dorsellum blue metallic (Fig. 156). Lateral pronotum golden; propleuron blue metallic; upper mesepimeron purple metallic; lower mesepimeron dark brown metallic. Propodeum bluish green metallic (Fig. 156). Legs with coxae yellowish brown to pale brown with base dark brown; femora pale brown; tibiae and tarsi yellowish brown. Forewing tran-sparent, veins yellowish brown and setae dark brown; admarginal setae 5–6; postmarginal vein 2.0× as long as stigmal vein.

Petiole dark brown. Gaster with first tergite bluish green metallic, remaining tergites black with golden and green metallic tinges, smooth, 1.3–1.4× as long as length of mesosoma. Phallobase and aedeagus as in Fig. 487.

###### Host.

*Dasineura pyri* (Diptera: Cecidomyiidae) (**new record**).

###### Distribution.

Czech Republic (Bouček and Askew 1968), Denmark (**new record**), France (**new record**), Germany (Bouček and Askew 1968), Hungary (**new record**), Netherlands (Gijswijt 1976), Russia (Yefremova 2002), Slovenia (**new record**), Sweden (Hansson 1991), United Kingdom (Walker 1839) (Fig. 512).

##### Omphale euphorbiae sp. n.

urn:lsid:zoobank.org:act:04CDB046-5B13-4393-841D-EE11A50A25A9

http://species-id.net/wiki/Omphale_euphorbiae

Figures 169 –182 488 513

###### Material.

**Holotype** NETHERLANDS: female (RMNH), glued to a card, labelled: “Wessum, 5.vii.1972, H.J. Vlug, ex Bayeria capitigena on Euphorbia esula”. **Paratypes**. 13♀ 11♂: CZECH REPUBLIC: 2♀ “Moravia, 12 km E Boskovice, 11.vi.1996, M.J. Gijswijt” (RMNH). FRANCE: 2♀ “Dept Aisne, Cerny, xerobrometum, 2.vii.1971, M.J. Gijswijt” (RMNH); 1♂ “Dept Aisne, Cerny en Laon, Mont de Fer, 6.vii.1983, M.J. Gijswijt” (RMNH); 1♀ “Hautes Alpes, Arvieux, 8.vii.1990, Brunissard, 1750-1900m, G. Delvare” (RMNH). GREECE: 1♀ “Thesprotias, 6 km SW Igoumenitsa, 13.iv.1998, M.J. Gijswijt” (RMNH); 1♀ “Peloponnesos, Lakonia, Parori, 5 km E Sparti, 3.v.1989, M.J. Gijswijt” (RMNH). NETHERLANDS: 4♀ 2♂ with same label data as holotype (RMNH); 1♀ 7♂ “Wessum, 28.vi.1972, H.J. Vlug, gal Bayeria capitigena on Euphorbia esula, coll. 22.vii.1971” (BMNH, RMNH). SWEDEN: 1♀ “Skåne, Degeberga, 9.vii.1938” (BMNH). UNITED KINGDOM: 1♂ “England, Cambs, Woodwalton fen, 19.vii-28.viii.1978, J.S. Noyes” (BMNH).

###### Diagnosis.

**Figures 169–175.**
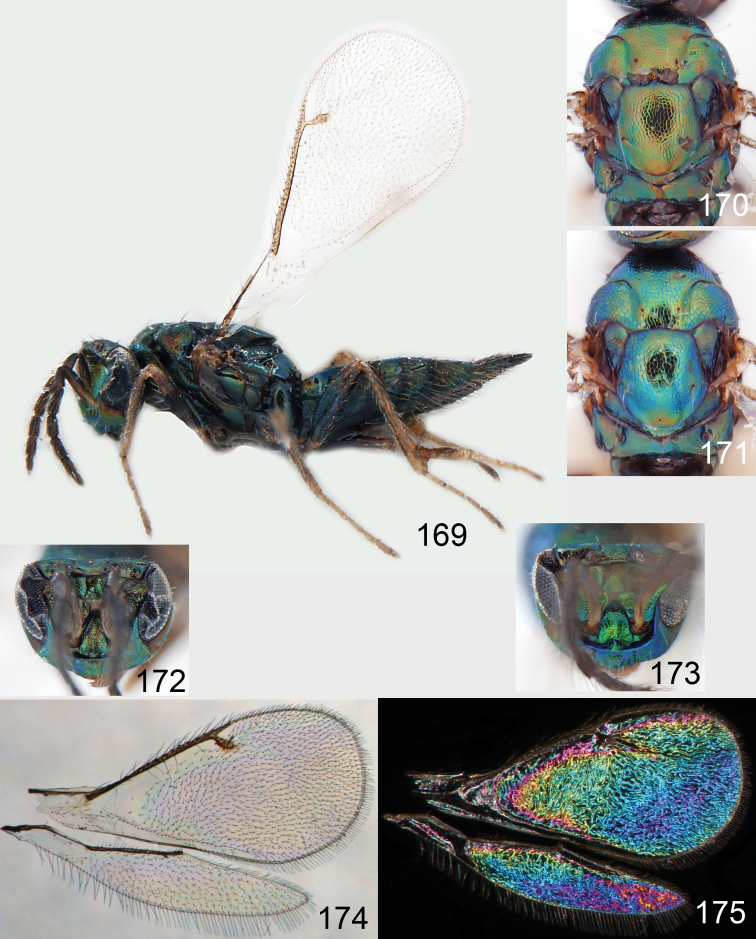
*Omphale euphorbiae*: **169** habitus in lateral view, female, length of specimen 1.8 mm **170** thoracic dorsum, female **171** thoracic dorsum, male **172** head in frontal view, female **173** head in frontal view, male **174** transparent wings, female **175** wing interference patterns, female.

**Figures 176–182.**
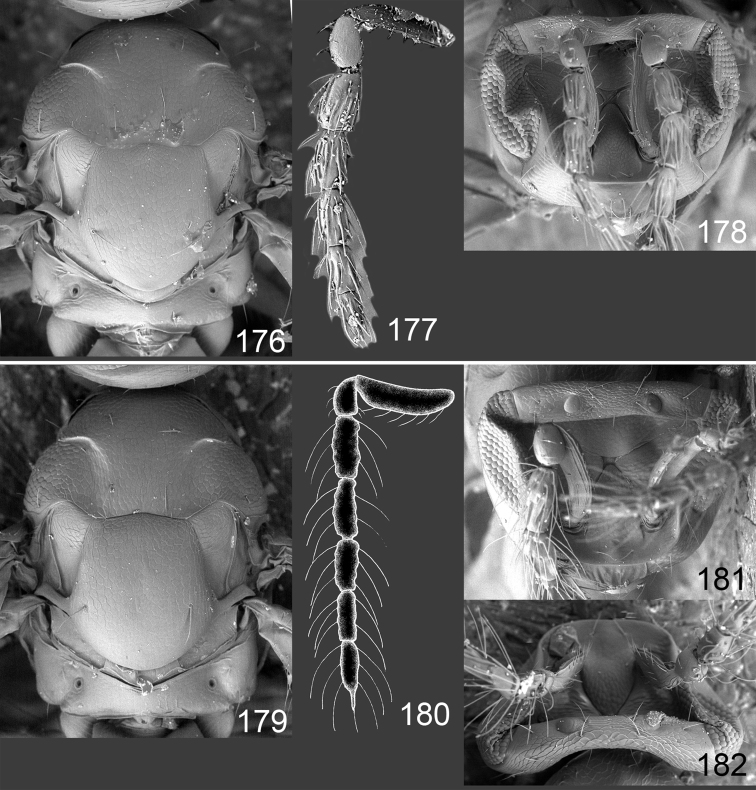
*Omphale euphorbiae*: **176** thoracic dorsum, female **177** antenna, female **178** head in frontal view, female **179** thoracic dorsum, male **180** antenna, male **181** head in frontal view, male **182** vertex, male.

Thoracic dorsum golden green or metallic bluish green (Figs 170, 171); coxae golden green and femora dark brown metallic (Fig. 169); female gaster elongate, 1.6–1.9× as long as length of mesosoma (Fig. 169); propodeum with very weak reticulation (Fig. 176). Similar to *Omphale phruron* but female flagellomeres 2–4 ventrally with two sets of setae (Fig. 177), both sexes with thoracic dorsum bright golden green or bluish green; more (9–12) admarginal setae; female gaster longer, 1.6–1.9× as long as mesosoma. Male genitalia: phallobase (Fig. 488) with volsellar setae on short extensions and with apex of setae 1.6× the length of setae from apex of phallobase, digitus 0.7× as long as wide; aedeagus short and stout (Fig. 488), with penis valves 1.5× as long as wide.

###### Description.

*Female*. Length of body 1.4–2.0 mm. Antenna with scape dark brown with base yellowish brown, pedicel and flagellum dark brown; pedicel + flagellum 1.8× as long as distance between eyes; first flagellomere 1.1× as long and 1.2× as wide as second flagellomere (Fig. 177); flagellomeres 2–4 ventrally with two sets of setae, one attached subbasally and one subapically; clava 2-segmented. Face golden red, reticulate (Fig. 178); clypeus golden green (Fig. 172), smooth, semicircular, 1.9× as wide as high; gena golden purple to golden red; lower frons golden green, with strong reticulation but smooth below toruli; antennal scrobes join on frontal suture; frontal suture V-shaped; upper frons golden red to golden green, with weak reticulation; vertex golden green inside ocellar triangle, golden red outside, with engraved weak reticulation. Occipital margin rounded.

Mesoscutum golden green (Fig. 170), with engraved reticulation (Fig. 176), midlobe with two pairs of setae; notauli as indistinct impressions in posterior ½. Scutellum golden green (Fig. 170), with engraved reticulation (Fig. 176); 1.0× as long as wide, with anterior margin almost straight. Axillae golden (Fig. 170). Dorsellum golden (Fig. 170), convex with very weak sculpture (Fig. 176), 0.2× as long as wide, and 0.6× as long as length of median propodeum. Entire lateral mesosoma golden green to golden purple (Fig. 169); transepimeral sulcus curved forwards. Propodeum golden green with median part golden red (Fig. 170), with very weak reticulation but smooth medially (Fig. 176); propodeal callus with two setae. Coxae golden green (Fig. 169); femora dark brown metallic; foretibia pale brown, mid- and hind tibiae dark brown; foretarsus dark brown, mid- and hind tarsi with tarsomeres 1–3 yellowish brown and 4 dark brown; midleg with first tarsomere 0.3× as long as length of tarsus. Forewing transparent with slight infuscation just below stigmal vein, veins yellowish brown (Fig. 174), setae dark brown; speculum closed; admarginal setae 9–12, arising from ventral marginal vein and some from membrane just behind marginal vein; radial cell bare, but with setae in posterior part; postmarginal vein 1.1× as long as stigmal vein, stigmal vein slender. Hind wing transparent, apex rounded (Fig. 174). Forewing WIP (Fig. 175) blue with margins along foremargin of wing, basal margin and basal ½ of hind margin magenta.

Petiole dark brown. Gaster with tergites 1–5 bluish green metallic with posterior margin golden purple, tergite 6 completely bluish green metallic, tergite 7 completely golden purple, elongate and 1.6–1.9× as long as length of mesosoma; 7^th^ tergite 0.1× as long as length of gaster.

*Male*. Length of body 1.1–1.4 mm. Features as in female except as follows. Antenna with scape golden green with base yellowish brown, pedicel golden green, flagellum dark brown metallic; pedicel + flagellum 1.6× as long as distance between eyes; flagellomeres 1–4 with verticillate setae and with several scattered setae apical to basal whorl (Fig. 180); clava 1-segmented. Face bluish green metallic (Fig. 173), strigose-reticulate (Fig. 181); clypeus bluish green metallic, smooth, trapezoid, 1.4× as wide as high; gena golden; lower frons golden green, with rather strong reticulation but smooth below toruli; antennal scrobes join frontal suture separately; upper frons golden green; vertex golden green.

Mesoscutum golden green to bluish green metallic (Fig. 171). Scutellum bluish green metallic (Fig. 171); 1.1× as long as wide. Axillae golden green (Fig. 171). Dorsellum bluish green metallic (Fig. 171), convex and smooth (Fig. 179), 0.3× as long as wide, and 0.5× as long as length of median propodeum. Propodeum golden green (Fig. 171). Tibiae and tarsi dark brown. Forewing admarginal setae 8–11, arising from marginal vein and from membrane just behind marginal vein; postmarginal vein 1.0× as long as stigmal vein.

Petiole dark brown. Gaster with first tergite bluish green metallic, remaining tergites dark brown to golden green, 1.0–1.2× as long as length of mesosoma. Phallobase and aedeagus as in Fig. 488.

###### Host.

*Bayeria capitigena* (Diptera: Cecidomyiidae) on *Euphorbia esula*.

###### Distribution.

Czech Republic, France, Greece, Netherlands, Sweden, United Kingdom, Netherlands (Fig. 513).

###### Etymology.

Name referring to host plant of host.

##### Omphale incognita sp. n.

urn:lsid:zoobank.org:act:13DB7447-4BA1-4D10-9A38-EC21A3CB1A2E

http://species-id.net/wiki/Omphale_incognita

Figures 183 –197 489 514

###### Material.

**Holotype** female (BMNH), glued to a card, labelled “SWEDEN: Skåne, Lake Kranke, Lottagården, 55°42'N, 13°29'E, 6.vi.2006, C. Hansson & E. Shevtsova”. **Paratypes**. 68♀ 18♂ FRANCE: 1♀ “St. Loi, 15 km W. Saint-Emiland, 14.viii.2001, J.S. Noyes” (BMNH); 1♀ ”Montals, ABPHY (Gard), 11.vii.1989, G. Delvare” (RMNH). GERMANY: 5♀”no specified locality, viii.1965, from Geocrypta galii on Galium pumilum” (RMNH); 1♀ 1♂ “Baden-Württemberg, 21.vii.1972, M.J. Gijswijt” (RMNH); 1♂ “Nordrhein-Westfalen, Brochterbeck, 20.v.1971, M.J. Gijswijt” (RMNH). HUNGARY: 9♀ “Veszprem Co., Nyirad, 47°00'N, 17°27'E, 213 m, 27.vi.2010, C. Hansson & J.S. Noyes” (BMNH, CH); 1♀ “Vas Co., Bárkás Lake, 46°52'N, 16°25'E, 28.vi.2010, C. Hansson” (BMNH). NETHERLANDS: 1♀ ”Delfzijl, 11.vi.1997, K. Alders” (RMNH). SWEDEN: 1♀ “Blekinge, Kristianopel, 21.vi.1998, R. Danielsson” (LUZM); 24♀ 4♂ with same label data as holotype (BMNH, CH, LUZM); 1♀ from same locality as holotype but collected 14.vii.2006 (BMNH); 1♀ “Skåne, Lake Kranke, West, 55°42'N, 13°27'E, 6.vi.2006, C. Hansson & E. Shevtsova” (BMNH); 3♀ 3♂ from same locality as previous but collected 8.viii.2006 (CH); 2♂ “Skåne, Silvåkra, 1.viii.1984, C. Hansson” (LUZM); 1♀ from same locality as previous but collected 2.vii.2005 (LUZM); 1♂ ”Skåne, Lake Kranke, Stensoffa, 55°42'N, 13°26'E, 8.viii.2006, C. Hansson & E. Shevtsova” (LUZM); 1♀ “Skåne, Vomb, 18.vii.2006, C. Hansson & E. Shevtsova” (LUZM); 1♂ ”Skåne, Vombsjöns SV strand, 21.vi.1983, C. Hansson” (LUZM); 5♀ 1♂ ”Skåne, Häckeberga swamp, 55°34'N, 13°25'E, 5.vii.2006, C. Hansson & E. Shevtsova” (BMNH, CH); 1♀”Skåne, Lund, 17.vii.1983, C. Hansson” (BMNH);1♀ “Skåne, Skäralid, 6-17.viii. 1994, M. Sporrong” (LUZM); 1♂ ”Närke, Örebro, 4.vii.1947, A. Jansson” (LUZM); 1♀ from same locality as previous but 14.vii.1953 (LUZM); 1♀ “Småland, Skillingaryd, 4.vii.1941, A. Jansson” (LUZM); 1♂ “Öland, Kalkstad, 56°37'N, 16°32'E, 20.vi.2007, C. Hansson” (BMNH). UNITED KINGDOM: 6♀ 1♂ ”England, Norfolk, Irrimes Graves, 25.vii.1978, J.S. Noyes” (BMNH); 1♀ ”England, Lincolnshire, Salmonby, 28.vii.1951, M.W.R. de V. Graham” (BMNH); 1♀ ”England, Berkshire, Mapledurham, 14.vi.1975, J.S. Noyes” (BMNH); 1♂ ”England, Oxfordshire, Lewknor, 13.vi.1970, M.W.R. de V. Graham” (BMNH).

###### Diagnosis.

**Figures 183–189.**
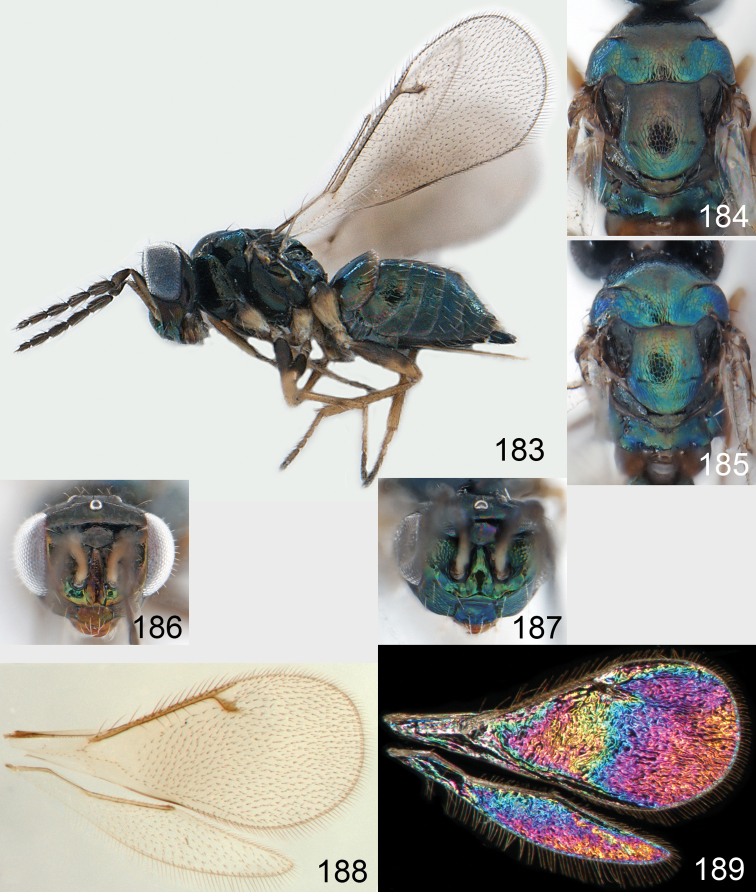
*Omphale incognita*: **183** habitus in lateral view, female, length of specimen 1.4 mm **184**  thoracic dorsum, female **185** thoracic dorsum, male **186** head in frontal view, female **187** head in frontal view, male **188** transparent wings, female **189** wing interference patterns, female.

Mesoscutum and scutellum usually bicoloured in golden purple or purple metallic and bluish green metallic (Fig. 184), and thus similar to *Omphale phruron*; female coxae bi-coloured (Fig. 183): brown with at least apices pale (yellowish brown or pale brown), femora dark brown with apices yellowish brown; tibiae yellowish brown (occasionally dark brown). Similar to *Omphale phruron* but female gaster shorter, scape predominantly pale (yellowish brown), coxae and femora with apical parts pale (yellowish brown), tibiae usually completely pale, male flagellomeres 1–4 longer and with several setae apical to basal whorl (Fig. 195). Male genitalia: phallobase (Fig. 489) with volsellar setae on long extensions and with apex of setae 0.1× the length of setae from apex of phallobase, digitus 1.6× as long as wide; aedeagus long and slender (Fig. 489), with penis valves 3.6× as long as wide. Male genitalia are thus very different from male genitalia in O. *phruron*.

###### Description.

*Female*. Length of body 0.9–1.5 mm. Antenna with scape yellowish brown with dorsal margin dark brown, pedicel and flagellum dark brown; pedicel + flagellum 1.8× as long as distance between eyes; first flagellomere 1.1× as long and 1.3× as wide as second flagellomere (Fig. 191); flagellomeres 2–4 ventrally with one set of long setae attached subbasally and reaching beyond apex of flagellomere attached to; clava 1-segmented. Face golden purple (Fig. 186), with weak striae (Fig. 192); clypeus golden with purple metallic tinges, smooth, semicircular, 1.8× as wide as high; gena golden purplish; lower frons golden purple, with weak reticulation, subtorular and interscrobal areas green metallic and smooth; antennal scrobes join on frontal suture; frontal suture V-shaped; upper frons golden with purple and green metallic tinges, with very weak reticulation; vertex golden purple, with engraved weak reticulation (Fig. 193). Occipital margin rounded (Fig. 193).

**Figures 190–197.**
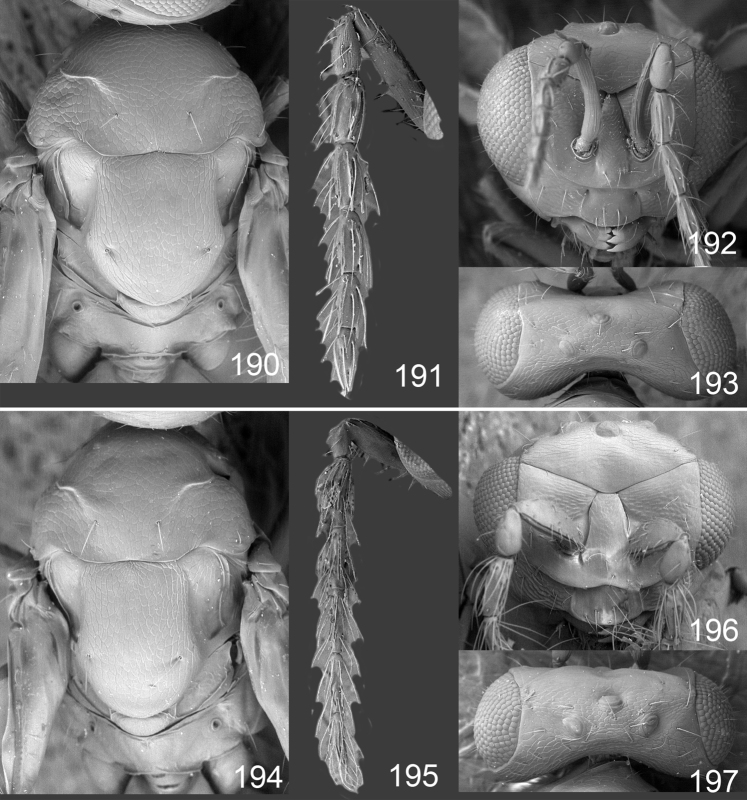
*Omphale incognita*: **190** thoracic dorsum, female **191** antenna, female **192** head in frontal view, female **193** vertex, female **194** thoracic dorsum, male **195** antenna, male **196** head in frontal view, male **197** vertex, male.

Mesoscutum with anterior ½ golden purple and posterior ½ bluish green metallic (Fig. 184), occasionally with entire midlobe golden purple, with engraved reticulation (Fig. 190), midlobe with one pair of setae (posterior pair); notauli as indistinct impressions in posterior ½. Scutellum with anterior ½–⅔ golden purple and posterior ⅓–½ bluish green metallic (Fig. 184), to completely golden purple, with engraved reticulation (Fig. 190); 1.2× as long as wide, with anterior margin almost straight. Axillae golden purple (Fig. 184). Dorsellum golden purple (Fig. 184), concave with weak reticulation (Fig. 190), 0.3× as long as wide, and 0.4× as long as length of median propodeum. Entire lateral mesosoma golden with green metallic tinges (Fig. 183); transepimeral sulcus strongly curved forwards. Propodeum golden green with median part purplish (Fig. 184), smooth (Fig. 190); propodeal callus with two setae. Coxae yellowish brown with bases brown to brown with apices yellowish brown (Fig. 183); femora dark brown with apex yellowish brown; tibiae yellowish brown, occasionally dark brown; foretarsus dark brown, mid- and hind tarsi yellowish brown becoming gradually darker towards apex; midleg with first tarsomere 0.3× as long as length of tarsus. Forewing transparent, veins yellowish brown, setae dark brown (Fig. 188); speculum closed; admarginal setae 7–11, arising from marginal vein; radial cell bare; postmarginal vein 1.0× as long as stigmal vein, stigmal vein slender. Hind wing tran-sparent, apex pointed (Fig. 188). Forewing WIP (Fig. 189) with apical ½ magenta, basal ½ with wide bands in blue, yellow and magenta.

Petiole dark brown. Gaster with first tergite bluish green metallic, remaining tergites dark brown with golden green tinges, ovate and 1.1–1.3× as long as length of mesosoma; 7^th^ tergite 0.07× as long as length of gaster.

*Male*. Length of body 0.9–1.3 mm. Features as in female except as follows. Antenna dark brown metallic; pedicel + flagellum 2.5× as long as distance between eyes; flagellomeres 1–4 with verticillate setae and with setae reaching beyond apex of flagellomere attached to, with scattered setae apical to basal whorl (Fig. 195); clava 1-segmented. Face golden red with green metallic tinges, to golden green, strigose; clypeus golden red with green metallic tinges to golden green, smooth, trapezoid to semicircular, 1.6× as wide as high; gena golden purple; lower frons golden red, golden green, or bluish green metallic (Fig. 187); antennal scrobes join frontal suture separately (Fig. 196); upper frons golden red, golden purple, or bluish green metallic, with weak reticulation; vertex dark brown with purple metallic tinges.

Mesoscutum golden red or golden purple with green metallic tinges (Fig. 185). Scutellum with anterior ⅔ golden purple, posterior ⅓ green metallic (Fig. 185), to completely golden purple or purple metallic; 1.3× as long as wide. Some Swedish speci- mens with mesoscutum and scutellum greenish blue metallic. Dorsellum golden to golden green (Fig. 185), slightly concave and smooth (Fig. 194), 0.4× as long as wide, and 0.5× as long as length of median propodeum. Lateral pronotum golden; prepectus, mesepisternum and mespeimeron dark brown metallic. Legs with coxae and femora dark brown metallic; tibiae pale brown to dark brown; tarsi yellowish brown to pale brown; midleg with first tarsomere 0.3× as long as length of tarsus. Forewing admarginal setae 6–7, arising from marginal vein; stigmal vein slightly enlarged to slender.

Petiole dark brown. Gaster with first tergite golden green, remaining tergites dark brown with golden tinges, 1.0× as long as length of mesosoma. Phallobase and aedeagus as in Fig. 489.

###### Host.

*Geocrypta galii* (Diptera: Cecidomyiidae) on *Galium* spp.

###### Distribution.

France, Germany, Hungary, Netherlands, Sweden, United Kingdom (Fig. 514).

###### Etymology.

From the Latin *incognita* = unknown.

##### Omphale lydia sp. n.

urn:lsid:zoobank.org:act:32F4138D-14EE-4A1E-8CA4-0F6205BE0C40

http://species-id.net/wiki/Omphale_lydia

Figures 198 –206 515

###### Material.

**Holotype** female (BMNH), glued to a card, labelled: “SWEDEN: Skåne, Häckeberga swamp, 55°34'N, 13°25'E, 25.vii.2006, C. Hansson & E. Shevtsova”. **Paratype.** 1♀ “SWEDEN: Skåne, Lake Kranke, Lottagården, 55°42'N, 13°29'E, 6.vi.2006, C. Hansson & E. Shevtsova” (BMNH).

###### Diagnosis.

**Figures 198–202.**
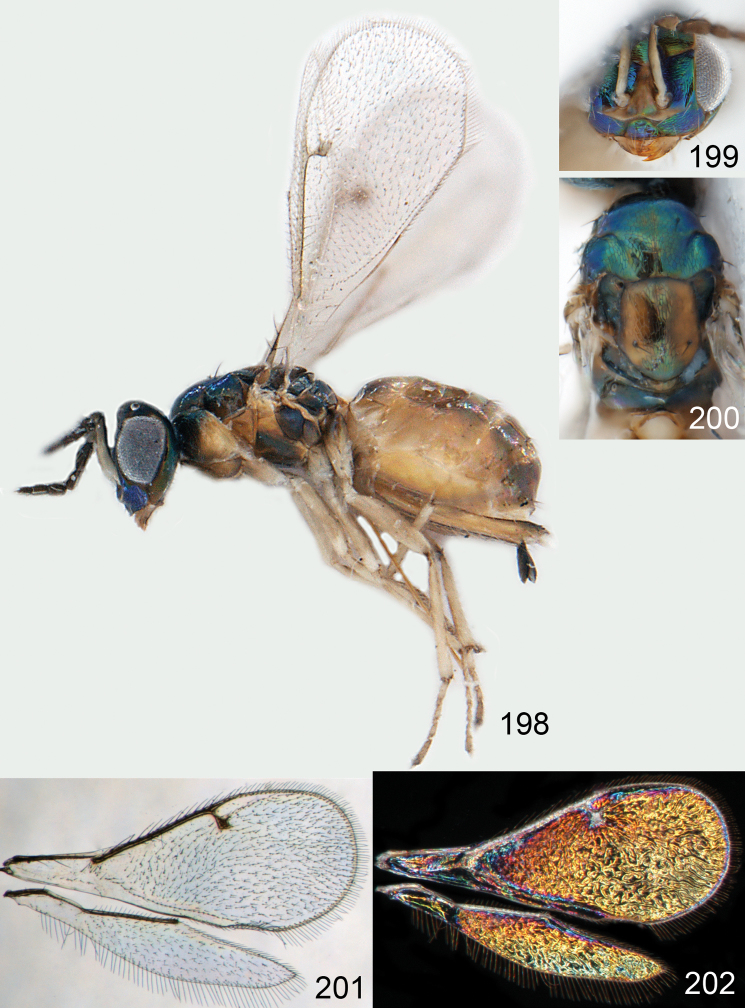
*Omphale lydia*, female: **198** habitus in lateral view, length of specimen 1.7 mm **199** head in frontal view **200** thoracic dorsum **201** transparent wings **202** wing interference patterns.

Several body parts pale non metallic: frons with interscrobal area and parts below level of toruli yellowish brown and smooth (Fig. 199); scutellum with three wide longitudinal stripes (Fig. 200), lateral ⅓ yellowish brown and median ⅓ golden; pronotum, propleuron, prepectus and mesepisternum yellowish brown (Fig. 198); legs yellowish brown with dark brown tarsi (Fig. 198); female gaster short, 1.3× as long as mesosoma (Fig. 198). Similar to *Omphale tenuicornis* but female flagellum much shorter, frons reticulate, with fewer admarginal setae (4), shorter postmarginal vein (1.0× as long as stigmal vein), and stigmal vein enlarged.

###### Description.

*Female*. Length of body 1.4–1.5 mm. Antenna with scape white with apical ⅓ and dorsal edge brown; pedicel and flagellum dark brown; pedicel + flagellum 1.9× as long as distance between eyes; first flagellomere 1.2× as long and 1.1× as wide as second flagellomere (Fig. 204); flagellomeres 2–4 ventrally with two sets of setae, one attached subbasally and one attached in apical ⅓ of flagellomeres attached to; clava 2-segmented. Face bluish green metallic (Fig. 199), strigose/reticulate (Fig. 205); clypeus purplish green metallic, smooth, semicircular, 2.0× as wide as high; gena dark brown with golden green tinges; lower frons with parts between antennal scrobes and eyes bluish green metallic with strong reticulation, interscrobal area and parts below level of toruli yellowish brown non metallic and smooth; antennal scrobes join frontal suture separately; frontal suture V-shaped; upper frons golden green, with weak reticulation; vertex dark brown with metallic tinges, with very weak reticulation (Fig. 206). Occipital margin rounded (Fig. 206).

**Figures 203–209.**
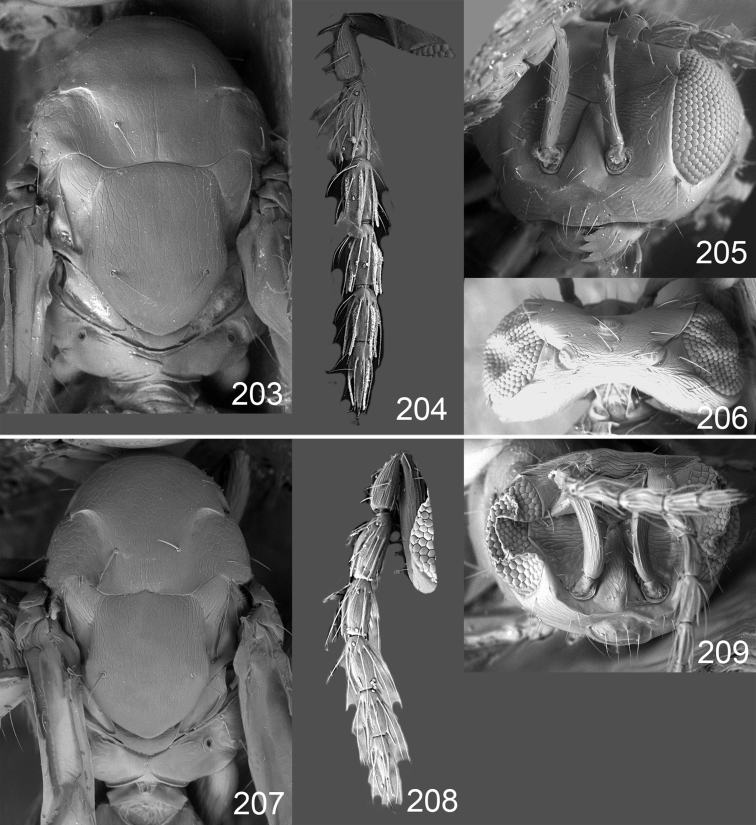
*Omphale* spp., females:**203–206**. *Omphale lydia*: **203** thoracic dorsum **204** antenna **205** head in frontal view **206** vertex **207–209**. *Omphale matrana*: **207** thoracic dorsum **208** antenna **209** head in frontal view.

Mesoscutum bluish green metallic with golden tinges (Fig. 200), with engraved reticulation (Fig. 203), midlobe with two pairs of setae; notauli as narrow grooves in anterior ½ and as indistinct impressions in posterior ½. Scutellum with lateral ⅓ yellowish brown and median ⅓ golden (Fig. 200), with engraved reticulation (Fig. 203); 1.2× as long as wide, with anterior margin smoothly curved forward. Axillae dark brown with golden tinges (Fig. 200). Dorsellum brown with metallic tinges (Fig. 200), smooth and convex (Fig. 203), 0.4× as long as wide, and 0.5× as long as length of median propodeum. Lateral pronotum and propleuron yellowish brown (Fig. 198); prepectus yellowish brown; mesepisternum yellowish brown; upper mesepimeron dark brown with bluish green metallic tinges and lower mesepimeron pale brown metallic; transepimeral sulcus curved forwards. Propodeum golden green with lateral parts brown non metallic (Fig. 200), smooth (Fig. 203); propodeal callus with two setae. Legs with coxae yellowish brown (Fig. 198), hind coxa with base dark brown metallic; femora and tibiae yellowish brown; tarsi dark brown; midleg with first tarsomere 0.4× as long as length of tarsus. Forewing transparent, veins yellowish brown and setae dark brown (Fig. 201); speculum closed; admarginal setae 4 arising from marginal vein; radial cell bare; postmarginal vein 1.0× as long as stigmal vein; stigmal vein enlarged. Hind wing transparent, apex pointed (Fig. 201). Forewing WIP (Fig. 202) yellow with narrow margins in blue.

Petiole yellowish brown. Gaster yellowish brown with metallic tinges, short ovate and 1.3× as long as length of mesosoma; 7^th^ tergite short, 0.3× as long as wide and 0.07× as long as length of gaster.

*Male*. Unknown.

###### Host.

Unknown.

###### Distribution.

Sweden (Fig. 515).

###### Etymology.

Lydia was a Kingdom where, according to Greek mythology, the Queen Omphale once reigned.

##### Omphale matrana

Erdös

http://species-id.net/wiki/Omphale_matrana

Figures 207 –214 516

Omphale matrana Erdös, 1954:337. Lectotype female in HNHM, examined.
Omphale matrana Erdös, Graham (1963).
Omphale matrana Erdös, Askew (2003).

###### Material.

**Type material.** Lectotype female, type no. 6001 in HNHM. **Additional material.** 6♀: Germany 1♀ (RMNH), Sweden 1♀ (BMNH), United Kingdom 4♀ (CH, BMNH).

###### Diagnosis.

**Figures 210–214.**
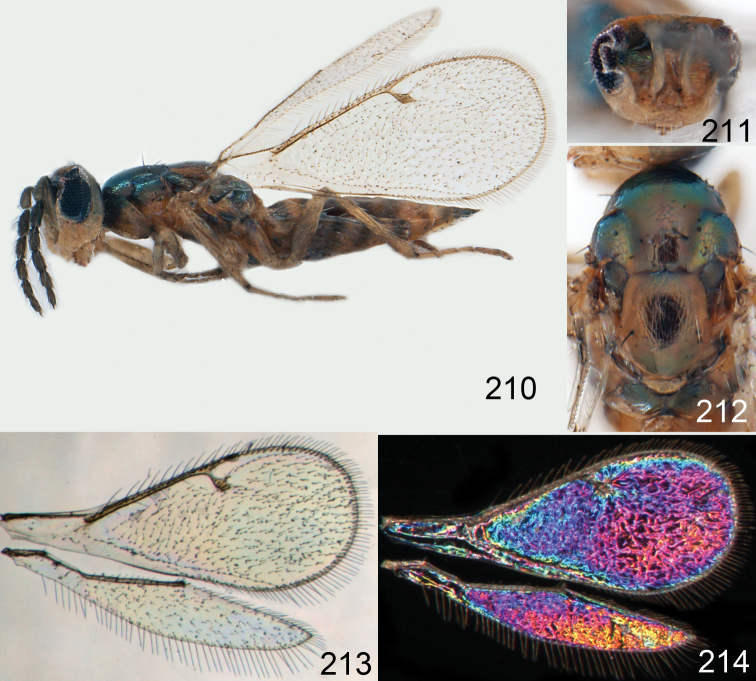
*Omphale matrana*, female: **210** habitus in lateral view, length of specimen 1.3 mm **211** head in frontal view **212** thoracic dorsum **213** transparent wings **214** wing interference patterns.

Lower ½ of head, lateral pronotum, prepectus, mesepisternum, dorsellum and legs yellow (Figs 210–212); gaster elongate, 1.4–1.5× as long as mesosoma with apex pointed, yellow with 4 transverse dark brown bands; forewing with 3–4 admarginal setae.

###### Description.

*Female*. Length of body 0.9–1.4 mm. Antenna with scape yellowish brown with dorsal edge brown; pedicel pale brown; flagellum dark brown; pedicel + flagellum 2.1× as long as distance between eyes; first flagellomere 1.1× as long and 1.1× as wide as second flagellomere (Fig. 208); flagellomeres 1–4 with a basal whorl of setae that are distinctly longer than flagellomere attached to, and with a few short and scattered setae apical to whorl; clava ±2-segmented. Face yellow (Fig. 211), with weak reticulation (Fig. 209); clypeus yellow, smooth, semicircular, 1.8× as wide as high; gena yellow; lower frons yellow, with raised reticulation, subtorular area smooth; interscrobal area smooth; antennal scrobes join on frontal suture; frontal suture V-shaped; upper frons brown with metallic tinges, with raised reticulation; vertex dark brown with metallic tinges, with very weak reticulation. Occipital margin rounded.

Mesoscutum dark brown with golden and green metallic tinges (Fig. 212), with engraved reticulation (Fig. 207), midlobe with one pair of setae (posterior pair); notauli as indistinct impressions. Scutellum with median ⅓ dark brown with golden and green metallic tinges, and lateral ⅓ yellowish brown (Fig. 212), to completely dark brown metallic, with engraved reticulation (Fig. 207); 1.2× as long as wide, with anterior margin almost straight. Axillae dark brown with golden tinges (Fig. 212). Dorsellum yellow (Fig. 212), smooth and convex (Fig. 207), 0.3× as long as wide, and 0.6× as long as length of median propodeum. Lateral pronotum, propleuron, prepectus, acropleuron and mesepisternum yellow (Fig. 210); mesepimeron dark brown with metallic tinges; transepimeral sulcus weakly curved forwards. Propodeum dark brown and golden (Fig. 212), smooth (Fig. 207); propodeal callus with two setae. Legs yellow (Fig. 210); midleg with first tarsomere 0.4× as long as length of tarsus. Forewing transparent, veins yellow and setae dark brown (Fig. 213); speculum closed; admarginal setae 4, arising from marginal vein or from membrane just behind marginal vein; radial cell bare and long, 2.2× as long as length of postmarginal vein; postmarginal vein 1.0× as long as stigmal vein; stigmal vein slender. Hind wing tran-sparent, apex pointed (Fig. 213). Forewing WIP (Fig. 214) with apical ½ magenta with blue borders, basal ½ is a mix of magenta and blue.

Petiole yellow. Gaster yellow with posterior ½ of tergites 1–4 dark brown, i.e. with dark brown transverse bands, apical parts of ovipositor sheaths black; elongate and 1.4–1.5× as long as length of mesosoma; 7^th^ tergite 0.2× as long as length of gaster.

*Male*. Unknown.

###### Host.

Unknown.

###### Distribution.

Czech Republic (Bouček and Askew 1968), Germany (Gijswijt 1976), Hungary (Erdös 1954), Sweden (Hedqvist 2003), United Kingdom (Askew 2003) (Fig. 516).

##### Omphale nitens

Graham

http://species-id.net/wiki/Omphale_nitens

Figures 215 –223 517

Omphale nitens Graham, 1963: 256. Holotype female in OUMNH, examined.

###### Material.

**Type material.** Holotype female, type no. 1295 in OUMNH. **Additional material.** 12♀: France 2♀ (BMNH), Sweden 4♀ (CH, LUZM), United Kingdom 6♀ (BMNH).

###### Diagnosis.

**Figures 215–219.**
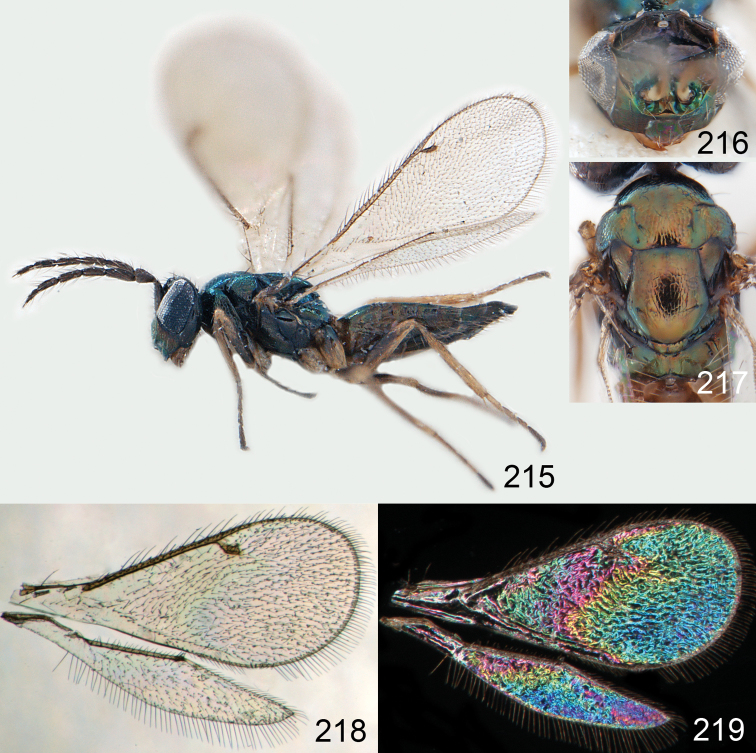
*Omphale nitens*, female: **215** habitus in lateral view, length of specimen 1.7 mm **216** head in frontal view **217** thoracic dorsum **218** transparent wings **219** wing interference patterns.

Coxae yellowish brown with base darker, femora predominantly dark brown (Fig. 215); thoracic dorsum bright golden green (Fig. 217); flagellomeres 2–4 ventrally with two sets of setae, one attached subbasally and one subapically (Fig. 221). Similar to *Omphale phruron* but female gaster shorter, fore- and midcoxae usually paler, female flagellomeres 2–4 ventrally with two sets of setae, and body with brighter colours.

###### Description.

*Female*. Length of body 1.3–1.8 mm. Antenna with scape dark brown with base yellowish brown, pedicel and flagellum dark brown; pedicel + flagellum 2.2× as long as distance between eyes; first flagellomere 1.2× as long and 1.2× as wide as second flagellomere (Fig. 221); flagellomeres 2–4 ventrally with two sets of setae, one attached subbasally and one subapically on each flagellomere; clava 1-segmented. Face golden purple (Fig. 216), strigose-reticulate (Fig. 222); clypeus golden green, smooth, semicircular, 1.4× as wide as high; gena purple metallic; lower frons golden purple, with very weak reticulation and shiny, to smooth; antennal scrobes join on frontal suture; frontal suture V-shaped; upper frons purple metallic, with very weak reticulation and shiny, to smooth; vertex purple metallic, with engraved weak reticulation (Fig. 223). Occipital margin rounded (Fig. 223).

**Figures 220–227.**
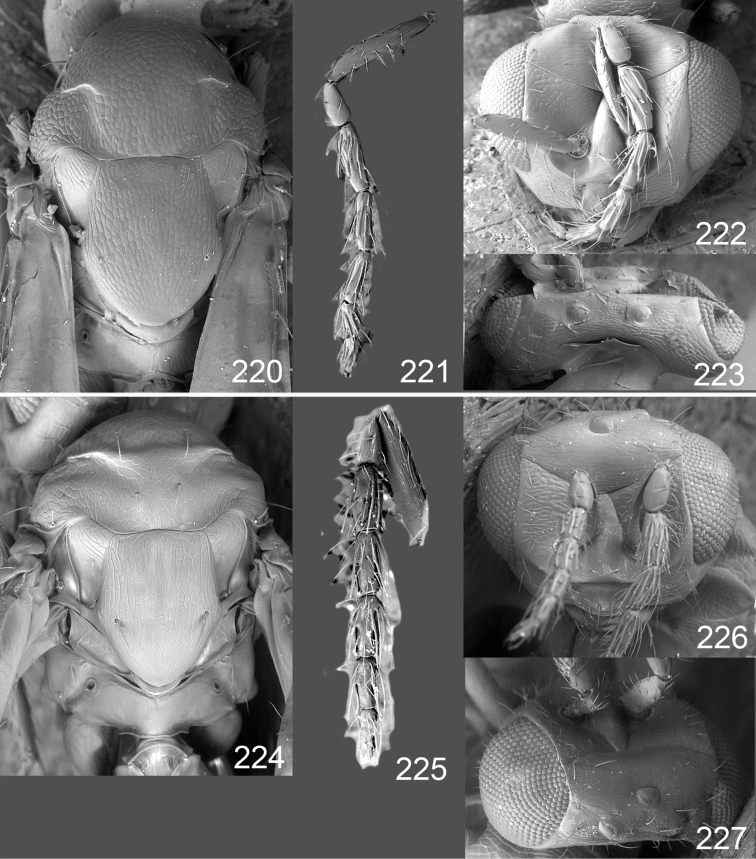
*Omphale* spp., females:**220–222**. *Omphale nitens*: **220** thoracic dorsum **221** antenna **222** head in frontal view **223** vertex **224–227**. *Omphale sti*: **224** thoracic dorsum **225** antenna **226** head in frontal view **227** vertex.

Mesoscutum golden green (Fig. 217), with engraved reticulation (Fig. 220), midlobe with one pair of setae (posterior pair); notauli as indistinct impressions in posterior ½. Scutellum golden green (Fig. 217), with engraved reticulation (Fig. 220); 1.2× as long as wide, with anteromedian margin almost straight. Axillae golden green (Fig. 217). Dorsellum golden green (Fig. 217), convex and smooth (Fig. 220), 0.3× as long as wide, and 0.6× as long as length of median propodeum. Lateral pronotum golden green (Fig. 215), remaining parts of lateral mesosoma golden with green and purple metallic tinges; transepimeral sulcus curved forwards. Propodeum golden green (Fig. 217), smooth (Fig. 220); propodeal callus with two setae. Forecoxa yellowish brown with base dark brown (Fig. 215), to predominantly dark brown with only apex yellowish brown, midcoxa yellowish brown with base pale brown, hind coxa yellowish brown with base golden green; femora predominantly dark brown with apex yellowish brown; tibiae yellowish brown; foretarsus dark brown, mid- and hind tarsi yellowish brown with 4^th^ tarsomere dark brown to completely dark brown; midleg with first tarsomere 0.3× as long as length of tarsus. Forewing transparent, veins yellowish brown, setae dark brown (Fig. 218); speculum closed; admarginal setae 7–9, arising from ventral marginal vein; radial cell with bare part small, with setae in posterior part; postmarginal vein 1.4× as long as stigmal vein, stigmal vein slender. Hind wing transparent, apex pointed (Fig. 218). Forewing WIP (Fig. 219) with apical ½ blue, basal ½ with wide bands in yellow, magenta and blue.

Petiole pale brown. Gaster with first tergite golden green, remaining tergites golden purple, slightly elongate and 1.3–1.5× as long as length of mesosoma; 7^th^ tergite 0.1× as long as length of gaster.

*Male*. Unknown

###### Host.

Unknown.

###### Distribution.

France (**new record**), Sweden (**new record**), United Kingdom (Graham 1963) (Fig. 517).

##### Omphale phruron

(Walker)

http://species-id.net/wiki/Omphale_phruron

Figures 3, 13 228 –242 490 518

Entedon phruron Walker, 1839:87. Lectotype male in BMNH, examined.
Omphale phruron (Walker), Graham (1963).
Omphale teresis Askew, 2003:29. Holotype female in BMNH, examined. **Syn. n.**

###### Material.

**Type material.** Lectotype male of *Entedon phruron*, type no. 5.3386 in BMNH; Holotype female of *Omphale teresis*, type no. 5.4614 in BMNH. **Additional material.** 100♀ 154♂: Denmark 1♂ (LUZM), France 2♀ 3♂ (RMNH), Germany 5♀ 7♂ (RMNH), Hungary 6♀ 9♂ (BMNH, CH), Ireland 3♀ 1♂ (BMNH), Netherlands 2♀ 1♂ (RMNH), Russia 1♀ (BMNH), Sweden 35♀ 74♂ (BMNH, CH, LUZM), United Kingdom 46♀ 58♂ (BMNH).

###### Diagnosis.

**Figures 228–234.**
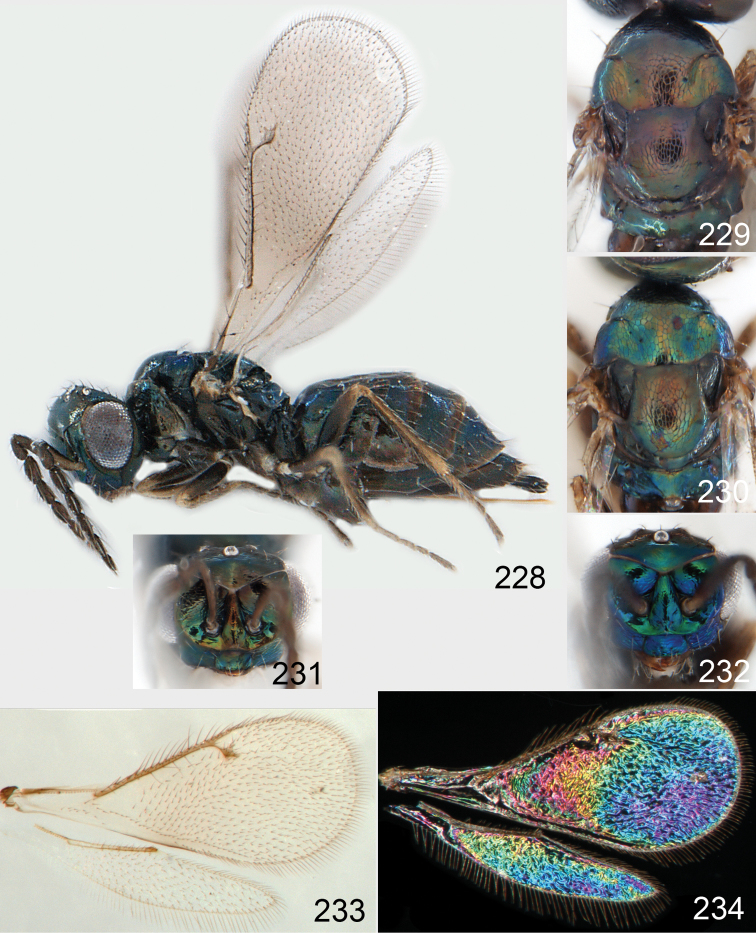
*Omphale phruron*: **228** habitus in lateral view, female, length of specimen 1.2 mm **229** thoracic dorsum, female **230** thoracic dorsum, male **231** head in frontal view, female **232** head in frontal view, male **233** transparent wings, female **234** wing interference patterns, female.

A small species (0.9–1.4 mm) with dark brown scape, coxae and femora (Fig. 228), female gaster elongate (1.6× as long as length of mesosoma) with apex distinctly pointed, mesoscutum and scutellum usually bicoloured in golden purple or purple metallic (occasionally golden red) and golden green or green metallic (Figs 229, 230); female flagellomeres 2–4 ventrally with one set of setae attached subbasaly and reaching beyond apex of flagellomere attached to (Fig. 236), male flagellomeres 1–4 with a basal whorl of setae and with setae reaching beyond apex of flagellomere attached to (Fig. 240), some specimens with a few setae apical to whorl. Male genitalia: phallobase (Fig. 490) with digitus triangular and 0.6× as long as wide, volsellar setae on long extensions and with apex of setae 1.4× the length of setae from apex of phallobase; aedeagus short and stout (Fig. 490), with penis valves 1.5× as long as wide.

###### Description.

*Female*. Length of body 1.1–1.4 mm. Antenna dark brown; pedicel + flagellum 1.8× as long as distance between eyes; first flagellomere 1.0× as long and 1.3× as wide as second flagellomere (Fig. 236); flagellomeres 2–4 ventrally with one set of setae attached subbasally and reaching beyond apex of flagellomere attached to; clava 2-segmented. Face dark brown with golden green and purple metallic tinges (Fig. 231), with weak striae (Fig. 237); clypeus golden green, golden red, or purple metallic, smooth, semicircular, 1.8× as wide as high; gena purple metallic; lower frons golden with green metallic tinges, with weak reticulation; antennal scrobes join on frontal suture; frontal suture V-shaped; upper frons golden purple, with very weak reticulation; vertex golden purple, inside ocellar triangle with engraved weak reticulation outside triangle smooth or with very weak reticulation, to completely with engraved weak reticulation (Fig. 238). Occipital margin rounded (Fig. 238).

**Figures 235–242.**
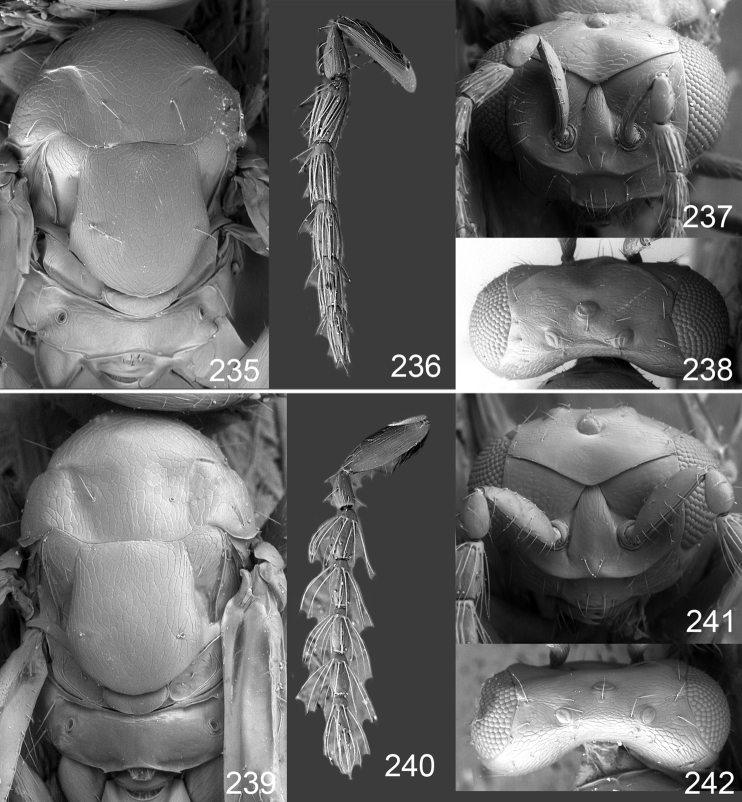
*Omphale phruron*: **235** thoracic dorsum, female **236** antenna, female **237** head in frontal view, female **238** vertex, female **239** thoracic dorsum, male **240** antenna, male **241** head in frontal view, male **242** vertex, male.

Mesoscutum golden with posterior ¼ green metallic, to completely golden purple (Fig. 229), with engraved reticulation (Fig. 235), midlobe with one pair of setae (posterior pair); notauli as indistinct impressions in posterior ½. Scutellum with anterior ⅔ golden purple, posterior ⅓ green metallic (Fig. 229), to completely golden purple or purple metallic, with engraved reticulation (Fig. 235); 1.1× as long as wide, with anteromedian margin smoothly curved forwards. Axillae golden purple (Fig. 229). Dorsellum golden green (Fig. 229), slightly concave and smooth (Fig. 235), 0.3× as long as wide, and 0.5× as long as length of median propodeum. Lateral pronotum golden (Fig. 228); prepectus, mesepisternum and mespeimeron dark brown metallic; trans-epimeral sulcus curved forwards. Propodeum golden green with median part purplish (Fig. 229), smooth (Fig. 235); propodeal callus with two setae. Legs with coxae and femora dark brown metallic (Fig. 228); tibiae pale brown to dark brown; tarsi yellowish brown to pale brown; midleg with first tarsomere 0.3× as long as length of tarsus. Forewing transparent, veins yellowish brown, setae dark brown (Fig. 228); speculum closed; admarginal setae 6–7 (lectotype male with 7), arising from marginal vein; radial cell bare; postmarginal vein 1.0× as long as stigmal vein, stigmal vein slightly enlarged to slender. Hind wing transparent, apex pointed (Fig. 233). Forewing WIP (Fig. 234) with apical ½ blue and magenta, basal ½ with wide bands in yellow, magenta and blue.

Petiole dark brown. Gaster dark brown with golden green tinges, elongate and 1.5–1.6× as long as length of mesosoma and with apex distinctly pointed; 7^th^ tergite 0.1× as long as length of gaster.

*Male*. Length of body 0.9–1.3 mm. Features as in female except as follows. Antenna dark brown metallic; pedicel + flagellum 2.5× as long as distance between eyes; flagellomeres 1–4 with verticillate setae and with setae reaching beyond apex of flagellomere attached to (Fig. 240), some specimens with a few setae apical to whorl; clava 1-segmented. Face golden red with green metallic tinges, to golden green, strigose; clypeus golden red with green metallic tinges, to golden green, smooth (Fig. 241), trapezoid to semicircular, 1.6× as wide as high; gena golden purple; lower frons golden red, golden green, or bluish green metallic (Fig. 232), with weak reticulation; antennal scrobes join frontal suture separately; upper frons golden red, golden purple, or bluish green metallic, with weak reticulation; vertex dark brown with purple metallic tinges.

Mesoscutum golden red or golden purple with green metallic tinges (Fig. 230). Scutellum with anterior ⅔ golden purple posterior ⅓ green metallic (Fig. 230), to completely golden purple or purple metallic, with engraved reticulation (Fig. 239); 1.3× as long as wide. Some Swedish specimens with mesoscutum and scutellum greenish blue metallic. Dorsellum golden to golden green (Fig. 230), 0.4× as long as wide (Fig. 239).

Petiole dark brown. Gaster with first tergite golden green, remaining tergites dark brown with golden tinges, 1.5–1.6× as long as length of mesosoma. Phallobase and aedeagus as in Fig. 490.

###### Hosts.

*Dasineura pyri* (Diptera: Cecidomyiidae) (Gijswijt 1976). The record for *Geocrypta galii* in Gijswijt (1976) concerns *Omphale incognita*. Material for *Dasineura pyri* not seen.

###### Distribution.

Denmark (**new record**), France (Gijswijt 1976), Germany (Gijswijt 1976), Hungary (**new record**), Ireland (**new record**), Netherlands (Gijswijt 1976), Russia (**new record**), Sweden (Hansson 1991), United Kingdom (Walker 1839) (Fig. 518).

###### Remarks.

Askew (2003) described *Omphale teresis* from four female specimens and compared it to *Omphale phruron*, which Askew considered the most similar species. According to Askew *Omphale teresis* differed from *Omphale phruron* in having fewer admarginal setae on the forewing, a greener and shinier thoracic dorsum that lacked sculpture on posterior part of both meso-scutum and scutellum, and in having a narrower mesosoma. The female holotype of *Omphale teresis* has 7 admarginal setae (i.e. same as lectotype male of *Omphale phruron*), the holotype of *Omphale teresis* has entire mesoscutum and scutellum reticulate and there are no smooth parts, and the scutellum is bi-coloured with anterior ⅔ golden-purple and posterior ⅓ green metallic, the mesosoma is 1.3× as long as wide (not “at least 1.5×” as stated by Askew). These data contradict the characters given by Askew and the holotype of *Omphale teresis* fits well into the concept of *Omphale phruron*, and *Omphale teresis* is therefore synonymized with *Omphale phruron*.

##### Omphale sti sp. n.

urn:lsid:zoobank.org:act:99A10FE3-6EC0-476B-8154-401F173D05A3

http://species-id.net/wiki/Omphale_sti

Figures 224–227 243–247 519

###### Material.

**Holotype** female (BMNH), glued to a card, labelled “SWEDEN: Skåne, Lake Kranke, 17.vii.2004, C. Hansson”. **Paratype.** SWEDEN: 1♀ with same label data as holotype (BMNH).

###### Diagnosis.

Female with flagellomeres 2-4 ventrally with one set of long setae attached subbasally and reaching beyond apex of flagellomere attached to (Fig. 225), and first flagellomere distinctly wider than second. Similar to *Omphale phruron* but midlobe of mesoscutum with two pairs of setae (Fig. 224); forewing speculum open below (Fig. 246); WIP different, medially with distinct lines (Fig. 247).

###### Description.

*Female*. Length of body 1.3–1.5 mm. Antenna with scape yellowish brown with dorsal margin dark brown, pedicel and flagellum dark brown; pedicel + flagellum 1.8× as long as distance between eyes; first flagellomere 1.1× as long and 1.1× as wide as second flagellomere (Fig. 225); flagellomeres 2–4 ventrally with one set of long setae attached subbasally and reaching beyond apex of flagellomere attached to; clava 1-segmented. Face golden green (Fig. 244), with striae (Fig. 226); clypeus blue metallic, smooth, rectangular, 1.9× as wide as high; gena golden purple; lower frons bluish green metallic, with raised and weak reticulation, interscrobal area golden green with very weak reticulation; antennal scrobes join frontal suture separately; frontal suture V-shaped; upper frons golden, with very weak reticulation; vertex golden green, with engraved weak reticulation (Fig. 227). Occipital margin rounded (Fig. 227).

**Figures 243–247.**
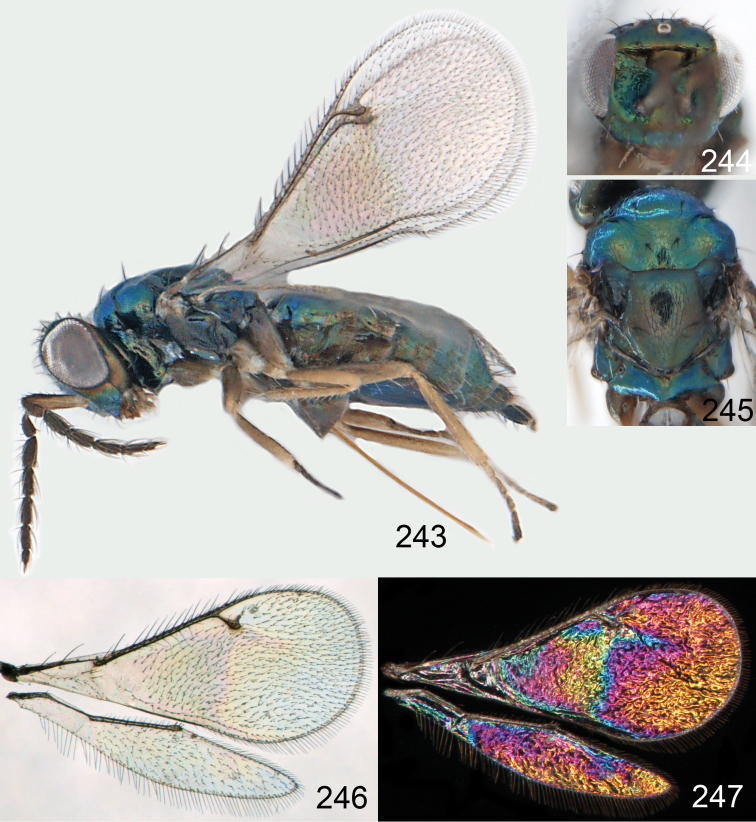
*Omphale sti*, female: **243** habitus in lateral view, length of specimen 1.5 mm **244** head in frontal view **245** thoracic dorsum **246** transparent wings **247** wing interference patterns.

Mesoscutum golden green (Fig. 245), with engraved reticulation (Fig. 224), midlobe with two pairs of setae; notauli as indistinct impressions in posterior ½. Scutellum golden with green tinges (Fig. 245), with engraved reticulation (Fig. 224); 1.2× as long as wide, with anterior margin almost straight. Axillae golden with green tinges (Fig. 245). Dorsellum golden (Fig. 245), concave and smooth (Fig. 224), 0.2× as long as wide, and 0.3× as long as length of median propodeum. Entire lateral mesosoma purple metallic (Fig. 243); transepimeral sulcus curved forwards. Propodeum bluish green metallic (Fig. 245), smooth (Fig. 224); propodeal callus with two setae. Fore- and midcoxae yellowish brown, hind coxae purple metallic with apical ⅓ yellowish-brown (Fig. 243); femora pale brown; fore- and midtibiae pale brown, hind tibia yellowish brown; tarsi yellowish brown; midleg with first tarsomere 0.2× as long as length of tarsus. Forewing transparent, veins yellowish brown, setae dark brown (Fig. 246); speculum open; admarginal setae 5, arising from marginal vein; radial cell bare; stigmal vein slender; postmarginal vein 1.5× as long as stigmal vein. Hind wing transparent, apex pointed (Fig. 246). Forewing WIP (Fig. 247) with apical ⅓ yellow, basal ⅔ with wide bands in magenta, blue and yellow.

Petiole dark brown. Gaster with first tergite golden green, remaining tergites golden with purple tinges, elongate and 1.5–1.6× as long as length of mesosoma; 7^th^ tergite 0.2× as long as length of gaster.

*Male*. Unknown.

###### Host.

Unknown.

###### Distribution.

Sweden (Fig. 519).

###### Etymology.

Named after the Swedish Taxonomy Initiative, a commendable initiative that supports taxonomy and systematics in Sweden.

##### Omphale tenuicornis sp. n.

urn:lsid:zoobank.org:act:4771FC2B-0ED1-4F8C-B8CA-7C8233638910

http://species-id.net/wiki/Omphale_tenuicornis

Figures 248 –262 491 520

###### Material.

**Holotype** female (BMNH), glued to a card, labelled “SWEDEN: Öland, Ismantorp, 56°45'N, 16°39'E, 20.vi.2007, C. Hansson”. **Paratypes**. 17♀ 2♂: HUNGARY: 1♀ “4-5 km SW Köszeg, Meszas Völgy, 47°22'N, 16°31'E, 431m, 26.vi.2010, J.S. Noyes” (BMNH); 1♀ ”Örzeg Nemzeti Park, Lugosi Valley, 46°54'N, 16°27'E, 231m, 28.vi.2010, J.S. Noyes” (BMNH). RUSSIA: 12♀ ”Gatchina, 25.vii.2006, E. Shevtsova” (BMNH, CH, LUZM). SWEDEN: 2♀ 2♂ with same label data as holotype (BMNH); 1♀ “Skåne, Silvåkra, 1.viii.1984, C. Hansson” (BMNH).

###### Diagnosis.

**Figures 248–254.**
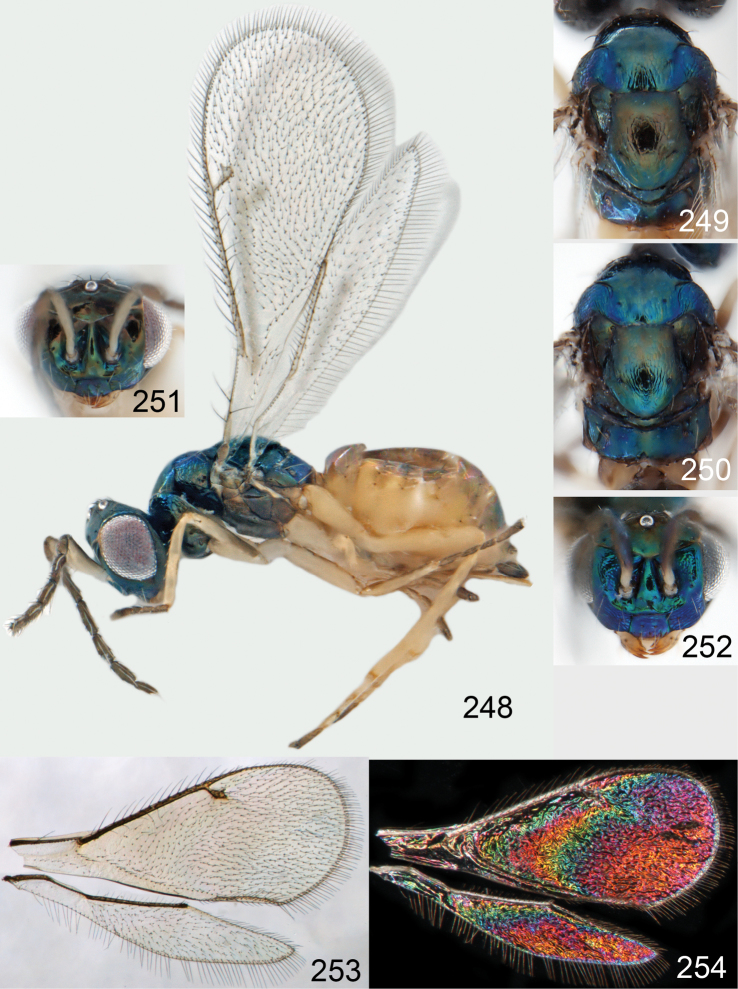
*Omphale tenuicornis*: **248** habitus in lateral view, female, length of specimen 1.5 mm **249** thoracic dorsum, female **250** thoracic dorsum, male **251** head in frontal view, female **252** head in frontal view, male **253** transparent wings, female **254** wing interference patterns, female.

Antennal flagellum very slender (Figs 256, 260); frons smooth and shiny (Figs 257, 261); coxae yellowish white, remaining parts of legs darker (yellowish brown to dark brown) (Fig. 248); female gaster short ovate, 1.3× as long as length of mesosoma (Fig. 248). Male genitalia: phallobase (Fig. 491) with volsellar setae on short extensions and with apex of setae 0.8× the length of setae from apex of phallobase, digitus 1.3× as long as wide; aedeagus long and slender (Fig. 491), with penis valves 2.7× as long as wide.

###### Description.

*Female*. Length of body 1.3–1.5 mm. Antenna with scape pale brown with basal part yellowish white and with dorsal edge dark brown; pedicel and flagellum dark brown; pedicel + flagellum 2.6× as long as distance between eyes; first flagellomere 1.1× as long and 1.3× as wide as second flagellomere (Fig. 256); flagello- meres 2–4 ventrally with two sets of setae, one attached at base and one attached in apical 1/3 of flagellomeres; clava 1-segmented. Face golden with green and red metallic tinges (Fig. 251), strigose (Fig. 257); clypeus greenish blue metallic, smooth, trapezoid, 1.9× as wide as high; gena golden purple; frontal cross-ridge present; lower frons golden, smooth or with engraved very weak reticulation, subtorular area smooth; interscrobal area golden green, smooth; antennal scrobes join on frontal suture; frontal suture V-shaped; upper frons golden, smooth or with engraved very weak reticulation; vertex golden purple, close to eyes golden, with engraved reticulation, outside ocellar triangle partly smooth (Fig. 258). Occipital margin rounded (Fig. 258).

**Figures 255–262.**
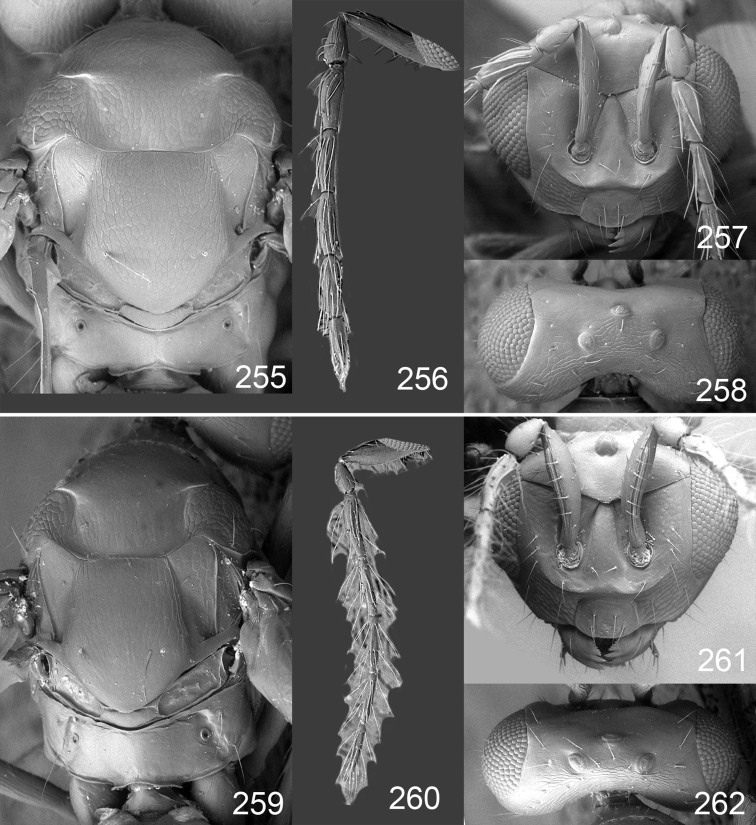
*Omphale tenuicornis*: **255** thoracic dorsum, female **256** antenna, female **257** head in frontal view, female **258** vertex, female **259** thoracic dorsum, male **260** antenna, male **261** head in frontal view, male **262** vertex, male.

Mesoscutum bluish green metallic (Fig. 249), with engraved reticulation (Fig. 255), midlobe with one pair of setae (posterior pair); notauli as narrow grooves in anterior ½ and as indistinct impressions in posterior ½. Scutellum golden with posterior margin green metallic (Fig. 249), with engraved strong reticulation (Fig. 255); 1.0× as long as wide, with anterior margin straight. Axillae golden (Fig. 249). Dorsellum golden green (Fig. 249), smooth and slightly convex (Fig. 255), 0.4× as long as wide, and 0.5× as long as length of median propodeum. Lateral pronotum and propleuron golden green with purple metallic tinges (Fig. 248); prepectus dark brown metallic; mesepisternum dark brown metallic; mesepimeron dark brown metallic; transepimeral sulcus strongly curved forwards. Propodeum green metallic (Fig. 249), smooth (Fig. 255); propodeal callus with two setae. Legs with coxae yellowish white (Fig. 248); femora dark brown to pale brown with ventral part yellowish brown; tibiae yellowish brown; foretarsus dark brown, mid- and hind tarsi yellowish brown with 4^th^ tarsomere dark brown, or all tarsi dark brown; midleg with first tarsomere 0.3× as long as length of tarsus. Forewing transparent, veins yellowish white and setae dark brown (Fig. 253); speculum closed; admarginal setae 7–8 arising from marginal vein; radial cell bare; postmarginal vein 1.6× as long as stigmal vein; stigmal vein slender. Hind wing transparent, apex pointed (Fig. 253). Forewing WIP (Fig. 254) with apical ½ magenta, basal ½ with wide bands in blue, yellow and magenta.

Petiole yellowish brown. Gaster pale brown metallic, short ovate and 1.3× as long as length of mesosoma; 7^th^ tergite short, 0.3× as long as wide and 0.05× as long as length of gaster.

*Male*. Length of body 1.4 mm. Features as in female except as follows. Antenna with scape expanded, distinctly wider than in female (Fig. 260), dark brown with basal ⅓ yellowish white; pedicel + flagellum 3.1× as long as distance between eyes; first flagellomere 1.2× as long as second; flagellomeres 1–4 with verticillate setae and with setae apical to whorl. Face bluish purple metallic (Fig. 252); clypeus bluish purple metallic, semicircular, 1.5× as wide as high; gena golden with green metallic tinges; lower frons bluish green metallic with purple tinges; interscrobal area with very weak reticulation; antennal scrobes join frontal suture separately; upper frons golden green, smooth; vertex inside ocellar triangle golden purple, outside golden green.

Mesoscutum with anterior ½ golden green, posterior ½ bluish green metallic (Fig. 250). Scutellum golden with posterior ⅓ bluish green metallic (Fig. 250), with engraved weak reticulation (Fig. 259); 1.1× as long as wide. Dorsellum 0.2× as long as wide, and 0.2× as long as length of median propodeum. Lateral pronotum and propleuron bluish green metallic; mesepisternum pale brown metallic. Propodeum golden green to bluish green metallic (Fig. 250). Legs with coxae yellowish white with base pale brown; femora dark brown with ventral margin yellowish. Forewing veins pale brown; admarginal setae 5, arising mainly from marginal vein; postmarginal vein 1.5× as long as stigmal vein.

Petiole dark brown. Gaster dark brown, shiny; 1.3× as long as length of mesosoma. Phallobase and aedeagus as in Fig. 491.

###### Host. 

Unknown.

###### Distribution.

Hungary, Russia, Sweden (Fig. 520).

###### Etymology.

From the Latin *tenuis* = thin, and *cornu* = horn, referring to the long and slender antennae.

#### Species group *aetius*

**Diagnosis**. Forewing with majority of admarginal setae arising from membrane behind marginal vein, and with radial cell hairy (Figs 9, 12); frons with surface between lower margin of eye and mouth opening smooth (Fig. 6), sides of clypeus straight, and with a frontal cross-ridge; flagellomeres with short and asymmetric sensilla; male flagellomeres with scattered arrangement of setae (Fig. 4). Male genitalia very distinctive (Figs 492–494): aedeagus with protruding chitin plates close to apex of penis valves; phallobase with weak volsellar setae and with two extra spines on lateral part of digitus.

The majority of species in this group are impossible to separate morphologically even with some degree of certainty. Apart from four species (*Omphale aethiops*, *Omphale connectens*, *Omphale dolichura* and *Omphale lugubris*) with diagnostic enough morphological characters the remainder of the species are inseparable. In previous studies (Graham 1959, 1963) these species have been separated in the female sex only, males were not possible to distinguish. In the latest study Graham (1963) thus separated the following species in a key: *Omphale acamas* (Walker), *Omphale aetius* (Walker), *Omphale betulicola* Graham, *Omphale coilus* (Walker), *Omphale epaphus* (Walker), *Omphale grahami* Gijswijt (this species was as “sp. indet.” in couplet 26, and was later described by Gijswijt (1976) who referred to the Graham key for distinguishing characters), *Omphale phaola* (Walker), and *Omphale varipes* (Thomson). Graham used the colour of the head, thoracic dorsum and legs; length of gaster, antenna and postmarginal vein; and body size to separate these species. With the large material of this group available for this study, including about 1500 specimens from BMNH (mainly the Graham collection of unidentified *Omphale*), RMNH (the Gijswijt collection) and material collected in nature for this project, variations in the characters used in the Graham key become evident. These variations are extensive and continuous, and make it impossible to separate the material into morphologically distinct groups. To check if the new characters introduced in this article were useful for species separation in this group, male genitalia and wing interference patterns on a selection of specimens with different colours and/or different measurements were included in the analyses. However, there were no variation in any of these characters, male genitalia were all as in Figs 492–494, and WIPs as in e.g. Fig. 269. Due to the continuous variation of the separating characters used by previous authors the species listed at the end of this group are not included here. However, it is not suggested here that these species represent a single very variable species. There are very probably more than one species among these and in the large material available for this project, but these species are not possible to define morphologically. Because of the uncertainty of the identity of these species no nomenclatural actions including these species are suggested. Such actions have to await future decisions based on studies including other characters than those found in the morphology.

##### Omphale aethiops

Graham

http://species-id.net/wiki/Omphale_aethiops

Figures 12 263 –277 492 521

Omphale aethiops Graham, 1963:263. Holotype female in OUMNH, examined.
Omphale aethiops Graham, Askew (2003).

###### Material.

**Type material.** **Holotype** female, type no. 1298 in OUMNH; 4♀ paratypes (BMNH). **Additional material.** 53♀ 1♂: Denmark 1♀ (LUZM), France 4♀ (RMNH), Germany 3♀ (RMNH), Hungary 1♀ (BMNH), Ireland 1♀ (BMNH), Netherlands 5♀ (RMNH), Slovenia 1♀ (RMNH), Sweden 16♀ (BMNH, LUZM, RMNH), United Kingdom 21♀ 1♂ (BMNH, CH).

###### Diagnosis.

**Figures 263–269.**
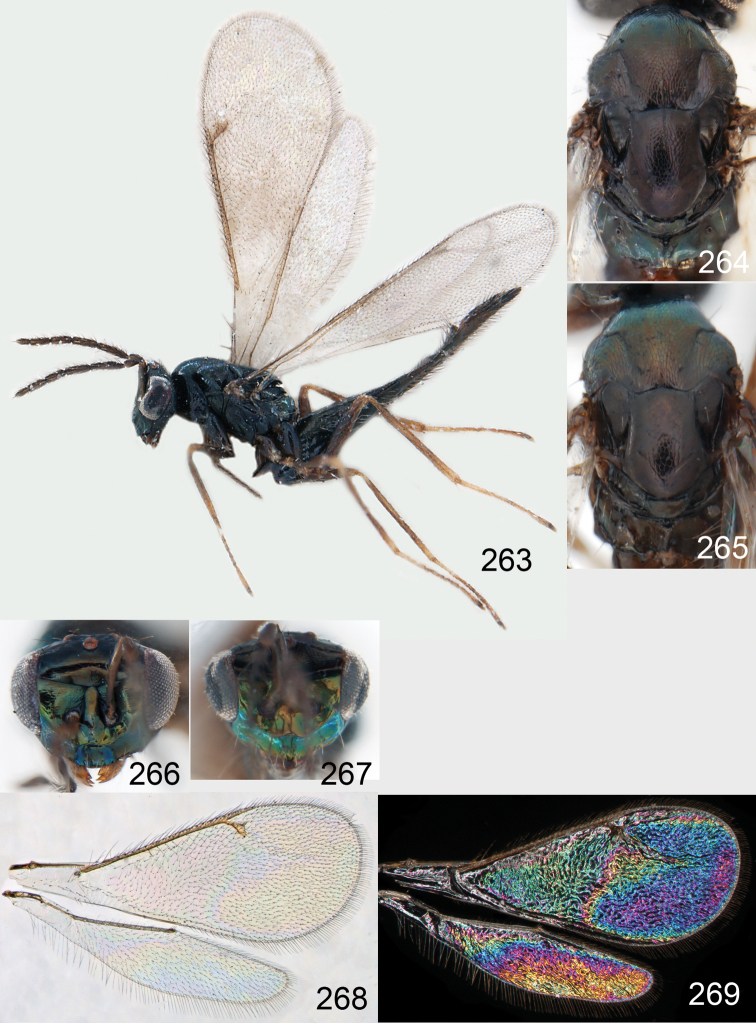
*Omphale aethiops*: **263** habitus in lateral view, female, length of specimen 2.6 mm **264** thoracic dorsum, female **265** thoracic dorsum, male **266** head in frontal view, female **267** head in frontal view, male **268** transparent wings, female **269** wing interference patterns, female.

Female gaster very long (Fig. 263), 2.0–2.2× as long as mesosoma, with 7th tergite 1.5–2× as long as its basal width and with posterior ⅔ thickly setose and with each seta usually on a tubercle (Fig. 277) – see remarks below; legs long and slender (e.g. hind tarsus 0.9× as long as hind tibia and 1.1× as long as hind femur) (Fig. 263), with coxae and femora dark; transepimeral sulcus distinctly curved forwards, angular (Fig. 276); body bronze-black; large species (1.9–3.1 mm).

###### Description.

*Female*. Length of body 1.9-3.1 mm. Antenna with scape yellowish brown to pale brown with dorsal edge dark brown; pedicel and flagellum dark brown; pedicel + flagellum 2.2× as long as distance between eyes; first flagellomere 1.4× as long as second, at base with same width as second flagellomere and gradually narrowing towards apex (Fig. 271); flagellomeres with scattered short setae; clava 1–2-segmented. Face bronze, with engraved reticulation, to smooth; clypeus green metallic (Fig. 266), smooth (Fig. 272), rectangular, 2.5× as wide as high; gena bronze; lower frons bronze and green metallic, with engraved reticulation, subtorular area smooth; interscrobal area with raised reticulation; antennal scrobes join frontal suture separately; frontal suture V-shaped; upper frons and vertex bronze, with engraved reticulation (Fig. 273). Occipital margin rounded (Fig. 273).

**Figures 270–277.**
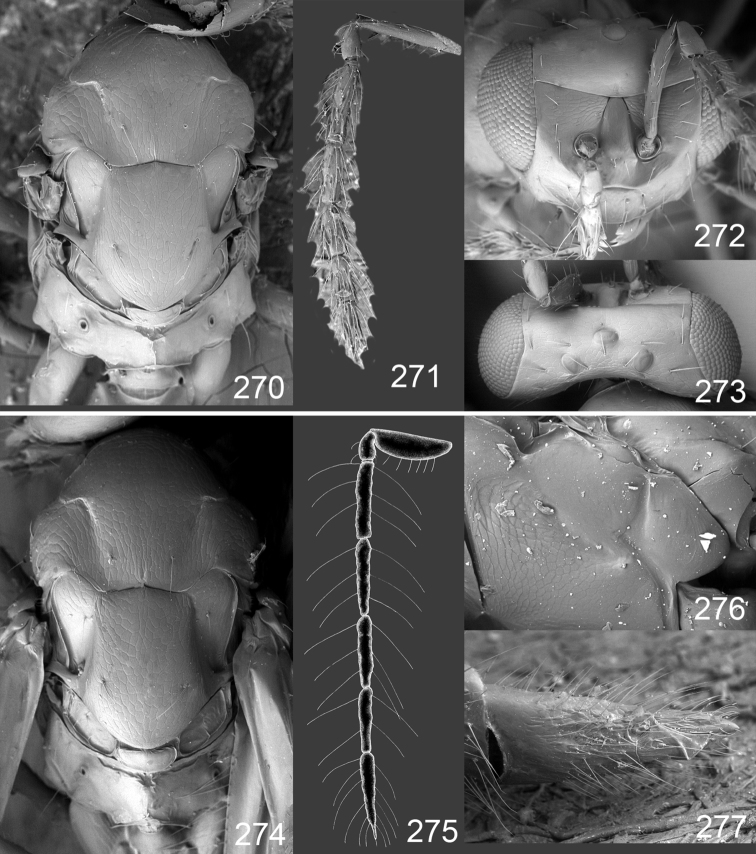
*Omphale aethiops*: **270** thoracic dorsum, female **271** antenna, female **272** head in frontal view, female **273** vertex, female **274** thoracic dorsum, male **275** antenna, male **276** mesosoma in lateral view, female **277** 7^th^ gastral tergite in lateral view, female.

Mesoscutum golden with anterior ½ of midlobe metallic green, to completely bronze (Fig. 264), with engraved reticulation (Fig. 270), midlobe with two pairs of setae; notauli as indistinct impressions. Scutellum bronze (Fig. 264) with engraved reticulation (Fig. 270); 1.2× as long as wide, with anterior margin smoothly and weakly curved forwards. Axillae golden with green tinges (Fig. 264). Dorsellum golden (Fig. 264), smooth and flat (Fig. 270), 0.4× as long as wide, and 0.6× as long as length of median propodeum. Entire lateral mesosoma black with golden and green metallic tinges (Fig. 263); transepimeral sulcus strongly curved forwards. Propodeum golden with green tinges (Fig. 264), smooth with a fovea anteromedially (Fig. 270); propodeal callus with two setae. Coxae and femora dark brown (Fig. 263), tibiae and tarsi yellowish brown; midleg with first tarsomere 0.3× as long as length of tarsus. Forewing infumate, veins yellowish brown and setae dark brown (Fig. 268); speculum closed; admarginal setae 7–12, arising from both marginal vein and wing membrane; radial cell setose; postmarginal vein 1.3× as long as stigmal vein; stigmal vein slender. Hind wing infumate, apex rounded (Fig. 268). Forewing WIP (Fig. 269) with apical ½ magenta with blue margins, and basal ½ yellow/blue (=green), with narrow bands in yellow and magenta separating these two areas.

Petiole dark brown. Gaster black with purple metallic tinges, first tergite with anterior ½ with green metallic tinges; elongate and 2.0–2.2× as long as length of mesosoma; 7^th^ tergite 1.6× as long as length of gaster; 7^th^ tergite with hairless basal ⅓ smooth and apical ⅔ hairy, each hair usually on a raised tubercle (Fig. 277) but in a few specimens hairs not on tubercle – see below under remarks.

*Male*. Length of body 1.9 mm. Features as in female except as follows. Antenna with scape expanded (Fig. 275), distinctly wider than in female, yellowish brown with dorsal edge dark brown; pedicel + flagellum 3.2× as long as distance between eyes; first flagellomere 1.1× as long as second flagellomere; flagellomeres with scattered setae; clava 1-segmented. Face green metallic with golden tinges (Fig. 267), smooth; clypeus green metallic with golden tinges, 1.2× as wide as high; lower frons green metallic with golden tinges, with raised very weak reticulation partly smooth; interscrobal area predominantly smooth with raised very weak reticulation in upper ⅓.

Mesoscutum bronze, anterior ½ of midlobe green metallic (Fig. 265), with engraved reticulation (Fig. 274), midlobe with one pair of setae (posterior pair). Scutellum bronze (Fig. 265); 1.3× as long as wide, with anteromedian part slightly protruding forwards. Axillae bronze (Fig. 265). Dorsellum bronze (Fig. 265), weakly convex (Fig. 274), 0.5× as long as length of median propodeum. Propodeum bronze (Fig. 265). Legs with coxae dark brown metallic. Forewing admarginal setae 9, arising mainly from wing membrane; postmarginal vein 1.2× as long as stigmal vein.

Petiole dark brown, as long as wide with anterior part narrowing off. Gaster black with golden and green metallic tinges, 1.3× as long as length of mesosoma. Phallobase and aedeagus as in Fig. 492.

###### Hosts.

*Dasineura epilobii* (Diptera: Cecidomyiidae) on *Chamaenerion angustifolium* (Gijswijt 1976), collected investigating flowers of *Silene dioica* with cecidomyiid larvae (Askew 2003); two of the female specimens from Sweden (in RMNH) have been reared from *Dasineura traili* (**new record**), a gall midge associated with *Ranunculus*.

###### Distribution.

Denmark (**new record**), France (**new record**), Germany (Gijswijt 1976), Hungary (**new record**), Ireland (**new record**), Netherlands (Gijswijt 1976), Slovenia (**new record**), Sweden (Hansson 1991), United Kingdom (Graham 1963) (Fig. 521).

###### Remarks.

In the material under this species there are several females in which the 7^th^ gastral tergite is smooth and thus without tubercles. No other morphological differences between these specimens and the type of *Omphale aethiops* have been found and provisionally these specimens are here regarded as *Omphale aethiops*.

##### Omphale connectens

Graham

http://species-id.net/wiki/Omphale_connectens

Figures 5, 10 278 –292 493 522

Omphale connectens Graham, 1963:261. Holotype female in OUMNH, examined.

###### Material.

**Type material.** **Holotype** female, type no. 1297 in OUMNH. **Additional material.** 188♀ 11♂: Czech Republic 2♀ (BMNH), Denmark 16♀ (LUZM), France 2♀ (RMNH), Germany 3♀ (RMNH), Hungary 14♀ (BMNH, CH), Netherlands 4♀ (RMNH), Russia 36♀ 4♂ (BMNH), Sweden 80♀ 3♂ (BMNH, CH, NHRS), United Kingdom 31♀ 4♂ (BMNH).

###### Diagnosis.

**Figures 278–284.**
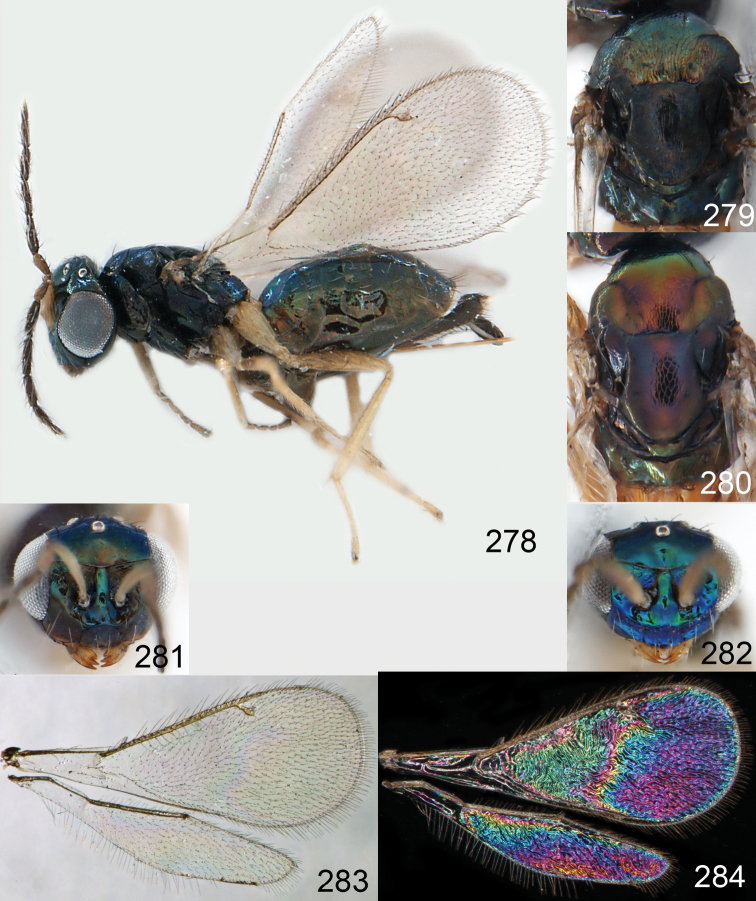
*Omphale connectens*: **278** habitus in lateral view, female, length of specimen 2.1 mm **279** thoracic dorsum, female **280** thoracic dorsum, male **281** head in frontal view, female **282** head in frontal view, male **283** transparent wings, female **284** wing interference patterns, female.

Forewing with row of admarginal setae with all, or most, from ventral marginal vein and with radial cell bare (Fig. 283); face shiny with very weak sculpture and partly smooth, to completely smooth (Fig. 287); forecoxa black or dark brown metallic, mid- and hind coxae yellowish brown (Fig. 278); female flagellomeres 1–3 ventrally with two rows of setae, attached basally and subbasally (Fig. 286); postmarginal vein 1.5–2× as long as stigmal vein.

###### Description.

*Female*. Length of body 1.0–2.4 mm. Antenna with scape yellowish brown with dorsal edge brown; pedicel and flagellum dark brown; pedicel + flagellum 2.4× as long as distance between eyes; first flagellomere 1.1× as long and 1.0× as wide as second (Fig. 286); flagellomeres with scattered short setae, flagellomeres 1–3 ventrally with two sets of setae, one set attached close to base and one attached subapically on the flagellomere; clava 2-segmented. Face purple metallic and golden green, to dark brown with metallic tinges (Fig. 281), with very weak sculpture and partly smooth, to completely smooth (Fig. 287); clypeus green metallic, to dark brown with metallic tinges, smooth, trapezoid to semicircular, 1.4× as wide as high; gena bronze to dark brown metallic; lower frons bronze, with engraved rather strong reticulation, smooth close to eyes, subtorular area smooth; interscrobal area smooth; antennal scrobes join on frontal suture; frontal suture V-shaped; upper frons golden green, smooth. Vertex bronze, with engraved reticulation inside ocellar triangle, smooth outside triangle (Fig. 288). Occipital margin rounded (Fig. 288).

**Figures 285–292.**
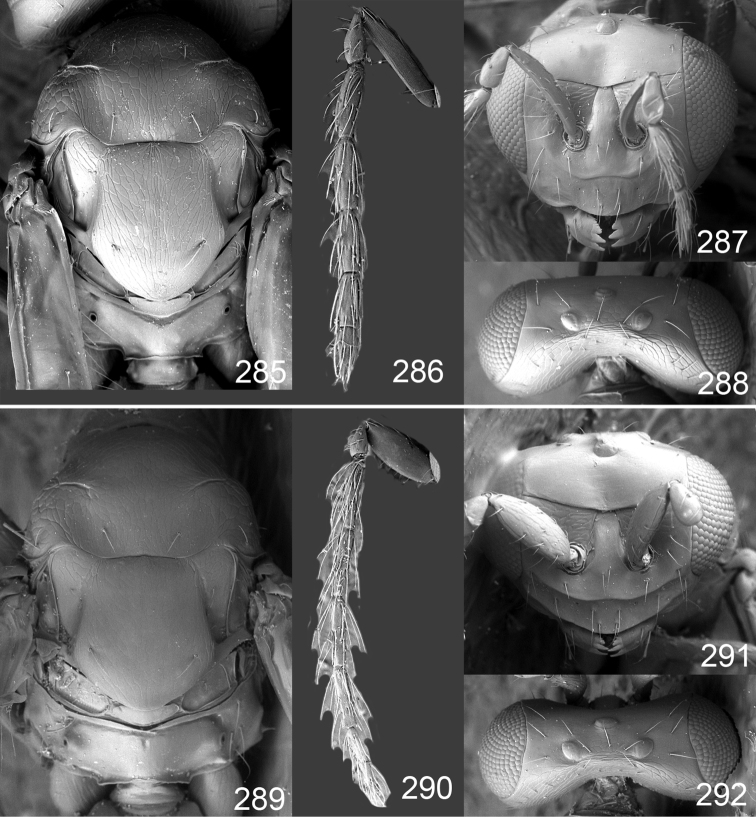
*Omphale connectens*: **285** thoracic dorsum, female **286** antenna, female **287** head in frontal view, female **288** vertex, female **289** thoracic dorsum, male **290** antenna, male **291** head in frontal view, male **292** vertex, male.

Mesoscutum bluish green metallic with posterior ½ of midlobe bronze, to completely bronze, golden green or green metallic (Fig. 279), with engraved reticulation (Fig. 285), midlobe with two pairs of setae; notauli as indistinct impressions. Scutellum bronze (Fig. 279) with engraved reticulation (Fig. 285); 1.2× as long as wide, with anterior margin smoothly and weakly curved forwards. Axillae black metallic (Fig. 279). Dorsellum bronze (Fig. 279), smooth and slightly convex (Fig. 285), 0.3× as long as wide, and 0.6× as long as length of median propodeum. Entire lateral mesosoma bronze (Fig. 278); transepimeral sulcus strongly curved forwards. Propodeum golden with purple tinges (Fig. 279), smooth (Fig. 285); propodeal callus with two setae. Legs with forecoxa dark brown with golden tinges, mid- and hind coxae yellowish brown (Fig. 278); femora pale brown to dark brown; tibiae yellowish brown to pale brown; foretarsus pale brown, mid- and hind tarsi yellowish brown; midleg with first tarsomere 0.3× as long as length of tarsus. Forewing transparent, veins yellowish brown and setae dark brown (Fig. 283); speculum closed; admarginal setae 6–10, arising from marginal vein and from membrane just below vein; radial cell bare; postmarginal vein 1.5–2.0× as long as stigmal vein; stigmal vein slender. Hind wing transparent, apex pointed (Fig. 283). Forewing WIP (Fig. 284) with apical ½ magenta with blue margins and basal ½ yellow/blue (=green), these two areas separated by narrow bands in yellow and magenta.

Petiole yellowish brown. Gaster with first tergite golden green, remaining tergites golden; elongate and 1.6–2.0× as long as length of mesosoma; 7^th^ tergite 0.1× as long as length of gaster.

*Male*. Length of body 1.1–1.4 mm. Features as in female except as follows. Antenna with scape expanded (Fig. 290), distinctly wider than in female, yellowish brown with dorsal and ventral edges dark brown, in some specimens entire inner surface green metallic; pedicel + flagellum 3.5× as long as distance between eyes; first flagellomere 1.1× as long as second flagellomere; flagellomeres with scattered setae; clava 1-segmented. Face bright bluish green metallic (Fig. 282), smooth (Fig. 291); clypeus bright bluish green metallic, trapezoid, 1.5× as wide as high; gena purple metallic; lower frons bright bluish green metallic, with engraved very weak reticulation, smooth close to eyes; interscrobal area bright green metallic; upper frons golden purple or bluish green metallic, with engraved very weak reticulation; vertex purple metallic, with engraved weak reticulation (Fig. 292).

Mesoscutum golden purple, golden red (Fig. 280), golden green or blue metallic, with engraved and weak to very weak reticulation (Fig. 289), midlobe sometimes smooth, midlobe with one pair of setae (posterior pair). Scutellum purple metallic (Fig. 280) or blue metallic, with engraved weak reticulation (Fig. 289); anteromedian margin straight. Axillae purple metallic (Fig. 280). Dorsellum golden (Fig. 280), 0.5× as long as wide. Entire lateral mesosoma black metallic to bronze. Propodeum golden purple or golden green (Fig. 280). Forewing infumate; admarginal setae 6–8, arising from ventral surface of marginal vein.

Petiole yellowish brown, as long as wide with anterior part narrowing off. Gaster black with golden and green metallic tinges, 1.0–1.1× as long as length of mesosoma. Phallobase and aedeagus as in Fig. 493.

###### Host.

Unknown. The record for *Geocrypta galii* in Gijswijt (1976) concerns *Omphale incognita*.

###### Distribution.

Czech Republic (**new record**), Denmark (**new record**), France (Gijswijt 1976), Germany (Gijswijt 1976), Hungary (**new record**), Netherlands (Gijswijt 1976), Russia (**new record**), Sweden (Hansson 1991), United Kingdom (Graham 1963) (Fig. 522).

###### Remarks.

Graham (1963) regarded *Omphale connectens* as similar to species in the *aetius*-group, but placed it outside the group as a single species because of different setation in the forewing and different sculpture on the head between lower margin of eye and mouth opening. However, the male genitalia, which are very distinctive for species-group *aetius*, clearly show that *Omphale connectens* belongs in the *aetius*-group.

##### Omphale dolichura sp. n.

urn:lsid:zoobank.org:act:65A34DA8-D542-4023-86C8-C1310C3D8A5B

http://species-id.net/wiki/Omphale_dolichura

Figures 293 –301 523

###### Material.

**Holotype** female (BMNH), glued to a card, labelled “HUNGARY: Vas Co., Köszeg, 47°22'N, 16°31'E, 26.vi.2010, C. Hansson”.

###### Diagnosis.

**Figures 293–297.**
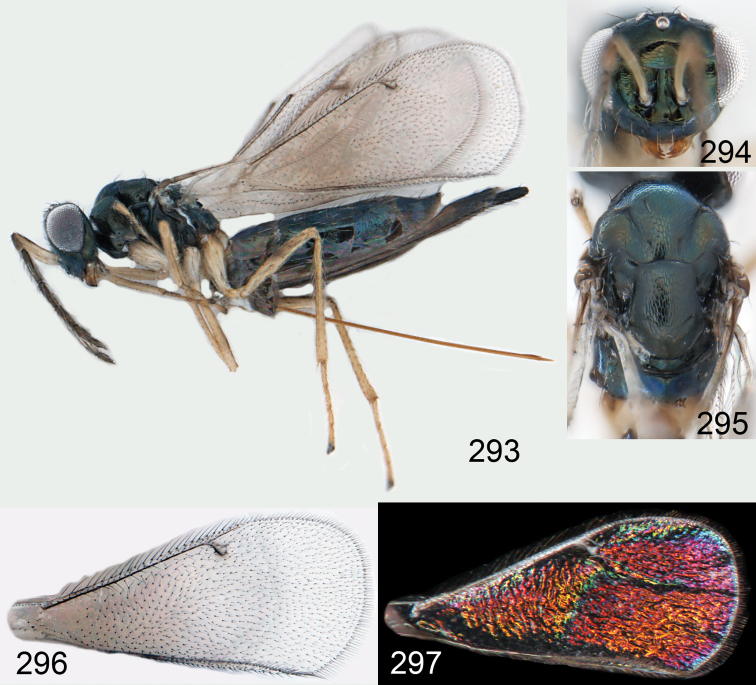
*Omphale dolichura*, female: **293** habitus in lateral view, length of specimen 2.4 mm **294** head in frontal view **295** thoracic dorsum **296** transparent wings **297** wing interference patterns.

Female gaster very long (Fig. 293), 2.5× as long as mesosoma; legs predominantly pale (Fig. 293); face smooth (Fig. 300); forewing admarginal setae 15, arising mainly from ventral surface of marginal vein, and radial cell hairy (Fig. 296).

###### Description.

*Female*. Length of body 2.4 mm. Antenna with scape yellowish brown with dorsal margin dark brown; pedicel and flagellum dark brown; pedicel + flagellum 2.4× as long as distance between eyes; first flagellomere 1.3× as long and 0.9× as wide as second flagellomere (Fig. 299); flagellomeres 2–4 ventrally with two sets of setae, one attached at base and one in apical ⅓ of flagellomere; clava 2-segmented. Face golden green (Fig. 294), smooth (Fig. 300); clypeus bluish green metallic, smooth, trapezoid and 1.5× as wide as high; gena golden with green tinges; lower frons golden green, with engraved weak reticulation; interscrobal area smooth; antennal scrobes join frontal suture separately; frontal suture weakly U-shaped; upper frons golden with weak reticulation; vertex bluish green metallic, with engraved weak reticulation (Fig. 301). Occipital margin rounded (Fig. 301).

**Figures 298–305.**
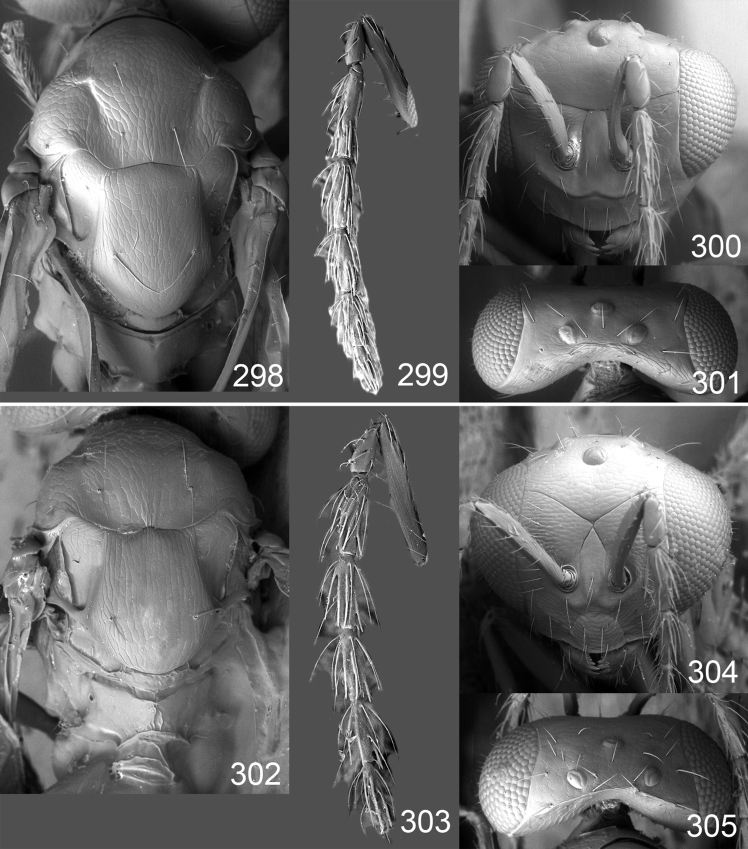
*Omphale* spp., females:**298–301**. *Omphale dolichura*: **298** thoracic dorsum **299** antenna **300** head in frontal view **301** vertex **302–305**. *Omphale brevibuccata*: **302** thoracic dorsum **303** antenna **304** head in frontal view **305** vertex.

Mesoscutum golden green (Fig. 295), with engraved reticulation (Fig. 298), midlobe with two pairs of setae; notauli as indistinct impressions in posterior ½. Scutellum golden with green tinges (Fig. 295), with engraved reticulation (Fig. 298); 1.3× as long as wide, with anterior margin smoothly curved forwards. Axillae golden green (Fig. 295). Dorsellum golden green (Fig. 295), smooth and slightly convex (Fig. 298), 0.3× as long as wide, and 0.4× as long as length of median propodeum. Lateral mesosoma black with golden tinges (Fig. 293); transepimeral sulcus strongly curved forwards. Propodeum black with bluish green tinges (Fig. 295), smooth (Fig. 298); propodeal callus with two setae. Coxae yellowish white with base brown (Fig. 293), femora, tibiae and tarsi yellowish brown, hind femur with dorsal part brown; midleg with first tarsomere 0.4× as long as length of tarsus. Forewing transparent, veins yellowish brown and setae dark brown (Fig. 296); speculum closed; admarginal setae 15, arising mainly from ventral surface of marginal vein; radial cell setose; stigmal vein long and slender; postmarginal vein 1.7× as long as stigmal vein. Hind wing transparent, apex rounded. Forewing WIP (Fig. 297) in magenta with narrow bands in blue and yellow from stigmal vein to hind margin of wing.

Petiole yellowish brown. Gaster with first tergite blue metallic, remaining tergites dark brown with golden tinges, elongate and 2.5× as long as length of mesosoma; 7^th^ tergite 0.2× as long as length of gaster.

*Male*. Unknown.

###### Hosts.

Unknown.

###### Distribution.

Hungary (Fig. 523).

###### Etymology.

From the Greek *dolichos* = long, and *oura* = tail, referring to the very long female gaster.

##### Omphale lugubris

Askew

http://species-id.net/wiki/Omphale_lugubris

Figures 306 –320 494 524

Omphale lugubris Askew, 2003:34. Holotype female in RMNH, examined.
Omphale ?coilus (Walker) var., Graham (1963).
Omphale ?coilus (Walker) var., Gijswijt (1976).

###### Material.

**Type material.** **Holotype** female in RMNH. **Additional material.** 161♀ 1♂: Ireland 2♀ (BMNH), Netherlands 1♀ (RMNH), Russia 123♀ (BMNH, CH, LUZM), Sweden 16♀ 1♂ (BMNH, CH, LUZM), United Kingdom 19♀ (BMNH).

###### Diagnosis.

**Figures 306–312.**
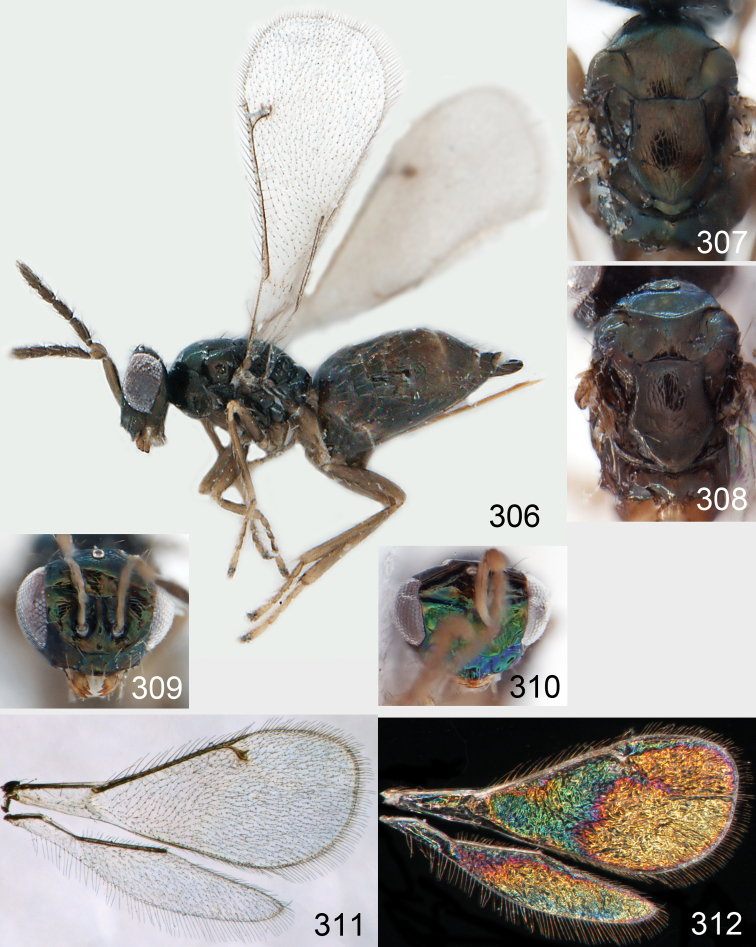
*Omphale lugubris*: **306** habitus in lateral view, female, length of specimen 1.5 mm **307** thoracic dorsum, female **308** thoracic dorsum, male **309** head in frontal view, female **310** head in frontal view, male **311** transparent wings, female **312** wing interference patterns, female.

Female flagellum short - pedicel + flagellum 1.7× as long as distance between eyes - with a solid 2-segmented clava (Fig. 314), flagellomeres 1–3 ventrally with one set of setae attached close to base and reaching beyond apex of flagellomere attached to; small species (1.1–1.6 mm); body black with weak bronze and violet reflections (Fig. 306).

###### Description.

*Female*. Length of body 1.1–1.6 mm. Antenna with scape and pedicel brown, flagellum dark brown; pedicel + flagellum 1.7× as long as distance between eyes; first flagellomere 1.4× as long and 1.0× as wide as second flagellomere (Fig. 314); flagellomeres with scattered short setae, ventral part of flagellomeres 1–3 also with one set of setae attached close to base and reaching beyond apex of flagellomere attached to; clava 2-segmented. Face dark brown with golden tinges (Fig. 309), smooth (Fig. 315); clypeus dark brown with green metallic tinges, smooth, trapezoid, 1.5× as wide as high; gena dark brown with golden tinges; lower frons green metallic with golden tinges, with engraved reticulation, subtorular area smooth; interscrobal area with engraved reticulation; antennal scrobes join on frontal suture; frontal suture V-shaped; upper frons green metallic with golden tinges; vertex dark brown with green metallic tinges, with engraved reticulation (Fig. 316). Occipital margin rounded (Fig. 316).

**Figures 313–320.**
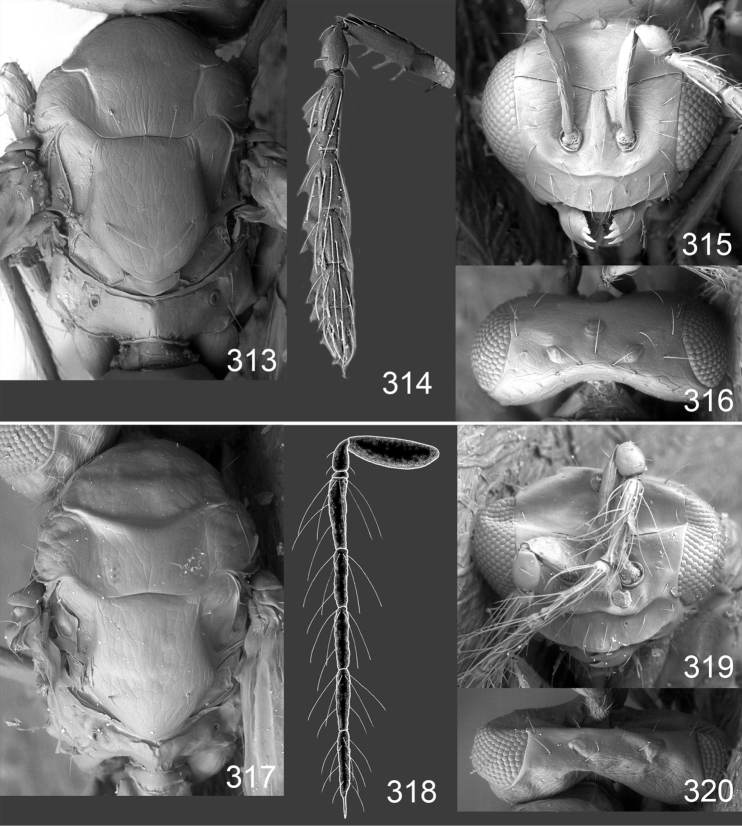
*Omphale lugubris*: **313** thoracic dorsum, female **314** antenna, female **315** head in frontal view, female **316** vertex, female **317** thoracic dorsum, male **318** antenna, male **319** head in frontal view, male **320** vertex, male.

Mesoscutum dark brown with golden and green metallic tinges (Fig. 307), with engraved reticulation (Fig. 313), midlobe with one pair of setae (posterior pair); notauli as indistinct impressions in posterior ½. Scutellum dark brown with golden tinges (Fig. 307), with engraved reticulation (Fig. 313); 1.2× as long as wide, with anterior margin straight. Axillae dark brown with purple metallic tinges (Fig. 307). Dorsellum dark brown with golden tinges (Fig. 307), smooth and slightly convex (Fig. 313), 0.3× as long as wide, and 0.4× as long as length of median propodeum. Entire lateral mesosoma dark brown with metallic tinges (Fig. 306); transepimeral sulcus strongly curved forwards. Propodeum dark brown with golden and green metallic tinges (Fig. 307), smooth (Fig. 313); propodeal callus with two setae. Legs with coxae pale brown with base dark brown, to completely dark brown (Fig. 306), femora dark brown, tibiae and tarsi yellowish brown; midleg with first tarsomere 0.3× as long as length of tarsus. Forewing transparent, veins and setae yellow (Fig. 311); speculum closed; admarginal setae 7, arising both from marginal vein and membrane behind vein; radial cell setose; postmarginal vein 0.8× as long as stigmal vein; stigmal vein slender. Hind wing transparent, apex rounded (Fig. 311). Forewing WIP (Fig. 312) with apical ½ yellow and basal ½ blue/yellow (=green), the two areas separated by narrow bands in magenta and blue.

Petiole yellowish brown. Gaster dark brown with purple and green metallic tinges; elongate and 1.4–1.6× as long as length of mesosoma; 7^th^ tergite 0.1× as long as length of gaster.

*Male*. Length of body 1.3 mm. Features as in female except as follows. Antenna with scape expanded, distinctly wider than in female (Fig. 318), inner surface with apical ½ dark brown with metallic tinges and inner ½ yellowish white, outer surface yellowish white with metallic tinges, dorsal edge dark brown; pedicel + flagellum 3.2× as long as distance between eyes; first flagellomere 1.1× as long as second flagellomere; flagellomeres with scattered setae; clava 1-segmented. Face bright bluish green metallic (Fig. 310), smooth (Fig. 319); clypeus bright bluish green metallic, trapezoid, 1.6× as wide as high; gena dark brown and shiny; lower frons golden green, smooth; interscrobal area golden green; upper frons golden, smooth; vertex dark brown with metallic tinges, with engraved weak reticulation (Fig. 320).

Mesosoma, wings and legs as in female.

Petiole yellowish brown, as long as wide with anterior part narrowing off. Gaster dark brown with metallic tinges, 1.2× as long as length of mesosoma. Phallobase and aedeagus as in Fig. 494.

###### Hosts.

Associated with *Picea* (Gijswijt 1976), but not reared from host.

###### Distribution.

Germany (Askew 2003), Ireland (**new record**), Netherlands (**new record**), Russia (**new record**), Sweden (**new record**), United Kingdom (Askew 2003) (Fig. 524).

####### Species of *aetius*-group not treated here (see above), with remarks on the type specimens for the species

##### Omphale acamas

(Walker)

Entedon Acamas Walker, 1839:83. Lectotype female in BMNH, examined.
Entedon Laelius Walker, 1839:86. Lectotype female in BMNH, examined. Synonymized by Graham (1963).
Omphale acamas (Walker), Graham (1959).
Omphale acamas (Walker), Graham (1963).

###### Remarks.

The lectotype of *Omphale acamas* differs from the lectotype of *Omphale aetius* in having thoracic dorsum golden green, but this colour is variable in the non-type material examined.

##### Omphale aetius

(Walker)

Entedon Aetius Walker, 1839:78. Lectotype female in BMNH, examined.
Entedon Metius Walker, 1839:90. Lectotype female in BMNH, examined. Synonymized by Graham (1963).
Omphale aetius (Walker), Graham (1959).
Omphale aetius (Walker), Graham (1963).
Omphale aetius (Walker), Gijswijt (1976).

###### Remarks.

Lectotype with purple metallic tinges on thoracic dorsum. Type does not run to *Omphale aetius* in the Graham (1963) key because the female gaster is too short on the type, about 2× as long as wide. According to couplet 26 in the Graham key the gaster should be at least 2.4× as long as wide to lead to *Omphale aetius*.

##### Omphale betulicola

Graham

Omphale betulicola Graham, 1963:265. Holotype female in OUMNH, examined.

###### Remarks.

The holotype of *Omphale betulicola* is very similar to the lectotype of *Omphale epaphus*, the main difference appears to be in size, the type of *Omphale betulicola* is 2.0 mm and the type of *Omphale epaphus* is 1.7 mm.

##### Omphale coilus

(Walker)

Entedon Coilus Walker, 1839:79. Lectotype female in BMNH, examined.
Entedon Lyaeus Walker, 1839:84. Lectotype female in BMNH, examined. Synonymized by Graham (1963).
Omphale montana Erdös, 1951:207. Syntypes in HNHM, not examined. Synonymized by Graham (1963).
Omphale coilus (Walker), Graham (1963).
Omphale coilus (Walker), Gijswijt (1976).

###### Remarks.

The lectotype of this species is very similar to the lectotype of *Omphale aetius*, the only difference is that the type of *Omphale coilus* is smaller (1.5 mm) than type of *Omphale aetius* (1.9 mm). In the Graham (1963) key it is stated that *Omphale coilus* has all coxae dark, but in the type of *Omphale coilus* these are predominantly pale.

##### Omphale epaphus

(Walker)

Entedon Epaphus Walker, 1839:89. Lectotype female in BMNH, examined.
Omphale epaphus (Walker), Graham (1959).
Omphale epaphus (Walker), Graham (1963).
Omphale epaphus (Walker), Gijswijt (1976).
Omphale epaphus (Walker), Askew (2003).

###### Remarks.

Lectotype with thoracic dorsum golden green. Similar to *Omphale varipes* and *Omphale betulicola*.

##### Omphale grahami

Gijswijt

Omphale grahami Gijswijt, 1976:81. Holotype female in Amsterdam, examined.
Omphale sp. indet., Graham (1963).
Omphale grahami Gijswijt, Askew (2003).

##### Omphale marica

(Walker)

Entedon Marica Walker, 1839:88. Lectotype female in BMNH, examined.
Omphale marica (Walker), Graham (1963).

###### Remarks.

Graham (1963) found *Omphale marica* difficult to identify and speculated that the type of this species was an aberrant specimen of either *Omphale aetius* or *Omphale phaola*, or perhaps a species that was difficult to diagnose.

##### Omphale phaola

(Walker)

Entedon Phaola Walker, 1839:89. Lectotype female in NMID, not examined, but conspecific paralectotype female in BMNH examined.
Omphale phaola (Walker), Graham (1963).

###### Remarks.

Graham (1963) separated *Omphale phaola* from the other species in the group through the very weak reticulation on anterior part of mesoscutum. However, it is very difficult to appreciate this character given the fact that there is a variation in it among the non-type material, and there is not much difference between the types of the species in the group.

##### Omphale varipes

(Thomson)

Derostenus (Omphale) varipes Thomson, 1878:269. Lectotype female in LUZM, examined.
Omphale varipes (Walker), Graham (1963).

###### Remarks.

The similarity of the type of this species with the type of *Omphale epaphus* was expressed by both Graham (1963) and Askew (2003), something that is also noted here.

#### Species group *clypealis*

**Diagnosis**. Clypeus paler than surrounding parts of frons (Figs 324, 339); females with a 3-segmented antennal clava (Figs 329, 344); male genitalia: phallobase with digital spines very different in size, outer spine much smaller than inner spine (Figs 495, 496). The Nearctic species *Omphale triclava* Hansson also belongs in this group.

##### Omphale clypealis (Thomson)

http://species-id.net/wiki/Omphale_clypealis

Figures 14 321 –335 495 525

Derostenus (Secodes) clypealis Thomson, 1878:270. Lectotype female in LUZM, examined.
Secodes clypealis (Thomson), Dalla Torre (1898).
Omphale clypealis (Thomson), Graham (1963).

###### Material.

**Type material.** Lectotype female, type no. 116:1 in LUZM. **Additional material.** 153♀ 3♂: Denmark 2♀ (LUZM, ZMUC), France 2♀ (RMNH), Germany 1♀ 1♂ (BMNH, RMNH), Hungary 26♀ (BMNH, CH), Netherlands 1♀ (RMNH), Russia 1♀ (BMNH), Spain 2♀ 2♂ (RMNH), Sweden 111♀ (BMNH, CH, LUZM, NHRS, RMNH), United Kingdom 7♀ (BMNH).

###### Diagnosis.

**Figures 321–327.**
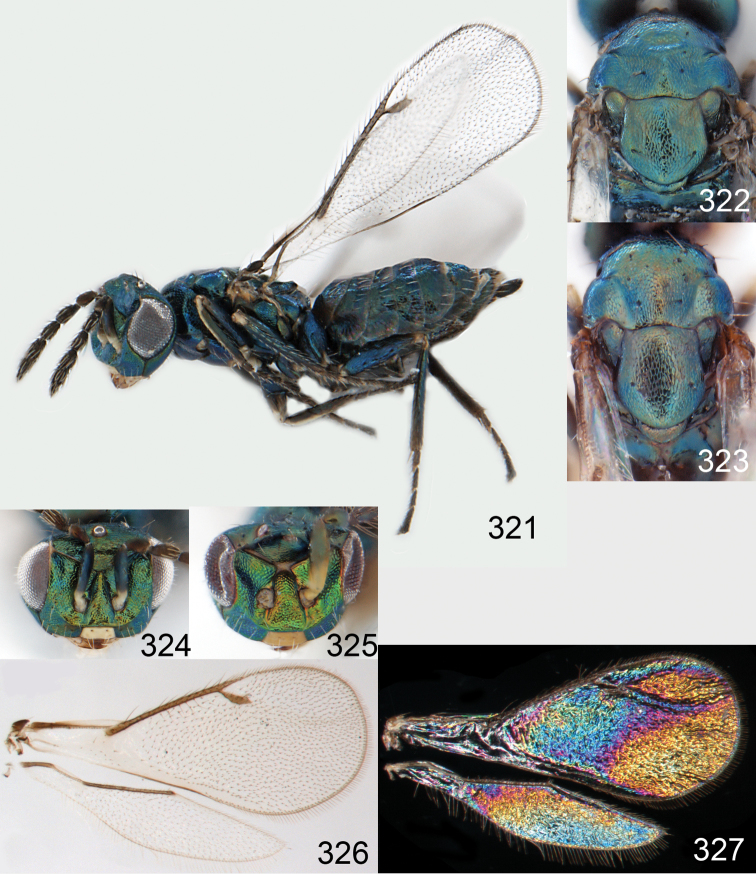
*Omphale clypealis*: **321** habitus in lateral view, female, length of specimen 1.6 mm **322** thoracic dorsum, female **323** thoracic dorsum, male **324** head in frontal view, female **325** head in frontal view, male **326** transparent wings, female **327** wing interference patterns, female.

Clypeus yellowish white (Figs 324, 325); female antennal clava 3-segmented (Fig. 329); forewing speculum open below (Fig. 326), admarginal setae 10–16 arising mainly from membrane, radial cell bare; femora and tibiae metallic, tarsi dark brown (Fig. 321).

###### Description.

*Female*. Length of body 1.3–1.7 mm. Antenna dark brown to black with metallic tinges; pedicel + flagellum 1.5× as long as distance between eyes; first flagellomere 1.2× as long and 1.0× as wide as second flagellomere (Fig. 329); flagellomeres 2–4 ventrally with a single set of setae attach close to base and reaching beyond apex of flagellomere attached to; longitudinal sensilla on flagellomeres distinctly shorter than flagellomere attached to; clava 3-segmented. Face golden green with blue metallic tinges (Fig. 324), with strong reticulation (Fig. 330); clypeus yellowish white, smooth, trapezoid, 2.0× as wide as high; gena bluish green metallic; lower frons bluish green metallic, with raised and strong reticulation; interscrobal area reticulate; antennal scrobes join on frontal suture; frontal suture V-shaped; upper frons bluish green metallic, with raised and weak reticulation; vertex golden green with blue metallic tinges, with raised and weak reticulation (Fig. 331). Occipital margin rounded (Fig. 331).

**Figures 328–335.**
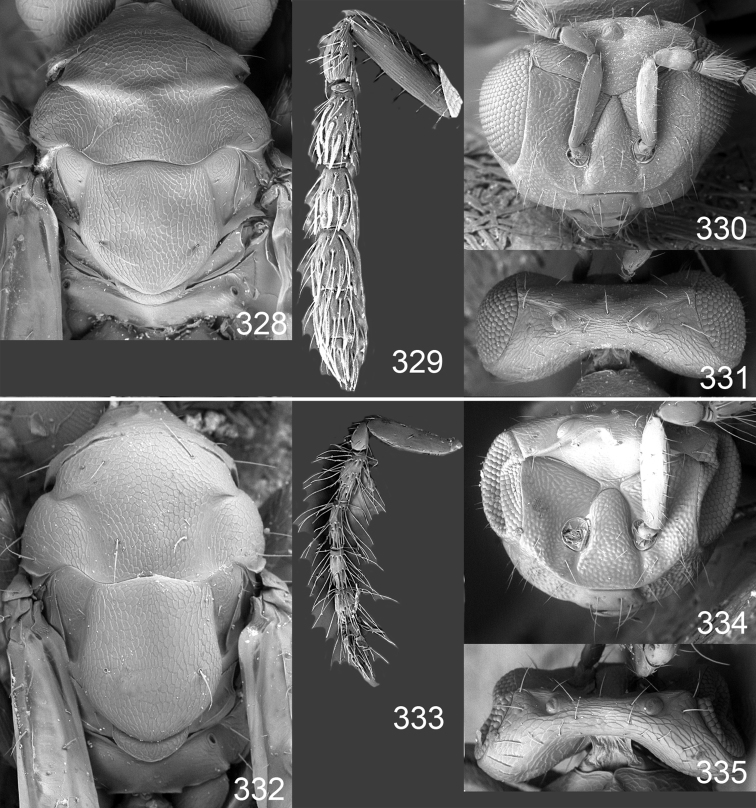
*Omphale clypealis*: **328** thoracic dorsum, female **329** antenna, female **330** head in frontal view, female **331** vertex, female **332** thoracic dorsum, male **333** antenna, male **334** head in frontal view, male **335** vertex, male.

Mesoscutum bluish green metallic with golden tinges (Fig. 322), with raised reticulation (Fig. 328), midlobe with two pairs of setae; notauli as indistinct depressions in posterior ½. Scutellum golden green with blue metallic tinges (Fig. 322), with raised reticulation (Fig. 328), 1.1× as long as wide, with anterior margin smoothly curved forwards. Axillae golden (Fig. 322). Dorsellum golden green (Fig. 322), with weak reticulation (Fig. 328), 0.3× as long as wide, and 0.4× as long as length of median propodeum. Entire lateral mesosoma bluish green metallic (Fig. 321), with or without golden tinges; transepimeral sulcus curved forwards. Propodeum bluish green metallic (Fig. 322), with very weak reticulation, to smooth (Fig. 328); propodeal callus with two setae. Coxae, femora and tibiae bluish green metallic with yellowish white “knees” (Fig. 321); tarsi dark brown; midleg with first tarsomere 0.2× as long as length of tarsus. Forewing transparent, veins and setae dark brown (Fig. 326); speculum open; admarginal setae 10–16, arising mainly from wing membrane; radial cell bare; postmarginal vein 0.9× as long as stigmal vein; stigmal vein elongate. Hind wing transparent, apex rounded (Fig. 326). Forewing WIP (Fig. 327) with apical ½ yellow and basal ½ blue and with a narrow band in magenta between these areas, also with a narrow blue line from stigmal vein towards apical margin of wing.

Petiole black. Gaster bluish green metallic with posterior ½ of tergite 1 and tergites 2+3 golden purplish, smooth, elongate and 1.3–1.4× as long as length of mesosoma; 7^th^ tergite 0.1× as long as length of gaster.

*Male*. Length of body 1.1–1.4 mm. Features as in female except as follows. Antenna with scape with basal ½ yellowish white and apical ½ dark brown; pedicel + flagellum 2.5× as long as distance between eyes; flagellomeres with scattered setae (Fig. 333); clava 1-segmented. Face golden green (Fig. 325), clypeus 2.2× as wide as high; gena golden green; lower frons golden green; antennal scrobes join frontal suture separately (Fig. 334); upper frons golden green, with weak reticulation; vertex golden green.

Mesoscutum golden green with blue metallic tinges (Fig. 323). Scutellum golden green (Fig. 323). Axillae golden green (Fig. 323). Dorsellum bluish green metallic (Fig. 323), 0.6× as long as length of median propodeum. Propodeum golden green (Fig. 323). Forewing hyaline, veins pale brown; admarginal setae 12; postmarginal vein 0.6× as long as stigmal vein.

Petiole black. Gaster golden green with blue metallic tinges, smooth. Phallobase and aedeagus as in Fig. 495.

###### Host.

*Dasineura brassicae* (Diptera: Cecidomyiidae) (e.g. Gijswijt 1976). See above in the introduction for more information on the biology of this species.

###### Distribution.

Czech Republic (Bouček and Askew 1968), Denmark (Bakkendorf 1955), France (**new record**), Germany (Buhl 1960), Hungary (Erdös 1956), Moldova (Bouček 1965), Netherlands (Gijswijt 1976), Poland (Miczulski 1968), Russia (**new record**), Spain (**new record**), Sweden (Thomson 1878), Switzerland (Büchi and Keller 1994), United Kingdom (Graham 1963) (Fig. 525).

##### Omphale parma sp. n.

urn:lsid:zoobank.org:act:07A07593-773F-4A21-9239-7EE5E2722F1D

http://species-id.net/wiki/Omphale_parma

Figures 336 –350 496 526

###### Material.

**Holotype** female (LUZM), glued to a point, labelled “DENMARK: Glatved, 12km S Grenaa, 56°18'N, 10°51'E, 8.vii.1998, R. Danielsson”. **Paratypes**. 39♀ 1♂: DENMARK: 26♀ with same label data as holotype (BMNH, LUZM, ZMUC); 1♀ from same locality as holotype but collected 4.vii.2001 (ZMUC). FRANCE: 1♀ “Montpellier le Vieux, 10.vii.1977, M.J. Gijswijt” (RMNH). GREECE: 4♀ “Loanina, Ipiros, Metsovo, Katara, 1600m, 24.vi.1989, M.J. Gijswijt” (RMNH). HUNGARY: 1♀ 1♂ “ Veszprém Co., Nyirád, 47°00'N, 17°27'E, 213m, 27.vi.2010, C. Hansson” (BMNH). SWEDEN: 6♀ “Bohuslän, Sydkoster, Kilesand, 6.vii.1956, E. Kjellander” (NHRS).

###### Diagnosis.

**Figures 336–342.**
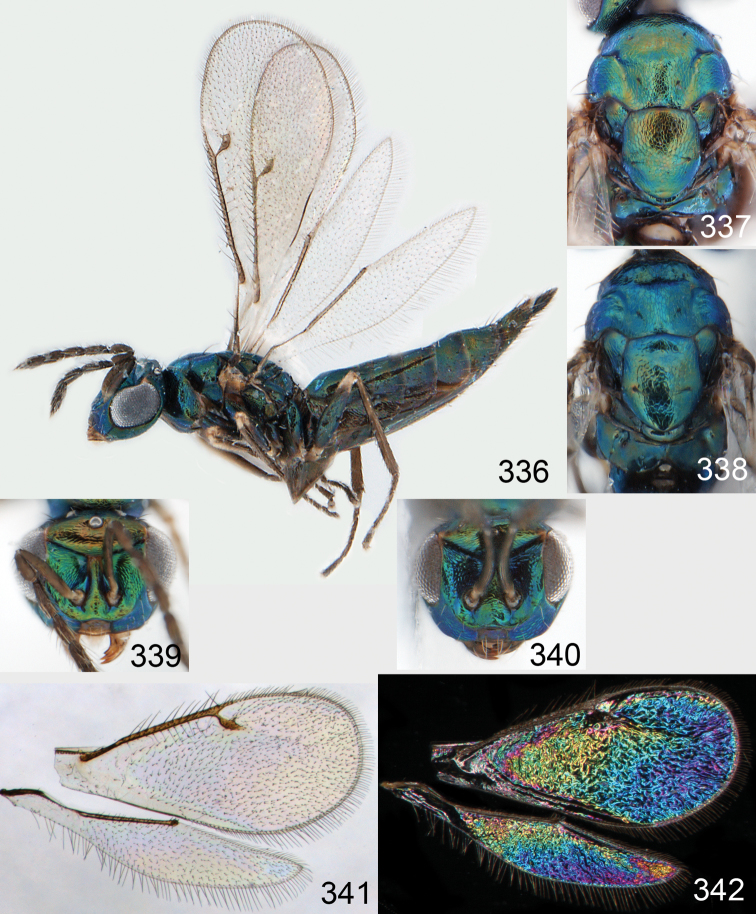
*Omphale parma*: **336** habitus in lateral view, female, length of specimen 2.0 mm **337** thoracic dorsum, female **338** thoracic dorsum, male **339** head in frontal view, female **340** head in frontal view, male **341** transparent wings, female **342** wing interference patterns, female.

**Figures 343–350.**
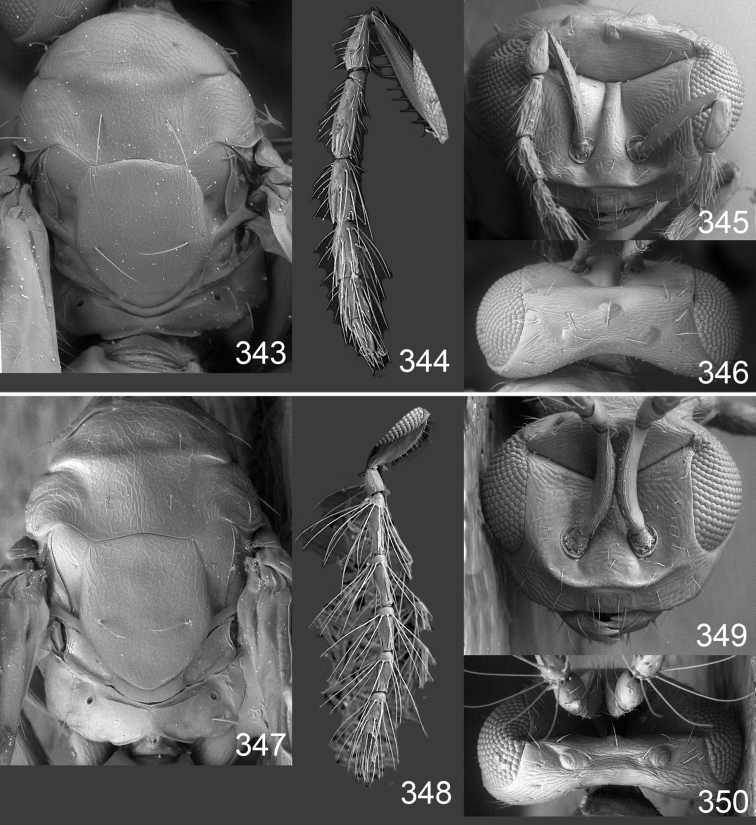
*Omphale parma*: **343** thoracic dorsum, female **344** antenna, female **345** head in frontal view, female **346** vertex, female **347** thoracic dorsum, male **348** antenna, male **349** head in frontal view, male **350** vertex, male.

Clypeus usually pale brown with weak to strong metallic tinges (Figs 339, 340), i.e. paler than face and frons (a few female specimens with clypeus completely metallic); female antennal clava 3-segmented (Fig. 344); male antenna with verticillate setae on flagellomeres 1–4 (Fig. 348); forewing speculum open below (Fig. 341), admarginal setae 6–10 arising mainly from membrane, radial cell bare; femora metallic, tibiae and tarsi dark brown (Fig. 336); petiolar foramen large and median propodeum hence very short (Fig. 343). Similar to *Omphale clypealis* but with clypeus darker, shape of stigmal vein different with base very narrow and rapidly expanding towards apex (Fig. 341), admarginal setae in forewing on average fewer, female gaster longer.

###### Description.

*Female*. Length of body 1.7–2.3 mm. Antenna dark brown with metallic tinges; pedicel + flagellum 1.8× as long as distance between eyes; first flagellomere 1.2× as long and 1.0× as wide as second flagellomere (Fig. 344); flagellomeres 2–4 ventrally with a single set of setae attached close to base and reaching beyond apex of flagellomere attached to; longitudinal sensilla on flagellomeres distinctly shorter than flagellomere attached to; clava 3-segmented. Face bluish green metallic to golden green (Fig. 339), with weak reticulation (Fig. 345); clypeus pale brown with weak to strong metallic tinges, in some specimens (from Greece and Hungary) golden green to bluish green metallic, smooth, trapezoid to semicircular with ventral margin straight, 1.5× as wide as high; gena golden green; lower frons bluish green metallic, with raised and strong reticulation; interscrobal area reticulate; antennal scrobes join frontal suture separately, or join on frontal suture; frontal suture V-shaped; upper frons golden green, with raised and weak reticulation; vertex golden green, inside ocellar triangle with engraved and weak reticulation, outside with raised and weak reticulation and partly smooth (Fig. 346). Occipital margin rounded (Fig. 346).

Mesoscutum bluish green metallic with golden tinges to golden green (Fig. 337), with raised reticulation (Fig. 343), midlobe with two pairs of setae; notauli as indistinct impressions in posterior ½. Scutellum golden green with or without blue metallic tinges (Fig. 337), with raised reticulation (Fig. 343), 1.2× as long as wide, with anterior margin smoothly curved forwards. Axillae bluish green metallic (Fig. 337). Dorsellum golden green (Fig. 337), with weak reticulation (Fig. 343), 0.2× as long as wide, and 0.7× as long as length of median propodeum. Entire lateral mesosoma bluish green metallic to golden green (Fig. 336); transepimeral sulcus curved forwards. Propodeum golden green (Fig. 337), smooth (Fig. 343); propodeal callus with two setae. Coxae and femora bluish green metallic (Fig. 336); tibiae and tarsi dark brown; midleg with first tarsomere 0.3× as long as length of tarsus. Forewing transparent, veins pale brown, setae dark brown (Fig. 341); speculum open; admarginal setae 6–10, arising mainly from wing membrane; radial cell bare; postmarginal vein 0.8× as long as stigmal vein; stigmal vein narrow at base and rapidly expanding towards apex. Hind wing transparent, apex pointed (Fig. 341). Forewing WIP (Fig. 342) with apical ½ magenta with blue margin, basal ½ yellow with narrow bands in magenta and blue towards base.

Petiole dark brown. Gaster bluish green metallic with tergite 7 golden green, smooth, elongate and 1.9–2.3× as long as length of mesosoma; 7^th^ tergite 0.1–0.2× as long as length of gaster.

*Male*. Length of body 1.0 mm. Features as in female except as follows. Antenna with pedicel + flagellum 2.8× as long as distance between eyes; flagellomeres with verticillate setae (Fig. 348); clava 1-segmented. Face bluish green metallic (Fig. 340), strigose-reticulate (Fig. 349); clypeus brown with metallic tinges, with weak sculpture, rectangular, 1.6× as wide as high.

Mesoscutum bluish green metallic (Fig. 338), midlobe with two pairs of setae (Fig. 347) – anterior pair very short. Scutellum bluish green metallic (Fig. 338). Dorsellum bluish green metallic (Fig. 338), 0.5× as long as length of median propodeum. Entire lateral thorax bluish green metallic. Propodeum bluish green metallic (Fig. 338). Forewing admarginal setae 8; postmarginal vein 0.7× as long as stigmal vein.

Petiole black. Gaster bluish green metallic with golden tinges, smooth, 1.1× as long as length of mesosoma. Phallobase and aedeagus as in Fig. 496.

###### Host.

Unknown.

###### Distribution.

Denmark, France, Greece, Hungary, Sweden (Fig. 526).

###### Etymology.

From the Latin *parma* = small shield, referring to the palish clypeus (clypeus = shield).

#### Species group *sulciscuta*

**Diagnosis**. Body strongly sclerotized and does not shrivel when dried. Head with occipital margin with an edge or a carina; clypeus semicircular or rounded triangular, about as wide as high, not transverse; without frontal cross-ridge; flagellomeres with long and asymmetric sensilla basiconica; male flagellomeres with verticillate setae. Forewing with majority of admarginal setae arising from membrane behind marginal vein, and with radial cell hairy. As treated here this may represent an artificial group, recognized mainly through the strongly sclerotized body and the elongate sensilla on the flagellomeres. The definition of the species groups used here rely quite heavily on characters in the male genitalia. Males are known only for two of the species in this group and the phallobase is very different in these species (Figs 497, 498), but the aedeagus is similar (Figs 497, 498) with short penis valves and long apodemes, apodemes 0.6–0.7× the length of aedeagus.

##### Omphale brevibuccata

Szelényi

http://species-id.net/wiki/Omphale_brevibuccata

Figures 302–305 351–355 527

Omphale brevibuccata Szelényi, 1978:222. Holotype female in HNHM, examined.
Omphale brevibuccata Szelényi, Askew (2003).

###### Material.

**Type material.** **Holotype** female, type no. 6017 in HNHM. **Additio-nal material.** 7♀: Bulgaria 2♀ (BMNH), Hungary 1♀ (BMNH), Netherlands 2♀ (RMNH), Russia 1♀ (BMNH), Sweden 1♀ (CH).

###### Diagnosis.

**Figures 351–355.**
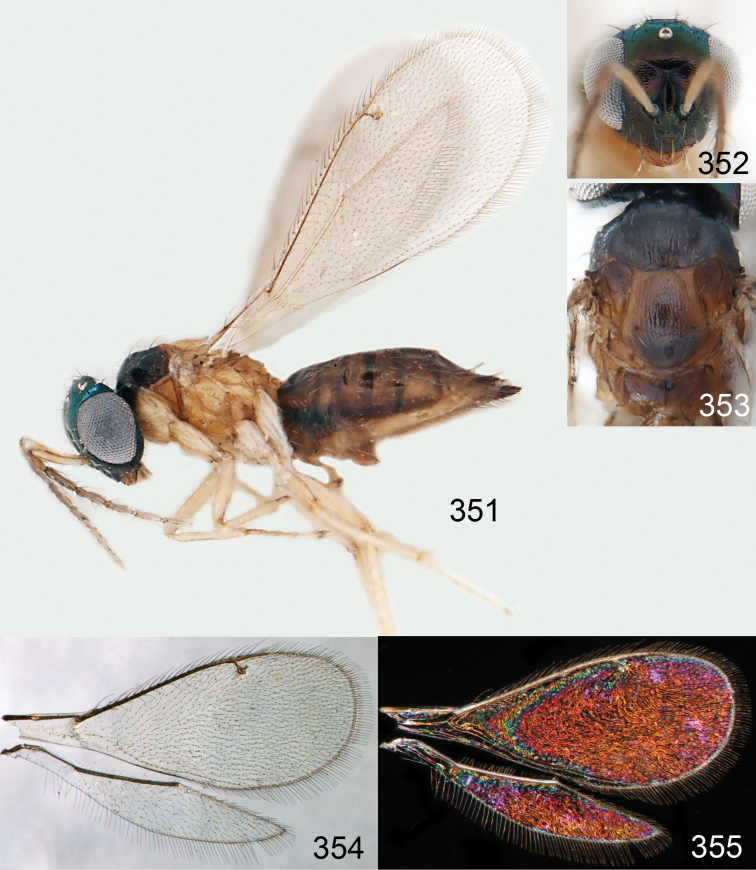
*Omphale brevibuccata*, female: **351** habitus in lateral view, length of specimen 1.7 mm **352** head in frontal view **353** thoracic dorsum **354** transparent wings **355** wing interference patterns.

Vertex green metallic and mesoscutum dark brown (Figs 351–353); mesosomal pleurae yellow (Fig. 351); legs yellowish white to yellow (Fig. 351); frontal cross ridge missing (Fig. 304); antennal scrobes join at or just below frontal suture (Fig. 304); malar space narrow, as narrow as width of scape; first tarsomere on midleg long, 0.4× as long as length of tarsus; flagellum slender and tapering towards apex (Fig. 303), flagellomeres 1–3 ventrally with one set of setae attached close to base and reaching to apex of flagellomere attached to; sensilla on flagellomeres elongate and asymmetric.

###### Description.

*Female*. Length of body 1.3–1.8 mm. Antenna with scape pale yellowish brown; pedicel and flagellum brown; pedicel + flagellum 2.4× as long as distance between eyes; first flagellomere 1.1× as long and 1.1× as wide as second flagellomere (Fig. 303); flagellomeres with scattered setae and flagellomeres 1–3 ventrally with one set of setae attached close to base and reaching to apex of flagellomere attached to; longitudinal sensilla as long as flagellomere attached to; clava 1-segmented. Face dark brown with golden tinges (Fig. 352), strigose (Fig. 304); clypeus dark brown with golden tinges, with engraved reticulation, semicircular, 1.7× as wide as high; gena purple metallic; frontal cross-ridge absent; lower frons golden with green or purple metallic tinges, with engraved reticulation, subtorular area smooth; interscrobal area smooth; antennal scrobes join at or just below frontal suture; frontal suture V-shaped; upper frons golden, sometimes with purple metallic tinges, with engraved reticulation; vertex bright green metallic to golden green, with engraved reticulation (Fig. 305). Occipital margin with an edge (Fig. 305).

Mesoscutum dark brown metallic (Fig. 353), with engraved reticulation (Fig. 302), midlobe with two pairs of setae; notauli as indistinct impressions in posterior ½. Scutellum with anterior ½ yellowish brown and posterior ½ dark brown metallic, to yellow with posteromedian ⅓ dark brown (Fig. 353), with engraved reticulation (Fig. 302); 1.0× as long as wide, with anterior margin almost straight. Axillae brown, yellowish brown to yellow (Fig. 353). Dorsellum yellowish brown to yellow (Fig. 353), smooth and flat (Fig. 302), 0.2× as long as wide, and 0.4× as long as length of median propodeum. Entire lateral mesosoma yellow (Fig. 351); transepimeral sulcus weakly curved forwards. Propodeum pale brown to yellow (Fig. 353), smooth with a median carina and anteromedially with a fovea (Fig. 302); propodeal callus with two setae. Legs yellowish white to yellow (Fig. 351); midleg with first tarsomere 0.4× as long as length of tarsus. Forewing transparent, veins yellowish brown and setae dark brown (Fig. 354); speculum closed; admarginal setae 7-8, arising from marginal vein and from membrane just behind marginal vein; radial cell setose; postmarginal vein 1.8× as long as stigmal vein; stigmal vein slightly enlarged. Hind wing transparent, apex pointed (Fig. 354). Forewing WIP (Fig. 355) predominantly magenta, towards base with thin lines in blue, yellow and magenta.

Petiole yellowish white. Gaster dark brown metallic, elongate and 1.6× as long as length of mesosoma; 7^th^ tergite 0.1× as long as length of gaster.

*Male*. Unknown.

###### Host.

Unknown.

###### Distribution.

Bulgaria (**new record**), Hungary (Szelényi 1978), Netherlands (**new record**), Russia (**new record**), Sweden (**new record**), United Kingdom (Askew 2003) (Fig. 527).

##### Omphale erugata sp. n.

urn:lsid:zoobank.org:act:9371679B-16A6-41DC-8ABE-33A1549EE388

http://species-id.net/wiki/Omphale_erugata

Figures 356 –364 528

###### Material.

**Holotype** female in BMNH, glued to a card, labelled: “ENGLAND: Middlesex, Southgate (1), 12.vii.1968, M.W.R. de V. Graham”.

###### Diagnosis.

**Figures 356–360.**
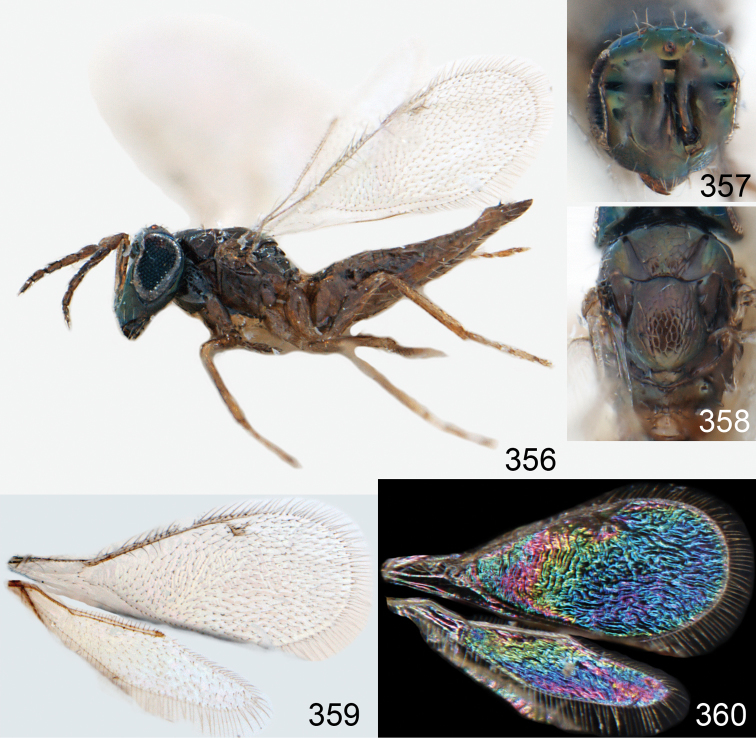
*Omphale erugata*, female: **356** habitus in lateral view, length of specimen 1.4 mm **357** head in frontal view **358** thoracic dorsum **359** transparent wings **360** wing interference patterns.

**Figures 361–368.**
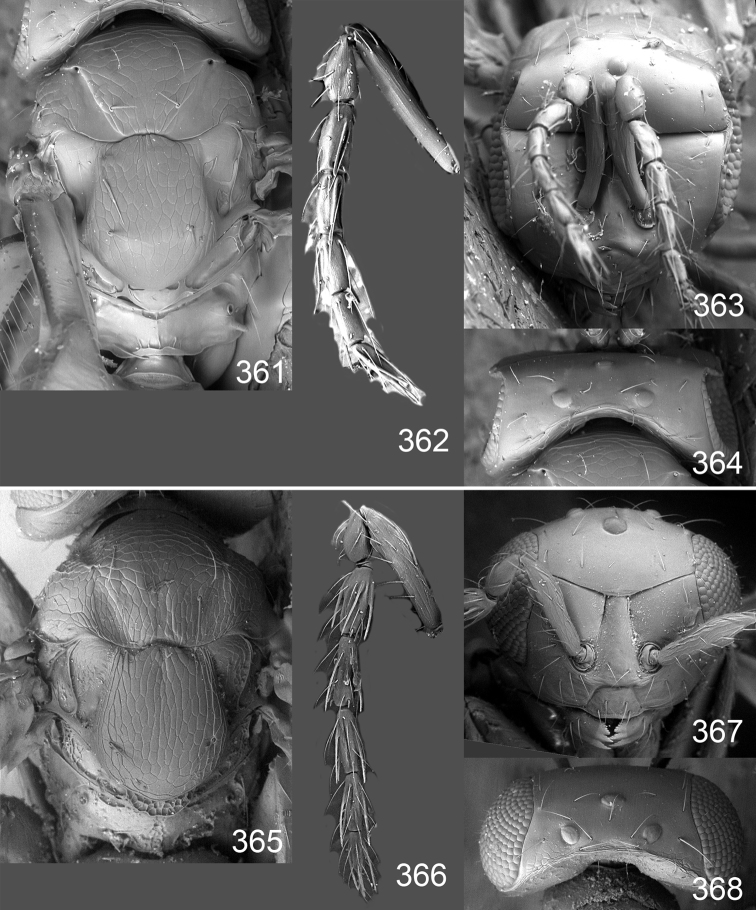
*Omphale* spp., females:**361–364**. *Omphale erugata*: **361** thoracic dorsum **362** antenna **363** head in frontal view **364** vertex **365–368**. *Omphale rossica*: **365** thoracic dorsum **366** antenna **367** head in frontal view **368** vertex.

Frons and vertex smooth (Figs 363, 364); frontal suture very weakly V-shaped, almost straight (Fig. 363); occipital margin with an edge (Fig. 364); antennal scrobes join at frontal suture; notauli as distinct smooth and deep grooves in posterior ⅔, grooves gradually widening towards posterior part (Fig. 361); female gaster elongate (Fig. 356), 1.9× as long as mesosoma; forewing with row of admarginal setae with all, or most, arising from ventral part of marginal vein and with radial cell bare (Fig. 359).

###### Description.

*Female*. Length of body 1.4 mm. Antenna with scape yellowish brown, pedicel and flagellum dark brown; pedicel + flagellum 1.5× as long as distance between eyes; first flagellomere 1.1× as long and 1.2× as wide as second flagellomere (Fig. 362); flagellomeres with scattered short setae, flagellomeres 1–4 ventrally also with a set of long setae attached close to base and reaching beyond apex of flagellomere attached to; longitudinal sensilla on flagellomeres as long as flagellomere attached to; clava 1-segmented. Face golden, strigose; clypeus golden green (Fig. 357), strigose, semicircular (Fig. 363), 1.2× as wide as high; gena dark brown with golden tinges; lower frons golden green, smooth; interscrobal area smooth; antennal scrobes join at frontal suture; frontal suture very weakly V-shaped, almost straight; upper frons and vertex golden, smooth (Fig. 364). Occipital margin with an edge (Fig. 364).

Mesoscutum golden purple (Fig. 358), with engraved and strong reticulation (Fig. 361), midlobe with two pairs of setae; notauli as distinct smooth and deep grooves in posterior ⅔, grooves gradually widening towards posterior part. Scutellum golden purple (Fig. 358), with engraved and strong reticulation (Fig. 361), with anterior margin curved forwards. Axillae golden purple (Fig. 358). Dorsellum golden (Fig. 358), smooth and flat (Fig. 361), 0.5× as long as wide, and 0.9× as long as length of median propodeum. Entire lateral mesosoma dark brown with metallic tinges (Fig. 356). Transepimeral sulcus weakly curved forwards. Propodeum dark brown with metallic tinges (Fig. 358), smooth (Fig. 361); propodeal callus with two setae. Legs with coxae and femora pale brown (Fig. 356); tibiae and tarsi pale brown; midleg with first tarsomere 0.3× as long as length of tarsus. Forewing transparent, veins yellowish brown, setae dark brown (Fig. 359); speculum closed; admarginal setae 7, arising from marginal vein; radial cell bare; postmarginal vein as long as stigmal vein. Hind wing transparent, apex rounded (Fig. 359). Forewing WIP (Fig. 360) with apical ½ blue, basal ½ with bands in yellow, magenta and blue.

Petiole yellowish brown. Gaster with first tergite golden green, remaining tergites dark brown with metallic tinges, smooth, elongate and 1.9× as long as length of mesosoma; 7^th^ tergite 0.1× as long as length of gaster.

*Male*. Unknown.

###### Hosts.

Unknown.

###### Distribution.

United Kingdom (Fig. 528).

###### Etymology.

From the Latin *erugo* = clear of wrinkles, smooth, referring to the smooth and shiny head.

##### Omphale obscura

(Förster) comb.n.

http://species-id.net/wiki/Omphale_obscura

Figures 369 –383 498 529

Elachestus obscurus Förster, 1841:40. Lectotype female in NMW, examined.
Holcopelte obscura (Förster), Förster (1856).
Derostenus obscurus (Förster), Thomson (1878).
Holcopelte fulvipes Förster, 1861. Type not located. Synonymized by Bouček and Askew (1968).
Horismenus obscurus (Förster), Schmiedeknecht (1909).
Holcopelte obscura (Förster), Graham (1959).
Holcopelte obscura (Förster), Bouček (1971).

###### Material.

**Type material.** **Holotype** female of *Elachestus obscurus* in NMW. **Additional material.** 69♀ 2♂: France 1♀ (BMNH), Hungary 5# (BMNH), Russia 7♀ 1♂ (BMNH, CH), Sweden 41♀ (BMNH, CH, LUZM, NHRS), United Kingdom 16♀ 1♂ (BMNH).

###### Diagnosis.

Frons and vertex smooth and shiny (Figs 378, 379, 382, 383); clypeus more or less triangular (Figs 378, 382); occipital margin with a sharp carina (Figs 379, 383); male scape with a sharp dent apicoventrally; midlobe of mesoscutum with a longitudinal groove in posteromedian ⅓ (Figs 376, 380); scutellum in some specimens with a weak median groove in anterior ¼ to complete (Fig. 376); dorsellum concave and sharply margined (Figs 376, 380); propodeum smooth with a wide and shallow groove along anterior margin, with a narrow median carina and laterally with a longitudinal carina close to spiracular sulcus (Fig. 376); petiole 0.7× as long as wide with irregular sculpture, narrows off in anterior part.

###### Description.

*Female*. Length of body 0.9–1.8 mm. Antenna with scape yellowish white with dorsal part yellowish brown; pedicel brown with apex yellowish brown; flagellum brown; pedicel + flagellum 2.1× as long as distance between eyes; first flagellomere 0.9× as long and 1.3× as wide as second flagellomere (Fig. 377); flagellomeres 1–4 with long setae in a basal whorl, with some setae reaching beyond apex of flagellomere attached to, and with short setae apical to whorl; clava 1-segmented. Face dark brown metallic, black metallic, or green metallic (Fig. 372), smooth to strigose (Fig. 378); clypeus with same colour as face, smooth to weakly strigose, triangular, 1.1× as wide (width measured at mouth margin) as high; gena dark brown to black metallic; lower frons with same colour as face, smooth; interscrobal area smooth; antennal scrobes join on or just below frontal suture; frontal suture V-shaped; upper frons dark brown to black metallic with green tinges; vertex dark brown to black metallic with purple tinges, smooth (Fig. 379). Occipital margin with a sharp carina (Fig. 379).

**Figures 369–375.**
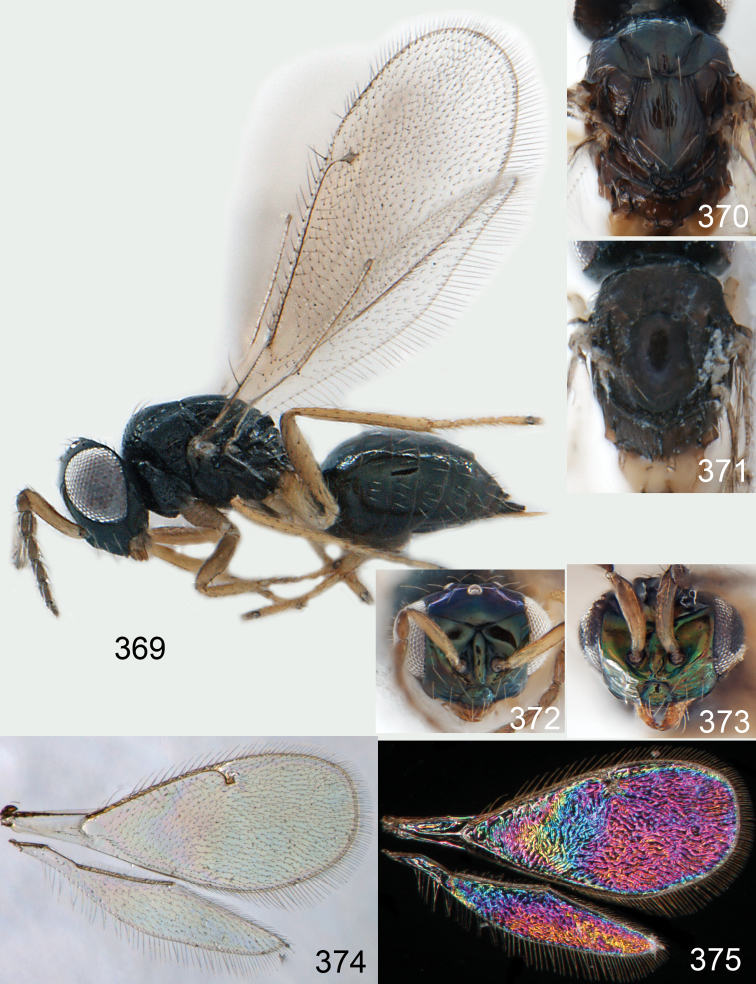
*Omphale obscura*: **369** habitus in lateral view, female, length of specimen 1.4 mm **370** thoracic dorsum, female **371** thoracic dorsum, male **372** head in frontal view, female **373** head in frontal view, male **374** transparent wings, female **375** wing interference patterns, female.

**Figures 376–383.**
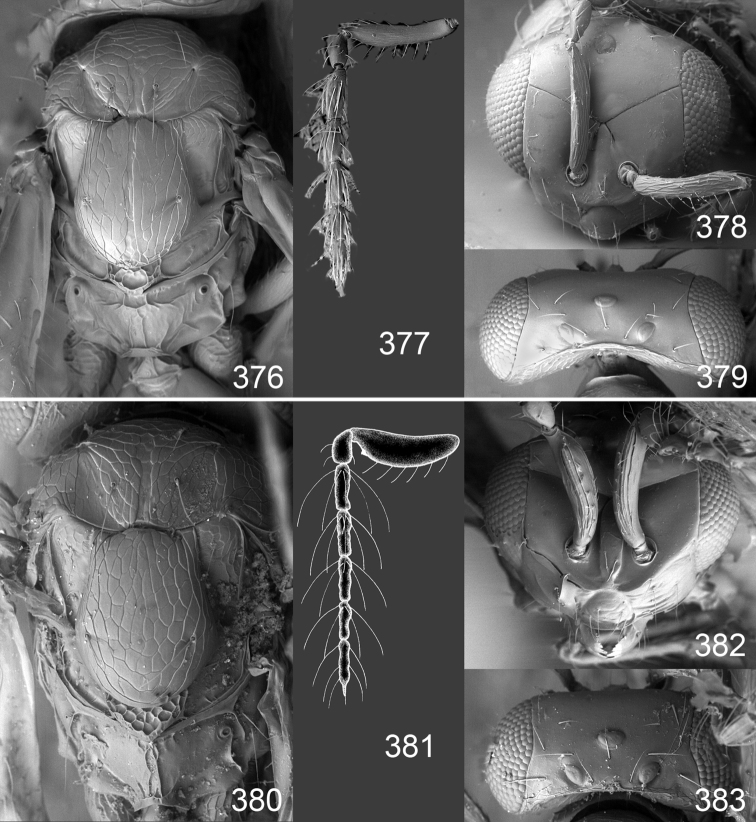
*Omphale obscura*: **376** thoracic dorsum, female **377** antenna, female **378** head in frontal view, female **379** vertex, female **380** thoracic dorsum, male **381** antenna, male **382** head in frontal view, male **383** vertex, male.

Mesoscutum dark brown to black metallic (Fig. 370) with engraved reticulation (Fig. 376), midlobe with two pairs of setae; notauli as indistinct impressions in posterior ½, impressions smooth in some specimens, with a longitudinal groove in posteromedian ⅓. Scutellum dark brown to black metallic (Fig. 370) with engraved reticulation (Fig. 376), in some specimens with a weak median groove in anterior ¼-½; 1.2× as long as wide, with anteromedian margin protruding forwards. Axillae dark brown to black metallic (Fig. 370). Dorsellum dark brown to black metallic (Fig. 370), tongue like (Fig. 376), concave and sharply margined, laterally with longitudinal carinae, 0.4× as long as wide, and 0.3× as long as length of median propodeum. Entire lateral mesosoma dark brown to black metallic (Fig. 369); transepimeral sulcus weakly curved forwards. Propodeum dark brown to black metallic (Fig. 370), smooth or with weak sculpture (Fig. 376), with a wide and shallow groove along anterior margin, with a narrow median carina, laterally with a longitudinal carina close to spiracular sulcus; propodeal callus with two setae. Legs yellowish white to yellowish brown (Fig. 369), forecoxa sometimes pale brown; midleg with first tarsomere 0.3× as long as length of tarsus. Forewing transparent, veins yellow, setae dark brown (Fig. 374); speculum closed; admarginal setae 10–13, arising mainly from wing membrane; radial cell setose; postmarginal vein 0.6× as long as stigmal vein; stigmal vein long and slender; marginal fringe with hairs as long as length of stigmal vein. Hind wing transparent, apex pointed (Fig. 374). Forewing WIP (Fig. 375) with apical ½ magenta, basal ½ with bands in blue, yellow and magenta.

Petiole yellowish brown, dorsal surface 0.7× as long as wide with irregular sculpture. Gaster dark brown to black metallic, smooth, elongate and 1.0–1.4× as long as length of mesosoma; 7^th^ tergite 0.06× as long as length of gaster.

*Male*. Length of body 1.2 mm. Features as in female except as follows. Antenna with scape dark yellowish brown with dorsal edge dark brown; pedicel and flagellum dark brown; pedicel + flagellum 1.9× as long as distance between eyes; flagello-meres 1–4 with verticillate setae and with setae reaching beyond apex of flagellomere attached to (Fig. 381); clava 1-segmented. Face bright metallic green (Fig. 373), smooth with striae close to clypeus (Fig. 382); clypeus bright metallic green, weakly strigose, triangular to almost semicircular, 1.4× as wide as high; gena black metallic; lower frons golden with green tinges; antennal scrobes join on frontal suture; upper frons golden, smooth; vertex golden.

Mesoscutum black metallic with golden tinges (Fig. 371). Scutellum black metallic with golden tinges (Fig. 371). Axillae black metallic with golden tinges (Fig. 371). Dorsellum black metallic (Fig. 371). Entire lateral mesosoma dark brown metallic (Fig. 369). Propodeum black metallic (Fig. 371). Foreleg with coxa dark brown, femur yellowish brown, tibia and tarsus yellow; midleg with coxa pale brown, femur, tibia and tarsus yellow; hind leg with coxa pale brown with base dark brown, femur yellowish brown, tibia and tarsus yellow. Forewing with veins pale brown; admarginal setae 10, arising mainly from wing membrane; postmarginal vein 0.8× as long as stigmal vein.

Petiole dark brown, as long as wide, narrows off in anterior part. Gaster black metallic, smooth, 1.0× as long as length of mesosoma. Phallobase and aedeagus as in Fig. 498.

###### Hosts.

*Dasineura viciae* (Diptera: Cecidomyiidae) (De Stefani 1905); unidentified budgall on *Galium mollugo* (Bouček and Askew 1968).

###### Distribution.

Austria (Kirchner 1867), Czech Republic (Bouček 1957), France (**new record**), Germany (Förster 1841), Hungary (Erdös 1956), Italy (De Stefani 1905), Russia (**new record**), Sweden (Thomson 1878), Switzerland (Förster 1861), United Kingdom (Graham 1959), Yugoslavia (Bouček and Askew 1968).

##### Omphale rossica sp. n.

urn:lsid:zoobank.org:act:CD8F1D00-5CAB-4986-97CB-479C5F857F25

http://species-id.net/wiki/Omphale_rossica

Figures 365–368 384–388 530

###### Material.

**Holotype** female in BMNH, glued to a card, labelled: “RUSSIA: Vaskelovo, Lesnoe, 23.vii.2006, E. Shevtsova”. **Paratype.** 1♀ with same label data as holotype (BMNH).

###### Diagnosis.

**Figures 384–388.**
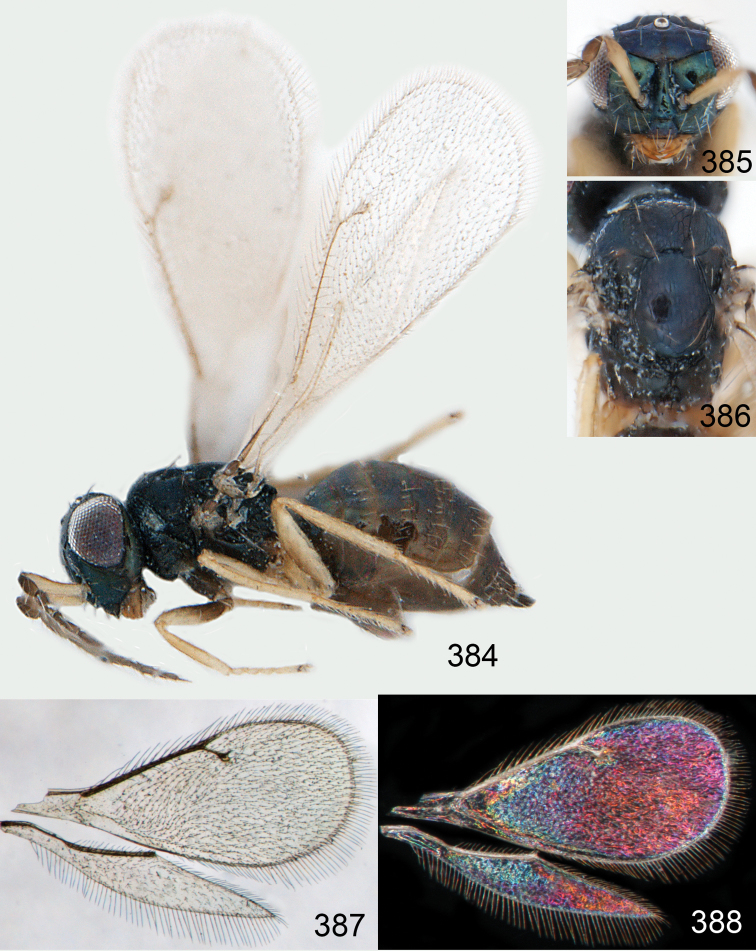
*Omphale rossica*, female: **384** habitus in lateral view, length of specimen 1.2 mm **385** head in frontal view **386** thoracic dorsum **387** transparent wings **388** wing interference patterns.

Frons and vertex smooth and shiny (Figs 367, 368); clypeus trapezoid (Fig. 367); occipital margin with a sharp carina (Fig. 368); midlobe of mesoscutum with a weakly indicated longitudinal groove in posteromedian ⅓ (Fig. 365); dorsellum concave and sharply margined (Fig. 365); propodeum with strong sculpture in median part, otherwise smooth and without longitudinal carinae (Fig. 365); petiole 0.7× as long as wide with irregular sculpture, narrows off in anterior part; forewing speculum small (Fig. 387). Similar to *Omphale obscura* but with antennal scrobes joining frontal suture separately in female (Fig. 367), forecoxa dark brown, mid- and hind coxae yellowish brown (Fig. 384); admarginal setae 9, arising mainly from marginal vein; propodeum without longitudinal carinae.

###### Description.

*Female*. Length of body 1.2 mm. Antenna with scape yellowish brown with apical ¼ dark brown; pedicel and flagellum brown; pedicel + flagellum 2.2× as long as distance between eyes; first flagellomere 1.0× as long and 1.4× as wide as second flagellomere (Fig. 366); flagellomeres 1–4 ventrally with a set of long setae attached subbasally and reaching beyond apex of flagellomere attached to; longitudinal sensilla on flagellomeres setae-like and as long as flagellomere attached to; clava 1-segmented. Face dark brown with strong golden green tinges (Fig. 385), smooth (Fig. 367); clypeus dark brown metallic, smooth, trapezoid, 1.4× as wide (width measured at mouth margin) as high; gena dark brown metallic; lower frons dark brown with strong golden green tinges, smooth; interscrobal area smooth; antennal scrobes join frontal suture separately; frontal suture V-shaped; upper frons dark brown with metallic tinges, smooth; vertex dark brown with metallic tinges, smooth (Fig. 368). Occipital margin with a sharp carina (Fig. 368).

Mesoscutum dark brown metallic (Fig. 386) with engraved reticulation (Fig. 365), midlobe with two pairs of setae; notauli as indistinct impressions in posterior ½, with a weakly indicated longitudinal groove in posteromedian 1/3. Scutellum dark brown metallic (Fig. 386), with engraved reticulation (Fig. 365); 1.2× as long as wide, with anterior margin curved forwards. Axillae dark brown metallic (Fig. 386). Dorsellum dark brown metallic (Fig. 386), tongue like (Fig. 365), concave with very strong reticulation and sharply margined, 0.3× as long as wide, and 0.4× as long as length of median propodeum. Entire lateral mesosoma dark brown metallic (Fig. 384); transepimeral sulcus weakly curved forwards. Propodeum dark brown metallic (Fig. 386), with strong sculpture in median part, otherwise smooth and without longitudinal carinae (Fig. 365); propodeal callus with two setae. Legs yellowish brown (Fig. 384); forecoxa dark brown; midleg with first tarsomere 0.3× as long as length of tarsus. Forewing transparent, veins yellowish brown, setae dark brown (Fig. 387); speculum closed; admarginal setae 9, arising mainly from marginal vein; radial cell setose; postmarginal vein 0.6× as long as stigmal vein, stigmal vein long and slender; marginal fringe with hairs as long as length of stigmal vein. Hind wing transparent, apex pointed (Fig. 387). Forewing WIP (Fig. 388) with apical ⅔ magenta, basal ⅓ blue.

Petiole hidden in type specimens. Gaster dark brown with weak golden green tinges, smooth, elongate and 1.2–1.6× as long as length of mesosoma; 7^th^ tergite 0.07× as long as length of gaster.

*Male*. Unknown.

###### Host.

Unknown.

###### Distribution. 

Russia (Fig. 530).

###### Etymology.

Name referring to country of type locality.

##### Omphale rubigus

(Walker)

http://species-id.net/wiki/Omphale_rubigus

Figures 389 –397 531

Entedon rubigus Walker, 1839:91. Lectotype female in BMNH, examined.
Omphale rubigus (Walker), Graham (1959).
Omphale rubigus (Walker), Graham (1963).

###### Material.

**Type material.** Lectotype female, type no. 5.2036 in BMNH. Additio-nal material. 299♀: France 6♀ (RMNH), Germany 1♀ (RMNH), Hungary 20♀ (BMNH, CH), Italy 1♀ (RMNH), Netherlands 4♀ (RMNH), Russia 61♀ (BMNH, CH, LUZM), Sweden 185♀ (CH, BMNH, LUZM), United Kingdom 21♀ (BMNH).

###### Diagnosis.

Frons above frontal suture and vertex smooth (Figs 396, 397); frontal suture very weakly V-shaped, almost straight (Fig. 396); occipital margin with an edge (Fig. 397); antennal scrobes join at or slightly below frontal suture (Fig. 396); meso-scutum with engraved and strong reticulation and with notauli as distinct smooth deep grooves in posterior ⅔, grooves gradually widening towards posterior part (Fig. 394); forewing with row of admarginal setae with all, or most, arising from ventral part of marginal vein and radial cell bare (Fig. 392).

###### Description.

*Female*. Length of body 1.2–2.0 mm. Antenna with scape pale brown with dorsal margin dark brown, pedicel and flagellum dark brown and shiny; pedicel + flagellum 1.9× as long as distance between eyes; first flagellomere 1.1× as long and 1.3× as wide as second flagellomere (Fig. 395); flagellomeres with scattered short setae, flagellomeres 1–4 ventrally also with a set of long setae attached close to base and reaching beyond apex of flagellomere attached to; longitudinal sensilla on flagellomeres as long as flagellomere attached to; clava 1-segmented. Face dark brown with green metallic tinges (Fig. 390), strigose (Fig. 396); clypeus green to blue metallic, strigose, semicircular, 1.4× as wide as high; gena dark brown metallic; lower frons green metallic, with very weak reticulation, almost smooth; interscrobal area smooth; antennal scrobes join at or slightly below frontal suture; frontal suture very weakly V-shaped, almost straight; upper frons and vertex green to blue metallic, sometimes brightly so, smooth (Fig. 397). Occipital margin with an edge (Fig. 397).

**Figures 389–393.**
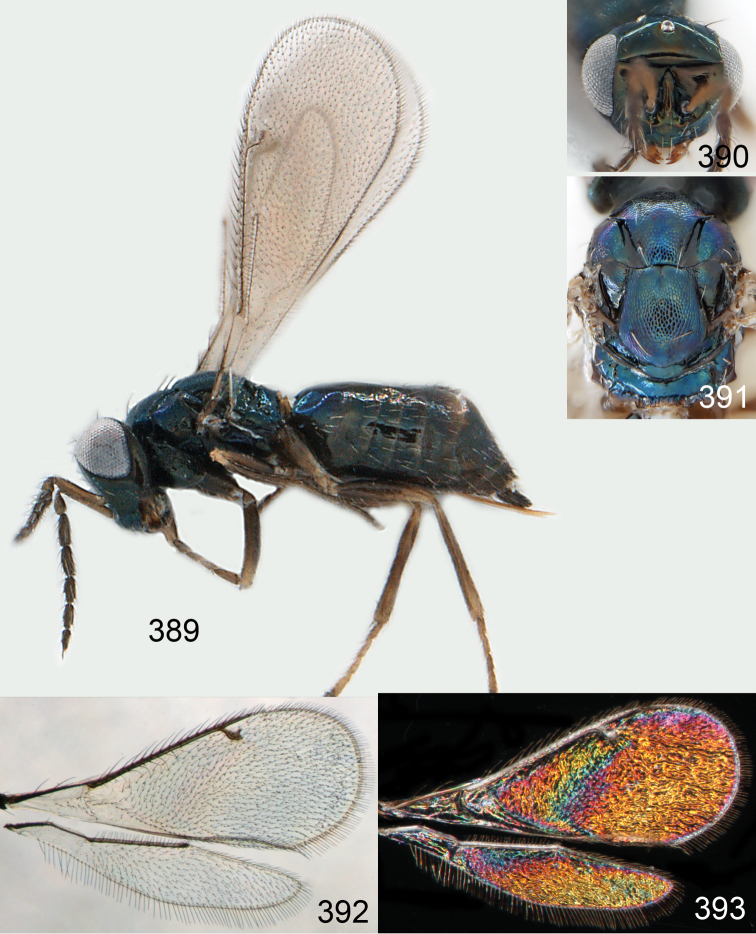
*Omphale rubigus*, female: **389** habitus in lateral view, length of specimen 1.8 mm **390** head in frontal view **391** thoracic dorsum **392** transparent wings **393** wing interference patterns.

**Figures 394–401.**
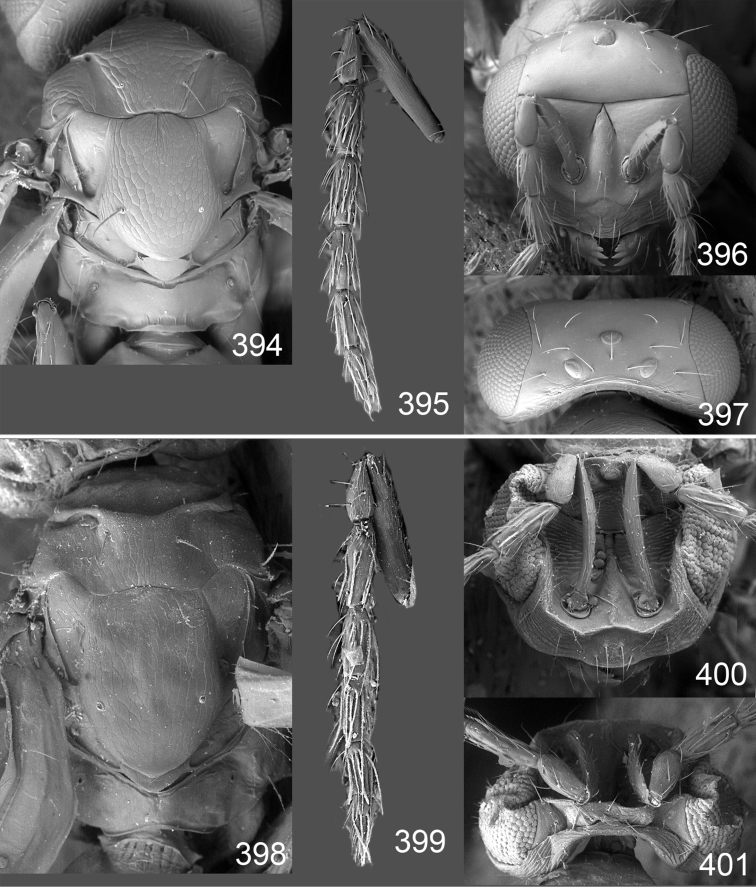
*Omphale* spp., females:**394–397**. *Omphale rubigus*: **394** thoracic dorsum **395** antenna **396** head in frontal view **397** vertex **398–401**. *Omphale ochra*: **398** thoracic dorsum **399** antenna **400** head in frontal view **401** vertex.

Mesoscutum golden with green and blue metallic tinges (Fig. 391), with engraved and strong reticulation (Fig. 394), midlobe with two pairs of setae; notauli as distinct smooth and deep grooves in posterior ⅔, grooves gradually widening towards posterior part. Scutellum golden with green metallic tinges (Fig. 391), with engraved and strong reticulation (Fig. 394), some specimens with a weak median groove in anterior ¼; 1.1× as long as wide, with anterior margin smoothly curved forwards. Axillae golden with green metallic tinges (Fig. 391). Dorsellum green metallic (Fig. 391), smooth and flat (Fig. 394), 0.5× as long as wide, and 0.8× as long as length of median propodeum. Entire lateral mesosoma black metallic (Fig. 389). Transepimeral sulcus weakly curved forwards. Propodeum green metallic (Fig. 391), smooth (Fig. 394); propodeal callus with two setae. Legs with coxae and femora dark brown (Fig. 389); tibiae pale brown to dark brown; foretarsus dark brown, mid- and hind tarsi yellowish brown; midleg with first tarsomere 0.3× as long as length of tarsus. Forewing transparent, occasionally infumate, veins pale brown, setae dark brown (Fig. 392); speculum closed; admarginal setae 7–14, arising from marginal vein or from membrane just behind vein; radial cell bare; postmarginal vein 1.7× as long as stigmal vein. Hind wing transparent, apex rounded (Fig. 392). Forewing WIP (Fig. 393) with apical ½ yellow and margined with magenta, basal ½ with wide bands in magenta, blue and yellow.

Petiole yellowish brown. Gaster with first tergite dark brown with green or blue metallic tinges, remaining tergites dark brown with metallic tinges, smooth, elongate and 1.4–1.6× as long as length of mesosoma; 7^th^ tergite 0.09× as long as length of gaster.

*Male*. Unknown.

###### Hosts.

*Trigonodiplosis* sp. (Diptera: Cecidomyiidae) on *Vicia cracca* (Bouček and Askew 1968). Yefremova et al. (2009) recorded *Omphale rubigus* as a parasitoid on *Phyllonorycter emberizaepennella* and *Phyllonorycter pyrifoliella* (Lepidoptera: Gracillariidae). In view of the other host record for this species, and host records for other species of *Omphale*, which are exclusively gall midges (Diptera: Cecidomyiidae), these records are dubious. Presumably the parasitoid is either misidentified, or the samples have been contaminated.

###### Distribution.

Austria (Bouček and Askew 1968), Czech Republic (Bouček and Askew 1968), France (Gijswijt 1976), Germany (Bouček and Askew 1968), Hungary (**new record**), Italy (**new record**), Netherlands (Gijswijt 1976), Russia (Yefremova 2002), Sweden (Hansson 1991), United Kingdom (Walker 1839) (Fig. 531).

###### Remarks.

Males have never been found in this species, possibly this is a species with thelytokous parthenogenesis.

##### Omphale sulciscuta

(Thomson) comb. n.

http://species-id.net/wiki/Omphale_sulciscuta

Figures 7, 9 402 –416 497 532

Derostenus (Holcopelte) sulciscuta Thomson, 1878:272. Holotype female in LUZM, examined.
Horismenus sulciscutus (Thomson), Schmiedeknecht (1909).
Holcopelte sulciscuta (Thomson), Graham (1959).
Holcopelte sulciscuta (Thomson), Bouček (1971).

###### Material.

**Type material.** Holotype female in LUZM (no type number). **Additional material.** 285♀ 6♂: Czech Republic 1♀ (BMNH), Denmark 1♀ 1♂ (LUZM), France 3♀ (BMNH), Hungary 43♀ 1♂ (BMNH, CH), Russia 12♀ (BMNH, CH, LUZM), Sweden 112♀ 1♂ (BMNH, CH, LUZM, NHRS), United Kingdom 113♀ 3♂ (BMNH).

###### Diagnosis.

Body strongly sclerotized and not collapsing after death (Figs 409, 413); head smooth and shiny (Figs 411, 412, 415, 416), frons black metallic with antennal scrobes joining below frontal suture in female (Figs 405, 411), in male bright metallic bluish green with antennal scrobes joining frontal suture separately (Figs 405, 415); occipital margin as a sharp carina (Fig. 412); notauli in female with posterior ½ as distinct and narrow grooves (Fig. 409); scutellum in anterior ⅔ with a distinct median groove (Figs 409, 413); propodeum with median and lateral longitudinal carinae (Figs 409, 413); petiole quadrangular with anterior part drawn out to a sharp edge that covers the propodeal nucha (Fig. 409); male gaster dark brown with a median white spot across tergites 1–3.

###### Description.

*Female*. Length of body 1.1–1.7 mm. Antenna with scape yellowish brown to pale brown with dorsal margin dark brown; pedicel and flagellum dark brown and shiny; pedicel + flagellum 1.3× as long as distance between eyes; first flagellomere 1.0× as long as second flagellomere, distinctly wider than remaining flagello-meres, 2.0× as wide as second flagellomere (Fig. 410); flagellomeres 1–4 with setae confined to a basal whorl and with setae reaching beyond apex of flagellomere attached to; longitudinal sensilla on flagellomeres as long as flagellomere attached to; clava 1-segmented. Face black metallic with green tinges (Fig. 405), smooth (Fig. 411); cly-peus black metallic with green tinges, smooth, trapezoid to almost semicircular, 1.5× as wide as high; gena black metallic; lower frons black metallic, smooth; interscrobal area smooth; antennal scrobes join below frontal suture; frontal suture V-shaped; upper frons and vertex black metallic, smooth (Fig. 412). Occipital margin with a sharp carina (Fig. 412).

**Figures 402–408.**
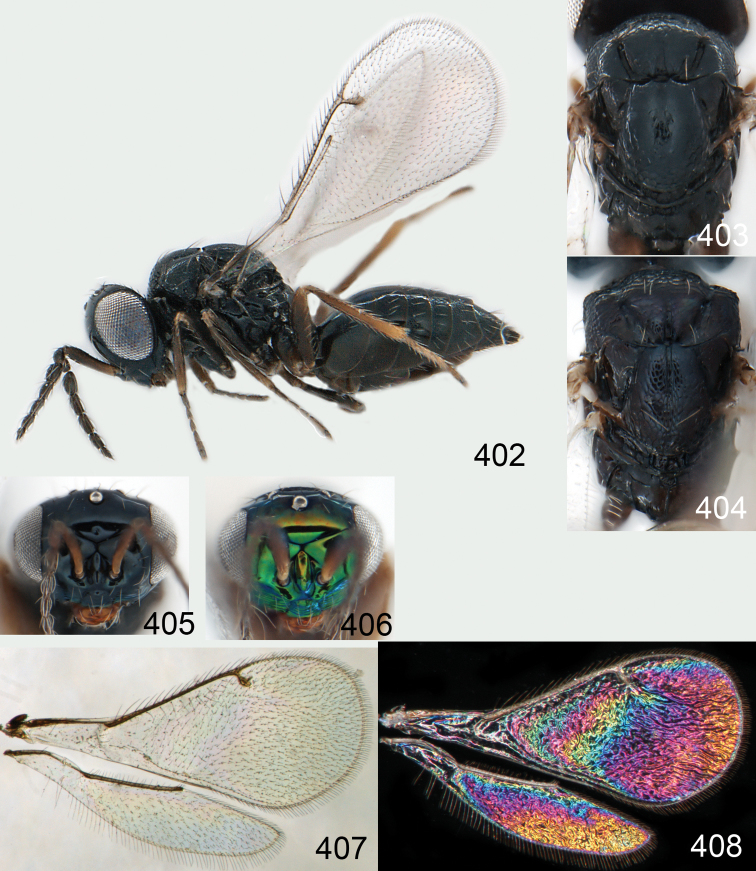
*Omphale sulciscuta*: **402** habitus in lateral view, female, length of specimen 1.6 mm **403**  thoracic dorsum, female **404** thoracic dorsum, male **405** head in frontal view, female **406** head in frontal view, male **407** transparent wings, female **408** wing interference patterns, female.

**Figures 409–416.**
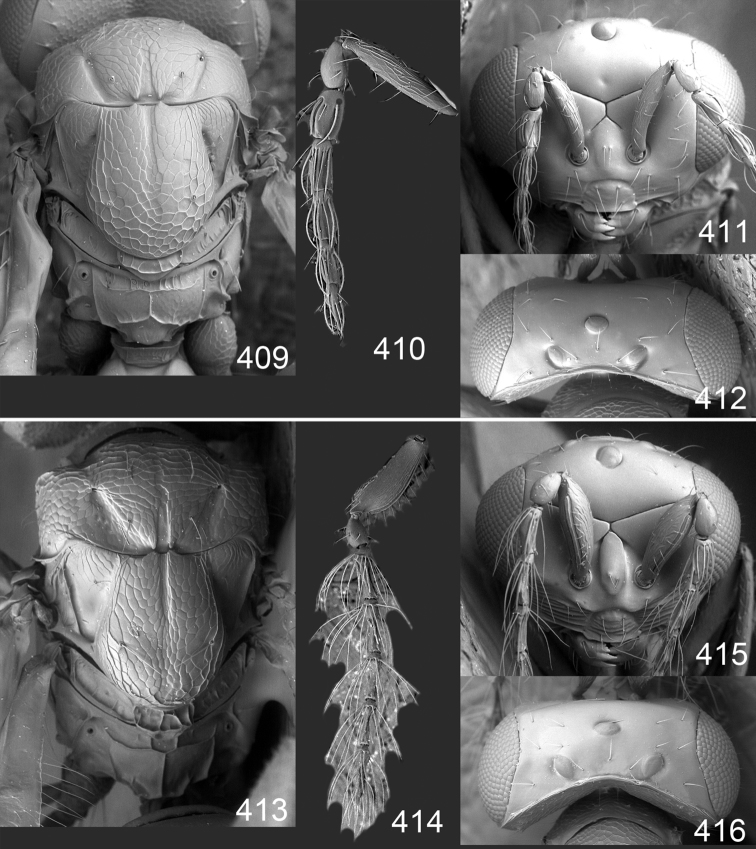
*Omphale sulciscuta*: **409** thoracic dorsum, female **410** antenna, female **411** head in frontal view, female **412** vertex, female **413** thoracic dorsum, male **414** antenna, male **415** head in frontal view, male **416** vertex, male.

Mesoscutum black metallic (Fig. 403) with engraved reticulation (Fig. 409), midlobe with two pairs of setae; notauli as distinct deep grooves in posterior ½. Scutellum black metallic (Fig. 403) with anteromedian ½ with engraved and weak reticulation, remainder with raised and strong reticulation (Fig. 409), with a distinct median groove in anterior ⅔; 1.2× as long as wide, with anterior margin straight. Axillae black metallic (Fig. 403). Dorsellum black metallic (Fig. 403), tongue like (Fig. 409), smooth and with anterior ½ concave and sharply margined, 0.4× as long as wide, and 0.3× as long as length of median propodeum. Entire lateral mesosoma black metallic (Fig. 402); transepimeral sulcus weakly curved forwards. Propodeum black metallic (Fig. 403), smooth with a wide and shallow groove along anterior margin (Fig. 409), with a narrow median carina, laterally with a longitudinal carina half way between median carina and spiracular sulcus, posteromedian part slightly drawn out to form a short nucha that is delimited anteriorly by a transverse carina; propodeal callus with two setae. Foreleg with coxa dark brown (Fig. 402), femur pale brown, tibia yellowish brown to pale brown, tarsus dark brown; midleg with coxa dark brown, femur pale brown to dark brown, tibia and tarsus yellowish brown to pale brown, first tarsomere 0.2× as long as length of tarsus; hind leg with very base of coxa dark brown to black, remaining coxa pale brown to dark brown, in some specimens the entire coxa is black, femur pale brown, tibia yellowish brown to pale brown, tarsus dark brown. Forewing transparent, veins and setae dark brown (Fig. 407); speculum closed; admarginal setae 8–12, arising mainly from wing membrane; radial cell setose; postmarginal vein 0.7× as long as stigmal vein. Hind wing transparent, apex pointed (Fig. 407). Forewing WIP (Fig. 408) with apical ½ magenta with apical margin yellow, basal ½ with wide bands in blue, yellow and magenta.

Petiole black, quadratic and about as long as wide, to transverse, with anterior part drawn out to a sharp margin that covers propodeal nucha. Gaster dark brown to black and metallic, smooth, elongate and 1.3× as long as length of mesosoma; 7^th^ tergite 0.04× as long as length of gaster.

*Male*. Length of body 1.2–1.7 mm. Features as in female except as follows. Antenna with scape dark brown with basal part yellowish white, pedicel and flagellum pale brown; pedicel + flagellum 1.9× as long as distance between eyes; flagellomeres 1–4 with verticillate setae and with setae reaching beyond apex of flagellomere attached to (Fig. 414); clava 1-segmented. Face bright metallic bluish green (Fig. 406), smooth (Fig. 415); clypeus bright metallic bluish green; lower frons bright metallic bluish green; antennal scrobes join frontal suture separately; upper frons bright metallic bluish green; vertex black metallic with blue and green tinges.

Mesoscutum black metallic with purple tinges (Fig. 404), with engraved reticulation (Fig. 413). Scutellum black metallic with purple tinges (Fig. 404), predominantly with engraved reticulation (Fig. 413), posterior and lateral margins with raised and strong reticulation, with a distinct median groove in anterior ⅓; with median part of anterior margin protruding forwards. Axillae black metallic with purple tinges (Fig. 404). Dorsellum black metallic with purple tinges (Fig. 404), with longitudinal carinae (Fig. 413). Propodeum dark brown metallic (Fig. 404). Foreleg with coxa and femur dark brown, tibia and tarsus yellowish brown; midleg with coxa and femur dark brown, tibia and tarsus yellowish brown, first tarsomere 0.3× as long as length of tarsus; hind leg with coxa and femur dark brown, femur tibia pale brown, tarsus yellowish white with 4^th^ tarsomere dark brown, to entirely dark brown. Forewing admarginal setae 10, arising mainly from wing membrane.

Gaster dark brown with a median white spot across tergites 1–3, smooth, 0.9–1.0× as long as length of mesosoma. Phallobase and aedeagus as in Fig. 497.

###### Host.

Unknown.

###### Distribution.

Armenian SSR (Bouček and Askew 1968), Czech Republic (Bouček 1957), Denmark (**new record**), France (**new record**), Germany (Bouček and Askew 1968), Hungary (Erdös 1956), Moldova (Bouček 1965), Russia (**new record**), Sweden (Thomson 1878); United Kingdom (Graham 1959) (Fig. 532).

###### Remarks.

The male is described here for the first time. Similar to several other *Omphale* species the sex ratio in the material at hand is skewed with considerably more females than males.

####### Unplaced species

##### Omphale aceris

(Erdös)

http://species-id.net/wiki/Omphale_aceris

Figures 417–422 533

Secodes aceris Erdös, 1951:208. Lectotype female in HNHM, examined.
Omphale aceris (Erdös), Graham (1963).

###### Material.

**Type material.** Lectotype female, type no. 6063 in HNHM.

###### Diagnosis.

Gaster short, 0.8× as long as length of mesosoma; forewing speculum open below, with veins and setae yellow; antennal flagellum with basal 2 flagellomeres pale brown and apical 3 flagellomeres yellow (Fig. 422); legs white; row of admarginal setae mainly arising from marginal vein; radial cell bare.

###### Description.

*Female*. Length of body 1.4 mm. Antenna with scape dark brown with basal part white; pedicel pale brown; flagellum with flagellomeres 1–2 pale brown, flagellomeres 3–5 yellow (Fig. 422); pedicel + flagellum 1.5× as long as distance between eyes; first flagellomere 1.2× as long as and about 1× as wide as second flagellomere; flagellomeres with scattered short setae; clava 2-segmented. Face golden red, with raised reticulation; clypeus golden red, smooth, trapezoid, 1.6× as wide as high; gena purple metallic; lower frons purple metallic (Fig. 420), with raised reticulation (Fig. 419), subtorular area smooth; interscrobal area with raised reticulation; antennal scrobes join on frontal suture; frontal suture V-shaped; upper frons and vertex purple metallic, with raised reticulation. Occipital margin rounded (Fig. 421).

**Figures 417–422.**
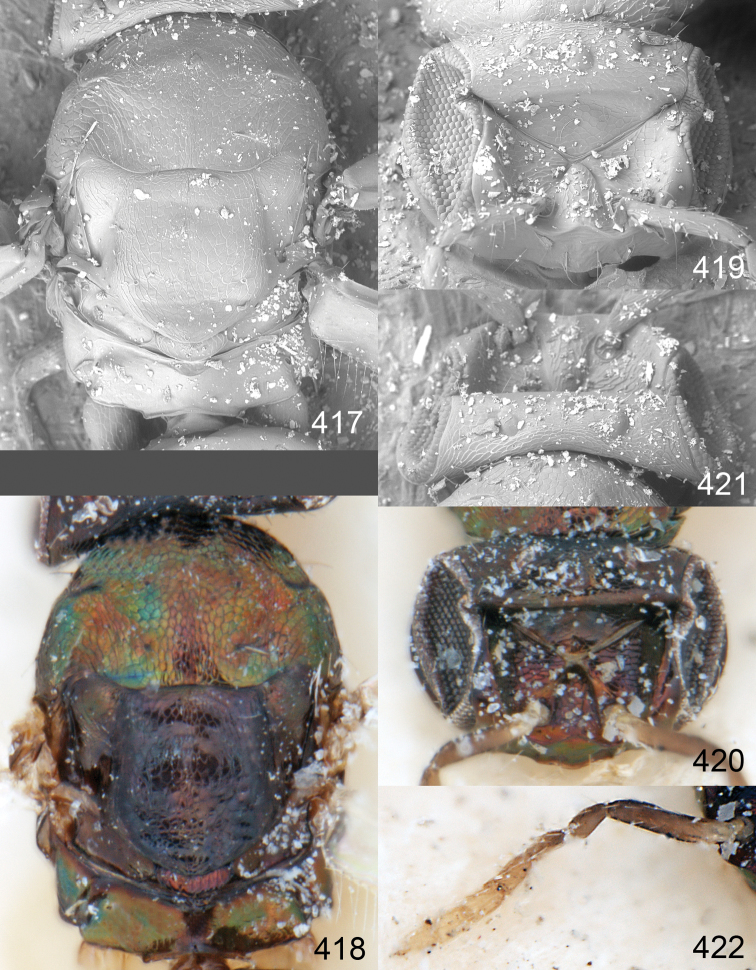
*Omphale aceris*, female lectotype: **417–418** thoracic dorsum **419–420** head frontal **421** vertex **422** antenna.

Mesoscutum golden with green metallic spots (Fig. 418), with engraved reticulation (Fig. 417), midlobe with two pairs of setae; notauli as indistinct impressions. Scutellum black with purple metallic tinges (Fig. 418), with engraved reticulation (Fig. 417); 1.0× as long as wide, with anterior margin straight. Axillae golden with purple tinges (Fig. 418). Dorsellum purple metallic (Fig. 418), with very weak sculpture and flat (Fig. 417), 0.4× as long as wide, and 0.4× as long as length of median propodeum. Entire lateral mesosoma purple metallic, except yellow acropleuron; transepimeral sulcus weakly curved forwards. Propodeum golden with red tinges (Fig. 418), smooth (Fig. 417); propodeal callus with two setae. Legs white; midleg with first tarsomere 0.3× as long as length of tarsus. Forewing transparent, veins and setae yellow; speculum open; radial cell bare; postmarginal vein about as long as stigmal vein; stigmal vein slender. Hind wing transparent, apex rounded. WIP not possible to see on single examined specimen because the wings are glues to the card.

Petiole dark brown. Gaster purple metallic, first tergite with golden tinges, short and 0.8× as long as length of mesosoma; 7^th^ tergite 0.06× as long as length of gaster.

*Male*. Unknown.

###### Hosts.

Unknown.

###### Distribution.

Hungary (Erdös 1951) (Fig. 533).

###### Remarks. 

Through the pale wing veins, pale legs and part of the antenna this is an easily recognizeable species. However, based on the single shriveled female specimen examined we find it difficult to place it in a group. Possibly it belongs in the *phruron*-group but to establish that fresh specimens, preferably both sexes, must be examined.

##### Omphale erginnus

(Walker)

http://species-id.net/wiki/Omphale_erginnus

Figures 423 –437 499 534

Entedon erginnus Walker, 1839:124. Lectotype male in BMNH, examined.
Omphale erginnus (Walker), Graham (1959).
Omphale erginnus (Walker), Graham (1963).
Omphale erginnus (Walker), Hansson (1996b).
Omphale erginnus (Walker), Hansson (1997).
Omphale erginnus (Walker), Hansson (2004).

###### Material.

**Type material.** Lectotype male, type no. 5.2038 in BMNH. **Additional material.** 9♀ 5♂: Netherlands 1♀ (RMNH), Sweden 5♀ 2♂ (LUZM, NHRS), Uni-ted Kingdom 3♀ 3♂ (BMNH).

###### Diagnosis.

Female flagellum with 1-segmented clava (Fig. 431); male scape with a sharp dent ventroapically (Fig. 435); clypeus poorly delimited with (short) grooves laterally only (Figs 432, 436); head without frontal cross-ridge (Figs 432, 436); setae on vertex and thoracic dorsum long (e.g. vertexal seta situated in middle of ocellar triangle as long as distance between posterior ocelli (Figs 433, 437)); transepimeral sulcus straight; male gaster dark brown with a median white spot across tergites 1–3.

###### Description.

*Female*. Length of body 1.4–1.9 mm. Antenna with scape yellowish brown with dorsal margin dark brown; pedicel and flagellum dark brown and shiny; pedicel + flagellum 2.2× as long as distance between eyes; first flagellomere 1.0× as long and 1.0× as wide as second flagellomere (Fig. 431); flagellomeres 1–4 with scattered short setae; longitudinal sensilla on flagellomeres as long as flagellomere attached to; clava 1-segmented. Face purple or green metallic (Fig. 426), smooth (Fig. 432); clypeus purple or green metallic, smooth, poorly delimited with grooves laterally only; gena purple or green metallic; frontal cross-ridge absent; lower frons purple or green metallic, with raised reticulation, smooth close to frontal suture; interscrobal area smooth; antennal scrobes join frontal suture separately; frontal suture V-shaped; upper frons purple or green metallic with very weak reticulation, shiny; vertex golden with green tinges or green metallic, smooth or with weak reticulation (Fig. 433). Occipital margin with a sharp carina (Fig. 433).

**Figures 423–429.**
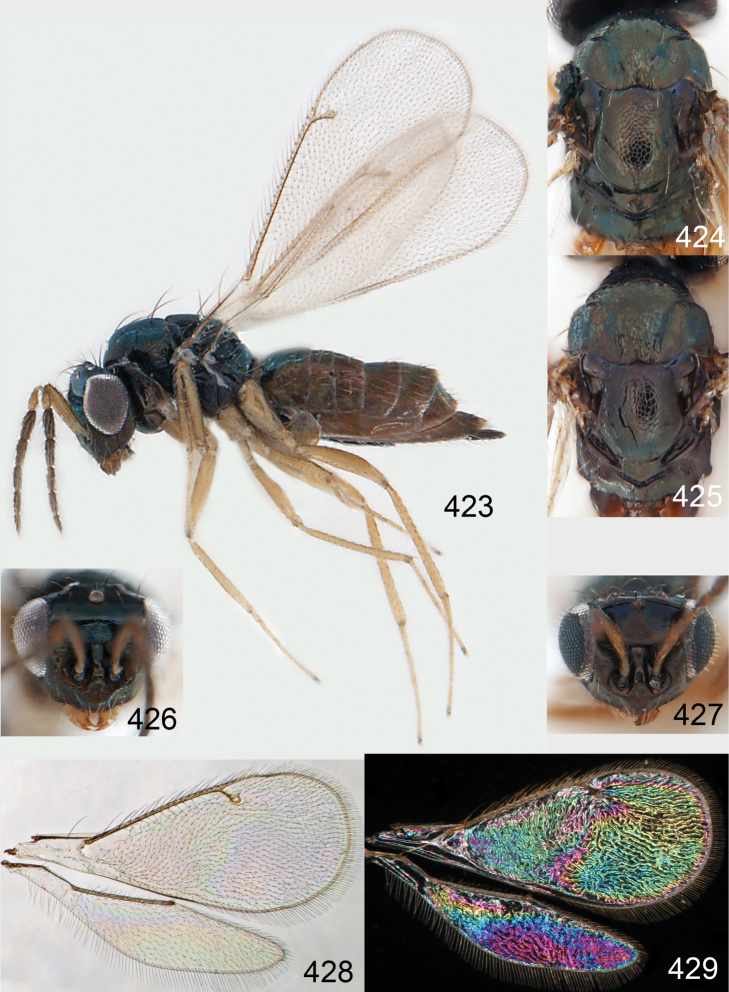
*Omphale erginnus*: **423** habitus in lateral view, female, length of specimen 1.8 mm **424** thoracic dorsum, female **425** thoracic dorsum, male **426** head in frontal view, female **427** head in frontal view, male **428** transparent wings, female **429** wing interference patterns, female.

**Figures 430–437.**
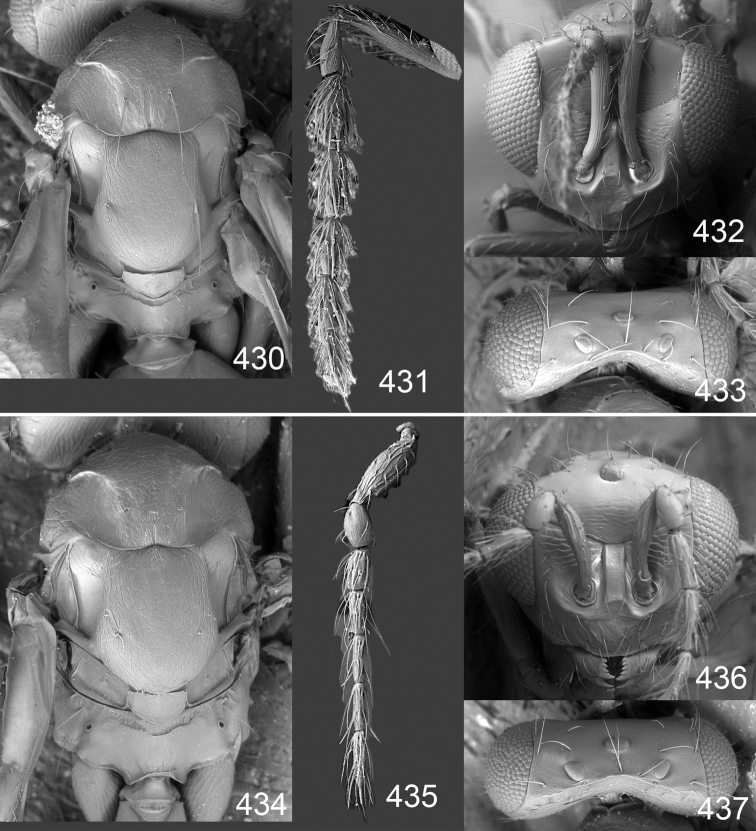
*Omphale erginnus*: **430** thoracic dorsum, female **431** antenna, female **432** head in frontal view, female **433** vertex, female **434** thoracic dorsum, male **435** antenna, male **436** head in frontal view, male **437** vertex, male.

Mesoscutum golden with green tinges (Fig. 424), with engraved reticulation (Fig. 430), midlobe with two pairs of setae; notauli as indistinct impressions in posterior ½. Scutellum golden (Fig. 424), with engraved reticulation (Fig. 430); 1.2× as long as wide, with anterior margin straight. Axillae purple metallic (Fig. 424). Dorsellum purple metallic to golden (Fig. 424), smooth and convex (Fig. 430), with or without a weak median carina, 0.5× as long as wide, and 0.7× as long as length of median propodeum. Lateral pronotum golden (Fig. 423); propleuron dark brown with metallic tinges; prepectus golden; acropleuron dark brown and mes-episternum dark brown with metallic tinges; upper mesepimeron dark brown with metallic tinges; lower mesepimeron dark brown with metallic tinges; transepimeral sulcus straight. Propodeum golden with green tinges (Fig. 424), smooth (Fig. 430); propodeal callus with two setae. Foreleg with coxa yellowish brown with base pale brown (Fig. 423), femur, tibia and tarsus yellow; mid- and hind legs yellow; midleg with first tarsomere 0.3× as long as length of tarsus. Forewing transparent, veins yellowish white and setae dark brown (Fig. 428); speculum closed; admarginal setae 10-13, arising from marginal vein and from membrane just below vein; radial cell setose; postmarginal vein 0.8× as long as stigmal vein; stigmal vein long and slender. Hind wing transparent, apex rounded (Fig. 428). Forewing WIP (Fig. 429) with apical ½ yellow and basal ½ blue, separated by a band of magenta, apical ½ also with a narrow blue band along upper margin.

Petiole yellow to yellowish brown. Gaster dark brown with golden, purple and green metallic tinges, smooth, elongate and 1.5–1.7× as long as length of mesosoma; 7^th^ tergite 0.1× as long as length of gaster.

*Male*. Length of body 1.2–1.6 mm. Features as in female except as follows. Antenna with scape yellowish brown with dorsal edge dark brown, to predominantly dark brown, pedicel pale brown, flagellum dark brown; pedicel + flagellum 2.5× as long as distance between eyes; flagellomeres 1–4 with scattered setae (Fig. 435); clava 1-segmented. Face purple metallic (Fig. 427); clypeus golden with green tinges; gena purple metallic; lower frons golden with purple tinges, with very weak reticulation; antennal scrobes parallel and join frontal suture separately; upper frons golden, with very weak reticulation; vertex purple metallic.

Mesoscutum black or golden with green tinges (Fig. 425). Scutellum 1.1× as long as wide. Dorsellum purple metallic (Fig. 425), 0.4× as long as wide (Fig. 434), and 0.5× as long as length of median propodeum. Propodeum golden (Fig. 425), smooth with some parts with very weak reticulation (Fig. 434). Legs with coxae dark brown; femora yellowish brown; tibiae and tarsi yellow. Forewing veins dark brown; admarginal setae 11–12, arising mainly from marginal vein; postmarginal vein 0.9× as long as stigmal vein.

Petiole dark brown. Gaster dark brown with a median white spot across tergites 1–3, smooth, 1.2× as long as length of mesosoma. Phallobase and aedeagus as in Fig. 499.

###### Hosts.

Associatedwith bracket fungi, possibly a Cecidomyiidae (Diptera) (Hansson 1996b).

###### Distribution.

Hungary (Graham 1963), Netherlands (**new record**), Sweden (**new record**), United Kingdom (Walker 1839); Canada (Hansson 1996b), USA (Hansson 1996b), Mexico (Hansson 1997), Costa Rica (Hansson 2004), Guatemala (Hansson 2004), Honduras (Hansson 2004) (Fig. 534).

###### Remarks.

The character-set present in this species (see diagnosis above) is unlike any other species in the genus, and the appearance of the male phallobase is also unique. These features makes it difficult to assess *Omphale erginnus* to any of the species-groups.

##### Omphale isander

(Walker)

http://species-id.net/wiki/Omphale_isander

Figures 438 –452 500 535

Cirrospilus isander Walker, 1839:326. Lectotype female in NMID, not examined.
Tetrastichus isander (Walker) Walker (1846).
Asecodes fimbriatus Jansson, 1955:87. Holotype female in LUZM, examined. Synonymized by Bouček and Askew 1968.
Eugerium isander (Walker) Graham (1959).
Omphale isander (Walker) Hansson (1996b).

###### Material.

**Type material.** **Holotype** female of *Asecodes fimbriatus*, type no. 131:1 in LUZM. **Additional material.** 51♀ 19♂: Finland 2♀ 2♂ (CH), Hungary 4♀ 5♂ (BMNH, CH), Norway 1♀ (BMNH), Russia 3♀ 2♂ (BMNH), Sweden 29♀ 10♂ (BMNH, CH, LUZM, NHRS), United Kingdom 12♀ (BMNH).

###### Diagnosis.

**Figures 438–444.**
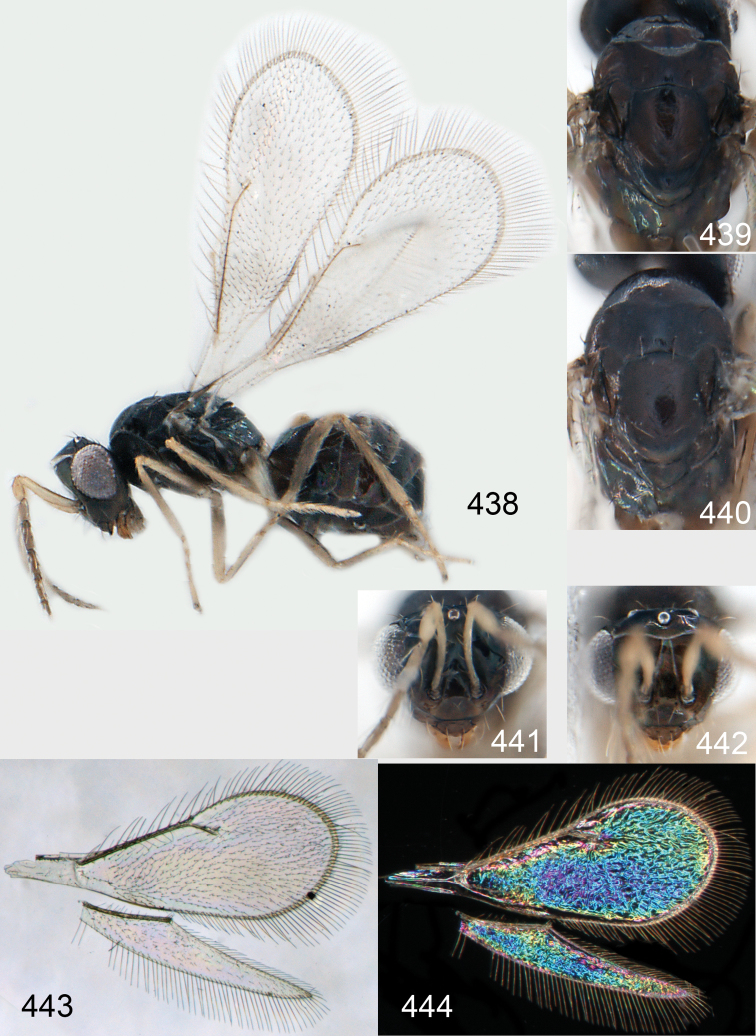
*Omphale isander*: **438** habitus in lateral view, female, length of specimen 0.9 mm **439** thoracic dorsum, female **440** thoracic dorsum, male **441** head in frontal view, female **442** head in frontal view, male **443** transparent wings, female **444** wing interference patterns, female.

**Figures 445–452.**
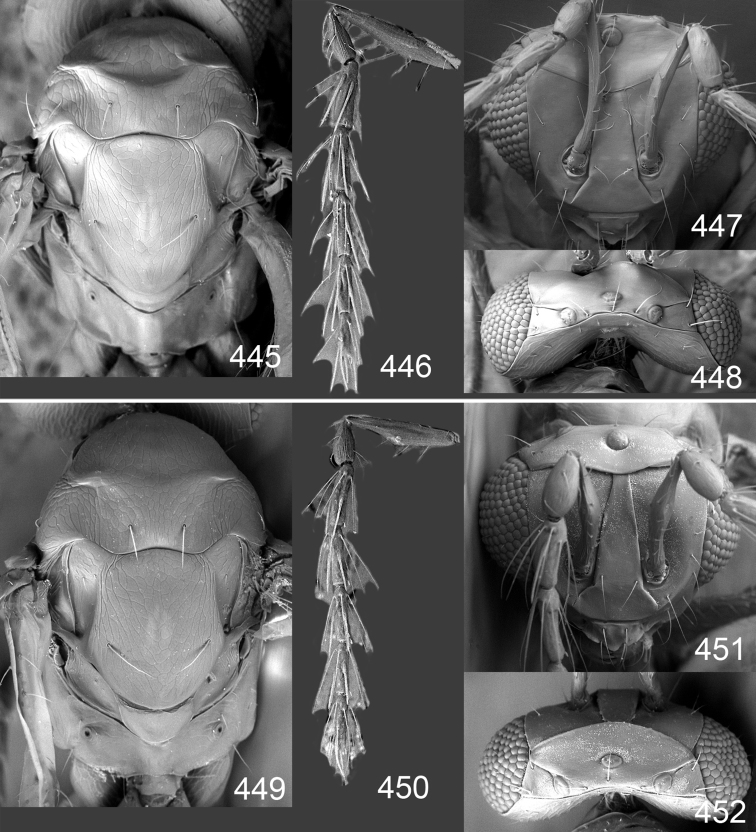
*Omphale isander*: **445** thoracic dorsum, female **446** antenna, female **447** head in frontal view, female **448** vertex, female **449** thoracic dorsum, male **450** antenna, male **451** head in frontal view, male **452** vertex, male.

Small species (0.7–1.1 mm); body dark brown with metallic tinges (Fig. 438); midlobe of mesoscutum with one pair of setae (posterior pair) (Figs 445, 449); forewing with long marginal fringe, e.g. setae along outer margin are 0.3× as long as width of wing, and a very short postmarginal vein (Fig. 443); head smooth (Figs 447, 448, 451, 452); vertex with distinct sulci between ocelli and eyes (Figs 448, 452); clypeus with median part of ventral margin drawn out into a rounded point (Figs 447, 451); male antenna with scape with basal 2/3 gradually expanding towards apical part and then abruptly narrowing off (Fig. 450).

###### Description.

*Female*. Length of body 0.7–1.1 mm. Antenna with scape and pedicel yellowish white; flagellum pale brown with metallic tinges; flagellum long and slender, pedicel + flagellum 2.6× as long as distance between eyes; first flagellomere 0.9× as long and about 1.2× as wide as second flagellomere (Fig. 446); flagellomeres with few long and scattered setae; longitudinal sensilla very long and setae-like but thicker and paler than setae; clava 1-segmented. Face dark brown with metallic tinges (Fig. 441), smooth (Fig. 447); clypeus dark brown with metallic tinges, smooth, rectangular but with lower margin protruding and with median part more or less pointed; 2.0× as wide as high; gena dark brown with metallic tinges; frontal cross-ridge absent; lower frons dark brown with metallic tinges, smooth, subtorular area smooth; interscrobal area smooth; antennal scrobes join frontal suture separately; frontal suture V-shaped; upper frons and vertex dark brown with metallic tinges, smooth (Fig. 448). Occipital margin with a carina (Fig. 448).

Mesoscutum dark brown with metallic tinges (Fig. 439) and engraved reticulation (Fig. 445), midlobe with one pair of setae (posterior pair); notauli as indistinct impressions in posterior ½. Scutellum dark brown with metallic tinges (Fig. 439), with engraved reticulation (Fig. 445); 1.2× as long as wide, with anterior margin smoothly curved forwards. Axillae dark brown with metallic tinges (Fig. 439). Dorsellum dark brown with metallic tinges (Fig. 439), smooth and convex (Fig. 445), 0.4× as long as wide, and 0.4× as long as length of median propodeum. Lateral mesosoma dark brown with metallic tinges (Fig. 438); transepimeral sulcus straight. Propodeum dark brown with metallic tinges (Fig. 439), smooth (Fig. 445); propodeal callus with two setae. Fore- and midlegs with coxae dark brown with metallic tinges (Fig. 438), femora pale brown in basal ⅔ and yellowish white in apical ⅓, tibiae and tarsi yellowish white; midleg with first tarsomere 0.3× as long as length of tarsus; hind leg with coxa white with base dark brown, femur dark brown with metallic tinges, tibia and tarsus yellowish white. Forewing transparent, veins yellowish white and setae dark brown (Fig. 443); speculum closed; admarginal setae 9–11, arising from marginal vein; radial cell bare; postmarginal vein rudimentary; stigmal vein long and narrow; marginal fringe with long setae, e.g. setae along outer margin are 0.3× as long as width of wing. Hind wing transparent, apex pointed (Fig. 443). Forewing WIP (Fig. 444) unicoloured in blue.

Petiole white. Gaster dark brown with metallic tinges, ovate and 1.2–1.3× as long as length of mesosoma; 7^th^ tergite very short and usually hidden under 6^th^ tergite.

*Male*. Length of body 0.9–1.1 mm. Features as in female except as follows. Antenna with scape with basal ⅔ gradually expanding towards apical part then abruptly narrowing off (Fig. 450); pedicel + flagellum 3.0× as long as distance between eyes; first flagellomere 1.0× as long as second flagellomere; flagellomeres 1–4 with setae confined to a basal whorl; clava 1-segmented.

Scutellum 1.3–1.4× as long as wide. Dorsellum 0.5× as long as wide, and 0.6× as long as length of median propodeum. Legs yellowish brown to yellowish white, except pale brown hind femur and base of all coxae. Forewing admarginal setae 5–7.

Petiole white. Gaster dark brown with metallic tinges, posteromedian part of first tergite and anteromedian part of second with a small white spot, 1.1–1.3× as long as length of mesosoma. Phallobase and aedeagus as in Fig. 500.

###### Host.

From *Mycodiplosis* sp. (Diptera: Cecidomyiidae) feeding on leaf rust on *Populus* (Kamijo 1986).

###### Distribution.

Czech Republic (Peck et al. 1964), Finland (**new record**), France (Erdös 1958), Hungary (Jansson 1955), Ireland (Walker 1839), Moldova (Bouček 1965), Norway (**new record**), Russia (**new record**), Sweden (Jansson 1955), United Kingdom (Walker 1839); Japan (Kamijo 1986) (Fig. 535).

###### Remarks.

The lectotype of *Cirrospilus isander* in NMID has not been available for examination, and the interpretation of this species is based on the information in Graham (1959) and the type of *Asecodes fimbriatus*. *Omphale isander* is a very characteristic species unlike any other species in the genus and therefore difficult to assign to a specific group. In connection with the synonymization of *Eugerium* Graham with *Omphale*, Hansson (1996b) discussed *Omphale isander* in detail.

##### Omphale lugens

(Nees)

http://species-id.net/wiki/Omphale_lugens

Figures 2, 6 453 –467 501 536

Eulophus lugens Nees, 1834:176. Neotype female in RMNH, designated here.
Entedon navius Walker, 1839:92. Lectotype female in BMNH, examined. Synonymized by Bouček and Askew (1968:134).
Entedon coactus Ratzeburg, 1848:167. Type material lost (Graham 1963). Synonymized by Bouček and Askew (1968:134).
Secodes fagi Förster, 1856:81. Type material not located. Synonymized by Bouček and Askew (1968:134).
Omphale lugens (Nees), Bouček and Askew (1968:134).

###### Designation of neotype.

Apart from specimens collected by Nees now in the Haliday collection in Oxford, all specimens of the Nees collection have been destroyed (Graham 1988). As there are no specimens of *Eulophus lugens* in Oxford (Graham 1988) this species is therefore not fixed by any type material. To maintain a stable nomenclature a neotype for *Eulophus lugens* is designated here. As neotype a female from Germany: Bavaria, Obersdorf, reared from *Mikiola fagi* is selected. The material from the original description was from Sickershausen, which is in Bavaria in Germany, collected from a window. The neotype agrees well with the original description.

###### Material.

**Type material.** Neotype female of *Eulophus lugens* in RMNH, lectotype female of *Entedon navius*, type no. 5.2035 in BMNH. **Additional material.** 439♀ 22♂: Austria 1♀ 1♂ (RMNH), Croatia 17♀ 3♂ (BMNH), France 4♀ 1♂ (RMNH), Germany 1♀ 1♂ (RMNH), Greece 2♀ 2♂ (RMNH), Hungary 1♀ (BMNH), Netherlands 10♀ 3♂ (BMNH, RMNH), Sweden 226♀ 1♂ (BMNH, CH, LUZM, NHRS, RMNH), United Kingdom 177♀ 10♂ (BMNH).

###### Diagnosis.

**Figures 453–459.**
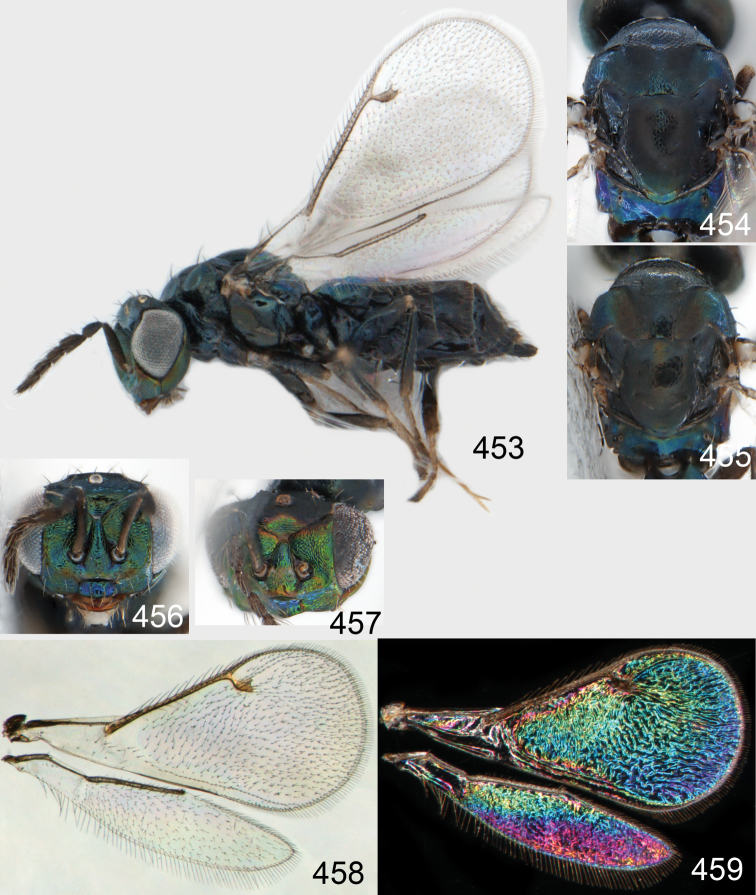
*Omphale lugens*: **453** habitus in lateral view, female, length of specimen 1.5 mm **454** thoracic dorsum, female **455** thoracic dorsum, male **456** head in frontal view, female **457** head in frontal view, male **458** transparent wings, female **459** wing interference patterns, female.

Female flagellum short (Fig. 453), pedicel and flagellum 1.3× as long as distance between eyes; legs completely dark (Fig. 453); wing shape characteristic with a short, high and rounded forewing (Fig. 458); stigmal vein enlarged and elongate (Fig. 458); mesoscutum and scutellum dark and drab, contrasting against bright bluish green metallic propodeum (Figs 454, 455); male genitalia very different from other *Omphale* species: aedeagus with very long apodemes (Fig. 501); phallobase with volsellae strongly protruding and with volsellar setae at apex (Fig. 501), digitus drawn out (downwards in illustration) with a terminal hook.

###### Description.

*Female*. Length of body 1.0**–**1.7 mm. Antenna dark brown; pedicel + flagellum 1.3× as long as distance between eyes; first flagellomere 1.4× as long and 1.3× as wide as second flagellomere (Fig. 461); flagellomeres 1–4 ventrally with one set of setae, attached at base and reaching beyond apex of flagellomere attached to; clava 2-segmented. Face golden (Fig. 456) or golden red, strigose-reticulate (Fig. 462); cly-peus golden green, smooth, rectangular and 2.5× as wide as high; gena golden purple; lower frons golden green to green metallic, with raised reticulation; antennal scrobes join frontal suture separately; frontal suture V-shaped; upper frons golden green with weak reticulation; vertex black with golden green tinges, with raised and weak reticulation (Fig. 463). Occipital margin rounded (Fig. 463).

**Figures 460–467.**
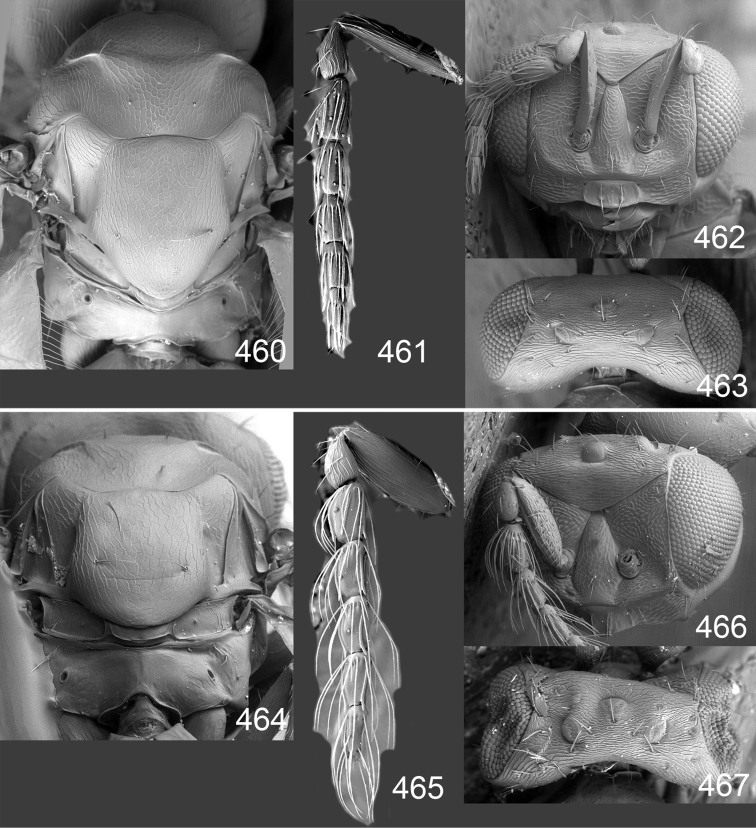
*Omphale lugens*: **460** thoracic dorsum, female **461** antenna, female **462** head in frontal view, female **463** vertex, female **464** thoracic dorsum, male **465** antenna, male **466** head in frontal view, male **467** vertex, male.

Mesoscutum golden green, purple metallic, to dark brown with green to purple metallic tinges (Fig. 454), with raised reticulation (Fig. 460), midlobe with two pairs of setae; notauli as indistinct impressions in posterior ½. Scutellum purple metallic, to dark brown with green to purple metallic tinges (Fig. 454), with raised reticulation (Fig. 460); 1.0× as long as wide, with anterior margin smoothly curved forwards. Axillae bluish green metallic (Fig. 454). Dorsellum golden green (Fig. 454), with weak reticulation and slightly convex (Fig. 460), 0.3× as long as wide, and 0.6× as long as length of median propodeum. Lateral thorax golden green (Fig. 453); transepimeral sulcus curved forwards. Propodeum bright bluish green metallic (Fig. 454), smooth (Fig. 460); propodeal callus with two setae. Coxae and femora dark brown with bluish green metallic tinges (Fig. 453); tibiae and tarsi dark brown. Forewing transparent, veins yellowish brown and setae dark brown (Fig. 458); speculum closed; admarginal setae 3–4, arising from marginal vein; radial cell bare; stigmal vein enlarged and elongate. Hind wing transparent, apex rounded (Fig. 458). Forewing WIP (Fig. 459) unicoloured in blue with a narrow area just behind marginal vein with narrow bands in yellow and magenta.

Petiole dark brown. Gaster with first tergite bluish green metallic, remaining tergites dark brown with metallic tinges, elongate and 1.2–1.5× as long as length of mesosoma; 7^th^ tergite 0.06× as long as length of gaster.

*Male*. Length of body 1.1**–**1.4 mm. Features as in female except as follows. Antenna with pedicel + flagellum 1.9× as long as distance between eyes; flagellomeres 1–4 with a single basal whorl of setae (Fig. 465). Face, clypeus and frons blue metallic (Fig. 457).

Phallobase and aedeagus as in Fig. 501.

###### Hosts.

*Mikiola fagi* (Dziurzynski 1961), *Contarinia tiliarum* & *Dasyneura alni* (Gij- swijt 1976), *Placochela nigripes* (**new record**), all hosts are Diptera: Cecidomyiidae.

###### Distribution.

Austria (Kirchner 1867), Azerbaidzhan (Bouček and Askew 1968), Croatia (**new record**), Czech Republic (Kirchner 1854), France (**new record**), Germany (Nees 1834), Greece (**new record**), Hungary (Erdös 1956), Moldova (Bouček 1965), Netherlands (Gijswijt 1976), Poland (Dziurzynski 1961), Sweden (Thomson 1878), Switzerland (Ratzeburg 1848); United Kingdom (Walker 1839) (Fig. 536).

###### Remarks.

Externomorphologically *Omphale lugens* fits best in the same group as *Omphale phruron*, something also suggested by Graham (1963). However, if male genitalia are considered, a character-set unknown to Graham, then *Omphale lugens* is something quite unique and far removed from the species in the *phruron*-group.

##### Omphale melina

Yefremova & Kriskovich

http://species-id.net/wiki/Omphale_melina

Figures 468–472 537

Omphale melinum Yefremova & Kriskovich, 1994:247. Holotype female in ZISP, not examined.

###### Material. 

**Type material.** Paratype female in ZISP.

###### Diagnosis.

**Figures 468–472.**
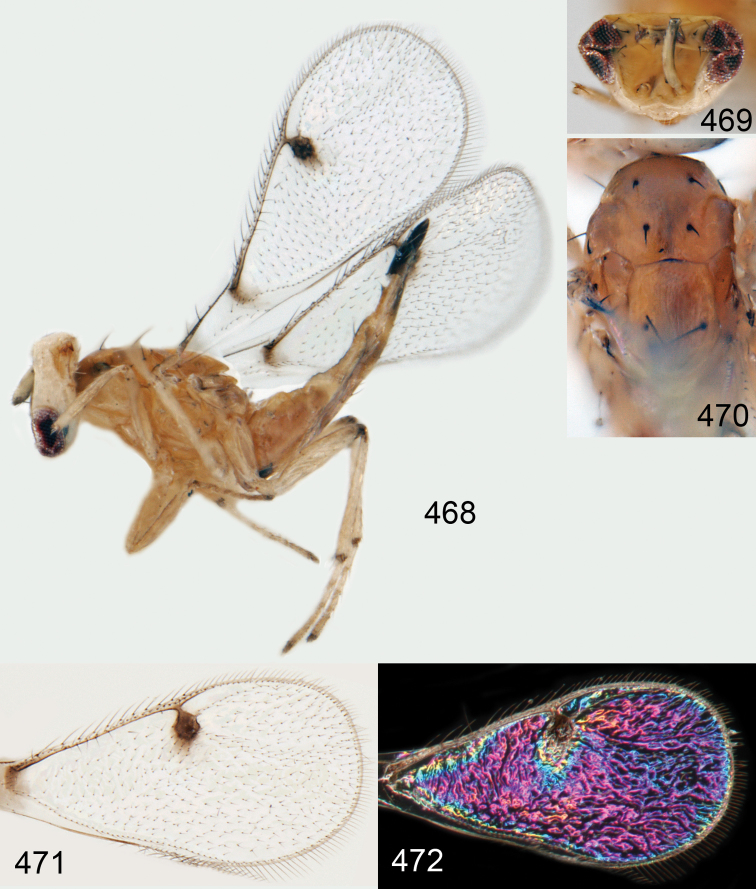
*Omphale melina*, female: **468** habitus in lateral view, length of specimen 1.5 mm **469** head in frontal view **470** thoracic dorsum **471** transparent wings **472** wing interference patterns.

Yellow non-metallic species with an enlarged stigmal vein and with area around stigmal vein infuscate (Figs 468–471).

###### Description.

*Female*. Length of body 1.5 mm. Antenna with scape yellowish with dorsal edge dark brown (Fig. 469); remaining parts of antenna missing in single examined specimen. Entire head yellowish white (Figs 468, 469). Face strigose; clypeus yellow (Fig. 469), strigose, semicircular, 1.3× as wide as high; lower frons with raised reticulation, subtorular area smooth; interscrobal area reticulate; antennal scrobes join on frontal suture; upper frons and vertex reticulate. Occipital margin rounded.

Mesosoma yellow with setae on thoracic dorsum black (Fig. 470). Mesoscutum with engraved weak reticulation, midlobe with two pairs of setae (Fig. 470); notauli as indistinct impressions. Scutellum with engraved weak reticulation; 1.2× as long as wide, with anterior margin weakly curved forwards. Dorsellum smooth and slightly convex, 0.4× as long as wide, and 0.8× as long as length of median propodeum. Propodeum smooth; propodeal callus with two setae. Lateral mesosoma yellowish white (Fig. 468); transepimeral sulcus curved forwards. Legs yellowish white (Fig. 468); midleg with first tarsomere 0.4× as long as length of tarsus. Forewing transparent with infuscate areas around stigmal vein and below base of marginal vein (Fig. 471), veins yellowish white and setae dark brown; speculum closed; admarginal setae 5, arising from marginal vein; radial cell bare; postmarginal vein 1.0× as long as stigmal vein; stigmal vein enlarged and circular. Hind wings missing in single examined specimen. Forewing WIP (Fig. 472) unicoloured purple with a small round area just below stigmal vein in blue and yellow.

Petiole yellow. Gaster pale brown with five yellow cross bands, 7^th^ tergite and apical parts of ovipositor sheaths black metallic; elongate (Fig. 468) and 1.7× as long as length of mesosoma; 7^th^ tergite 0.1× as long as length of gaster.

*Male*. Unknown.

###### Hosts.

Unknown.

###### Distribution.

Russia (Yefremova and Kriskovich 1994) (Fig. 537).

###### Remarks.

The examined paratype specimen is damaged. It lacks the entire right antenna and of the left antenna only the scape remains; both hind wings are missing; the gaster has been gnawed upon and parts of the left hand side are gone. In spite of this the species is easy to recognize through its non-metallic body and enlarged stigmal vein. The species is difficult to assign to a specific group, when males are found these may hold morphological clues as to its placement.

##### Omphale ochra sp. n.

urn:lsid:zoobank.org:act:B0552C5D-FAEF-43D8-9A81-37354532D79A

http://species-id.net/wiki/Omphale_ochra

Figures 398–401 473–477 538

###### Material.

**Holotype** female (BMNH), glued to a card, labelled “SWEDEN: Skåne, Häckeberga, castle, 55°35'N, 13°26'E, 5.vii.2006, C. Hansson & E. Shevtsova”. **Paratype.** 1♀ with same label data as holotype (BMNH).

###### Diagnosis.

**Figures 473–477.**
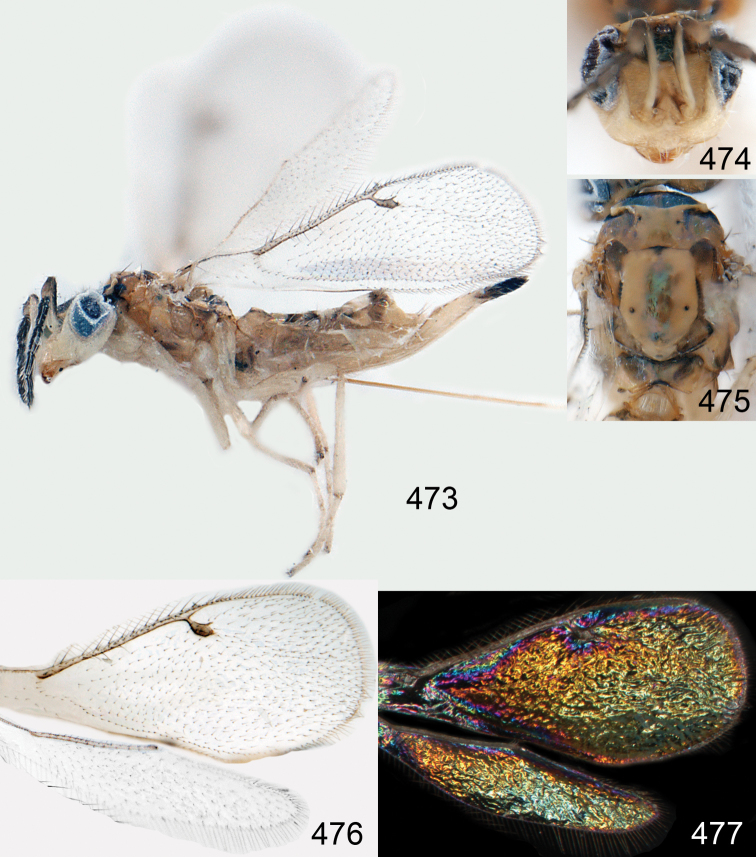
*Omphale ochra*, female: **473** habitus in lateral view, length of specimen 1.8 mm **474**  head in frontal view **475** thoracic dorsum **476** transparent wings **477** wing interference patterns.

A pale non metallic species, predominantly yellowish brown to yellowish white (Figs 473–475); female gaster very long (Fig. 473), 2× as long as length of mesosoma.

###### Description.

*Female*. Length of body 1.6–1.8 mm. Antenna with scape yellowish brown with dorsal margin dark brown, pedicel pale brown, flagellum dark brown; pedicel + flagellum 2.1× as long as distance between eyes; first flagellomere 1.3× as long and 1.1× as wide as second flagellomere (Fig. 399); flagellomeres 2–4 ventrally with a single set of setae attached close to base and reaching beyond apex of flagellomere attached to; clava 2-segmented. Face yellowish-white (Fig. 474), strigose-reticulate (Fig. 400); clypeus yellowish white, smooth, semicircular, 1.2× as wide as high; gena yellowish white; lower frons yellowish white with antennal scrobes dark brown, with raised and weak reticulation; antennal scrobes join frontal suture separately; frontal suture V-shaped; upper frons dark brown with metallic tinges, with raised and weak reticulation; vertex yellowish brown, with very weak reticulation (Fig. 401). Occipital margin rounded (Fig. 401).

Mesoscutum with anterior ½ golden green with a median yellowish brown stripe, posterior ½ yellowish brown (Fig. 475), with very weak reticulation (Fig. 398), midlobe with two pairs of setae; notauli as indistinct depressions in posterior ½. Scutellum yellowish brown with a median dark brown longitudinal stripe (Fig. 475), with very weak engraved reticulation (Fig. 398), 1.2× as long as wide, with anterior margin smoothly curved forwards. Axillae with anterior ½ dark brown with metallic tinges, posterior ½ yellowish brown (Fig. 475). Dorsellum yellowish brown (Fig. 475), convex and smooth (Fig. 398), 0.3× as long as wide, and 0.6× as long as length of median propodeum. Entire lateral mesosoma yellowish brown (Fig. 473); transepimeral sulcus curved forwards. Propodeum yellowish brown with median part dark brown (Fig. 475), smooth (Fig. 398); propodeal callus with two setae. Legs yellowish white (Fig. 473); midleg with first tarsomere 0.4× as long as length of tarsus. Forewing transparent, veins pale brown, setae dark brown (Fig. 476); speculum closed; admarginal setae 5, arising from ventral marginal vein; radial cell bare; postmarginal vein 0.7× as long as stigmal vein; stigmal vein slightly enlarged. Hind wing transparent, apex rounded (Fig. 476). Forewing WIP (Fig. 477) unicoloured in yellow with narrow bands in magenta and blue close to foremargin.

Petiole yellowish brown. Gaster yellowish brown with posterior margin of tergites brown, apical parts of ovipositor sheaths dark brown (Fig. 473), smooth; elongate and 2.0× as long as length of mesosoma; 7^th^ tergite 0.2× as long as length of gaster.

*Male*. Unknown.

**Figures 478–483.**
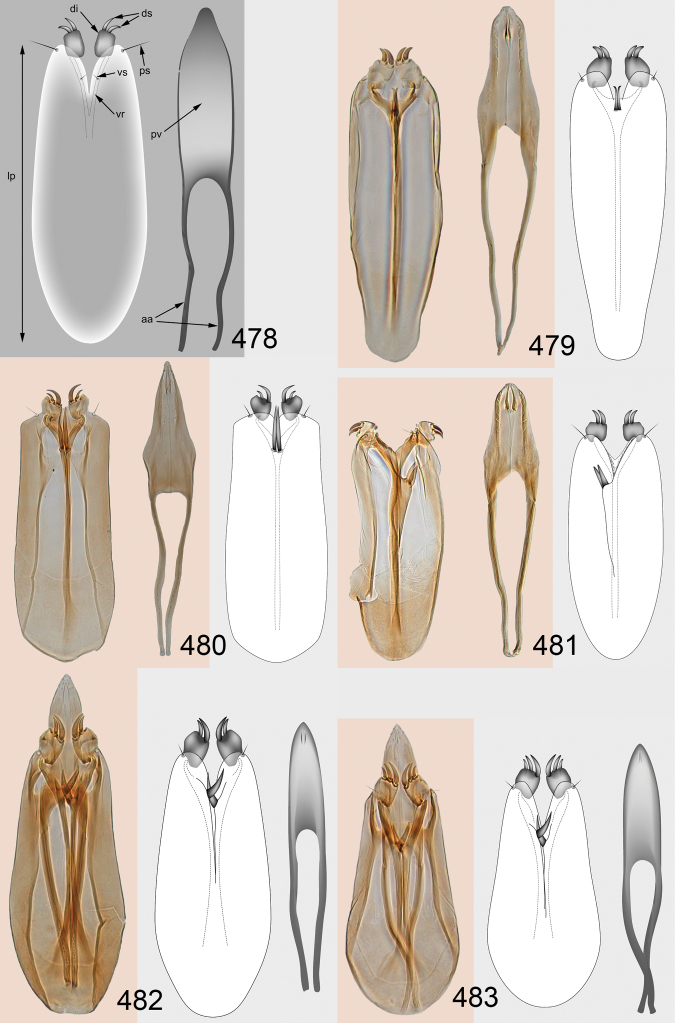
Male genitalia (phallobase+aedeagus): **478** *Entedon fufius* (Walker) (Hymenoptera: Eulophidae: Entedoninae), phallobase to the left, aedeagus to the right, abbreviations: aa = aedeagal apo-demes, di = digitus, ds = digital spines, lp = length of phallobase, ps = parameral setae, pv = penis valve, vr = volsellar ridge, vs = volsellar setae **479–483** *Omphale* spp.: **479** *Omphale admirabilis*, length of phallobase 0.30 mm **480** *Omphale telephe*, length of phallobase 0.32 mm **481** *Omphale versicolor*, length of phallobase 0.31 mm **482** *Omphale chryseis*, length of phallobase 0.25 mm **483** *Omphale cornula*, length of phallobase 0.24 mm.

**Figures 484–489.**
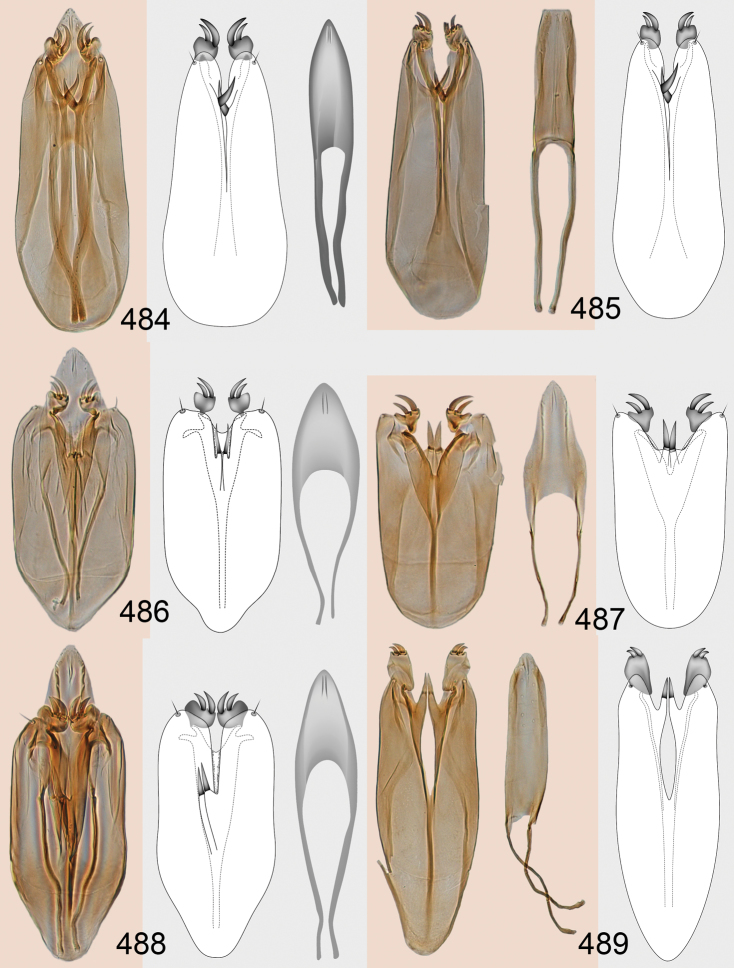
Male genitalia (phallobase and aedeagus) *Omphale* spp.: **484** *Omphale salicis*, length of phallobase 0.27 mm **485** *Omphale theana*, length of phallobase 0.26 mm **486** *Omphale brevis*, length of phallobase 0.17 mm **487** *Omphale clymene*, length of phallobase 0.20 mm **488** *Omphale euphorbiae*, length of phallobase 0.21 mm **489** *Omphale incognita*, length of phallobase 0.27 mm.

**Figures 490–494.**
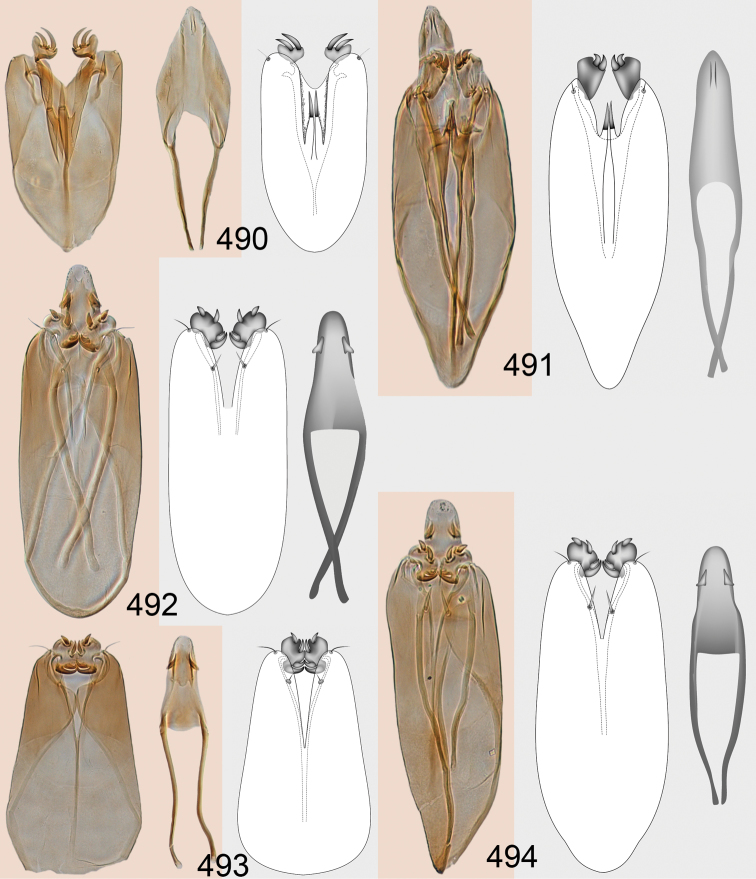
Male genitalia (phallobase and aedeagus) *Omphale* spp.: **490** *Omphale phruron*, length of phallobase 0.19 mm **491** *Omphale tenuicornis*, length of phallobase 0.24 mm **492** *Omphale aethiops*, length of phallobase 0.30 mm **493** *Omphale connectens*, length of phallobase 0.22 mm **494** *Omphale lugubris*, length of phallobase 0.27 mm.

**Figures 495–499.**
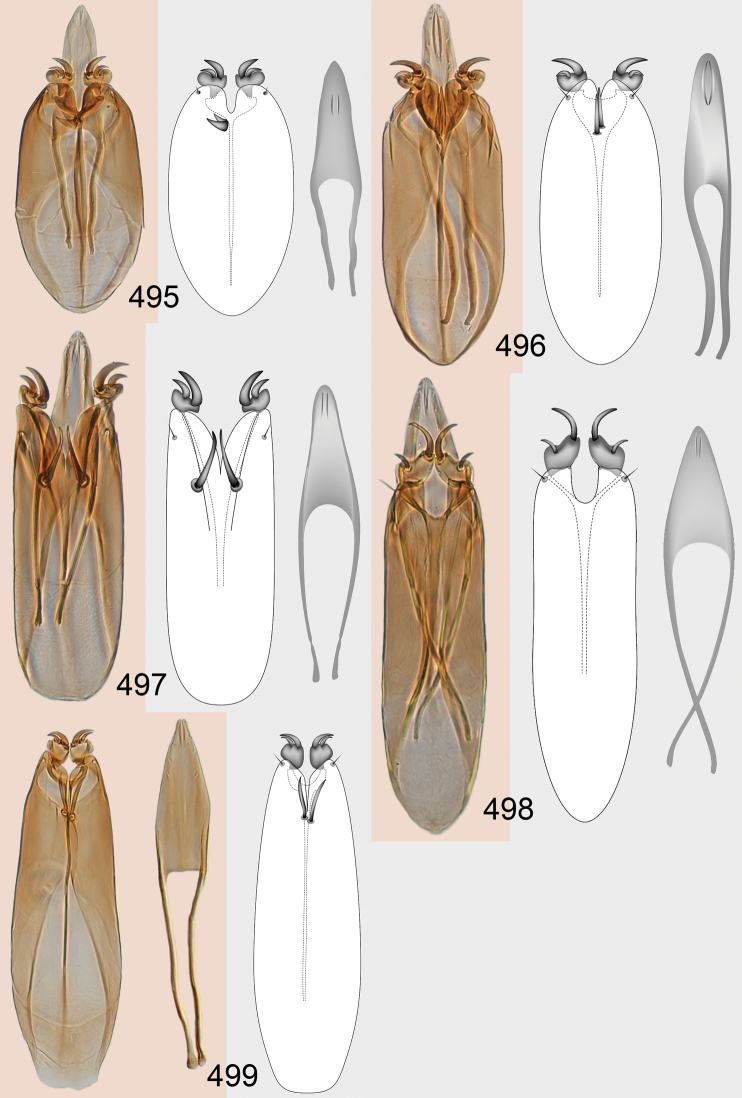
Male genitalia (phallobase and aedeagus) *Omphale* spp.: **495** *Omphale clypealis*, length of phallobase 0.21 mm **496** *Omphale parma*, length of phallobase 0.22 mm **497** *Omphale sulciscuta*, length of phallobase 0.24 mm **498** *Omphale obscura*, length of phallobase 0.22 mm **499** *Omphale erginnus*, length of phallobase 0.22 mm.

**Figures 500–501.**
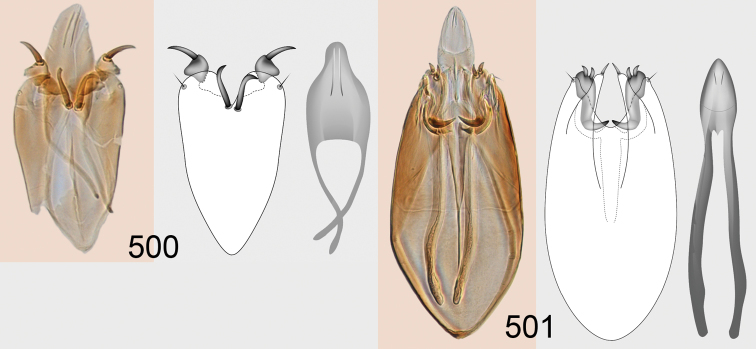
Male genitalia (phallobase and aedeagus) *Omphale* spp.: **500** *Omphale isander*, length of phallobase 0.13 mm **501** *Omphale lugens*, length of phallobase 0.26 mm.

###### Host.

Unknown.

###### Distribution.

Sweden (Fig. 538).

**Figures 502–517.**
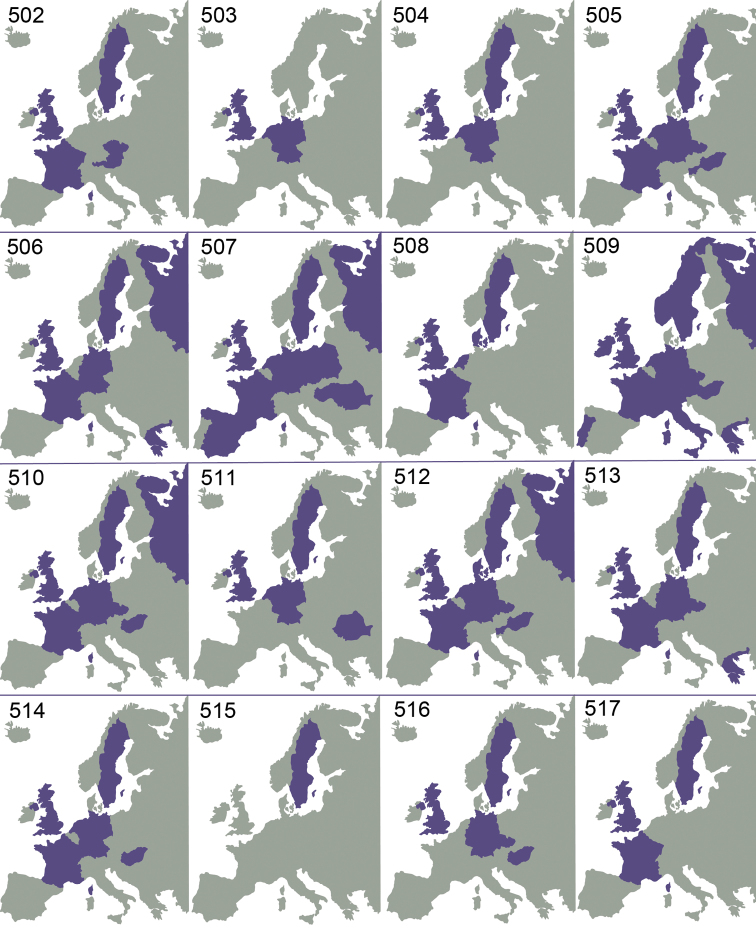
*Omphale* spp. distribution in Europe: **502** *Omphale admirabilis* **503** *Omphale breviventris* **504** *Omphale telephe* **505** *Omphale versicolor* **506** *Omphale acuminata* **507** *Omphale chryseis* **508** *Omphale cornula* **509** *Omphale salicis* **510** *Omphale theana* **511** *Omphale brevis* **512** *Omphale clymene* **513** *Omphale euphorbiae* **514** *Omphale incognita* **515** *Omphale lydia* **516** *Omphale matrana* **517** *Omphale nitens*.

**Figures 518–533.**
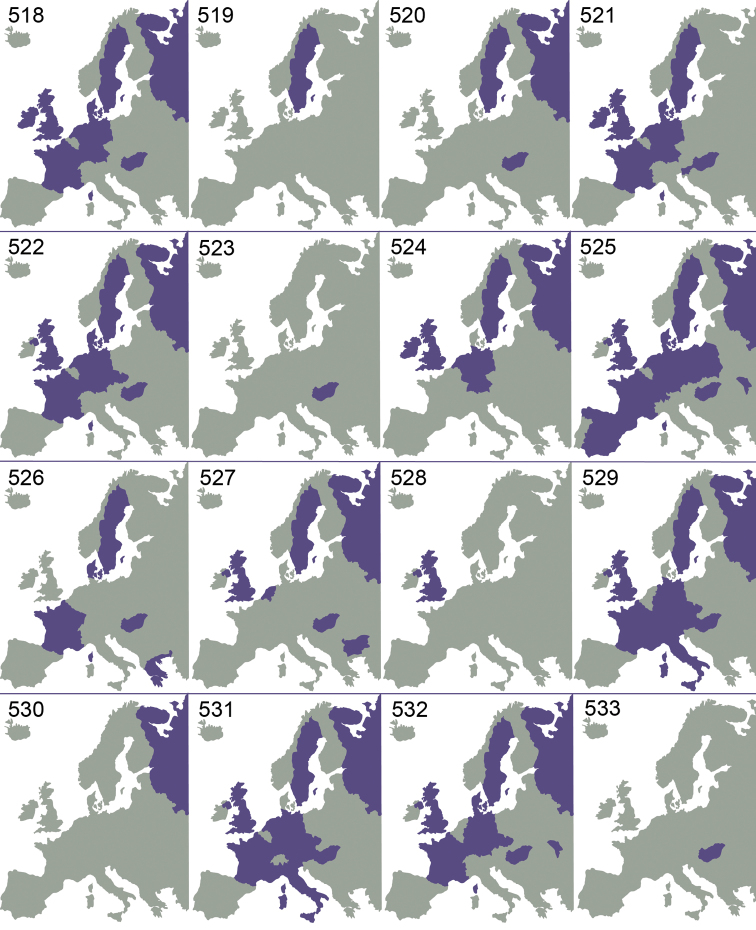
*Omphale* spp. distribution in Europe: **518** *Omphale phruron* **519** *Omphale sti* **520** *Omphale tenuicornis* **521** *Omphale aethiops* **522** *Omphale connectens* **523** *Omphale dolichura* **524** *Omphale lugubris* **525** *Omphale clypealis* **526** *Omphale parma* **527** *Omphale brevibuccata* **528** *Omphale erugata* **529** *Omphale obscura* **530** *Omphale rossica* **531** *Omphale rubigus* **532** *Omphale sulciscuta* **533** *Omphale aceris*.

**Figures 534–538.**
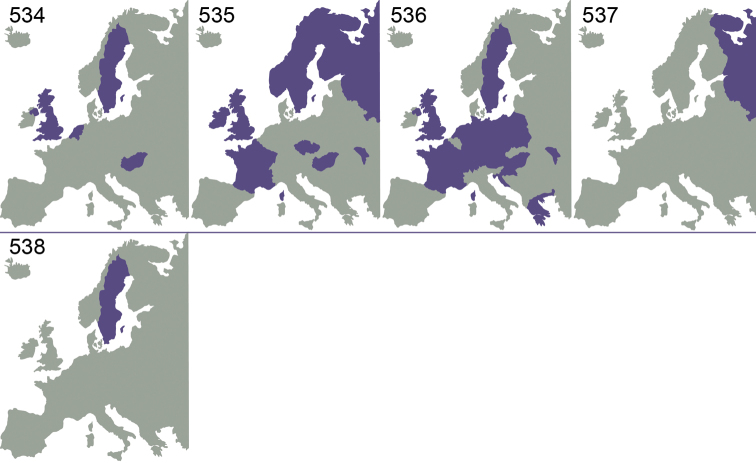
*Omphale* spp. distribution in Europe: **534** *Omphale erginnus* **535** *Omphale isander* **536** *Omphale lugens* **537** *Omphale melina* **538** *Omphale ochra*.

**Figures 539–540.**
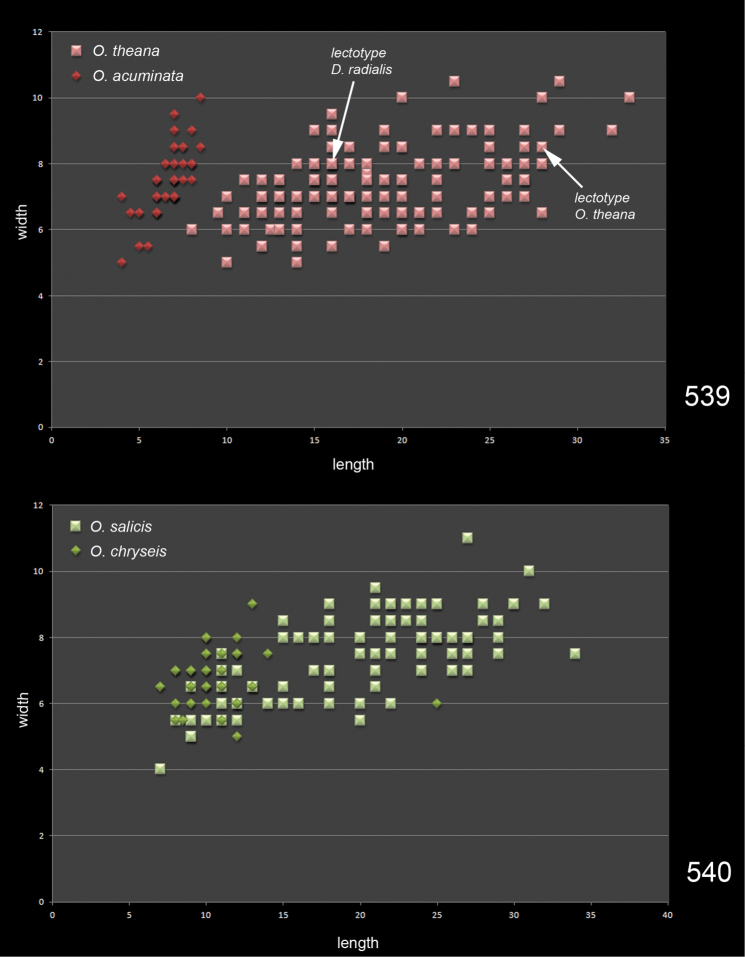
Scatter diagrams to compare length/width of 7th gastral tergite in female: **539** *Omphale acuminata* (red diamonds), n = 59, mean = 0.9 and *Omphale theana* (pink squares), n = 156, mean = 1.3 **540** *Omphale chryseis* (green diamonds), n = 53, mean = 1.6 and *Omphale salicis* (green squares), n = 109, mean = 2.6.

###### Etymology.

From the Greek *ochros* = pale yellow, referring to the predominant colour of the body.

###### Remarks.

Both type specimens are shrivelled, thus measurements and ratios are not exact. Known only from shrivelled females this species is difficult to place to group. Characters involving the setation in forewing suggest an affinity to either *phruron*- or *salicis*-group. When males are found a firmer idea on this can be established.

####### Other European species associated with *Omphale*

Two additional species from Europe, *Pholema microstoma* Graham and *Eugerium orbatum* Szelényi, were associated with *Omphale* prior to this revision. Their association with Omphale is because the genus they were originally described in, *Eugerium* and *Pholema* respectively, were synonymized with *Omphale*. However, new data presented here suggest a different classification of these species.

##### Genus Pholema

Graham stat. rev.

http://species-id.net/wiki/Pholema

###### Remarks. 

Graham (1963) described genus *Pholema* to include the single species *microstoma*, which was described in the same paper. Graham motivated the new genus because he regarded some morphological features present in *Pholema*: small clypeus, large malar space, small mouth opening, too discordant for an *Omphale* species. Schauff (1991) synonymized *Pholema* with *Omphale* because he found the characters mentioned by Graham too variable and/or overlapping with some *Omphale* species.

**Figures 541–543.**
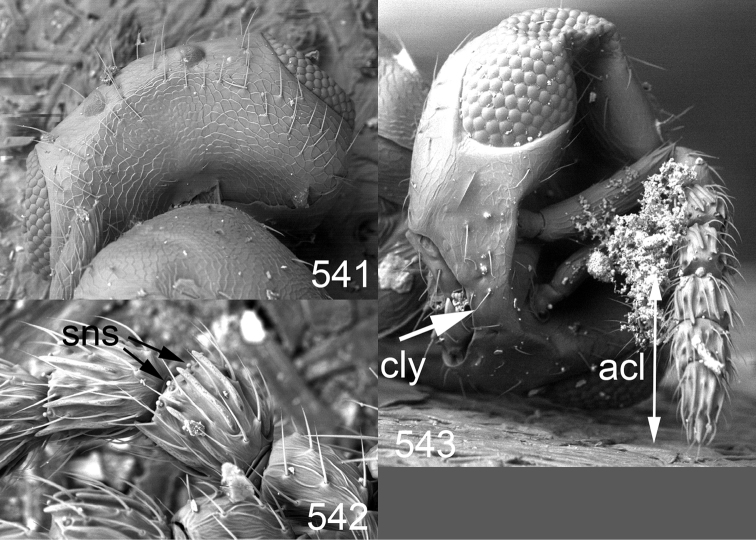
*Neochrysocharis microstoma*, female: **541** occiput **542** flagellomere 1 with sensillae (= sns) **543** head in fronto-ventral view, acl = 3-segmented antennal clava, cly = clypeus.

The type species and the only known species of *Pholema*, *Pholema microstoma*, has the following characters important for classification on genus level: clypeus undelimited (Fig. 543), occiput without a groove or fold between occipital margin and occipital foramen (Fig. 541), sensilla ampullacea (peglike sensilla) on flagellomeres short and symmetric (Fig. 542), forewing with postmarginal vein 0.8× as long as stigmal vein, ovipositor very short – gaster short and rotund and ovipositor only ½ as long as length of gaster, male genitalia with volsellar setae as “normal” thin setae. Some of these characters disagree with a placement of *Pholema microstoma* in *Omphale*: undelimited clypeus, antennal sensilla symmetric, and perhaps the most critical – not having enlarged volsellar setae in male genitalia, the sole autapomorphy for *Omphale*. Habitually *Pholema microstoma* looks like a *Neochrysocharis* Kurdjumov, mainly because of the distinct and 3-segmented antennal clava and the short postmarginal vein, and some additional characters also agree with a placement in *Neochrysocharis* (undelimited clypeus, symmetric antennal sensilla). Absence of an occipital groove/fold is very unusual in *Neochrysocharis* but it is absent in at least one other species, *Neochrysocharis albiscapus* Erdös. Furthermore, this character state is variable within *Neochrysocharis*, some species have a complete fold between occipital margin and occipital foramen, while others only have a short fold close to the occipital margin, and as mentioned some species lack it altogether. Therefore the absence of such a fold does not justify a separate genus for *Pholema microstoma*. The short ovipositor, not present in other *Neochrysocharis* species (nor in any *Omphale* species), is probably an adaptation to a close access of the host, and doubtfully of such a value that it justifies a separate genus. Another option is to place *Pholema microstoma* in *Asecodes* Förster, but some critical characters disagree with such a placement. *Pholema microstoma* has symmetrical antennal sensilla, a distinct 3-segmented antennal clava and lacks a fold or groove on occiput; *Asecodes* species have asymmetric antennal sensilla, a 1-segmented antennal clava and a strong groove between occipital margin and occipital foramen. All things considered *Pholema microstoma* is a *Neochrysocharis* and *Pholema* is thus a synonym of *Neochrysocharis*.

##### Neochrysocharis microstoma

(Graham) comb.n.

http://species-id.net/wiki/Neochrysocharis_microstoma

Figures 541–543

Pholema microstoma Graham, 1963:268. Holotype female in OUMNH, examined.
Omphale microstoma (Graham), Schauff (1991).

###### Material.

**Type material.** **Holotype** female, type no. 1300 in OUMNH. **Additional material.** 11♀ 6♂: France 1♀ (CH), Germany 1♀ 5♂ (BMNH, CH), Hungary 3♀ 1♂ (BMNH, CH), Russia 1♀ (CH), Slovenia 2♀ (RMNH), Sweden 2♀ (CH, LUZM), United Kingdom 1♀ (BMNH).

###### Diagnosis. 

Female antenna with a distinct and 3-segmented clava (Fig. 543); sensilla ampullacea (peglike sensilla) short and symmetric (Fig. 542); clypeus undelimited (Fig. 543); occiput without median groove from occipital margin to occipital foramen (Fig. 541); ovipositor very short, reaching half the length of gaster.

###### Description. 

See Graham (1963).

###### Host.

*Cassida deflorata* Suffrian (Coleoptera: Chrysomelidae) (**new record**).

###### Distribution.

France (**new record**), Germany (**new record**), Hungary (**new record**), Russia (**new record**), Slovenia (**new record**), Sweden (Hansson 1991), United Kingdom (Graham 1963).

##### Eugerium orbatum

Szelényi

http://species-id.net/wiki/Eugerium_orbatum

###### Remarks.

*Eugerium orbatum* was originally described on material from Hungary (Szelényi 1978), and was later transferred to *Omphale* (Noyes 2001). The male holotype of *Eugerium orbatum* (type no. 6011 in HNHM, examined) is shrivelled, but is recognizeable as a small male of *Asecodes congruens* (Nees) and is here established as a synonym of that species.

## Supplementary Material

XML Treatment for Omphale

XML Treatment for Omphale admirabilis

XML Treatment for Omphale breviventris

XML Treatment for Omphale telephe

XML Treatment for Omphale versicolor

XML Treatment for Omphale acuminata

XML Treatment for Omphale chryseis

XML Treatment for Omphale cornula

XML Treatment for Omphale salicis

XML Treatment for Omphale theana

XML Treatment for Omphale brevis

XML Treatment for Omphale clymene

XML Treatment for Omphale euphorbiae

XML Treatment for Omphale incognita

XML Treatment for Omphale lydia

XML Treatment for Omphale matrana

XML Treatment for Omphale nitens

XML Treatment for Omphale phruron

XML Treatment for Omphale sti

XML Treatment for Omphale tenuicornis

XML Treatment for Omphale aethiops

XML Treatment for Omphale connectens

XML Treatment for Omphale dolichura

XML Treatment for Omphale lugubris

XML Treatment for Omphale acamas

XML Treatment for Omphale aetius

XML Treatment for Omphale betulicola

XML Treatment for Omphale coilus

XML Treatment for Omphale epaphus

XML Treatment for Omphale grahami

XML Treatment for Omphale marica

XML Treatment for Omphale phaola

XML Treatment for Omphale varipes

XML Treatment for Omphale clypealis

XML Treatment for Omphale parma

XML Treatment for Omphale brevibuccata

XML Treatment for Omphale erugata

XML Treatment for Omphale obscura

XML Treatment for Omphale rossica

XML Treatment for Omphale rubigus

XML Treatment for Omphale sulciscuta

XML Treatment for Omphale aceris

XML Treatment for Omphale erginnus

XML Treatment for Omphale isander

XML Treatment for Omphale lugens

XML Treatment for Omphale melina

XML Treatment for Omphale ochra

XML Treatment for Pholema

XML Treatment for Neochrysocharis microstoma

XML Treatment for Eugerium orbatum
